# Health Outcomes in Children and Adolescents With Overweight or Obesity Exposed to Physical Activity Interventions: An Umbrella Review Covering Over 1200 Trials

**DOI:** 10.1111/obr.70085

**Published:** 2026-01-05

**Authors:** Fernanda Dias Massierer, Cíntia Ehlers Botton, Jessica Pietra da Silva Carvalho, Gisele Cassão, Angélica Trevisan de Nardi, Jayne Feter, Andresa Conrado Ignacio, Rodrigo Leal‐Menezes, Nórton Luís Oliveira, Lucineia Orsolin Pfeifer, Leandro dos Santos, Lucas Porto Santos, Larissa Xavier Neves da Silva, Luciana dos Passos Silva, Frederico Morais Schwingel, Carolina Weingärtner Welter, Daniel Umpierre

**Affiliations:** ^1^ Universidade Federal do Rio Grande do Sul (UFRGS) Porto Alegre Brazil; ^2^ LADD Lab, Hospital de Clínicas de Porto Alegre (HCPA) Porto Alegre Brazil; ^3^ Universidade Federal do Ceará (UFC) Fortaleza Brazil; ^4^ Universidade de São Paulo (USP) São Paulo Brazil; ^5^ Pontifícia Universidade Católica do Rio Grande do Sul (PUCRS) Porto Alegre Brazil; ^6^ Universidade Federal de Santa Maria (UFSM) Santa Maria Brazil

**Keywords:** obesity, overweight, physical activity, youth

## Abstract

Childhood and adolescent obesity poses health risks. Standardizing outcome measurements in physical activity interventions enhances evidence comparability. This study summarizes health outcomes reported in systematic reviews and classifies them under an existing taxonomy as a preliminary step toward the future development of a core outcome set. Eligible studies were systematic reviews with or without meta‐analyses involving children and adolescents (4–19 years) with overweight or obesity, reporting health outcomes from physical activity interventions. Data extraction was performed independently in pairs, collecting study characteristics, intervention details, participant demographics, and outcomes. Outcomes were classified into taxonomy‐based domains. This review identified key outcomes in body composition, lipid profile, blood pressure, and physical functioning. The most frequent body composition outcomes were BMI, body weight, and body fat. Lipid profile outcomes included HDL, total cholesterol, and LDL. Blood pressure outcomes comprised systolic and diastolic measurements. Physical functioning was assessed by time spent in physical activity. Among 137 reviews, 841 outcomes were extracted, identifying 169 unique outcomes across 16 domains. The most reported unique outcomes were BMI (52.6%), body weight, body fat, HDL, and systolic blood pressure. This study highlights the need for a Core Outcome Set to standardize outcomes in physical activity interventions for children with overweight or obesity.

**Abbreviations**
BMI: body mass index
COMET: core outcome measures in effectiveness trials
COS: core outcome set
HDL: high‐density lipoprotein
LDL: low‐density lipoprotein
MeSH: medical subject headings
PRISMA‐P: Preferred Reporting Items for Systematic Reviews and Meta‐Analysis Protocols
PROSPERO: International Prospective Register of Systematic Reviews
WHO: World Health Organization

## 1 Introduction

Obesity among children and adolescents represents a global health issue [1, 2], increasing the risk of cardiometabolic [3, 4] and physical and psychological conditions [5, 6, 7, 8, 9]. The World Health Organization (WHO) recommends to this population at least 60 min of moderate to vigorous physical activity daily, including muscle and bone strengthening activities at least three times a week [10]. Conversely, a global survey indicates that four out of five students aged 11–17 do not meet this guideline [11, 12]. Numerous physical activity interventions have been assessed in clinical trials, reinforcing the importance of behavioral strategies to mitigate obesity's impact [13, 14].

The high heterogeneity of outcomes used in physical activity programs can hinder comparative analysis and evidence synthesis among the existing interventions. A review of 137 studies was unable to provide a comprehensive synthesis due to the high variability in outcome measures, which hindered the interpretation of the interventions' impact on the physical fitness of students [15]. A solution to minimize this inconsistency is through a core outcome set (COS), which is a standardized group of outcomes for a given intervention and population, therefore increasing comparability across clinical trials. This process involves the engagement of relevant stakeholders and a comprehensive approach to selecting important outcomes [16, 17, 18]. Such need is exemplified by the highlight of critical outcomes for studies in children and adolescents aged 5–17 by the WHO [10], emphasizing the importance of a coordinated selection process for future trials.

Therefore, we aimed to summarize and classify outcomes reported in systematic reviews, seeking to inform the development of a COS for physical activity interventions for children and adolescents. Our objectives were (1) to describe individual health outcomes reported in reviews and (2) to classify the individual outcomes under an existing taxonomy of health domains [19].

## 2 Methods

This study is designed as an umbrella review encompassing systematic reviews with or without meta‐analyses. The present work is part of the development of a COS for physical activity intervention for children and adolescents with overweight or obesity. The COS project is registered in the COMET Database (https://www.comet‐initiative.org/Studies/Details/1356), whereas this umbrella review was registered in the International Prospective Register of Systematic Reviews (PROSPERO database) (CRD42019120334). The protocol, following the Preferred Reporting Items for Systematic Reviews and Meta‐Analysis Protocols (PRISMA‐P) [20]; materials; and data are available at the Open Science Framework (https://osf.io/7vaw5/files/osfstorage). The study reporting follows the PRISMA Statement for Reporting Systematic Reviews and Meta‐Analyses [21].

That were three protocol deviations: (1) expanding the age range by 1 year at the low and high limits to avoid excluding studies that encompass the population of interest, given the variability in school‐age representation across the studies; (2) restricting the population to children and adolescents with overweight or obesity to generate more specific evidence; and (3) the exclusion of cognitive outcomes due to the consensus among the reviewers of the update not considering these outcomes as health outcomes.

### 2.1 Eligibility Criteria

Inclusion criteria were (1) reports written in English, Portuguese or Spanish; (2) systematic reviews with or without meta‐analysis of intervention studies (randomized or nonrandomized, controlled, or noncontrolled); (3) samples including children and adolescents with overweight or obesity (from 4 to 19 years old), healthy, or at risk/diagnosis of cardiometabolic diseases; (4) included health lifestyle interventions with physical activity component (e.g., exercise, leisure‐time physical activity, counseling, and parent participation) with a duration of at least 4 weeks; and (5) report any health outcome measured in children or adolescents, which is defined as any measurable health variable resulting from a physical activity intervention. This includes, but is not limited to, variables related to body composition, blood biomarkers, cardiovascular measures, physical functioning, mental health, and emotional well‐being.

### 2.2 Information Sources

A search was conducted across five databases for indexed full‐text publications (PubMed, EMBASE, ERIC, SportDiscus, and Cochrane Database). Google Scholar and PROSPERO were databases used to retrieve nonpublished or nonretrieved literature (gray literature). The search period was from inception to January 2023, except for PROSPERO registration records, in which we carried out searches up to February 2024. Literature search strategies were developed using Medical Subject Headings (MeSH) and keywords. The full‐search strategies for all databases are available in Table S1.

### 2.3 Study Selection

At first, title and abstract screening was conducted by three pairs of independent reviewers (CEB, LPS, ADN, LOP, LS, and LXNS) using predefined eligibility criteria. Prior to formal screening, a pilot test involving 100 randomly selected records was conducted to standardize the interpretation of the eligibility criteria across reviewer pairs. In the second stage, full‐text screening was conducted by four independent reviewer pairs (FDM, LXNS, TSA, JF, ACI, LPS, and CWW). Each pair independently reviewed and assessed the full texts of potentially eligible studies. Discrepancies at any stage were resolved through discussion between reviewers. If consensus was not reached, a third reviewer (DU) was consulted to make the final decision. All full‐text articles assessed during the second stage are listed in Table S2.

### 2.4 Definition of Outcomes and Domains

The definition of domain, outcome, and outcome measure was based on Sinha et al. [22]. The domain is a relatively broad aspect (e.g., cardiac) of the effect of a condition (e.g., overweight/obesity) on a population (e.g., children/adolescents), within which an improvement may occur in response to an intervention (e.g., physical activity). In general, these domains may not be directly measurable themselves, so outcomes are selected to assess change within the domains. The outcome is a measurable variable (e.g., systolic blood pressure) within a domain. In contrast, the outcome measure is a scale, scoring system, questionnaire, or other tools (e.g., office blood pressure measurement) used for measuring an outcome.

The domains were predefined according to a taxonomy developed for outcomes in medical research [19]. The taxonomy comprises 38 outcomes domains, according to five core areas: mortality; physiological or clinical (e.g., endocrine, cardiac, psychiatric, and musculoskeletal); life impact (e.g., physical functioning, social functioning, and global quality of life); resource use (e.g., economic and societal/carer burden); and adverse events. The general domain encompassed variables that did not clearly fit into other predefined domains. This could include measures related to body composition, parental characteristics, or other variables not explicitly categorized elsewhere.

### 2.5 Data Extraction Process

Initially, three pairs of authors (CEB, NLO, ATDN, LOP, GC, and LXNS) independently pilot‐tested the extraction form for three included papers to ensure consistency in the interpretation of items and to generate internal definitions and improvements to the form components. Data extraction from the included studies was carried out on a standardized coded sheet, and any discrepancies were resolved by consensus or with the assistance of a third reviewer. Subsequently, during the update phase, five pairs of independent reviewers (FDM, LXNS, ACI, JPSC, RLM, TSL, FMS, YFS, CWW, and LPS) conducted a pilot with 10 papers to ensure standardization and consistency in the interpretation of items. This process aimed to enhance the quality and reliability of data extraction, contributing to a more robust and comparable analysis of the included studies. Two reviewers extracted the outcome names based on the exact wording used in the included systematic reviews. Additionally, the outcome names were cross‐checked against the original descriptions in the full texts of the systematic reviews to ensure consistency and accuracy in reflecting the terminology used by the original authors. Due to inconsistent or generic definitions of outcomes across several studies, the more specific description of the outcomes was also extracted, typically found in the methods and outcome measure sections. For instance, although authors identified the primary outcome as “adiposity,” the assessed outcome was specifically “abdominal fat” measured by “densitometry.” In such cases, both the broader outcome information, as described by the authors in the introduction and objectives (“adiposity”), and the more specific description in the methods (“abdominal fat”) were extracted. Both descriptions of outcomes are listed for each review in Table S3. Additionally, there were audit rounds to standardize equal outcomes with distinct names. Therefore, similar outcome labels (e.g., “muscle endurance” and “muscle resistance”) that were verified to reflect a unique outcome were standardized accordingly.

In this context, a total of 24 items were extracted, with key elements including (1) year and country of publication; (2) the number of studies and participants included; (3) characteristics of interventions; (4) intervention settings; (5) participant characteristics (such as age, sex, presence of overweight/obesity, or other cardiometabolic diseases); (6) health outcomes considered in the reviews; and (7) measuring instruments.

To clarify the scope of this umbrella review, we focused on the outcomes of interest identified in the included reviews, rather than encompassing all outcomes investigated by the primary studies. For example, a systematic review synthesizing the effects of physical activity on blood pressure might have included primary studies evaluating diverse outcomes such as cognitive function, body composition, or academic performance. However, if the review did not explicitly consider these additional outcomes, they were not taken into account in the present umbrella review.

### 2.6 Data Synthesis and Analysis

The included reviews and extracted data were analyzed through qualitative synthesis and descriptive statistics. Initially, we summarized each included review, identifying its investigated outcomes and assigning each outcome to a corresponding domain. The outcomes were allocated to the domain of greatest affinity, according to the concept and examples described by the Core Outcome Measures in Effectiveness Trials (COMET) initiative [18] and to the established taxonomy for outcomes in medical research [19]. We then computed the total number of outcomes observed across all reviews by summing the counts of each outcome, regardless of potential repetitions. Thereafter, we cleaned all repeated outcomes to generate a set of unique outcomes. The results are presented as the number of reviews containing different outcomes or domains, as well as the frequencies of unique outcomes or domains relative to the total number of outcomes observed.

Finally, we listed the included reviews along with their respective included trials and sample sizes. To avoid duplicate counting, we coded the RCTs included in each of the included reviews using their DOIs (Digital Object Identifier) or PMIDs (Pubmed IDs), assuring that any trial was counted only once.

## 3 Results

### 3.1 Search Results

**FIGURE 1:**
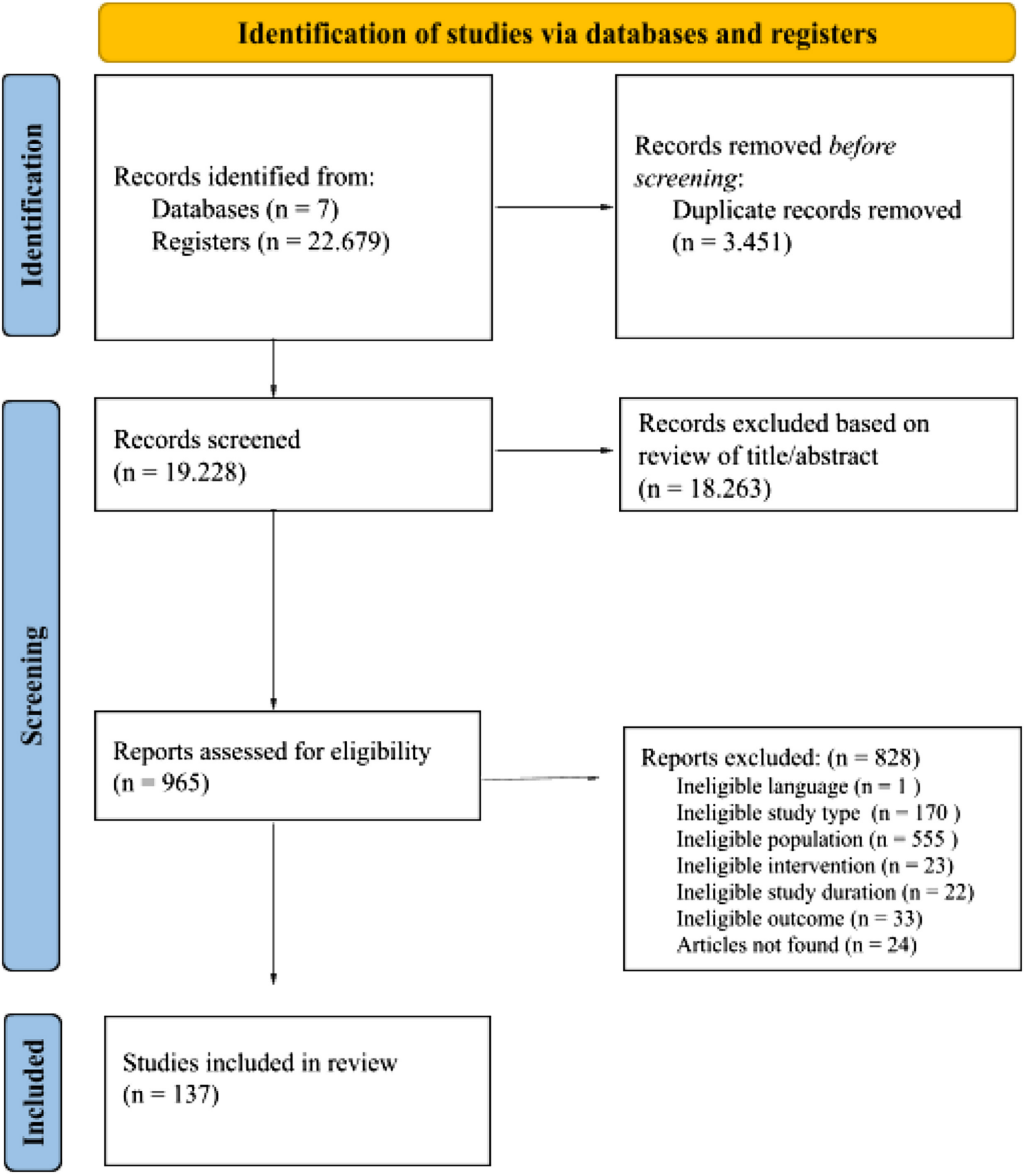
Identification and selection of articles.

After excluding 3446 duplicates, 19,233 articles were reviewed at the first stage of eligibility criteria assessment, with 18,263 being excluded after screening titles and abstracts (Figure 1). Among 970 full texts assessed (Table S2), 137 were eligible and included in the qualitative analysis. This subset comprised 48 (35.0%) systematic reviews and 89 (64.9%) systematic reviews with meta‐analysis, involving 369,285 participants in 1202 clinical trials (Table S4).

### 3.2 Study Characteristics

Participants were school‐aged children and adolescents, aged 4–19 years old. Ten (7.3%) reviews included trials assessing only children, 16 (11.6%) focused solely on adolescents, 78 (56.9%) assessed both age groups, and 33 (24.1%) assessed children or adolescents with family involvement. All reviews summarized trials that included children or adolescents with overweight or obesity, whereas 48 (34.7%) reviews also included trials that recruited participants with healthy weight. Moreover, all reviews summarized studies with participants of both sexes, with the exception of one review that considered only females.

Regarding intervention settings, 73 (53.3%) reviews did not report this information. Among the reviews that reported intervention settings, 20 (14.6%) were conducted exclusively in schools, 3 (2.2%) in health clinics or hospitals, 4 (2.9%) in home‐based settings, and 1 (0.7%) in community settings. Additionally, 36 (26.3%) reviews were delivered across two or more settings. Concerning intervention components, 71 (51.8%) reviews included only physical activity, whereas 66 (48.2%) combined physical activity with other components such as dietary, educational, and behavioral strategies. Across the included reviews, interventions lasted at least 4 weeks. The characteristics of the reviews are available in Table S5.

### 3.3 Health Outcomes and Domains

From the 137 included reviews, we cumulatively extracted 841 outcomes. These outcomes often overlapped across the reviews and were subsequently grouped into 169 unique outcomes, which represent distinct conceptual categories. This classification was organized into 16 domains according to an established taxonomy (Table S6), presented in descending order based on the number of outcomes included in each domain.

The three most frequent domains were, respectively, “endocrine” (39 outcomes), “physical functioning” (28 outcomes), and “emotional functioning/wellbeing” (18 outcomes). On the other hand, the less frequent domains were, respectively, “perceived health status,” “delivery of care” (each domain having three to four outcomes), and overall “psychiatric” and “global quality of life” (2 outcomes).

A total of 19 out of 169 outcomes were summarized in at least 10 reviews (Table 1). From the total of 137 reviews, body mass index (BMI) was the outcome most largely assessed (*n* = 72 reviews; 52.6%), followed by body weight (*n* = 39; 28.5%), body fat (*n* = 33; 24.1%), high‐density lipoprotein (HDL cholesterol) (*n* = 31; 22.6%), and systolic blood pressure (*n* = 31; 22.6%).

**TABLE 1:** Unique outcomes, domains, and subdomains (if any) for outcomes appearing in at least 10 systematic reviews.

Outcomes	Frequency, *N*	Frequency, %	Domain (subdomain)
Body mass index (BMI)	72	52.6	General (body composition)
Body weight	39	28.5	General (body composition)
Body fat	33	24.1	General (body composition)
High‐density lipoproteins (HDL)	31	22.6	Blood and lymphatic system (lipid profile)
Systolic blood pressure (SBP)	31	22.6	Cardiac (blood pressure)
Body fat‐free mass	29	21.2	General (body composition)
Total cholesterol	28	20.4	Blood and lymphatic system (lipid profile)
Diastolic blood pressure (DBP)	27	19.7	Cardiac (blood pressure)
Low‐density lipoproteins (LDL)	26	19.0	Blood and lymphatic system (lipid profile)
Triglycerides	25	18.2	Blood and lymphatic system (lipid profile)
Skinfold thickness	17	12.4	General (body composition)
Time spent in physical activity	16	11.7	Physical functioning
Regional fat	16	11.7	General (body composition)
Waist circumference	15	10.9	General (body composition)
Muscle strength	13	9.5	Physical functioning
Physical activity level	13	9.5	Physical functioning
Fasting blood glucose	12	8.8	Blood and lymphatic system (lipid profile)
Sedentary behavior	12	8.8	Physical Functioning

*Note:* Frequencies are expressed either in absolute (*N*) or relative terms (%). Absolute frequencies indicate the number of systematic reviews reporting each of the listed outcomes, whereas relative frequencies represent the proportion of reviews within the pool of 137 summarized reviews.

**FIGURE 2:**
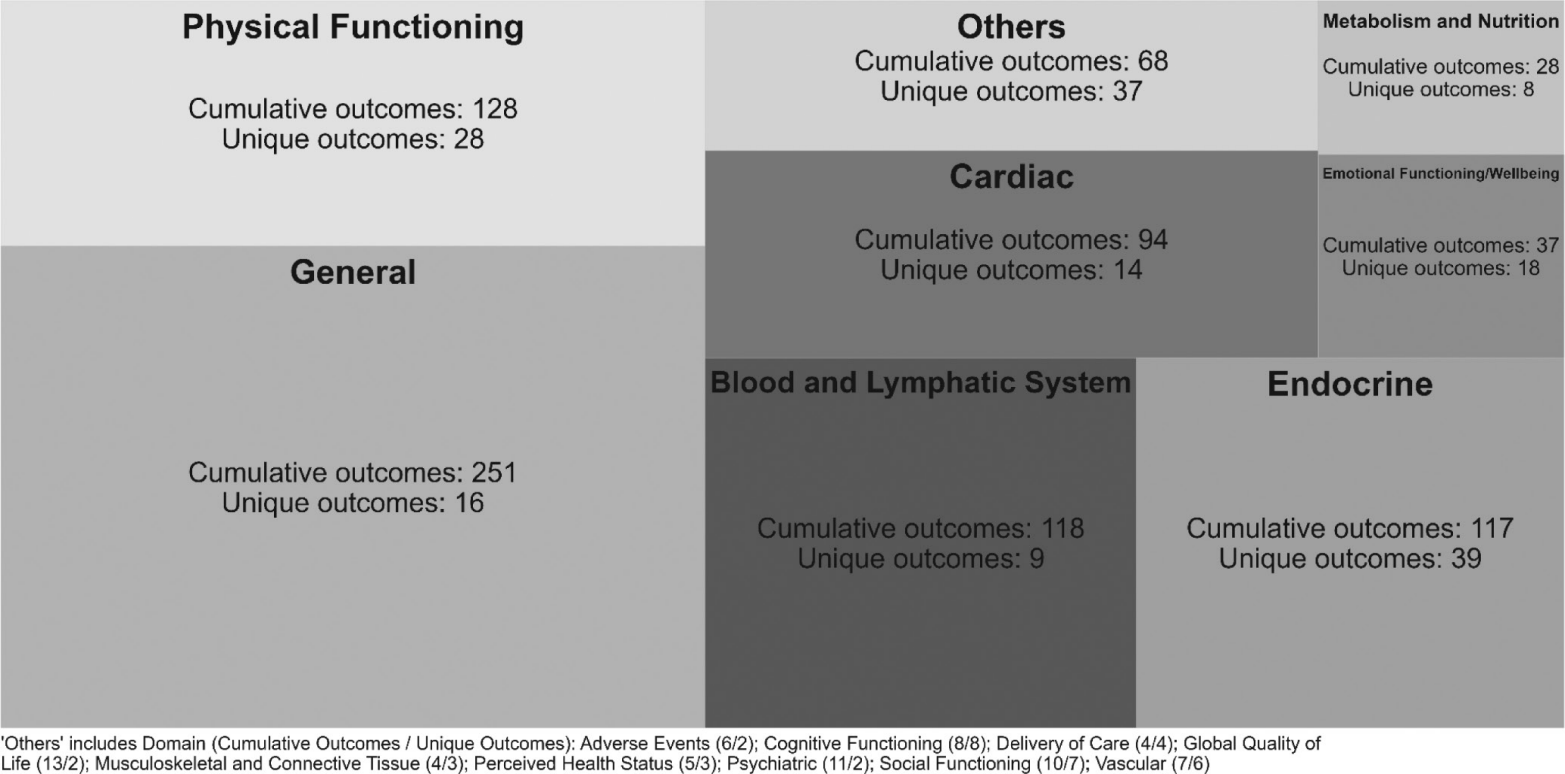
Graphical representation of outcomes by domains.

Although the “endocrine” domain showed the highest number of outcomes (39 outcomes), the “general” domain, which was mostly composed of body composition measures, also included other outcomes that together totalized a larger cumulative frequency (*n* = 251) (Figure 2).

## 4 Discussion

In this study, we systematically summarized outcomes assessed in systematic reviews of physical activity interventions in children and/or adolescents with overweight or obesity. Based on the 137 eligible reviews, we identified a total of 841 outcomes, comprising 169 unique ones, distributed into 16 domains. When analyzing the most relevant domain (General), outcomes related to body composition were cumulatively observed 251 times (30%) across the reviews. Meanwhile, the second most frequent domain (physical functioning) showed 128 outcomes (15%) related to physical function and fitness. Following this, the domains “blood and lymphatic system,” “endocrine,” and “cardiac” were identified, representing 14%, 13.8%, and 11% of the outcomes, respectively.

BMI is a simple, low‐cost, and widely used anthropometric measure to detect overweight and obesity among several population subgroups. In our study, one out of two systematic reviews (*n* = 72; 52.6%) used BMI as an outcome in their syntheses. However, it is important to highlight its limitations to measure body fatness [23]. In children aged 6–14 years, waist‐to‐height ratio could identify increased LDL cholesterol levels in children who would not be classified as overweight by the WHO BMI standard [24, 25]. Although both measures are useful in assessing overweight or obesity, we observed that only a few reviews assessed waist‐to‐height ratio as an outcome, despite its diagnostic usefulness to characterize cardiometabolic risk factors in children [26].

We highlight that WHO guidelines on physical activity and sedentary behavior recently classified adiposity outcomes as critical for children and adolescents aged 5–17 [10]. However, it is worth questioning whether body composition should be prioritized as the primary domain when selecting outcomes for physical activity programs for children with overweight or obesity. This consideration is particularly relevant given that the benefits of physical activity may extend beyond body composition, including improvements in cardiometabolic health, motor skills, and other physical and psychological domains. A broader perspective on outcome selection could provide a more comprehensive assessment of programs tailored for children or adolescents.

Although all interventions in this review included a physical activity component, only a few studies reported outcomes related to either time spent in physical activity or sedentary behavior. Importantly, children and adolescents with obesity are 20%–30% less physically active and present lower physical fitness [27, 28, 29] compared to their peers without obesity [30, 31, 32, 33]. Given the WHO's emphasis on the role of physical activity in improving several health outcomes and reducing obesity risk [34, 35], measures related to physical activity itself or sedentary behavior should be considered in future studies. Likewise, we emphasize that psychological and social outcomes were scarcely explored in the included systematic reviews, especially in the “psychiatric” and “social functioning” domains. Therefore, outcomes related to depressive symptoms, anxiety, social acceptance, and body dissatisfaction could be further considered in clinical trials, because there is evidence showing their importance in children and adolescents with overweight or obesity [8, 9].

Some studies have assessed whether obesity is associated with the cognitive function of children and adolescents [7, 36]. Lastly, current evidence has shown the importance of social support or parents' engagement in physical activities for their children's behavior, as well as parental involvement in weight control and engagement in lifestyle strategies [37, 38, 39]. Although some studies have designed interventions with parental participation, only a few assess outcomes related to parents' or family aspects. Because children and adolescents may rely on family support to engage and maintain health intervention, exploring parental and family outcomes, both related to physical activity and not, seems important in future research.

Building upon this need for standardized evaluations, domains are proposed by the COS developers, based on a summary of outcomes presented in the literature and consensus from stakeholders [16]. Similar studies with the pediatric population have abandoned the use of taxonomy and classified their outcomes using their own system [13, 40]. To our knowledge, our study is a primary comprehensive synthesis of outcomes investigated in physical activity interventions for children and adolescents with overweight or obesity. The present review intends to inform a future COS, which requires other stages such as collecting outcomes from stakeholders, undertaking Delphi rounds, and convening a diverse panel to reach consensus in a COS [41, 42, 43]. As outcomes may vary and be sensitive to specific age ranges, it is reasonable that a COS development will need to consider children and adolescents separately. More importantly, the adoption of a COS would allow standardizing outcomes in future studies, providing a more consistent and comparable assessment of physical activity interventions for this population.

## 5 Limitations

Some limitations should be considered in our study. First, classifying outcomes into domains was challenging because the implemented taxonomy [19] was created for trials. Because we analyzed systematic reviews, which do not provide such detailed descriptions of outcomes, the classification was more imprecise than we anticipated. Similarly, it was created based on clinical conditions, disregarding outcomes that would be important for pediatric work, such as parental information. As a result, our description of outcomes is limited to broader terms. Second, the wide age range considered in our meta‐epidemiological assessment may require further data filtering if a future COS development occurs apart for children and adolescents. Finally, our search covers studies up to February 2024, so more recent publications were not included. The large volume of citations to assess makes data preparation time‐consuming and challenging to complete close to the search end. However, we consider it unlikely that an updated search would substantially alter the results.

## 6 Conclusion

This umbrella review presents a high number of domains and outcomes used in studies with physical activity interventions for children and adolescents with overweight or obesity. Although body composition outcomes were quite frequent, other outcomes related to physical activity, mental health, and social aspects were largely underexplored. This review informs the development of a future COS that may increase the standardization and comparability across trials of physical activity interventions for these populations.

## Author Contributions

Daniel Umpierre, Cíntia Ehlers Botton, and Fernanda Dias Massier had full access to all of the data in the study and took responsibility for the integrity of the data and the accuracy of the data analysis. *Concept and design*: Daniel Umpierre, Cíntia Ehlers Botton, and Fernanda Dias Massier. *Study selection and data extraction*: Fernanda Dias Massier, Cíntia Ehlers Botton, Jessica Pietra da Silva Carvalho, Gisele Cassão, Angélica Trevisan de Nardi, Jayne Feter, Andresa Conrado Ignacio, Rodrigo Leal‐Menezes, Nórton Luís Oliveira, Lucineia Orsolin Pfeifer, Lucas Porto Santos, Leandro dos Santos, Larissa Xavier Neves da Silva, Luciana dos Passos e Silva, Frederico Morais Schwingel, and Carolina Weingärtner Welter. *Acquisition, analysis, or interpretation of data*: Daniel Umpierre, Fernanda Dias Massier, Cíntia Ehlers Botton, Larissa Xavier Neves da Silva, and Nórton Luís Oliveira. *Drafting the manuscript*: Daniel Umpierre, Fernanda Dias Massier, and Cíntia Ehlers Botton.

## Funding

The project was hosted at the Hospital de Clínicas de Porto Alegre (Porto Alegre, Brazil). All researchers received funding from the Coordenação de Aperfeiçoamento de Pessoal de Nível Superior (CAPES, Brazil, 001‑42001013017P9) and the Conselho Nacional de Desenvolvimento Científico e Tecnológico (313206/2022‐8).

## Conflicts of Interest

The authors declare no conflicts of interest.

## Supporting information


**Table S1:** Complete database search strategy.
**Table S2:** Articles assessed at the full text level.
**Table S3:** Total outcomes.
**Table S4:** List of clinical trials included in all 137 reviews.
**Table S5:** Characteristics of 137 reviews included.
**Table S6:** Frequency of all unique outcomes and domains.

## Data Availability

The data for this study are available on the Open Science Framework Platform (https://osf.io/7vaw5/).
