# Supplementary material for: Health Outcomes in Children and Adolescents With Overweight or Obesity Exposed to Physical Activity Interventions: An Umbrella Review Covering Over 1200 Trials
Source: Obes Rev. 2026 Jan 5;27(6):e70085. doi: 10.1111/obr.70085 (PMC13136782; doi:10.1111/obr.70085)
Supplement: Supplementary file 1 — Table S1: Complete database search strategy. Table S2: Articles assessed at the full text level. Table S3: Total outcomes. Table S4: List of clinical trials included in all 137 reviews. Table S5: Characteristics of 137 reviews included. Table S6: Frequency of all unique outcomes and domains. [file OBR-27-e70085-s001.pdf]

**Table S1. Complete database search strategy.**

| Database | Search Strategy |
|---|---|
| Pubmed | #1 Child[Mesh] OR Child*[tiab] OR Youth[tiab] OR Adolescent[Mesh] OR Adolesc*[tiab] OR Pube*[tiab] OR Boys[tiab] OR Girls[tiab] OR School*[tiab] OR Pediatric*[tiab] OR Juvenile[tiab] OR Kid[tiab]<br><br>#2 Exercise Therapy[mesh] OR Exercise[mesh] OR Exercis*[tiab] OR Physical Activ*[tiab] OR Healthy Lifestyle[mesh] OR Health Behavior[mesh] OR Sports[mesh]<br><br>#3 MEDLINE[Title/Abstract] OR (systematic[Title/Abstract] AND review[Title/Abstract]) OR meta-analysis[Publication Type] OR (system*[Title/Abstract] AND review[Title/Abstract]) OR meta-an*[tiab]<br><br>#1 AND #2 AND #3 |
| Embase | #1 'child'/exp OR child*:ti,ab OR 'juvenile'/exp OR 'adolescent'/exp OR adolescen*:ti,ab OR puberty:ti,ab OR boy:ti,ab OR girl:ti,ab OR 'school'/exp OR pediatrics:ti,ab OR kid:ti,ab<br><br>#2 'exercise'/exp OR exercis*:ti,ab OR 'physical activity'/exp OR physical activit*:ti,ab OR 'healthy lifestyle'/exp OR healthy lifestyle:ti,ab OR 'health behavior'/exp OR health behavior:ti,ab OR 'sport'/exp OR sport:ti,ab<br><br>#3 'systematic review'/exp OR systematic review:ti,ab OR 'meta analysis'/exp OR meta anal*:ti,ab<br><br>#1 AND #2 AND #3 |
| Cochrane | #1 [Child] OR [Adolescent] OR [Puberty] OR [Schools] OR boys OR girls<br><br>#2 [Exercise] OR [Healthy Lifestyle] OR [Health Behavior] OR [Sports] OR Physical Activity<br><br>#3 [Meta-Analysis as Topic] OR Systematic Review<br><br>#1 AND #2 AND #3 |
| SPORTDiscus | #1 Children OR Youth OR Adolescent OR Puberty OR Boys OR Girls OR School OR Pediatrics OR Juvenile OR Kid<br><br>#2 Exercise Therapy OR Exercise OR Physical Activity OR Healthy Lifestyle OR Health Behavior OR Sports<br><br>#3 systematic review OR meta-analysis OR review<br><br>#1 AND #2 AND #3 |

| | |
|---|---|
| ERIC | #1 "Child" OR "Youth" OR "Adolescent" OR "Puberty" OR "Boys" OR "Girls" OR "School" OR "Pediatrics" OR "Juvenile" OR "Kid"<br><br>#2 "Exercise Therapy" OR "Exercise" OR "Physical Activity" OR "Healthy Lifestyle" OR "Health Behavior" OR "Sports"<br><br>#3 "systematic review" OR "meta-analysis"<br><br>#1 AND #2 AND #3 |
| PROSPERO | #1 Child [Mesh]/exp OR Adolescent [Mesh]/exp OR Pediatrics [Mesh]/exp OR Puberty:ti OR Youth:ti OR Boys:ti OR Girls:ti OR School:ti OR Juvenile:ti<br><br>#2 Exercise [Mesh]/exp OR Healthy Lifestyle [Mesh]/exp OR Health Behavior [Mesh]/exp OR Sport:ti OR Physical Activity:ti<br><br>#1 AND #2 |

## Table S2. Articles assessed at the full text level.

| Title | Authors (Year) | Decision/Reason for exclusion |
|---|---|---|
| Interventions to Treat Obesity in Mexican Children and Adolescents: Systematic Review and Meta-Analysis. | Aceves-Martins et al., 2022 | INCLUDED |
| Diet, physical activity and behavioural interventions for the treatment of overweight or obese adolescents aged 12 to 17 years | Al-Khudairy et al., 2017 | INCLUDED |
| Characteristics of Multicomponent Interventions to Treat Childhood Overweight and Obesity in Extremely Cold Climates: A Systematic Review of a Randomized Controlled Trial | Albornoz-Guerrero et al., 2021 | INCLUDED |
| The Effect of Physical Activity Interventions on Children's Cognition and Metacognition: A Systematic Review and Meta-Analysis | Álvarez-Bueno et al., 2017 | INCLUDED |
| Systematic Review: Frameworks Used in School-Based Interventions, the Impact on Hispanic Children's Obesity-Related Outcomes | Andrade et al., 2018 | INCLUDED |
| The Psychological Effects of Exergames for Children and Adolescents with Obesity: A Systematic Review and Meta-Analysis | Andrade et al., 2019 | INCLUDED |
| The effectiveness of e-health interventions for the treatment of overweight or obesity in children and adolescents: systematic review and meta-analysis | Azevedo et al., 2023 | INCLUDED |
| School-Based High-Intensity Interval Training Programs for Promoting Physical Activity | Bento et al., 2022 | INCLUDED |

| **Title** | **Authors (Year)** | **Decision/Reason for exclusion** |
|---|---|---|
| and Fitness in Adolescents: A Systematic Review. | | |
| What change in body mass index is associated with improvement in percentage body fat in childhood obesity? A meta-regression | Birch et al., 2019 | INCLUDED |
| Weight loss interventions for overweight and obese adolescents: a systematic review | Boff et al,. 2017 | INCLUDED |
| Impact of Lifestyle Intervention Programs for Children and Adolescents with Overweight or Obesity on Body Weight and Selected Cardiometabolic Factors—A Systematic Review | Bondyra-Wisniewska et al., 2021 | INCLUDED |
| Characteristics of physical activity interventions and effects on cardiorespiratory | Braaksma et al., 2018 | INCLUDED |

| **Title** | **Authors (Year)** | **Decision/Reason for exclusion** |
|---|---|---|
| fitness in children aged 6-12 years-A systematic review | | |
| Diet and physical activity interventions to prevent or treat obesity in South Asian children and adults: a systematic review and meta-analysis | Brown et al., 2015 | INCLUDED |
| Interventions for preventing obesity in children (Review) | Brown et al., 2019 | INCLUDED |
| Resistance Training and Insulin Sensitivity in Youth: A Meta-analysis | Burns et al., 2019 | INCLUDED |
| Effects of Exercise in Improving Cardiometabolic Risk Factors in Overweight Children: A Systematic Review and Meta-Analysis | Busnatu et al., 2022 | INCLUDED |
| Effect of High-Intensity Interval Training versus Moderate-Intensity Continuous | Cao et al., 2019 | INCLUDED |

| Title | Authors (Year) | Decision/Reason for exclusion |
|---|---|---|
| Training on Cardiorespiratory Fitness in Children and Adolescents: A Meta-Analysis | | |
| Effects of High-Intensity Interval Training and Moderate-Intensity Continuous Training on Cardiometabolic Risk Factors in Overweight and Obesity Children and Adolescents: A Meta-Analysis of Randomized Controlled Trials | Cao et al., 2021 | INCLUDED |
| Efectos del entrenamiento de la aptitud muscular sobre la adiposidad corporal y el desempeÃ±o motriz en niÃ±os y jÃ³venes: un meta-anÃ¡lisis. / Effects of Resistance Training on body fat and motor performance skills in children and adolescents: A Meta-Analysis | Casas et al., 2018 | INCLUDED |

| Title | Authors (Year) | Decision/Reason for exclusion |
|---|---|---|
| Physical activity and cardiovascular risk factors in children: meta-analysis of randomized clinical trials | Cesa et al., 2014 | INCLUDED |
| Effects of aerobic exercise and resistance exercise on physical indexes and cardiovascular risk factors in obese and overweight school-age children: A systematic review and meta-analysis | Chen et al., 2021 | INCLUDED |
| Effect of exercise training on arterial stiffness in obese and overweight children: a meta-analysis. | Cheng et al., 2022 | INCLUDED |
| Effectiveness of obesity interventions among South Korean children and adolescents and importance of the type of intervention component: a meta-analysis | Choe et al., 2022 | INCLUDED |

| Title | Authors (Year) | Decision/Reason for exclusion |
|---|---|---|
| Recreational Soccer Training Effects on Pediatric Populations Physical Fitness and Health: A Systematic Review | Clemente et al., 2022 | INCLUDED |
| The effect of resistance training interventions on weight status in youth: a meta-analysis | Collins et al., 2018 | INCLUDED |
| The Effect of Resistance Training Interventions on 'The Self' in Youth: a Systematic Review and Meta-analysis | Collins et al., 2019 | INCLUDED |
| Diet, physical activity, and behavioural interventions for the treatment of overweight or obesity in preschool children up to the age of 6 years | Colquitt et al., 2016 | INCLUDED |
| The effects of active video games on health-related physical fitness and motor competence in overweight or | Comeras-Chueca et al., 2021 | INCLUDED |
| obese youth a systematic review and meta analysis | | |
| Effects of supervised exercise training on lipid profile of children and adolescents: Systematic review, meta-analysis and meta-regression. | Costa et al., 2020 | INCLUDED |
| The Morphofunctional Adaptations of Resistance Exercise on Reducing Cardiovascular Risk Factors in Adolescents: A Systematic Review (2012 to 2018). | da Rosa-Santos et al., 2019 | INCLUDED |
| RESPONSES OF PLASMA ADIPOKINES TO HIGH INTENSITY INTERVAL TRAINING: SYSTEMATIC REVIEW. / RESPUESTAS DE LAS ADIPOCINAS PLASMÁTICAS AL ENTRENAMIENTO POR | Da Silva et al., 2020 | INCLUDED |

| Title | Authors (Year) | Decision/Reason for exclusion |
|---|---|---|
| INTERVALOS DE ALTA INTENSIDAD: REVISIÓN SISTEMÁTICA | | |
| Feasibility of incorporating high-intensity interval training into physical education programs to improve body composition and cardiorespiratory capacity of overweight and obese children: A systematic review. | Delgado-Floody et al., 2019 | INCLUDED |
| The Effects of Park-Based Interventions on Health-Related Outcomes Among Youth: | Deshira et al., 2022 | INCLUDED |
| Exercise and Vascular Function in Child Obesity: A Meta-Analysis | Dias et al., 2015 | INCLUDED |
| Serious games as an educational strategy to control childhood obesity: a systematic literature review1 | Dias et al., 2018 | INCLUDED |

| Title | Authors (Year) | Decision/Reason for exclusion |
|---|---|---|
| Influence of Chronic Exposure to Exercise on Heart Rate Variability in Children and Adolescents Affected by Obesity: A Systematic Review and Meta-Analysis | Dias et al., 2021 | INCLUDED |
| School-based physical activity programs for promoting physical activity and fitness in children and adolescents aged 6 to 18 | Dobbins et al., 2013 | INCLUDED |
| Effects of dance on the physical activity level of children and adolescents - a systematic review | Dos Santos et al., 2021 | INCLUDED |
| Football as a Health Promotion Strategy | Eberl et al., 2019 | INCLUDED |
| Physiological Responses to Combat Sports in Metabolic Diseases: A Systematic Review | Eckstein et al., 2022 | INCLUDED |

| Title | Authors (Year) | Decision/Reason for exclusion |
|---|---|---|
| Effects of Physical Training on Heart Rate Variability in Children and Adolescents with Chronic Diseases: A Systematic Review and Meta-analysis | Estéves-González et al., 2022 | INCLUDED |
| Effect of physical training on the blood pressure of adolescents with obesity | Farah et al., 2012 | INCLUDED |
| Systematic review and meta-analysis of school-based obesity interventions in mainland China | Feng et al., 2017 | INCLUDED |
| Child and youth participatory interventions for addressing lifestyle-related childhood obesity: a systematic review | Frerichs et al., 2016 | INCLUDED |
| Endocrinology and Adolescence: aerobic exercise reduces insulin resistance markers in obese youth: a meta-analysis of randomized controlled trials | García-Hermoso et al., 2014 | INCLUDED |
| Effects of Aerobic Plus Resistance Exercise on Body Composition Related Variables in Pediatric Obesity: A Systematic Review and Meta-Analysis of Randomized Controlled Trials | García-Hermoso et al., 2015 | INCLUDED |
| Is high-intensity interval training more effective on improving cardiometabolic risk and aerobic capacity than other forms of exercise in overweight and obese youth? A meta-analysis | García-Hermoso et al., 2016-a | INCLUDED |
| Concurrent aerobic plus resistance exercise versus aerobic exercise alone to improve health outcomes in paediatric obesity: a systematic review and meta-analysis | García-Hermoso et al., 2016-b | INCLUDED |

| Title | Authors (Year) | Decision/Reason for exclusion |
|---|---|---|
| Exercise-based interventions and C-reactive protein in overweight and obese youths: a meta-analysis of randomized controlled trials | García-Hermoso et al., 2016-c | INCLUDED |
| Exercise, adipokines and pediatric obesity: a meta-analysis of randomized controlled trials | García-Hermoso et al., 2017-a | INCLUDED |
| Effects of Exercise on Carotid Arterial Wall Thickness in Obese Pediatric Populations: A Meta-Analysis of Randomized Controlled Trials | García-Hermoso et al., 2017-b | INCLUDED |
| Association of Physical Education With Improvement of Health-Related Physical Fitness Outcomes and Fundamental Motor Skills Among Youths A Systematic Review and Meta-analysis | Garcia-Hermoso et al., 2020-a | INCLUDED |

| Title | Authors (Year) | Decision/Reason for exclusion |
|---|---|---|
| Association between Exercise-Induced Changes in Cardiorespiratory Fitness and Adiposity among Overweight and Obese Youth: A Meta-Analysis and Meta-Regression Analysis | Garcia-Hermoso et al., 2020-b | INCLUDED |
| Effects of Exercise Intervention on Health‑Related Physical Fitness and Blood Pressure in Preschool Children: A Systematic Review and Meta‑Analysis of Randomized Controlled Trials | García-Hermoso et al., 2020-c | INCLUDED |
| A systematic review of the effectiveness of school-based obesity prevention programmes for First Nations, Inuit and Metis youth in Canada | Godin et al., 2015 | INCLUDED |
| The Effects of Exercise on Abdominal Fat and Liver Enzymes in Pediatric Obesity: | González-Ruiz et al., 2017 | INCLUDED |

| Title | Authors (Year) | Decision/Reason for exclusion |
|---|---|---|
| A Systematic Review and Meta-Analysis | | |
| School-Based Interventions on Childhood Obesity. A Meta-Analysis | Gonzalez-Suarez et al., 2009 | INCLUDED |
| The effect of school-based physical activity interventions on body mass index: a meta-analysis of randomized trials | Guerra et al., 2013 | INCLUDED |
| School-based physical activity and nutritional education interventions on body mass index: a meta-analysis of randomised community trials - project PANE | Guerra et al., 2014 | INCLUDED |
| Physical activity interventions for the mental health of children: A systematic review | Hale et al., 2023 | INCLUDED |

| Title | Authors (Year) | Decision/Reason for exclusion |
|---|---|---|
| Computer- and web-based interventions to increase preadolescent and adolescent physical activity: a systematic review | Hamel et al., 2011 | INCLUDED |
| Does Physical Activity-Based Intervention Improve Systemic Proinflammatory Cytokine Levels in Overweight or Obese Children and Adolescents? Insights from a Meta-Analysis of Randomized Control Trials | Han et al., 2019 | INCLUDED |
| Effect of school-based physical activity interventions on body mass index in children: a meta-analysis | Harris et al., 2009 | INCLUDED |
| Effects of Physical Exercise on Cardiometabolic Biomarkers and Inflammatory | Hejazi et al., 2022 | INCLUDED |
| The Effects of Football Practice on Nutritional Status and Body | Hernandez-Martin et al., 2021 | INCLUDED |

| Title | Authors (Year) | Decision/Reason for exclusion |
|---|---|---|
| Composition in Children: A Systematic Review and Meta-Analysis | | |
| Impact of dietary and exercise interventions on weight change and metabolic outcomes in obese children and adolescents: a systematic review and meta-analysis of randomized trials | Ho et al., 2013 | INCLUDED |
| Non-curricular approaches for increasing physical activity in youth: a review | Jago et al., 2004 | INCLUDED |
| Parent-only vs. parent-child (family-focused) approaches for weight loss in obese and overweight children: A systematic review and meta-analysis | Jull and Chen., 2013 | INCLUDED |
| Effects of Exercise Intervention on Visceral Fat in Obese | Jung et al., 2018 | INCLUDED |

| Title | Authors (Year) | Decision/Reason for exclusion |
|---|---|---|
| Children and Adolescents: Meta-analysis | | |
| Evaluation of Physical Activity and Lifestyle Interventions Focused on School Children with Obesity Using Accelerometry: A Systematic Review and Meta-Analysis | Jurado-Castro et al., 2020 | INCLUDED |
| Effects of aerobic exercise on non-high-density lipoprotein cholesterol in children and adolescents: a meta-analysis of randomized controlled trials | Kelley and Kelley., 2008 | INCLUDED |
| Exercise and adiposity in overweight and obese children and adolescents: a systematic review with network meta-analysis of randomised trials | Kelley et al., 2019 | INCLUDED |
| Exercise and Cardiovascular Disease Risk Factors in | Kelley et al., 2021 | INCLUDED |

| Title | Authors (Year) | Decision/Reason for exclusion |
|---|---|---|
| Children and Adolescents With Obesity: A Systematic Review With Meta-Analysis of Randomized Controlled Trials | | |
| Effectiveness of family-based eHealth interventions in cardiovascular disease risk reduction: A systematic review | Kemp et al., 2021 | INCLUDED |
| Sport-based physical activity intervention on body weight in children and adolescents: a meta-analysis | Kim et al., 2017 | INCLUDED |
| School-based obesity prevention programs: an evidence-based review | Kropski et al., 2008 | INCLUDED |
| The effects of aerobic physical activity on adiposity in school-aged children and youth: a systematic review of randomized controlled trials | Laframboise et al., 2011 | INCLUDED |

| Title | Authors (Year) | Decision/Reason for exclusion |
|---|---|---|
| Influences of exercise interventions on overweight and obesity in children and adolescents | Lee et al., 2020 | INCLUDED |
| The Effects of Different Exercise Modalities in the Treatment of Cardiometabolic Risk Factors in Obese Adolescents with Sedentary Behavior—A Systematic Review and Meta-Analysis of Randomized Controlled Trials | Li and Chen et al., 2021 | INCLUDED |
| Effectiveness of lifestyle interventions for treatment of overweight/obesity among children in China: A systematic review and meta-analysis | Li et al., 2022 | INCLUDED |
| Interventions to prevent or treat childhood obesity in Māori & Pacific Islanders: a systematic review | Littlewood et al., 2020 | INCLUDED |

| Title | Authors (Year) | Decision/Reason for exclusion |
|---|---|---|
| Comparative Effectiveness of High-Intensity Interval Training and Moderate-Intensity Continuous Training for Cardiometabolic Risk Factors and Cardiorespiratory Fitness in Childhood Obesity: A Meta-Analysis of Randomized Controlled Trials | Liu et al., 2020 | INCLUDED |
| Does process evaluation enhance school-based High-Intensity Interval Training (HIIT) Interventions? A systematic review and meta-analysis for Randomized Controlled Trials | Liu et al., 2024 | INCLUDED |
| Effect of Active Video Games on Healthy Children's Fundamental Motor Skills and Physical Fitness: A Systematic Review | Liu, W et al., 2020 | INCLUDED |

| Title | Authors (Year) | Decision/Reason for exclusion |
|---|---|---|
| Effect of Exercise on Concentration of High-Density Lipoprotein in Youth: A Systematic Review and Meta-Analysis. | Lopes et al., 2019 | INCLUDED |
| Effectiveness of interventions to promote healthy weight in general populations of children and adults: a meta-analysis | Luckner et al., 2012 | INCLUDED |
| High Intensity Interval Training (HIIT) Improves Cardiorespiratory Fitness (CRF) in Healthy, Overweight and Obese Adolescents: A Systematic Review and Meta-Analysis of Controlled Studies | Martin-Smith et al., 2020 | INCLUDED |
| Family involvement in weight control, weight maintenance and weight-loss interventions: A systematic review of randomised trials | Mclean et al., 2003 | INCLUDED |

| Title | Authors (Year) | Decision/Reason for exclusion |
|---|---|---|
| Diet, physical activity and behavioural interventions for the treatment of overweight or obese children from the age of 6 to 11 years | Mead et al., 2017 | INCLUDED |
| Effectiveness of after-school interventions at increasing moderate-to-vigorous physical activity levels in 5- to 18-year olds: a systematic review and meta-analysis | Mears and Jago., 2016 | INCLUDED |
| [Intervention programs to promote physical activity in school children: systematic review] | Medina-Blanco et al., 2011 | INCLUDED |
| The impact of long-term school-based physical activity interventions on body mass index of primary school children - a meta-analysis of randomized controlled trials | Mei et al., 2016 | INCLUDED |

| Title | Authors (Year) | Decision/Reason for exclusion |
|---|---|---|
| Effects of Strength Training on Body Fat in Children and Adolescents with Overweight and Obesity: A Systematic Review with Meta-Analysis | Méndez-Hernández et al., 2022 | INCLUDED |
| High-Intensity Interval Training Improves physical morphology, Cardiopulmonary Fitness and Metabolic Risk Indicators of Cardiovascular Disease in Children and Adolescents: A Systematic Review and Meta-Analysis | Menjie et al., 2022 | INCLUDED |
| School-Based Exercise Programs for Promoting Cardiorespiratory Fitness in Overweight and Obese Children Aged 6 to 10 | Mijalkovic et al., 2022 | INCLUDED |
| Is Physical Fitness Associated with Health in Overweight and Obese Youth? A Systematic Review | Millard-Staffor et al., 2013 | INCLUDED |

| Title | Authors (Year) | Decision/Reason for exclusion |
|---|---|---|
| Interventions for the treatment of obesity among children and adolescents in Latin America: a systematic review | Nagle et al., 2013 | INCLUDED |
| Effects of group sports on health-related physical fitness of overweight youth: A systematic review and meta-analysis | Oliveira et al., 2017 | INCLUDED |
| The effects of school-based lifestyle interventions on body mass index and blood pressure: a multivariate multilevel meta-analysis of randomized controlled trials | Oosterhoff et al., 2016 | INCLUDED |
| Metabolic effects of exercise on childhood obesity: a current view | Paes et al., 2015 | INCLUDED |
| Influence of Intensity of Physical Activity on Adiposity and Cardiorespiratory Fitness in 5-18 Year Olds | Parikh et al., 2011 | INCLUDED |
| Physical Activity Interventions to Alleviate Depressive Symptoms in Children and | Recchia et al., 2023 | INCLUDED |
| The Benefits of Resistance Training in Obese Adolescents: A Systematic Review and Meta-analysis | Ribeiro et al., 2022 | INCLUDED |
| Systematic review of physical activity and exercise interventions on body mass indices, subsequent physical activity and psychological symptoms in overweight and obese adolescents | Ruotsalainen et al., 2015 | INCLUDED |
| Improvement of aerobic fitness in obese children: a meta-analysis | Saavedra et al., 2011 | INCLUDED |

| Title | Authors (Year) | Decision/Reason for exclusion |
|---|---|---|
| Educational interventions in childhood obesity: a systematic review with meta-analysis of randomized clinical trials | Sbruzzi et al., 2013 | INCLUDED |
| What is the effect of resistance training on the strength, body composition and psychosocial status of overweight and obese children and adolescents? A Systematic review and meta-analysis | Schranz et al., 2013 | INCLUDED |
| A systematic review and meta-analysis of energy and macronutrient intake responses to physical activity interventions in children and adolescents with obesity | Schwartz et al., 2017 | INCLUDED |
| Effectiveness and process evaluation in obesity and type 2 diabetes prevention programs in children: a systematic review and meta-analysis | Seral-Cortes et al., 2021 | INCLUDED |

| Title | Authors (Year) | Decision/Reason for exclusion |
|---|---|---|
| Mobile phone interventions to improve adolescents' physical health: A systematic review and meta‐analysis | Shin et al., 2019 | INCLUDED |
| Efectos de los programas de intervención enfocados al tratamiento del sobrepeso/obesidad infantil y adolescenteEffects of intervention programs focused on the treatment of overweight/obese children and adolescents | Silva et al., 2014 | INCLUDED |
| The Effectiveness of Interventions on Sustained Childhood Physical Activity: A Systematic Review and Meta-Analysis of Controlled Studies | Sims et al., 2015 | INCLUDED |
| Effects of Physical Exercise on Adiponectin, Leptin, and Inflammatory Markers in | Sirico et al., 2018 | INCLUDED |

| Title | Authors (Year) | Decision/Reason for exclusion |
|---|---|---|
| Childhood Obesity: Systematic Review and Meta-Analysis | | |
| Effective Weight Loss for Children: A Meta-analysis of Intervention Studies 2002-2015 | Snethen et al., 2016 | INCLUDED |
| High-Intensity Interval Training and Cardiometabolic Risk Factors in Children: A Meta-analysis | Solera-Martinez et al., 2021 | INCLUDED |
| Beyond bariatric surgery and weight loss medications. A systematic review of the current practice in obesity rehabilitative inpatient programs in adults and pediatrics | Spadaccini et al., 2022 | INCLUDED |
| Treatment of Childhood Obesity: A Systematic Review | Staniford et al., 2011 | INCLUDED |
| Slow and Steady, or Hard and Fast? A Systematic Review and Meta-Analysis of Studies | Steele et al., 2021 | INCLUDED |

| Title | Authors (Year) | Decision/Reason for exclusion |
|---|---|---|
| Comparing Body Composition Changes between Interval Training and Moderate Intensity Continuous Training | | |
| Efficacy of Exercise Intervention for Weight Loss in Overweight and Obese Adolescents: Meta-Analysis and Implications | Stoner et al., 2016 | INCLUDED |
| High-intensity interval training in overweight and obese children and adolescents: systematic review and meta-analysis | Thivel et al., 2018 | INCLUDED |
| Family-based childhood obesity interventions in the UK: a systematic review of published studies | Upton et al., 2014 | INCLUDED |
| Exergames in Childhood Obesity Treatment: A Systematic Review | Valeriani et al., 2021 | INCLUDED |

| Title | Authors (Year) | Decision/Reason for exclusion |
|---|---|---|
| The impact of school-based prevention of overweight on psychosocial well-being of children: Obesity Prevention | van Wijnen et al., 2009 | INCLUDED |
| Physical activity in overweight and obese adolescents: systematic review of the effects on physical fitness components and cardiovascular risk factors | Vasconcellos et al., 2014 | INCLUDED |
| Effectiveness of school-based physical activity and nutrition interventions with direct parental involvement on children's BMI and energy balance-related behaviors - A systematic review | Verjans-Janssen et al., 2018 | INCLUDED |
| The Effect of Diet or Exercise on Visceral Adipose Tissue in Overweight Youth | Vissers et al., 2016 | INCLUDED |
| Effect of Physical Activity Interventions for Girls on | Voskuil et al., 2017 | INCLUDED |

| Title | Authors (Year) | Decision/Reason for exclusion |
|---|---|---|
| Objectively Measured Outcomes: A Systematic Review of Randomized Controlled Trials | | |
| Effects of different exercise types on visceral fat in young individuals with obesity aged 6–24years old: A systematic review and meta-analysis | Wang et al., 2022 | INCLUDED |
| Effectiveness of weight management interventions in children: A targeted systematic review for the USPSTF | Whitlock et al., 2010 | INCLUDED |
| EFFECTIVENESS OF OBESITY INTERVENTION PROGRAMS BASED ON GUIDELINES FOR ADOLESCENT STUDENTS: SYSTEMATIC REVIEW | Wolfa et al., 2019 | INCLUDED |
| Effects and dose-response relationships of exercise | Xu et al., 2022 | INCLUDED |

| Title | Authors (Year) | Decision/Reason for exclusion |
|---|---|---|
| intervention on weight loss in overweight and obese children: a meta-regression and system review | | |
| School‑Based Intervention Programs for Preventing Obesity and Promoting Physical Activity and Fitness: A Systematic Review | Yuksel et al., 2020 | INCLUDED |
| Effectiveness of interventions aiming at reducing sedentary behaviour in a non-surgical population with overweight or obesity: A systematic review and meta-analysis | Zabatiero et al., 2018 | INCLUDED |
| Effects of exercise combined with diet intervention on body composition and serum biochemical markers in adolescents with obesity: a systematic review and meta-analysis | Zhao et al., 2022-a | INCLUDED |

| Title | Authors (Year) | Decision/Reason for exclusion |
|---|---|---|
| A Meta-Analysis of the Effects of Different Training Modalities on the Inflammatory Response in Adolescents with Obesity | Zhao et at., 2022-b | INCLUDED |
| The effect and safety of high-intensity interval training in the treatment of adolescent obesity: a meta-analysis | Zhu et al., 2021 | INCLUDED |
| Effects of physical training on anthropometrics, physical and physiological capacities in individuals with obesity: A systematic review | Zouhal et al., 2020 | INCLUDED |
| Diet, physical activity and behavioural interventions for the treatment of overweight or obese adolescents aged 12 to 17 years | Al‑Khudairy et al 2017 | Duplicate |
| School-Based High-Intensity Interval Training Programs for Promoting Physical Activity | Bento et al., 2021 | Duplicate |

| Title | Authors (Year) | Decision/Reason for exclusion |
|---|---|---|
| and Fitness in Adolescents: A Systematic Review | | |
| Efectos del entrenamiento de la aptitud muscular sobre la adiposidad corporal y el desempeño motriz en niños y jóvenes: un meta-análisis. / Effects of Resistance Training on body fat and motor performance skills in children and adolescents: A Meta-Analysis. | Casas et al., 2018 | Duplicate |
| Effects of physical activity programs on body composition, physical fitness and cardiometabolic parameters in pre-school children: a meta-analysis of randomized controlled trials | Garcia-Hermoso et al, 2020. | Duplicate |
| Family-based, eHealth interventions to reduce | Kemp et al., 2021 | Duplicate |

| Title | Authors (Year) | Decision/Reason for exclusion |
|---|---|---|
| cardiovascular risk: a systematic review | | |
| What impact does physical activity have on obesity in 3- to 6-year-olds? | Ducret et al, 2012. | Ineligible language |
| Sleep health promotion interventions and their effectiveness: An umbrella review | Albakri et al, 2021. | Ineligible study type |
| Sleep Health Promotion Interventions and Their Effectiveness: An Umbrella Review | Albakri et al., 2021 | Ineligible study type |
| Systematic review of paediatric weight management interventions delivered in the home setting | Appelhans et al, 2016. | Ineligible study type |
| Obesity in children | Appelhans et al, 2016. | Ineligible study type |

| Title | Authors (Year) | Decision/Reason for exclusion |
|---|---|---|
| Effects of the FIFA 11+ program on performance, biomechanical measures, and physiological responses: A systematic review | Asgari et al., 2023 | Ineligible study type |
| Efficacy of exercise for treating overweight in children and adolescents: a systematic review | Atlantis et al., 2006 | Ineligible study type |
| Análisis De Las Características Y Los Logros Físico-Motrices De Las Clases Basadas En Actividad Física: Una Revisión Sistemática. / Analysis Of The Characteristics And Physical-Motor Achievements Of The Classes Based On Physical Activity: A Systematic Review. | Ayuso, 2020 | Ineligible study type |
| The Effects of Exercise on BDNF Levels in Adolescents: A | Azevedo et al., 2020 | Ineligible study type |

| Title | Authors (Year) | Decision/Reason for exclusion |
|---|---|---|
| Systematic Review with Meta-Analysis | | |
| The Effect of Diet, Exercise, and Lifestyle Intervention on Childhood Obesity: A Network Meta-Analysis | Bae et al., 2021 | Ineligible study type |
| Comparing interventions with network meta-analysis. | Bagg et al., 2018 | Ineligible study type |
| Overview of meta-analysis on prevention and treatment of childhood obesity | Bahia et al., 2019 | Ineligible study type |
| Effects of core training on dynamic balance stability: A systematic review and meta- | Barrio et al., 2022 | Ineligible study type |
| Association between physical activity, fitness and cardiovascular risk factors in children. Systematic review | Batalau et al., 2013 | Ineligible study type |

| Title | Authors (Year) | Decision/Reason for exclusion |
|---|---|---|
| mHealth Interventions to Reduce Physical Inactivity and Sedentary Behavior in Children and Adolescents: Systematic Review and Meta-analysis of Randomized Controlled Trials | Baumann et al., 2022 | Ineligible study type |
| Effects of resistance training on metabolic fitness in children and adolescents: a systematic review | Benson et al., 2008 | Ineligible study type |
| Interdisciplinarity as a strategy for the prevention of systemic arterial hypertension in children: a systematic review | Bernardi et al., 2017 | Ineligible study type |
| Physical Activity and Cognitive Functioning of Children: A Systematic Review | Bidzan-Bluma et al., 2018 | Ineligible study type |
| Does organized sports participation in childhood and adolescence positively influence health? A review of reviews | Bjørnarå et al., 2021 | Ineligible study type |

| Title | Authors (Year) | Decision/Reason for exclusion |
|---|---|---|
| Association of objectively measured physical activity and bone health in children and | Bland et al., 2020 | Ineligible study type |
| Systematic review of community-based childhood obesity prevention studies | Bleich et al., 2013 | Ineligible study type |
| Interventions to prevent global childhood overweight and obesity: a systematic review | Bleich et al., 2018 | Ineligible study type |
| A comparison of the associations between bone health and three diferent intensities of accelerometer‑derived habitual physical activity in children and adolescents: a systematic review | Brailey et al., 2022 | Ineligible study type |
| Effectiveness of obesity prevention and control policies and programs for children: A | Bramante et al., 2019 | Ineligible study type |

| Title | Authors (Year) | Decision/Reason for exclusion |
|---|---|---|
| systematic review of natural experiment studies | | |
| Systematic Review of Natural Experiments for Childhood Obesity Prevention and Control | Bramante et al., 2019 | Ineligible study type |
| Effectiveness of parent-centred interventions for the prevention and treatment of childhood overweight and obesity in community settings: A systematic review | Bray et al., 2010 | Ineligible study type |
| Evidence Supporting the Essential Components of Physical Education as a Measure of Quality | Bryant et al., 2021 | Ineligible study type |
| Skeletal adaptations associated with pre-pubertal gymnastics participation as determined by DXA and pQCT: a systematic review and meta-analysis | Burt et al., 2013 | Ineligible study type |

| Title | Authors (Year) | Decision/Reason for exclusion |
|---|---|---|
| Physical Activity Interventions for Neurocognitive and Academic Performance in Overweight and Obese Youth: A Systematic Review | Bustamante et al., 2016 | Ineligible study type |
| Effect of childhood obesity prevention programmes on blood lipids: a systematic review and meta-analysis | Cai et al., 2014 | Ineligible study type |
| Effect of childhood obesity prevention programs on blood pressure: a systematic review and meta-analysis | Cai et al., 2014 | Ineligible study type |
| Effects of childhood obesity prevention programs on blood pressure and lipids: A systematic review and meta-analysis | Cai et al., 2014 | Ineligible study type |
| Obesity in children | Canoy et al., 2011 | Ineligible study type |

| Title | Authors (Year) | Decision/Reason for exclusion |
|---|---|---|
| The effect of physical activity on anxiety in children and young people: a systematic review and meta-analysis | Carter et al., 2021 | Ineligible study type |
| Impact of physical activity intervention programs on self-efficacy in youths: a systematic review | Cataldo et al., 2013 | Ineligible study type |
| Motor competence and health related physical fitness in youth: A systematic review | Cattuzzo et al., 2016 | Ineligible study type |
| An investigation of the relationship between new fasting hormone asprosin, obesity and acute-chronic exercise: current systematic review. | Ceylan et al., 2021 | Ineligible study type |
| Effectiveness of family-based weight management interventions for children with | Chai et al., 2019 | Ineligible study type |

| Title | Authors (Year) | Decision/Reason for exclusion |
|---|---|---|
| overweight and obesity: an umbrella review | | |
| Dose Response of Cardiorespiratory Fitness Interventions in Adolescents: A Systematic Review: | Chen et al., 2018 | Ineligible study type |
| Effects of physical activity on heart rate variability in children and adolescents: a systematic review and meta-analysis | Chen et al., 2022 | Ineligible study type |
| A systematic review of interventions for promoting active transportation to school | Chillón et al., 2011 | Ineligible study type |
| Contralateral Effects after Unilateral Strength Training: A Meta-Analysis Comparing Training Loads. | Cirer-Sastre et al., 2017 | Ineligible study type |
| Exercise-associated prevention of adult cardiovascular disease in children and adolescents: | Cooper et al., 2020 | Ineligible study type |

| Title | Authors (Year) | Decision/Reason for exclusion |
|---|---|---|
| monocytes, molecular mechanisms, and a call for discovery | | |
| Rebound Effect Of Intervention Programs To Reduce Overweight And Obesity In Children And Adolescents; Systematic Review | Cordero et al, 2015. | Ineligible study type |
| Iniciativas escolares y deportivas lideradas desde la Fédération Internationale de Football Association (FIFA): revision sistematica | Correa et al., 2015 | Ineligible study type |
| Cardiometabolic risk and health behaviours in adolescents with normal-weight obesity: a systematic review | Cota et al., 2021 | Ineligible study type |
| A systematic review of lifestyle patterns and their association with adiposity in children aged 5–12 years | D'Souza et al., 2020 | Ineligible study type |
| School-based interventions promoting both physical activity and healthy eating in Europe: a systematic review within the HOPE project | De Bourdeaudhuij et al., 2011 | Ineligible study type |
| Interventions with children and parents to improve physical activity and body mass index: a meta-analysis | Dellert et al., 2014 | Ineligible study type |
| Association between cardiorespiratory fitness and depressive symptoms | Donato et al, 2021. | Ineligible study type |
| Physical Activity, Fitness, Cognitive Function, and Academic Achievement in Children: A Systematic Review | Donnelly et al., 2016 | Ineligible study type |
| Effects of dancing on physical activity levels of children and adolescents: a systematic review | Dos Santos et al., 2021 | Ineligible study type |

| Title | Authors (Year) | Decision/Reason for exclusion |
|---|---|---|
| A systematic review investigating the effects of implementing game-based approaches in school-based Physical Education and Sport among Primary school children | Eather et al., 2021 | Ineligible study type |
| ‘Measuring’ Physical Literacy and Related Constructs: A Systematic Review of Empirical Findings | Edwards et al., 2018 | Ineligible study type |
| Sedentary Time and Behavior during School: A Systematic Review and Meta-Analysis | Egan et al., 2019 | Ineligible study type |
| Comparison of accelerometer measured levels of physical activity and sedentary time between obese and non-obese children and adolescents: a systematic review | Elmesmari et al., 2018 | Ineligible study type |
| Impact of recess interventions on children's physical activity--a meta-analysis | Erwin et al., 2014 | Ineligible study type |
| Recent Trends in Sedentary Time: A Systematic Literature Review | Fang et al., 2021 | Ineligible study type |
| Active school transport, physical activity levels and body weight of children and youth: a systematic review | Faulkner et al., 2009 | Ineligible study type |
| The Effects of Physical Activity and Physical Fitness on Children's Achievement and Cognitive Outcomes: A Meta-Analysis | Fedewa et al., 2011 | Ineligible study type |
| Revisión sistemática sobre los estudios de intervención de actividad fisica para el tratamiento de la obesidad. / Systematic Review of Physical | Fernández et al, 2018 | Ineligible study type |

| Title | Authors (Year) | Decision/Reason for exclusion |
|---|---|---|
| Activity Programs for the treatment of Obesity | | |
| Interventions to prevent obesity in children and adolescents: a systematic literature review | Flodmark et al., 2006 | Ineligible study type |
| Capacity-oriented approaches to developing childhood obesity interventions: a systematic review | Foster et al., 2018 | Ineligible study type |
| Increasing weight-bearing physical activity and calcium intake for bone mass growth in children and adolescents: A review of intervention trials | French et al., 2000 | Ineligible study type |
| Community-based interventions to reduce overweight and obesity in China: a systematic review of the Chinese and English literature | Gao et al., 2008 | Ineligible study type |

| Title | Authors (Year) | Decision/Reason for exclusion |
|---|---|---|
| Are field-based exergames useful in preventing childhood obesity? A systematic review | Gao et al., 2014 | Ineligible study type |
| Exercise, health outcomes, and pædiatric obesity: A systematic review of meta-analyses | García-Hermoso et al, 2019 | Ineligible study type |
| Is adherence to the Mediterranean Diet associated with healthy habits and physical fitness? A systematic review and meta-analysis including 565,421 youths | García-Hermoso et al, 2019. | Ineligible study type |
| Is device-measured vigorous-intensity physical activity associated with health-related outcomes in children and adolescents? A systematic review and meta-analysis | García-Hermoso et al, 2021. | Ineligible study type |

| Title | Authors (Year) | Decision/Reason for exclusion |
|---|---|---|
| Exercise, health outcomes, and paediatric obesity: A systematic review of meta-analyses | Garcia-Hermoso et al., 2019 | Ineligible study type |
| Non-pharmacological interventions to reduce the risk of diabetes in people with impaired glucose regulation: a systematic review and economic evaluation | Gillett et al., 2012 | Ineligible study type |
| A Systematic Review of Children's Physical Activity Patterns: Concept, Operational Definitions, Instruments, Statistical Analyses, and Health Implications | Gomes et al., 2020 | Ineligible study type |
| The Effect of Swimming During Childhood and Adolescence on Bone Mineral Density: A Systematic Review and Meta-Analysis | Gomez-Bruton et al., 2016 | Ineligible study type |

| Title | Authors (Year) | Decision/Reason for exclusion |
|---|---|---|
| School and family-based interventions for promoting a healthy lifestyle among children and adolescents in Italy: a systematic review | Gorga et al., 2016 | Ineligible study type |
| Associations of Objectively Measured Vigorous Physical Activity With Body Composition, Cardiorespiratory Fitness, and Cardiometabolic Health in Youth: A Review | Gralla et al., 2016 | Ineligible study type |
| Systematic Review Of Physical Activity And Sedentary Behavior Indicators In South-American Preschool Children | Guerra et al., 2019 | Ineligible study type |
| The Effects of Resistance Training on Blood Pressure in Preadolescents and Adolescents: A Systematic Review and Meta-Analysis | Guillem et al., 2020 | Ineligible study type |

| Title | Authors (Year) | Decision/Reason for exclusion |
|---|---|---|
| Moving beyond the stigma: systematic review of video games and their potential to combat obesity | Guy et al., 2011 | Ineligible study type |
| Physical Activity, Academic Performance and Cognition in Children and Adolescents. A Systematic Review | Haapala, 2012 | Ineligible study type |
| Effectiveness of exercise intervention on improving fundamental movement skills and motor coordination in overweight/obese children and adolescents: A systematic review. | Han et al., 2018 | Ineligible study type |
| Effectiveness of lifestyle interventions in preventing harmful weight gain among adolescents: A systematic review of systematic reviews | Hayba et al., 2020 | Ineligible study type |

| Title | Authors (Year) | Decision/Reason for exclusion |
|---|---|---|
| Effectiveness of lifestyle interventions in preventing harmful weight gain among | Hayba et al., 2021 | Ineligible study type |
| The effect of influencing autonomy for obesity prevention: A review and meta-analysis of school based interventions | Haynes et al., 2019 | Ineligible study type |
| Active Commuting and Physical Fitness: A Systematic Review | Henriques-Neto et al., 2020 | Ineligible study type |
| Active video game on children and adolescents' physical activity and weight | Ho et al., 2022 | Ineligible study type |
| Relationships of physical activity and sedentary behaviour with the previous and subsequent nights' sleep in children and youth: A systematic review and meta-analysis | Huang et al., 2021 | Ineligible study type |

| Title | Authors (Year) | Decision/Reason for exclusion |
|---|---|---|
| A meta-analysis of school-based obesity prevention programs demonstrates limited efficacy of decreasing childhood obesity | Hung et al., 2015 | Ineligible study type |
| A systematic review and meta-analysis of school-based interventions with health education to reduce body mass index in adolescents aged 10 to 19 years | Jacob et al., 2021 | Ineligible study type |
| Systematic review of the health benefits of physical activity and fitness in school-aged children and youth | Janssen et al., 2010 | Ineligible study type |
| Peer-assisted learning in school physical education, sport and physical activity programmes: a systematic review | Jenkinson et al., 2014 | Ineligible study type |
| In search of quality evidence for lifestyle management and | Johnson et al., 2010 | Ineligible study type |

| Title | Authors (Year) | Decision/Reason for exclusion |
|---|---|---|
| glycemic control in children and adolescents with type 2 diabetes: A systematic review | | |
| Nonphysical effects of exergames on child and adolescent well-being: a comprehensive systematic review | Joronen et al., 2017 | Ineligible study type |
| Health-Related Physical Fitness and Activity in Homeschool: A Systematic Review With Implications for Return to Public School | Kabiri et al., 2021 | Ineligible study type |
| Behavioral interventions to prevent childhood obesity: A systematic review and metaanalyses of randomized trials | Kamath et al., 2008 | Ineligible study type |
| Physical activity increases bone mass during growth | Karlsson et al., 2008 | Ineligible study type |

| Title | Authors (Year) | Decision/Reason for exclusion |
|---|---|---|
| Childhood obesity: a global public health crisis | Karnik et al., 2012 | Ineligible study type |
| Physical activity and low back pain in children and adolescents: a systematic review | Kędra et al, 2021. | Ineligible study type |
| How Many US Children and Adolescents with Overweight and Obesity Could Improve Their Percent Body Fat by Exercising?: Meta-Analytic Based Estimates | Kelley et al, 2021. | Ineligible study type |
| Are There Inter-Individual Differences in Fat Mass and Percent Body Fat as a Result of Aerobic Exercise Training in Overweight and Obese Children and Adolescents? A Meta-Analytic Perspective | Kelley et al., 2020 | Ineligible study type |
| Inter-individual differences in body mass index were not observed as a result of aerobic exercise in children and adolescents with overweight and obesity | Kelley et al., 2021 | Ineligible study type |
| E-&mHealth interventions targeting nutrition, physical activity, sedentary behavior, and/or obesity among children: A scoping review of systematic reviews and meta-analyses | Kracht et al., 2021 | Ineligible study type |
| Effect Of Virtual Reality Equipment By Active Video Gaming On Physical Activity. | Kurt et al., 2018 | Ineligible study type |
| Exergaming as a strategic tool in the fight against childhood obesity: a systematic review | Lamboglia et al., 2013 | Ineligible study type |
| The relationship between physical activity and sleep from mid adolescence to early adulthood. A systematic review | Lang et al., 2016 | Ineligible study type |

| **Title** | **Authors (Year)** | **Decision/Reason for exclusion** |
|---|---|---|
| of methodological approaches and meta-analysis | | |
| Physical fitness related to sportive performance in artistic gymnastics. / Aptidão física relacionada ao desempenho esportivo na ginástica artística | Lemos et al., 2017 | Ineligible study type |
| Effects of Resistance Training on Physical Fitness in Healthy Children and Adolescents: An Umbrella Review | Lesinski et al., 2020 | Ineligible study type |
| What works in China? A literature review on effectiveness of current intervention for combating obesity among Chinese school-aged children | Li et al., 2015 | Ineligible study type |
| Effects of Active Videogames on Physical Activity and Related Outcomes Among | Liang et al., 2014 | Ineligible study type |

| **Title** | **Authors (Year)** | **Decision/Reason for exclusion** |
|---|---|---|
| Healthy Children: A Systematic Review | | |
| The effects of high-intensity interval training versus moderate-intensity continuous training on fat loss and cardiometabolic health in pediatric obesity | Liu et al., 2019 | Ineligible study type |
| A Systematic Review of Interventions to Enhance Healthy Lifestyle Behaviors in Adolescents Delivered via Mobile Phone Text Messaging | Loescher et al., 2018 | Ineligible study type |
| Association of blood pressure, obesity and physical activity with arterial stiffness in children: a systematic review and meta-analysis | Lona et al., 2022 | Ineligible study type |
| Soccer helps build strong bones during growth: a systematic review and meta-analysis | Lozano-Berges et al., 2018 | Ineligible study type |

| Title | Authors (Year) | Decision/Reason for exclusion |
|---|---|---|
| The relationship between active travel to school and health-related fitness in children and adolescents: a systematic review | Lubans et al., 2011 | Ineligible study type |
| Neighbourhood speed limit and childhood obesity | Luo et al., 2020 | Ineligible study type |
| Interventions for treating obesity in children | Luttikhuis et al., 2009 | Ineligible study type |
| Chances and Limitations of Video Games in the Fight against Childhood Obesity-A Systematic Review | Mack et al., 2017 | Ineligible study type |
| Weight training in youth-growth, maturation, and safety: an evidence-based review | Malina, 2006 | Ineligible study type |
| Sport and dance interventions for healthy young people (15–24 years) to promote | Mansfield et al., 2018 | Ineligible study type |

| Title | Authors (Year) | Decision/Reason for exclusion |
|---|---|---|
| subjective well-being: a systematic review | | |
| Contribution of Walking to School to Individual and Population Moderate-Vigorous Intensity Physical Activity: Systematic Review and Meta-Analysis | Martin et al., 2016 | Ineligible study type |
| Treatment of pediatric obesity: A systematic review and meta-analysis of randomized trials | McGovern et al., 2008 | Ineligible study type |
| Exergames As A Tool For The Acquisition And Development Of Motor Skills And Abilities: A Systematic Review | Medeiros et al., 2017 | Ineligible study type |
| The effectiveness of intervention on the physical activity of children: Systematic review and meta-analysis of | Metcalf et al., 2011 | Ineligible study type |

| Title | Authors (Year) | Decision/Reason for exclusion |
|---|---|---|
| controlled trials with objectively-measured outcomes | | |
| Republished research: effectiveness of intervention on physical activity of children: systematic review and meta-analysis of controlled trials with objectively measured outcomes (EarlyBird 54) | Metcalf et al., 2012 | Ineligible study type |
| A Review of Systematic Reviews Targeting the Prevention and Treatment of | Militello et al., 2018 | Ineligible study type |
| Review article: the management of paediatric nonalcoholic fatty liver disease | Mitchel et al., 2014 | Ineligible study type |
| Physical Activity in Young Children: A Systematic Review of Parental Influences | Mitchell et al., 2012 | Ineligible study type |
| The school environment and adolescent physical activity and | Morton et al., 2016 | Ineligible study type |

| Title | Authors (Year) | Decision/Reason for exclusion |
|---|---|---|
| sedentary behaviour: a mixed-studies systematic review | | |
| The impact of multicomponent weight management interventions on quality of life in adolescents affected by overweight or obesity: a meta-analysis of randomized controlled trials | Murray et al., 2018 | Ineligible study type |
| Effectiveness of educational interventions conducted in latin america for the prevention of overweight and obesity in scholar children from 6-17 years old; a systematic review | Navarrete et al., 2014 | Ineligible study type |
| A Systematic Review And Meta-Analysis Of The Effects Of Impact Exercise On Bone Structure Across The Lifespan | Ng et al., 2022 | Ineligible study type |

| Title | Authors (Year) | Decision/Reason for exclusion |
|---|---|---|
| A review of electronic interventions for prevention and treatment of overweight and obesity in young people | Nguyen et al., 2011 | Ineligible study type |
| Parental influence on models of primary prevention of cardiovascular disease in children | Norton et al., 2003 | Ineligible study type |
| The Effect of Aerobic Exercise on Recovery in Adolescents who Report Emotional and Cognitive Symptoms after Sport-Related Concussion | Nowak et al, 2022. | Ineligible study type |
| Systematic review of the effectiveness of interventions used in the management of obesity | O'Meara et al., 1998 | Ineligible study type |
| Digital Interventions for Universal Health Promotion in Children and Adolescents: A Systematic Review | Oh et al., 2022 | Ineligible study type |

| Title | Authors (Year) | Decision/Reason for exclusion |
|---|---|---|
| Approaches to Promoting More Widespread Participation in Physical Activity | Owen et al., 1988 | Ineligible study type |
| Access to bike lanes and childhood obesity: A systematic review and meta-analysis | Pan et al., 2021 | Ineligible study type |
| Systematic review of effectiveness of universal self-regulation-based interventions and their effects on distal health and social outcomes in children and adolescents: Review protocol | Pandey et al., 2017 | Ineligible study type |
| After-school interventions to increase physical activity among youth | Pate et al., 2008 | Ineligible study type |
| Physical education and sport in Brazil | Pignone et al., 2001 | Ineligible study type |

| Title | Authors (Year) | Decision/Reason for exclusion |
|---|---|---|
| Psychological Wellbeing in Physical Education and School Sports: A Systematic Review | Piñeiro-Cossio et al., 2021 | Ineligible study type |
| Effects of nutrition, exercise and lifestyle interventions on ghrelin in obese children and adolescents | Poggiogalle et al., 2013 | Ineligible study type |
| Prevention and treatment of childhood and adolescent obesity: a systematic review of meta‐analyses | Psaltopoulou et al., 2019 | Ineligible study type |
| Regular Doses of Nature: The Efficacy of Green Exercise Interventions for Mental Wellbeing | Regerson et al., 2020 | Ineligible study type |
| Physical activity interventions in the prevention and treatment of paediatric obesity: systematic review and critical appraisal | Reilly et al., 2003 | Ineligible study type |

| Title | Authors (Year) | Decision/Reason for exclusion |
|---|---|---|
| Associations between accelerometry measured physical activity and sedentary time and the metabolic syndrome: A meta‐analysis of more than 6000 children and adolescents | Renninger et al., 2020 | Ineligible study type |
| O Efeito Da Atividade Física Na Composição Corporal E Aptidão Cardiorrespiratória: Uma Revisão Sistemática. / The effect of physical activity on body composition and cardiorespiratory fitness: A systematic review | Rosário et al., 2019 | Ineligible study type |
| Combinations of physical activity, sedentary time, and sleep duration and their associations with depressive symptoms and other mental health problems in children and adolescents: a systematic review | Sampasa-Kanyinga et al., 2020 | Ineligible study type |

| Title | Authors (Year) | Decision/Reason for exclusion |
|---|---|---|
| Health Literacy in Adolescents and Young Adults: An Updated Review | Sansom-Daly et al., 2016 | Ineligible study type |
| Body composition changes after weight-loss interventions for overweight and obesity | Santarpia et al., 2013 | Ineligible study type |
| Recreational football is medicine against non-communicable diseases: A systematic review. | Sarmento et al., 2020 | Ineligible study type |
| What are the health benefits of active travel? A systematic review of trials and cohort studies | Saunders et al., 2013 | Ineligible study type |
| Efficacy of interventions that use apps to improve diet, physical activity and sedentary behaviour: a systematic review | Schoeppe et al., 2016 | Ineligible study type |

| Title | Authors (Year) | Decision/Reason for exclusion |
|---|---|---|
| School-based obesity interventions: a literature review | Shaya et al., 2008 | Ineligible study type |
| Jump rope training effects on health- and sport-related physical fitness in young participants: A systematic review with meta-analysis | Singh et al., 2022 | Ineligible study type |
| Biomechanical variations in children who are overweight and obese during high-impact activities: A systematic review and meta-analysis | Spech et al., 2022 | Ineligible study type |
| Exercise Dose and Weight Loss in Adolescents with Overweight–Obesity: A Meta-Regression | Stoner et al., 2019 | Ineligible study type |
| Culturally Competent Interventions to Address Obesity Among African | Suarez-Balcazar et al., 2013 | Ineligible study type |

| Title | Authors (Year) | Decision/Reason for exclusion |
|---|---|---|
| American and Latino Children and Youth | | |
| Contribution of structured settings to time spent in physical activity and sedentary in youth: systematic review and meta-analysis | Tassitano et al., 2020 | Ineligible study type |
| European normative values for physical fitness in children and adolescents aged 9–17 years: results from 2 779 165 Eurofit performances representing 30 countries | Tomkinson et al., 2018 | Ineligible study type |
| Non-alcoholic fatty liver disease in children and adolescents: Efficacy of lifestyle change-a systematic review and meta-analysis | Utz-Melere et al., 2018 | Ineligible study type |
| Assessing and Increasing Physical Activity | Van Camp et al., 2012 | Ineligible study type |

| Title | Authors (Year) | Decision/Reason for exclusion |
|---|---|---|
| An online family-based self-monitoring and goal-setting intervention to improve children's physical activity: the FRESH feasibility trial and three-arm pilot RCT | van Sluijs et al., 2021 | Ineligible study type |
| Childhood obesity: prevention and strategies of intervention. A systematic review of school-based interventions in primary schools | Verrotti et al., 2014 | Ineligible study type |
| Interventions in outside-school hours childcare settings for promoting physical activity amongst schoolchildren aged 4 to 12 years | Virgara et al., 2021 | Ineligible study type |
| The effect of high Intensity interval training versus moderate intensity continuous training on arterial stiffness and 24h blood pressure responses: A | Way et al., 2019 | Ineligible study type |

| Title | Authors (Year) | Decision/Reason for exclusion |
|---|---|---|
| systematic review and meta-analysis. | | |
| Unravelling the association between accelerometer‐derived physical activity and adiposity among preschool children: A systematic review and meta‐analyses | Wiersma et al., 2020 | Ineligible study type |
| Combinations of Physical Activity, Sedentary Behavior, and Sleep Duration and Their Associations With Physical, Psychological, and Educational Outcomes in Children and Adolescents: A Systematic Review | Wilhite et al., 2023 | Ineligible study type |
| Systematic review and meta-analysis of the association between childhood overweight and obesity and primary school diet and physical activity policies | Willliams et al., 2013 | Ineligible study type |

| Title | Authors (Year) | Decision/Reason for exclusion |
|---|---|---|
| Before-school physical activity programs and youth physical activity, health and learning-related outcomes: a systematic review. | Woodforde et al., 2021 | Ineligible study type |
| Associations between physical activity, sedentary behaviour and self-rated health among the general population of children and adolescents: a systematic review and meta-analysis | Zhang et al., 2020 | Ineligible study type |
| Magnitude of Muscle Strength and Mass Adaptations Between High-Load Resistance Training Versus Low-Load Resistance Training Associated with Blood-Flow Restriction: A Systematic Review and Meta-Analysis. | Lixandrão et al, 2018. | Ineligible population |
| School-Based Interventions Targeting Nutrition and Physical Activity, and Body | Adom et al, 2019. | Ineligible population |

| Title | Authors (Year) | Decision/Reason for exclusion |
|---|---|---|
| Weight Status of African Children: A Systematic Review | | |
| "Kids Get in Shape with Nature": A Systematic Review Exploring the Impact of Green Spaces on Childhood Obesity | Alejandre et al, 2020. | Ineligible population |
| High-Intensity Interval Training upon Cognitive and Psychological Outcomes in Youth: A Systematic Review | Alves et al, 2021. | Ineligible population |
| The effectiveness of incidental physical activity interventions compared to other interventions in the management of people with low back pain: A systematic review and meta-analysis of randomised controlled trials. | Alzahrani et al, 2019. | Ineligible population |

| Title | Authors (Year) | Decision/Reason for exclusion |
|---|---|---|
| Active School-Based Interventions to Interrupt Prolonged Sitting Improve Daily Physical Activity: A Systematic Review and Meta-Analysis | Amor-Barbosa et al, 2022. | Ineligible population |
| School-related physical activity interventions and mental health among children: a systematic review and meta-analysis | Andermo et al, 2020. | Ineligible population |
| Comparing the effects of variable and traditional resistance training on maximal strength and muscle power in healthy adults: A systematic review and meta-analysis. | Andersen et al, 2022. | Ineligible population |
| Systematic Review of Setting-Based Interventions for Preventing Childhood Obesity. | Angawi et al, 2021. | Ineligible population |
| Physical activity and sleep are inconsistently related in healthy | Antczak et al, 2020. | Ineligible population |

| Title | Authors (Year) | Decision/Reason for exclusion |
|---|---|---|
| children: A systematic review and meta-analysis | | |
| Skeletal Muscle Glycogen Content at Rest and During Endurance Exercise in Humans: A Meta-Analysis. | Areta et al, 2018. | Ineligible population |
| Aerobic fitness and its relationship to sport, exercise training and habitual physical activity during youth | Armstrong et al, 2011. | Ineligible population |
| Family-based childhood obesity prevention interventions: a systematic review and quantitative content analysis | Ash et al, 2017. | Ineligible population |
| Change in Central Cardiovascular Function in Response to Intense Interval Training: A Systematic Review and Meta-analysis | Astorino et al, 2022. | Ineligible population |
| Interventions to promote physical activity in young people conducted in the hours immediately after school: a systematic review | Atkin et al, 2011. | Ineligible population |
| Design features associated with engagement in mHealth physical activity interventions among youth: A systematic review of qualitative and quantitative studies | Ayla Schwarz et al. 2023 | Ineligible population |
| Physical activity and physical self-concept in youth: systematic review and meta-analysis | Babic et al, 2014. | Ineligible population |
| Effects of Taekwondo Training on Body Composition: A Systematic Review and Meta-Analysis | Baek et al, 2021. | Ineligible population |

| Title | Authors (Year) | Decision/Reason for exclusion |
|---|---|---|
| Combined Diet and Physical Activity Promotion Programs to Prevent Type 2 Diabetes Among Persons at Increased Risk: A Systematic Review for the Community Preventive Services Task Force | Balk et al, 2015. | Ineligible population |
| The Effects of Exclusive Walking on Lipids and Lipoproteins in Women with Overweight and Obesity: A Systematic Review and Meta-Analysis. | Ballard et al, 2022. | Ineligible population |
| Digital exercise interventions for improving measures of central obesity: a systematic review | Ballin et al, 2020. | Ineligible population |
| Effects Of Combat Sports On Bone Mass: Systematic Review. /Efecto De Los Deportes De Combate Sobre La Masa Ósea: Revisión Sistemática. | Barbeta et al, 2019. | Ineligible population |

| Title | Authors (Year) | Decision/Reason for exclusion |
|---|---|---|
| Football can tackle type 2 diabetes: a systematic review of the health effects of recreational football practice in individuals with prediabetes and type 2 diabetes. | Barbosa et al, 2021. | Ineligible population |
| School-based high-intensity interval training programs in children and adolescents: A Systematic Review and Meta-Analysis. | Barker et al, 2022. | Ineligible population |
| Active video games for youth: a systematic review | Barnett et al, 2011. | Ineligible population |
| Through the Looking Glass: A Systematic Review of Longitudinal Evidence, Providing New Insight for Motor Competence and Health | Barnett et al, 2022. | Ineligible population |
| Effects of High-Intensity Interval Training in School on the Physical Performance and | Bauer et al, 2022. | Ineligible population |

| Title | Authors (Year) | Decision/Reason for exclusion |
|---|---|---|
| Health of Children and Adolescents: A Systematic Review with Meta-Analysis | | |
| Individualization and effectiveness of mobile health interventions to reduce physical inactivity and sedentary behavior in healthy children and adolescents: A systematic review and meta-analysis | Baumann et al. 2022 | Ineligible population |
| Resistance Training Effects on Metabolic Function Among Youth: A Systematic Review | Bea et al, 2017. | Ineligible population |
| Effectiveness of diet and physical activity interventions among Chinese-origin populations living in high income countries: a systematic review | Beasley et al, 2020. | Ineligible population |

| Title | Authors (Year) | Decision/Reason for exclusion |
|---|---|---|
| Efficacy of hamstring stretching programs in schoolchildren. A systematic review | Becerra Fernandez et al, 2015. | Ineligible population |
| Effect of Physical Education-based stretching programs on hamstring extensibility in high school students: A systematic review./ Efecto de los programas de estiramiento en Educación Física sobre la extensibilidad de la musculatura isquiosural en estudiantes de Educación Secundaria: Una revisión sistemática. | Becerra-Fernández et al, 2020. | Ineligible population |
| After-school program impact on physical activity and fitness: a meta-analysis | Beets et al, 2009. | Ineligible population |
| Effects of Strength Training Using Unstable Surfaces on Strength, Power and Balance Performance Across the | Behm et al, 2015, | Ineligible population |

| Title | Authors (Year) | Decision/Reason for exclusion |
|---|---|---|
| Lifespan: A Systematic Review and Meta-analysis | | |
| Effects of resistance training in children and adolescents: a meta-analysis | Behringer et al, 2010. | Ineligible population |
| Effects of weight-bearing activities on bone mineral content and density in children and adolescents: a meta-analysis | Behringer et al, 2014. | Ineligible population |
| Effectiveness of physical activity programs in enhancing sleep outcomes among adolescents: a systematic review | Bello et al, 2023. | Ineligible population |
| Types of Interventions Targeting Dietary, Physical Activity, and Weight-Related Outcomes among University Students: A Systematic Review of Systematic Reviews | Belogianni et al, 2019. | Ineligible population |

| Title | Authors (Year) | Decision/Reason for exclusion |
|---|---|---|
| Family-Based Interventions Targeting Childhood Obesity: A Meta-Analysis | Berge et al, 2011. | Ineligible population |
| Active video games to promote physical activity in children and youth: a systematic review | Biddiss et al, 2010. | Ineligible population |
| The effectiveness of interventions to increase physical activity among young girls: a meta-analysis | Biddle et al, 2014. | Ineligible population |
| Clinical Applications of Yoga for the Pediatric Population: A Systematic Review | Birdee et al, 2009. | Ineligible population |
| Effects of Strength Training on the Physiological Determinants of Middle- and Long-Distance Running Performance: A Systematic Review. | Blagroove et al, 2018. | Ineligible population |
| Motivational Interviewing for Parent-child Health | Borrelli et al, 20215. | Ineligible population |

| Title | Authors (Year) | Decision/Reason for exclusion |
|---|---|---|
| Interventions: A Systematic Review and Meta-Analysis | | |
| Prevention of Type 2 Diabetes among Youth: A Systematic Review, Implications for the School Nurse | Brackney et al, 2015. | Ineligible population |
| Resistance Training and Handball Players' Isokinetic, Isometric and Maximal Strength, Muscle Power and Throwing Ball Velocity: A Systematic Review and Meta-Analysis | Bragazzi et al, 2020. | Ineligible population |
| A rapid review of the effect of The Daily Mile on children | Breslin et al, 2022. | Ineligible population |
| Temporal Trends in the Standing Broad Jump Performance of United States Children and Adolescents | Bridget et al, 2021. | Ineligible population |

| Title | Authors (Year) | Decision/Reason for exclusion |
|---|---|---|
| The Effectiveness of Sedentary Behaviour Reduction Workplace Interventions on Cardiometabolic Risk Markers: A Systematic Review. | Brierley et al, 2019. | Ineligible population |
| School-based interventions to improve spinal health of children and adolescents: a systematic review | Brink et al, 2022. | Ineligible population |
| A systematic review of intervention effects on potential mediators of children's physical activity | Brown et al, 2013. | Ineligible population |
| A Systematised Review of Primary School Whole Class Child Obesity Interventions: Effectiveness, Characteristics, and Strategies | Brown et al, 2016. | Ineligible population |
| Interventions for preventing obesity in children | Brown et al, 2019. | Ineligible population |

| Title | Authors (Year) | Decision/Reason for exclusion |
|---|---|---|
| Effects of Workplace-Based Physical Activity Interventions on Cardiorespiratory Fitness: A Systematic Review and Meta-Analysis of Controlled Trials. | Burn et al, 2019. | Ineligible population |
| Moderators of School-Based Physical Activity Interventions on Cardiorespiratory Endurance in Primary School-Aged Children: A Meta-Regression | Burns et al, 2018. | Ineligible population |
| Healthier Minds in Fitter Bodies: A Systematic Review and Meta‑Analysis of the Association between Physical Fitness and Mental Health in Youth | Cademas-Sanchez et al, 2021, | Ineligible population |
| Interventions to Promote Physical Activity among Young and Adolescent Girls: A Systematic Review | Camacho-Minano et al, 2011. | Ineligible population |

| Title | Authors (Year) | Decision/Reason for exclusion |
|---|---|---|
| Interventions for preventing obesity in childhood. A systematic review | Campbell et al, 2001. | Ineligible population |
| Effects of exercise interventions on body image: a meta-analysis | Campbell et al, 2009. | Ineligible population |
| Interventions for preventing obesity in children | Campbell et al, 2019. | Ineligible population |
| Effects of aerobic exercise on obese children with metabolic syndrome: a systematic | Cao et al, 2021. | Ineligible population |
| A meta-analysis on the effects of exercise training on the VO2max in children and adolescents. / Meta análisis de los efectos del entrenamiento en el VO2máx en niños y adolescentes | Carazo-Vargas et al, 2015. | Ineligible population |
| Do Interventions to Increase Walking Work? A Systematic | Carlin et al, 2016. | Ineligible population |

| Title | Authors (Year) | Decision/Reason for exclusion |
|---|---|---|
| Review of Interventions in Children and Adolescents | | |
| Getting patients to exercise more: a systematic review of underserved populations | Carroll et al, 2008. | Ineligible population |
| Muscle hypertrophy and strength gains after resistance training with different volume-matched loads: a systematic review and meta-analysis. | Carvalho et al, 2022. | Ineligible population |
| Adoption, implementation and sustainability of school-based physical activity and sedentary behaviour interventions in real-world settings: a systematic review | Cassar et al, 2019. | Ineligible population |
| Long-Term Dietary and Physical Activity Interventions in the School Setting and Their Effects on BMI in Children | Cerrato-Carretero et al, 2021. | Ineligible population |

| Title | Authors (Year) | Decision/Reason for exclusion |
|---|---|---|
| Aged 6–12 Years: Meta-Analysis of Randomized Controlled Clinical Trials | | |
| Effects of Resistance Training on Change‑of‑Direction Speed in Youth and Young Physically Active and Athletic Adults: A Systematic Review with Meta‑Analysis | Chaabene et al, 2020. | Ineligible population |
| Effectiveness of school-based eHealth interventions to prevent multiple lifestyle risk behaviours among adolescents: a systematic review and meta-analysis | Champion et al, 2019. | Ineligible population |
| A systematic review of parent-based interventions to improve multiple lifestyle risk behaviours among adolescents and parents | Champion et al, 2022. | Ineligible population |

| Title | Authors (Year) | Decision/Reason for exclusion |
|---|---|---|
| Effects of Regular Physical Activity on the Immune System, Vaccination and Risk of Community-Acquired Infectious Disease in the General Population: Systematic Review and Meta-Analysis. | Chastin et al, 2021. | Ineligible population |
| School-based obesity prevention interventions in Latin America: A systematic review | Chavez et al, 2020. | Ineligible population |
| Is exercise a senolytic medicine? A systematic review | Chen et al., 2021. | Ineligible population |
| Acute effect of breaking up prolonged sitting on cognition: a systematic review | Chueh et al, 2022. | Ineligible population |
| Effective Interventions for Improving Functional Movement Screen Scores Among "High-Risk" Athletes: A Systematic Review. | Clark et al, 2022. | Ineligible population |

| Title | Authors (Year) | Decision/Reason for exclusion |
|---|---|---|
| Effects of high-intensity interval training in men soccer player's physical fitness: A systematic review with meta-analysis of randomized-controlled and non-controlled trials | Clemente et al, 2021, | Ineligible population |
| Effects of plyometric jump training on soccer player's balance: a systematic review and meta-analysis of randomized-controlled trials | Clemente et al, 2021. | Ineligible population |
| Increasing activity to reduce obesity in adolescent girls: a research review | Clemmens et al, 2004. | Ineligible population |
| The impact of child and adolescent obesity treatment interventions on physical activity: a systematic review | Cliff et al, 2010. | Ineligible population |
| Impact of non-diet approaches on attitudes, behaviors, and | Clifford et al, 2015. | Ineligible population |

| Title | Authors (Year) | Decision/Reason for exclusion |
|---|---|---|
| health outcomes: a systematic review | | |
| The effect of resistance training interventions on fundamental movement skills in youth: a meta-analysis | Collins et al, 2019. | Ineligible population |
| Muscle Fiber Hypertrophy and Myonuclei Addition: A Systematic Review and Meta-analysis. | Conceição et al, 2018. | Ineligible population |
| Time Trends in Physical Activity Using Wearable Devices: A Systematic Review and Meta-analysis of Studies from 1995 to 2017 | Conger et al, 2022. | Ineligible population |
| Impact of physical activity interventions on anthropometric outcomes: systematic review and meta-analysis | Conn et al, 2014. | Ineligible population |

| Title | Authors (Year) | Decision/Reason for exclusion |
|---|---|---|
| A systematic review of controlled trials of interventions to prevent childhood obesity and overweight: a realistic synthesis of the evidence | Connelly et al, 2007. | Ineligible population |
| Right heart exercise‐training‐adaptation and remodelling in endurance athletes | Conti et al, 2021. | Ineligible population |
| Physical activity programmes to reduce overweight and obesity in children and adolescents; a systematic review | Cordero et al, 2014. | Ineligible population |
| Effects of Strength Training on Blood Pressure and Heart Rate Variability—A Systematic Review | Corso et al, 2022. | Ineligible population |
| High-intensity interval training for improving health-related fitness in adolescents: a | Costigan et al, 2015. | Ineligible population |

| Title | Authors (Year) | Decision/Reason for exclusion |
|---|---|---|
| systematic review and meta-analysis | | |
| Eficacy of School‑Based Interventions for Improving Muscular Fitness Outcomes in Adolescent Boys: A Systematic Review and Meta‑analysis | Cox et al, 2020. | Ineligible population |
| A meta-analytic review of eHealth interventions for pediatric health promoting and maintaining behaviors | Cushing et al, 2010. | Ineligible population |
| Systematic review and meta-analysis of health promotion interventions for children and adolescents using an ecological framework | Cushing et al, 2014. | Ineligible population |
| The Effect of Nordic Hamstring Exercise Intervention Volume on Eccentric Strength and Muscle Architecture | Cuthbert et al, 2020. | Ineligible population |

| Title | Authors (Year) | Decision/Reason for exclusion |
|---|---|---|
| Adaptations: A Systematic Review and Meta-analyses. | | |
| The effect of physical training on heart rate variability in healthy children: a systematic review with meta-analysis | da Silva et al, 2014. | Ineligible population |
| The impacts of unstructured nature play on health in early childhood development: A systematic review | Dankiw et al, 2020. | Ineligible population |
| Chronic Effects of Altering Resistance Training Set Configurations Using Cluster Sets: A Systematic Review and Meta-Analysis. | Davies et al, 2021. | Ineligible population |
| Impact of training modes on fitness and body composition in women with obesity: A systematic review and meta-analysis. | Davis et al, 2022. | Ineligible population |

| Title | Authors (Year) | Decision/Reason for exclusion |
|---|---|---|
| Exercise programs may be effective in preventing a new episode of neck pain: a systematic review and meta-analysis. | De Campos et al, 2018. | Ineligible population |
| Interventions for promoting physical activity among European teenagers: a systematic review | De Meester et al, 2009. | Ineligible population |
| Small-sided games in volleyball: A systematic review of the state of the art. | de Oliveira Castro et al, 2022. | Ineligible population |
| Universal and selective interventions to promote good mental health in young people: Systematic review and meta-analysis | de Pablo et al, 2020. | Ineligible population |
| Physical activity interventions in the school setting: A systematic review | Demetriou et al, 2012. | Ineligible population |

| Title | Authors (Year) | Decision/Reason for exclusion |
|---|---|---|
| Interventions on children's and adolescents' physical activity and sedentary behaviour: protocol for a systematic review from a sex/gender perspective | Demetriou et al, 2019 | Ineligible population |
| Effects of plyometric training on skill and physical performance in healthy tennis players: A systematic review and meta-analysis | Deng et al, 2022. | Ineligible population |
| The effect of aquatic high-intensity interval training on aerobic performance, strength and body composition in a non-athletic population: systematic review and meta-analysis. | Depiazzi et al, 2019. | Ineligible population |
| The Impact of Sex on Left Ventricular Cardiac Adaptations to Endurance Training: a Systematic Review and Meta-analysis. | Diaz-Canestro et al, 2020. | Ineligible population |

| Title | Authors (Year) | Decision/Reason for exclusion |
|---|---|---|
| Influence of exclusive resistance training on body composition and cardiovascular risk factors in overweight or obese children: a systematic review | Dietz et al, 2012. | Ineligible population |
| School-based physical activity programs for promoting physical activity and fitness in children and adolescents aged 6 to 18 (Review) | Dobbins et al, 2013. | Ineligible population |
| Smartphone-Based Interventions for Physical Activity Promotion: Scoping Review of the Evidence Over the Last 10 Years | Domin et al, 2021. | Ineligible population |
| Slackline Training (Balancing Over Narrow Nylon Ribbons) and Balance Performance: A Meta-Analytical Review | Donath et al, 2017. | Ineligible population |

| Title | Authors (Year) | Decision/Reason for exclusion |
|---|---|---|
| Chronic Adaptations to Eccentric Training: A Systematic Review | Douglas et al, 2017. | Ineligible population |
| The Effectiveness of Gait Retraining on Running Kinematics, Kinetics, Performance, Pain, and Injury in Distance Runners: A Systematic Review With Meta-analysis. | Doyle et al, 2022. | Ineligible population |
| A Systematic Review of the Effectiveness of Physical Education and School Sport Interventions Targeting Physical Activity, Movement Skills and Enjoyment of Physical Activity | Dudley et al, 2025. | Ineligible population |
| Effective physical education and school sport: A systematic review of physical education and school sport interventions targeting physical activity, | Dudley et al, 2025. | Ineligible population |

| **Title** | **Authors (Year)** | **Decision/Reason for exclusion** |
|---|---|---|
| movement skills and enjoyment of physical activity | | |
| Effects of weight-neutral approaches compared with traditional weight-loss approaches on behavioral, physical, and psychological health outcomes: a systematic review and meta-analysis. | Dugmore et al, 2020. | Ineligible population |
| Monitoring and adapting endurance training on the basis of heart rate variability monitored by wearable technologies: A systematic review with meta-analysis. | Duing et al, 2021. | Ineligible population |
| School-based high-intensity interval training programs in children and adolescents: A systematic review and meta-analysis | Duncombe et al, 2022. | Ineligible population |

| **Title** | **Authors (Year)** | **Decision/Reason for exclusion** |
|---|---|---|
| School-based high-intensity interval training programs in children and adolescents: A systematic review and meta-analysis | Duncombe et al, 2022. | Ineligible population |
| School-based high-intensity interval training programs in children and adolescents: A systematic review and meta-analysis | Duncombe et al, 2022. | Ineligible population |
| Preventative interventions that target cardiovascular dysfunction in children and young people: a systematic review of their effectiveness and an investigation of sexual dimorphism | Edwards et al, 2023. | Ineligible population |
| Sedentary Time and Behavior during School: A Systematic Review and Meta-Analysis. | Egan et al, 2019. | Ineligible population |

| Title | Authors (Year) | Decision/Reason for exclusion |
| --- | --- | --- |
| Exercise to improve self-esteem in children and young people | Ekeland et al, 2004. | Ineligible population |
| Can exercise improve self esteem in children and young people? A systematic review of randomised controlled trials | Ekeland et al, 2005. | Ineligible population |
| Exploring the Relationship Between Fundamental Motor Skill Interventions and Physical Activity Levels in Children: A Systematic Review and Meta-analysis | Engel et al, 2018. | Ineligible population |
| Exploring the Relationship Between Fundamental Motor Skill Interventions and Physical Activity Levels in Children: A Systematic Review and Meta-analysis. | Engel et al, 2018. | Ineligible population |
| High-Intensity Interval Training Performed by Young Athletes: | Engel et al, 2018. | Ineligible population |

| Title | Authors (Year) | Decision/Reason for exclusion |
| --- | --- | --- |
| A Systematic Review and Meta-Analysis | | |
| High-Intensity Interval Training Performed by Young Athletes: A Systematic Review and Meta-Analysis | Engel et al, 2018. | Ineligible population |
| Effect of Plyometric Training on Sport Performance in Adolescent Overhead Athletes: A Systematic Review | Eraslan et al, 2021. | Ineligible population |
| Systematic Review of Physical Education-Based Physical Activity Interventions Among Elementary School Children | Errisuriz et al, 2018. | Ineligible population |
| Improvement of the lipid profile with exercise in obese children: a systematic review | Escalante et al, 2012. | Ineligible population |
| Exercise-Based Strategies to Prevent Muscle Injury in Elite Footballers: A Systematic | Fachini et al, 2020. | Ineligible population |

| Title | Authors (Year) | Decision/Reason for exclusion |
|---|---|---|
| Review and Best Evidence Synthesis. | | |
| Hiit, Resistance Training, And Risk Factors In Adolescents: A Systematic Review. / Hiit, Entrenamiento Resistido Y Factores De Riesgo En Adolescentes: Una Revisión Sistemática | Farie et al, 2020. | Ineligible population |
| Longitudinal changes in moderate‐to‐vigorous‐intensity physical activity in children and adolescents: A systematic review and meta‐analysis | Farooq et al, 2020. | Ineligible population |
| Mobile Health Interventions for Improving Health Outcomes in Youth: A Meta-analysis | Fedele et al, 2017. | Ineligible population |
| Exercise and insulin resistance in youth: a meta-analysis | Fedewa et al, 2014. | Ineligible population |

| Title | Authors (Year) | Decision/Reason for exclusion |
|---|---|---|
| Associations between meeting 24-hour movement guidelines and health in the early | Feng et al, 2021. | Ineligible population |
| Revisión sistemática sobre los estudios de intervención de actividad física para el tratamiento de la obesidad. / Systematic Review of Physical Activity Programs for the treatment of Obesity. | Fernández et al, 2018. | Ineligible population |
| Effects of chronic exercise on the inhibitory control of children and adolescents: A systematic review and meta-analysis | Fernández et al, 2021. | Ineligible population |
| Effects of Vest and Sled Resisted Sprint Training on Sprint Performance in Young Soccer Players: A Systematic Review and Meta-analysis. | Fernández-Galván et al, 2022. | Ineligible population |

| Title | Authors (Year) | Decision/Reason for exclusion |
|---|---|---|
| Are There Benefits from Teaching Yoga at Schools? A Systematic Review of Randomized Control Trials of Yoga-Based Interventions | Ferreira-Vorkapic et al, 2015. | Ineligible population |
| Effects of physical exercise on myelin sheath regeneration: A systematic review and meta-analysis. | Feter et al, 2018. | Ineligible population |
| Effectiveness of centre-based childcare interventions in increasing child physical activity: a systematic review and meta-analysis for policymakers and practitioners | Finch et al, 2016. | Ineligible population |
| The Effect of Classroom-Based Interventions on Sedentary Behavior and Spinal Health in Schoolchildren:Systematic Review | Fisher et al, 2022. | Ineligible population |

| Title | Authors (Year) | Decision/Reason for exclusion |
|---|---|---|
| Entrenamiento de la capacidad aerobia en prepúberes. Revisión sistemática. / Trainability of aerobic capacity in prepubescent: Systematic review. | Flores et al, 2019. | Ineligible population |
| Self-report use-of-time tools for the assessment of physical activity and sedentary behaviour in young people: systematic review | Foley et al, 2012. | Ineligible population |
| Treatment Interventions for Early Childhood Obesity: A Systematic Review | Foster et al, 2015. | Ineligible population |
| Impact of isolated aerobic exercise in obese adolescents: systematic review | Fragnani et al, 2017. | Ineligible population |
| Effects Of High-Intensity Interval Training On Olympic Combat Sports Athletes' Performance And Physiological | Franchini et al, 2019. | Ineligible population |

| Title | Authors (Year) | Decision/Reason for exclusion |
|---|---|---|
| Adaptation: A Systematic Review | | |
| Can chronic stretching change the muscle‐tendon mechanical properties? A review. | Freitas et al, 2018. | Ineligible population |
| Effect of interventions on the body mass index of school-age students | Friedrich et al, 2012. | Ineligible population |
| The Effects of Concurrent Strength and Endurance Training on Physical Fitness and Athletic Performance in Youth: A Systematic Review and Meta-Analysis | Gabler et al, 2018. | Ineligible population |
| A meta-analysis of active video games on health outcomes among children and adolescents | Gao et al, 2015. | Ineligible population |
| A Systematic Review of Active Video Games on Youth's Body | Gao et al, 2020. | Ineligible population |

| Title | Authors (Year) | Decision/Reason for exclusion |
|---|---|---|
| Composition and Physical Activity | | |
| Effects of exercise on resting blood pressure in obese children: a meta-analysis of randomized controlled trials | Garcia-Hermoso et al, 2013. | Ineligible population |
| Effects of physical education interventions on cognition and academic performance | Garcia-Hermoso et al, 2021. | Ineligible population |
| Effects of Resistance Training on Arterial Stiffness in Healthy People: A Systematic Review. | García-Mateo et al, 2020. | Ineligible population |
| Effects and Dose–Response Relationship of Balance Training on Balance Performance in Youth: A Systematic Review and Meta-Analysis | Gebel et al, 2018. | Ineligible population |
| Effectiveness of Adult Health Promotion Interventions | George et al, 2022. | Ineligible population |

| Title | Authors (Year) | Decision/Reason for exclusion |
|---|---|---|
| Delivered Through Professional Sport: Systematic Review and Meta-Analysis | | |
| Pediatric Obesity Prevention and Treatment Among Hispanics: A Systematic Review and Meta-Analysis | George et al, 2022. | Ineligible population |
| Interventions addressing general parenting to prevent or treat childhood obesity | Gerards et al, 2011. | Ineligible population |
| Systematic Review: Nutrition and Physical Activity in the Management of Paediatric Nonalcoholic Fatty Liver Disease | Gibson et al, 2017. | Ineligible population |
| Comparing Active Pediatric Obesity Treatments Using Meta-Analysis | Gilles et al, 2008. | Ineligible population |
| A systematic review of school based interventions aimed at the promotion of physical activity and/or healthy dietary behaviours in adolescents | Gilmartin et al, 2012. | Ineligible population |
| The treatment and prevention of obesity: a systematic review of the literature | Glenny et al, 1997. | Ineligible population |
| The Effects of Physical Activity and Diet Interventions on Body Mass Index in Latin American Children and Adolescents: A Systematic Review and Meta-Analysis | Godoy-Cumillaf et al, 2020. | Ineligible population |
| Are primary/elementary school-based interventions effective in preventing/ameliorating excess weight gain? A systematic review of systematic reviews | Goldthorpe et al 2020. | Ineligible population |
| Plyometric exercise and bone health in children and | Gomez-Bruton et al, 2017. | Ineligible population |

| Title | Authors (Year) | Decision/Reason for exclusion |
|---|---|---|
| adolescents: a systematic review | | |
| Physical activity promotion in children and adolescents using peer support: Systematic review of the literature | Goncalves et al, 2013. | Ineligible population |
| Hybridizing pedagogical models: A systematic review | González-Villora et al, 2019. | Ineligible population |
| Effectiveness of educational and lifestyle interventions to prevent paediatric obesity: systematic review and meta-analyses of randomized and non-randomized controlled trials | Gori et al, 2017. | Ineligible population |
| Biomarkers of Physiological Responses to Periods of Intensified, Non-Resistance-Based Exercise Training in Well-Trained Male | Greenham et al, 2018. | Ineligible population |

| Title | Authors (Year) | Decision/Reason for exclusion |
|---|---|---|
| Athletes: A Systematic Review and Meta-Analysis. | | |
| Effects of resistance training performed to repetition failure or non-failure on muscular strength and hypertrophy: A systematic review and meta-analysis. | Grgic et al , 2022. | Ineligible population |
| Does Aerobic Training Promote the Same Skeletal Muscle Hypertrophy as Resistance Training? A Systematic Review and Meta-Analysis. | Grgic et al 2019. | Ineligible population |
| Effects of Open versus Closed Skill Exercise on Cognitive Function: A Systematic Review | Gu et al, 2018. | Ineligible population |
| School-based intervention practices to reduce body mass index: Meta-analysis of 57 randomized trials | Guerra et al, 2013. | Ineligible population |

| Title | Authors (Year) | Decision/Reason for exclusion |
|---|---|---|
| Effects of community health worker-based interventions on physical activity levels in children: a systematic review | Guerra et al, 2021. . | Ineligible population |
| Effectiveness of intervention programs in schools to reduce health risk factors in adolescents: a systematic review. / Efetividade de programas de intervenção escolar para reduzir fatores de risco à saúde em adolescentes: uma revisão sistemática | Guimarães et al, 2015. | Ineligible population |
| Effects of Classroom Active Desks on Children and Adolescents' Physical Activity, Sedentary Behavior, Academic Achievements and Overall Health: A Systematic Review | Guirrado et al, 2021. | Ineligible population |
| Effect of Resistance Training Methods and Intensity on the Adolescent Swimmer's | Guo et al, 2022. | Ineligible population |

| Title | Authors (Year) | Decision/Reason for exclusion |
|---|---|---|
| Performance: A Systematic Review | | |
| The Effect of Resistance Training in Women on Dynamic Strength and Muscular Hypertrophy: A Systematic Review with Meta-analysis. | Hagstrom et al, 2020. | Ineligible population |
| Effects of CrossFit intervention on students' physical fitness in physical education: a systematic review and meta-analysis. | Han et al., 2021. | Ineligible population |
| Interventions to prevent weight gain: a systematic review of psychological models and behaviour change methods | Hardeman et al, 2000. | Ineligible population |
| Resistance training to improve power and sports performance in adolescent athletes: a systematic review and meta-analysis | Harries et al, 2012. | Ineligible population |

| Title | Authors (Year) | Decision/Reason for exclusion |
|---|---|---|
| Systematic review and meta-analysis of linear and undulating periodized resistance training programs on muscular strength | Harries et al, 2015. | Ineligible population |
| Reducing aggression with martial arts: A meta-analysis of child and youth studies | Harwood et al, 2017. | Ineligible population |
| Comparative Effectiveness of Physical Activity Intervention Programs on Motor Skills in Children and Adolescents: A Systematic Review and Network Meta-Analysis | Hassan et al, 2022. | Ineligible population |
| Effectiveness of Lifestyle Interventions for Prevention of Harmful Weight Gain among Adolescents from Ethnic Minorities: A Systematic Review | Hayba et al, 2020. | Ineligible population |

| Title | Authors (Year) | Decision/Reason for exclusion |
|---|---|---|
| School-based Interventions to Reduce Sedentary Behaviour in Children: A Systematic Review | Hegarty et al, 2016. | Ineligible population |
| Combined Home and School Obesity Prevention Interventions for Children: What Behavior Change Strategies and Intervention Characteristics Are Associated With Effectiveness? | Hendrie et al, 2012. | Ineligible population |
| The effect of diet or exercise on ectopic adiposity in children and adolescents with obesity: a systematic review and meta-analysis | Hens et al, 2017. | Ineligible population |
| Systematic review of the efficacy of the prescription of physical activity in obese child population | Hernandez et al, 2015. | Ineligible population |
| The Effectiveness of Aquatic Plyometric Training in | Heywood et al, 2022. | Ineligible population |

| Title | Authors (Year) | Decision/Reason for exclusion |
|---|---|---|
| Improving Strength, Jumping, and Sprinting: A Systematic Review. | | |
| Electronic media-based health interventions promoting behavior change in youth: a systematic review | Hieftje et al, 2013. | Ineligible population |
| Weight-bearing exercise and bone mineral accrual in children and adolescents: a review of controlled trials | Hind et al, 2007. | Ineligible population |
| Effectiveness of lifestyle interventions in child obesity: systematic review with meta-analysis | Ho et al, 2012. | Ineligible population |
| Physical activity interventions in Latin America: expanding and classifying the evidence | Hoehner et al, 2013. | Ineligible population |
| School-wide programs aimed at obesity among Latino youth in | Holub et al, 2014. | Ineligible population |

| Title | Authors (Year) | Decision/Reason for exclusion |
|---|---|---|
| the United States: a review of the evidence | | |
| Effect of Pilates Intervention on Physical Function of Children and Youth: A Systematic Review. | Hornsby et al, 2020. | Ineligible population |
| A Majority of Anterior Cruciate Ligament Injuries Can Be Prevented by Injury Prevention Programs: A Systematic Review of Randomized Controlled Trials and Cluster–Randomized Controlled Trials With Meta-analysis. | Huang et al, 2020. | Ineligible population |
| Exergame-based exercise training for depressive symptoms in adults: A systematic review and meta-analysis | Huang et al, 2022. | Ineligible population |
| Effect modification by cardiorespiratory fitness on the | Husøy et al, 2021. | Ineligible population |

| Title | Authors (Year) | Decision/Reason for exclusion |
|---|---|---|
| association between physical activity and cardiometabolic health in youth: A systematic review | | |
| Does physical fitness moderate the effect of physical activity on cardiometabolic health in children?: a systematic review | Husøy et al. 2021. | Ineligible population |
| Systematic review of recess interventions to increase physical activity | Ickes et al, 2013. | Ineligible population |
| Effectiveness of Mobile Apps to Promote Health and Manage Disease: Systematic Review and Meta-analysis of Randomized Controlled Trials | Inbarren et al, 2021. | Ineligible population |
| Interventions that impact weight status in Hispanic preschool children | Innella et al, 2020 | Ineligible population |

| Title | Authors (Year) | Decision/Reason for exclusion |
|---|---|---|
| Effects of weight-bearing exercise on bone health in girls: a meta-analysis | Ishikawa, et al, 2013. | Ineligible population |
| Use of Mobile Phone App Interventions to Promote Weight Loss: Meta-Analysis | Islam et al, 2020, | Ineligible population |
| Physical Activity and Cognitive Development: A Meta-Analysis | Jackson et al, 2016. | Ineligible population |
| A systematic review of primary healthcare provider education and training using the Chronic Care Model for childhood obesity | Jacobson et al, 2011. | Ineligible population |
| A systematic review of interventions to increase physical activity and reduce sedentary behaviour following bariatric surgery. | James et al, 2022. | Ineligible population |
| Yoga as an Intervention for the Reduction of Symptoms of | James-Palmer et al, 2020. | Ineligible population |

| Title | Authors (Year) | Decision/Reason for exclusion |
| --- | --- | --- |
| Anxiety and Depression in Children and Adolescents: A Systematic Review | | |
| The effects of exercise interventions on physical function tests and glycemic control in adults with type 2 diabetes: A systematic review. | Janssen et al, 2021. | Ineligible population |
| The Acute and Chronic Effects of Implementing Velocity Loss Thresholds During Resistance Training: A Systematic Review, Meta-Analysis, and Critical Evaluation of the Literature. | Jikic et al, 2023. | Ineligible population |
| Effects of Multicomponent Injury Prevention Programs on Children and Adolescents' Fundamental Movement Skills: A Systematic Review With Meta-Analyses | Jimenez-Garcia et al, 2023. | Ineligible population |

| Title | Authors (Year) | Decision/Reason for exclusion |
| --- | --- | --- |
| Nature-Based Early Childhood Education and Children's Physical Activity, Sedentary Behavior, Motor Competence, and Other Physical Health | Johnstone et al, 2022. | Ineligible population |
| The effectiveness of interventions to increase physical activity. A systematic review | Kahn et al, 2002 | Ineligible population |
| Clinical review: behavioral interventions to prevent childhood obesity: a systematic review and metaanalyses of randomized trials | Kamath et al, 2008. | Ineligible population |
| Effect of pedometer-based physical activity interventions: a meta-analysis | Kang et al, 2009. | Ineligible population |
| Systematic review of text messaging as an intervention for adolescent obesity | Keating et al, 2015. | Ineligible population |

| Title | Authors (Year) | Decision/Reason for exclusion |
|---|---|---|
| A systematic review and meta-analysis of interval training versus moderate-intensity continuous training on body adiposity | Keating et al, 2017. | Ineligible population |
| Controlling childhood obesity: A systematic review on strategies and challenges | Kelishadi et al, 2014. | Ineligible population |
| The effects of exercise on resting blood pressure in children and adolescents: a meta-analysis of randomized controlled trials | Kelley et al, 2003. | Ineligible population |
| Exercise and resting blood pressure in children and adolescents: A meta-analysis | Kelley et al, 2008. | Ineligible population |
| Exercise and BMI in Overweight and Obese Children and Adolescents: A Systematic Review and Trial Sequential Meta-Analysis | Kelley et al, 2015. | Ineligible population |

| Title | Authors (Year) | Decision/Reason for exclusion |
|---|---|---|
| Exercise and BMI z-score in Overweight and Obese Children and Adolescents: A Systematic Review and Network Meta-Analysis of Randomized Trials | Kelley et al, 2017. | Ineligible population |
| Prevention of unhealthy weight in children by promoting physical activity using a socio-ecological approach: what can we learn from intervention studies? | Kellou et al, 2014. | Ineligible population |
| Systematic review of multicomponent interventions with overweight middle adolescents: implications for clinical practice and research | Kelly et al, 2008. | Ineligible population |
| Improving youth physical, mental and social health through physical activity: A | Kemel et al, 2022. | Ineligible population |

| Title | Authors (Year) | Decision/Reason for exclusion |
|---|---|---|
| A systematic review exploring body image programmes and interventions in physical education | Kerner et al, 2022. | Ineligible population |
| A systematic review to determine the effectiveness of interventions designed to prevent overweight and obesity in pre-adolescent girls | Kesten et al, 2011. | Ineligible population |
| The impact of high-intensity interval training on inflammatory markers in metabolic disorders: A meta-analysis. | Khalafi et al, 2020. | Ineligible population |
| Effect of resistance training with and without caloric restriction on visceral fat: A systemic review and meta-analysis | Khalafi et al, 2021. | Ineligible population |
| High-intensity interval exercise versus moderate-intensity | Khalafi et al, 2022. | Ineligible population |

| Title | Authors (Year) | Decision/Reason for exclusion |
|---|---|---|
| continuous exercise on postprandial glucose and insulin responses: A systematic review and meta-analysis | | |
| The impact of exercise training versus caloric restriction on inflammation markers: a systematic review and meta-analysis. | Khalafi et al, 2022. | Ineligible population |
| The Natural History of Full-Thickness Rotator Cuff Tears in Randomized Controlled Trials: A Systematic Review and Meta-analysis. | Khatri et al, 2019. | Ineligible population |
| Effects of Accumulated Short Bouts of Exercise on Weight and Obesity Indices in Adults: A Meta-Analysis | Kim et al, 2020. | Ineligible population |
| Family-based child weight management intervention in | Kim et al, 2020. | Ineligible population |

| Title | Authors (Year) | Decision/Reason for exclusion |
|---|---|---|
| early childhood in low-income families: A systematic review | | |
| Lifestyle interventions for youth who are overweight: a meta-analytic review | Kitzamann et al, 2010. | Ineligible population |
| Childhood Obesity Prevention in Africa: A Systematic Review of Intervention Effectiveness and Implementation | Klingberg et al, 2019. | Ineligible population |
| Systematic review of school-based obesity interventions targeting African American and Hispanic children | Knowlden et al, 2013. | Ineligible population |
| The Effects of Thai Yoga on Physical Fitness: A Meta-Analysis of Randomized Control Trials | Kongkaew et al, 2018. | Ineligible population |
| BONE GEOMETRY AND PHYSICAL ACTIVITY IN CHILDREN AND | Krahenbuhl et al, 2018. | Ineligible population |

| Title | Authors (Year) | Decision/Reason for exclusion |
|---|---|---|
| ADOLESCENTS: SYSTEMATIC REVIEW | | |
| Moderators of environmental intervention effects on diet and activity in youth | Kremers et al, 2007. | Ineligible population |
| A Systematic Review of Multi-Component Comprehensive School Physical Activity Program (CSPAP) Interventions | Kuhn et al, 2021. | Ineligible population |
| Chronic Physiological Effects of Swim Training Interventions in Non-Elite Swimmers: A Systematic Review and Meta-Analysis | Lahart et al, 2018. | Ineligible population |
| The Effects of Green Exercise on Physical and Mental Wellbeing: A Systematic Review | Lahart et al, 2019. | Ineligible population |

| Title | Authors (Year) | Decision/Reason for exclusion |
|---|---|---|
| The Effectiveness of Physical Activity-Promoting Web- and Mobile-Based Distance Weight Loss Interventions on Body Composition in Rehabilitation Settings: Systematic Review, Meta-analysis, and Meta-Regression Analysis | Lahtio et al, 2022. | Ineligible population |
| Systematic review of the relationship between 20m shuttle run performance and health indicators among children and youth | Lang et al, 2018. | Ineligible population |
| The WHO Health Promoting School framework for improving the health and well-being of students and their academic achievement | Langford et al, 2014. | Ineligible population |
| The effectiveness of exercise interventions to prevent sports injuries: a systematic review | Lauersen et al, 2014. | Ineligible population |

| Title | Authors (Year) | Decision/Reason for exclusion |
|---|---|---|
| and meta-analysis of randomised controlled trials | | |
| Systematic review and meta-analysis of school-based interventions to reduce body mass index | Lavelle et al, 2012. | Ineligible population |
| Review of High-Intensity Interval Training for Cognitive and Mental Health in Youth | Leahy et al, 2020. | Ineligible population |
| A Systematic Review and Meta-Analysis of Intervention for Pediatric Obesity Using Mobile Technology | Lee et al, 2016. | Ineligible population |
| Effects of Pokémon GO on Physical Activity and Psychological and Social Outcomes: A Systematic Review | Lee et al, 2021. | Ineligible population |
| The Effectiveness of Classical Ballet Training on | Letton et al, 2020. | Ineligible population |

| Title | Authors (Year) | Decision/Reason for exclusion |
|---|---|---|
| Health-Related Outcomes: A Systematic Review | | |
| A systematic review of school-based intervention studies for the prevention or reduction of excess weight among Chinese children and adolescents | Li et al, 2008. | Ineligible population |
| The effect of acute and chronic exercise on cognitive function and academic performance in adolescents: A systematic review | Li et al, 2017. | Ineligible population |
| Wearable activity trackers for promoting physical activity: A systematic meta-analytic review | Li et al, 2021. | Ineligible population |
| The Effects of Physical Activity on Positive Emotions in Children and Adolescents: A Systematic Review and Meta-Analysis | Li et al, 2022. | Ineligible population |

| Title | Authors (Year) | Decision/Reason for exclusion |
|---|---|---|
| Which type of sedentary behaviour intervention is more effective at reducing body mass index in children? A meta-analytic review | Liao et al, 2014. | Ineligible population |
| Effect of Exercise on Risk Factors of Diabetic Foot Ulcers: A Systematic Review and Meta-Analysis. | Liao et al, 2019. | Ineligible population |
| Effects of isometric, eccentric, or heavy slow resistance exercises on pain and function in individuals with patellar tendinopathy: A systematic review. | Lim et al, 2018. | Ineligible population |
| Effects of school-based neuromuscular training on fundamental movement skills and physical fitness in children: a systematic review | Lin et al, 2022. | Ineligible population |

| Title | Authors (Year) | Decision/Reason for exclusion |
|---|---|---|
| Quadriceps Strength and Volitional Activation After Anterior Cruciate Ligament Reconstruction: A Systematic Review and Meta-analysis. | Lisee et al, 2019. | Ineligible population |
| Effect of eccentric overload training on change of direction speed performance: A systematic review and meta-analysis. | Liu et al, 2020. | Ineligible population |
| Effects of Acute and Chronic Exercises on Executive Function in Children and Adolescents: A Systemic Review and Meta-Analysis | Liu et al, 2020. | Ineligible population |
| The Effects of Tai Chi and Qigong Exercise on Psychological Status in Adolescents: A Systematic Review and Meta-Analysis | Liu et al, 2021. | Ineligible population |

| Title | Authors (Year) | Decision/Reason for exclusion |
|---|---|---|
| A systematic review and meta-analysis of interventions designed to increase moderate-to-vigorous physical activity in school physical education lessons | Lonsdale et al, 2013. | Ineligible population |
| Effects Of School-Based Physical Activity And Nutrition Programs In Spanish Adolescents: Systematic Review | López et al, 2015. | Ineligible population |
| Resistance Training Load Effects on Muscle Hypertrophy and Strength Gain: Systematic Review and Network Meta-analysis. | Lopez et al, 2021. | Ineligible population |
| Moderators of Resistance Training Effects in Overweight and Obese Adults: A Systematic Review and Meta-analysis | Lopez et al, 2022. | Ineligible population |

| Title | Authors (Year) | Decision/Reason for exclusion |
| --- | --- | --- |
| Improving cognition in school children and adolescents through exergames. A systematic review and practical guide | López-Serrano et al, 2021. | Ineligible population |
| The Effects of Physical Education on Motor Competence in Children and Adolescents: A Systematic Review and Meta-Analysis | Lorås et al, 2020. | Ineligible population |
| Equity effects of children's physical activity interventions: a systematic scoping review | Love et al, 2017. | Ineligible population |
| Parent-only interventions for childhood overweight or obesity in children aged 5 to 11 years | Loveman et al, 2015. | Ineligible population |
| Review: A systematic review of the impact of physical activity programmes on social and emotional well-being in at-risk youth | Lubans et al, 2012. | Ineligible population |

| Title | Authors (Year) | Decision/Reason for exclusion |
| --- | --- | --- |
| Meta-analysis of interventions to prevent overweight and obesity in children and adults | Luckner et al., 2019 | Ineligible population |
| Acute effects of moderate aerobic exercise on specific aspects of executive function in different age and fitness groups: A meta-analysis | Ludyga et al, 2016. | Ineligible population |
| Systematic review and meta-analysis investigating moderators of long-term effects of exercise on cognition in healthy individuals | Ludyga et al, 2016. | Ineligible population |
| A systematic review and meta-analysis of the overall effects of school-based obesity prevention interventions and effect differences by intervention components | Lui et al, 2019. | Ineligible population |
| Interventions for treating obesity in children | Luttikhuis et al, 2009. | Ineligible population |

| Title | Authors (Year) | Decision/Reason for exclusion |
|---|---|---|
| The effectiveness of low intensity exercise and blood flow restriction without exercise on exercise induced muscle damage: A systematic review. | Ma et al, 2020. | Ineligible population |
| The effect of exercise interventions on resting metabolic rate: A systematic review and meta-analysis. | MacKenzie-Shalders et al, 2020. | Ineligible population |
| Digital technology use by and with young children: A systematic review for the Statement on Young Children and Digital Technologies | Mantila et al, 2019. | Ineligible population |
| Efficacy of dietary intervention or in combination with exercise on primary prevention of cardiovascular disease: A systematic review | Manuela-Abbate et al, 2020. | Ineligible population |
| Single Leg Balance Training: A Systematic Review. | Marcori et al, 2022. | Ineligible population |

| Title | Authors (Year) | Decision/Reason for exclusion |
|---|---|---|
| Physical activity and health-related quality of life in children and adolescents: A systematic review and meta-analysis | Marker et al, 2018. | Ineligible population |
| Family-based interventions for reducing sedentary time in youth: a systematic review of randomized controlled trials | Marsh et al, 2014. | Ineligible population |
| Weight-related child behavioral interventions in Brazil: a systematic review | Marshall et al, 2013. | Ineligible population |
| Effects of aerobic, resistance, and combined exercise training on insulin resistance markers in overweight or obese children and adolescents: A systematic review and meta-analysis | Marson et al, 2016. | Ineligible population |
| Impact of weight loss interventions on patient‐reported outcomes in | Martenstyn et al, 2020. | Ineligible population |

| Title | Authors (Year) | Decision/Reason for exclusion |
| --- | --- | --- |
| overweight and obese adults with type 2 diabetes: a systematic review | | |
| Effective behaviour change techniques in the prevention and management of childhood obesity | Martin et al, 2013. | Ineligible population |
| Physical activity, diet and other behavioural interventions for improving cognition and school achievement in children and adolescents with obesity or overweight | Martin et al, 2018. | Ineligible population |
| The effect of dietary (poly)phenols on exercise-induced physiological adaptations: A systematic review and meta-analysis of human intervention trials. | Martinez-Negrin et al, 2022. | Ineligible population |

| Title | Authors (Year) | Decision/Reason for exclusion |
| --- | --- | --- |
| Revisión sistemática del entrenamiento de fuerza en futbolistas preadolescentes y adolescentes. / Systematic review of strength training in preadolescent and adolescent football players. | Martínez-Pérez et al, 2020. | Ineligible population |
| Evaluation of school-based interventions of active breaks in primary schools: A systematic review and meta-analysis | Masini et al, 2020. | Ineligible population |
| Evaluation of school-based interventions including extracurricular activities and homeworks promoting healthy lifestyle (physical activity, healthy diet and sleep hygiene) in children and adolescents: feasibility, effectiveness and sustainability over time. A systematic review. | Masini et al, 2024 | Ineligible population |

| Title | Authors (Year) | Decision/Reason for exclusion |
| --- | --- | --- |
| Evaluation of feasibility, effectiveness, and sustainability of school-based physical activity “active break” interventions in pre-adolescent and adolescent students: a systematic review | Massini et al, 2022. | Ineligible population |
| Evaluating the Effectiveness of Gamification on Physical Activity: Systematic Review and Meta-analysis of Randomized Controlled Trials | Mazeas et al, 2022. | Ineligible population |
| Impact of physical activity/ structured exercise programs on bone health in pre-school children: a systematic review | McCaskie et al. 2022. | Ineligible population |
| Prevention of Type 2 Diabetes in U.S. Hispanic Youth: A Systematic Review of Lifestyle Interventions | McCurley et al, 2017. | Ineligible population |

| Title | Authors (Year) | Decision/Reason for exclusion |
| --- | --- | --- |
| Specific Strategies for Promotion of Physical Activity in Kids-Which Ones Work? A Systematic Review of the Literature | McDonald et al, 2015. | Ineligible population |
| Clinical review: treatment of pediatric obesity: a systematic review and meta-analysis of randomized trials | McGovern et al, 2008. | Ineligible population |
| A Systematic Literature Review of Peer-led Strategies for Promoting Physical Activity Levels of Adolescents | McHale et al, 2022. | Ineligible population |
| A Systematic Literature Review of Peer-led Strategies for Promoting Physical Activity Levels of Adolescents | McHale et al, 2022. | Ineligible population |
| Classroom-Based Physical Activity and Sedentary Behavior Interventions in | McMichan et al, 2018. | Ineligible population |

| Title | Authors (Year) | Decision/Reason for exclusion |
|---|---|---|
| Adolescents: A Systematic Review and Meta-Analysis | | |
| Diet, physical activity and behavioural interventions for the treatment of overweight or obese children from the age of 6 to 11 years | Mead et al, 2017. | Ineligible population |
| Influence of chronic stretching on muscle performance: Systematic review | Medeiros et al, 2017. | Ineligible population |
| Exercise in school Physical Education increase bone mineral content and density: systematic review and meta-analysis | Mello et al, 2022. | Ineligible population |
| Exercise attenuates bone mineral density loss during diet-induced weight loss in adults with overweight and obesity: A systematic review and meta-analysis. | Mesinovic et al, 2021. | Ineligible population |

| Title | Authors (Year) | Decision/Reason for exclusion |
|---|---|---|
| Effectiveness of intervention on physical activity of children: systematic review and meta-analysis of controlled trials with objectively measured outcomes (EarlyBird 54) | Metcalf et al, 2012. | Ineligible population |
| How effective are physical activity intervention programmes in children? Systematic review and metaanalysis of controlled trials with objectively-measured outcomes | Metcalf et al, 2012. | Ineligible population |
| The effectiveness of intervention on the physical activity of children: Systematic review and metaanalysis of controlled trials with objectively measured outcomes | Metcalf et al, 2012. | Ineligible population |
| Recreational Football and Bone Health: A Systematic Review and Meta-analysis | Milanović et al, 2022. | Ineligible population |

| Title | Authors (Year) | Decision/Reason for exclusion |
|---|---|---|
| Psychosocial and Physiological Health Outcomes of Green Exercise in Children and Adolescents—A Systematic Review | Mnich et al, 2019. | Ineligible population |
| Effect of interventions based on regular physical activity on weight management in adolescents: a systematic review and a meta-analysis | Moeini et al, 2021. | Ineligible population |
| DE-PASS Best Evidence Statement (BESt) - modifiable determinants, their association with and effect on physical activity and sedentary behaviour in children and adolescents aged 5-19 years: systematic reviews and meta-analyses | Mohammed Khudair et al, 2022. | Ineligible population |
| The effect of martial arts training on mental health outcomes: A systematic review and meta-analysis. | Moorte et al, 2020. | Ineligible population |
| Age-Related Variation in Male Youth Athletes' Countermovement Jump After Plyometric Training: A Meta-Analysis of Controlled Trials | Moran et al, 2017. | Ineligible population |
| Effects of Bilateral and Unilateral Resistance Training on Horizontally Orientated Movement Performance: A Systematic Review and Meta-analysis. | Moran et al, 2021. | Ineligible population |
| Effects of Vertically and Horizontally Orientated Plyometric Training on Physical Performance: A Meta-analytical Comparison. | Moran et al, 2021. | Ineligible population |
| Comparison of Weightlifting, Traditional Resistance Training | Morris et al, 2022. | Ineligible population |

| Title | Authors (Year) | Decision/Reason for exclusion |
|---|---|---|
| and Plyometrics on Strength, Power and Speed: A Systematic Review with Meta-Analysis. | | |
| Home- and Community-Based Interventions for Physical Activity and Early Child Development: A Systematic Review of Effective Strategies | Moss et al, 2022. | Ineligible population |
| The Effectiveness of Interventions to Reduce Sedentary Time in Different Target Groups and Settings in Germany: Systematic Review, Meta-Analysis and Recommendations on Interventions | Mugler et al, 2022. | Ineligible population |
| Weight loss in children and adolescents | Muhlig et al, 2014. | Ineligible population |
| Nutrition, Physical Activity, and New Technology Programs on Obesity Prevention in Primary | Navidad et al, 2021. | Ineligible population |

| Title | Authors (Year) | Decision/Reason for exclusion |
|---|---|---|
| Education: A Systematic Review | | |
| Effect of exercise on intima-media thickness in children and adolescents with obesity: A meta-analysis | Ngai et al, 2015. | Ineligible population |
| The Training of Medium- to Long-Distance Sprint Performance in Football Code Athletes: A Systematic Review and Meta-analysis. | Nicholson et al, 2022. | Ineligible population |
| Parent participation in weight-related health interventions for children and adolescents: a systematic review and meta-analysis | Niemeier et al, 2012. | Ineligible population |
| Targeted exercise against osteoporosis: A systematic review and meta-analysis for optimising bone strength throughout life | Nikander et al, 2010. | Ineligible population |

| Title | Authors (Year) | Decision/Reason for exclusion |
|---|---|---|
| Exercise to improve pediatric bone and fat: a systematic review and meta-analysis | Nogueira et al 2014. | Ineligible population |
| A review of eHealth interventions for physical activity and dietary behavior change | Norman et al, 2007. | Ineligible population |
| Physically active lessons in schools and their impact on physical activity, educational, health and cognition outcomes: a systematic review and meta-analysis | Norris et al, 2020. | Ineligible population |
| Active Video Games in Schools and Effects on Physical Activity and Health: A Systematic Review | Norris et al., 2016 | Ineligible population |
| Engaging parents to increase youth physical activity a systematic review | O'Connor et al, 2009. | Ineligible population |
| Physical Activity for the Treatment of Adolescent Depression: A Systematic Review and Meta-Analysis | Oberste et al, 2020. | Ineligible population |
| Interventions to Promote Physical Activity and Healthy Digital Media Use in Children and Adolescents: A Systematic Review | Oh et al, 2022. | Ineligible population |
| Interventions to promote walking: systematic review | Olgivie et al, 2007, | Ineligible population |
| Physical Activity–Based Interventions Using Electronic Feedback May Be Ineffective in Reducing Pain and Disability in Patients With Chronic Musculoskeletal Pain: A Systematic Review With Meta-Analysis. | Oliveira et al, 2018. | Ineligible population |
| Effects of active video games on children and adolescents: A | Oliveira et al, 2020. | Ineligible population |

| **Title** | **Authors (Year)** | **Decision/Reason for exclusion** |
|---|---|---|
| systematic review with meta‐analysis | | |
| Isometric training and long‐term adaptations: Effects of muscle length, intensity, and intent: A systematic review. | Oranchuk et al, 2019. | Ineligible population |
| Comparison of the effects of velocity-based vs. traditional resistance training methods on adaptations in strength, power, and sprint speed: A systematic review, meta-analysis, and quality of evidence appraisal | Orange et al, 2022. | Ineligible population |
| Review of Physical Activity and the IGF Family | Orenstein et al, 2004. | Ineligible population |
| Shaping Pathways to Child Health: A Systematic Review of Street-Scale Interventions in City Streets | Ortegon-Sanchez et al, 2022. | Ineligible population |

| **Title** | **Authors (Year)** | **Decision/Reason for exclusion** |
|---|---|---|
| Is ankle plantar flexor strength associated with balance in healthy adults? A systematic review and meta-analysis. | Oskouei et al, 2021. | Ineligible population |
| Clinical outcomes to exercise training in type 1 diabetes: A systematic review and meta-analysis | Ostman et al, 2018. | Ineligible population |
| Ghrelin Response to Acute and Chronic Exercise: Insights and Implications from a Systematic Review of the Literature | Ouerghi et al, 2021. | Ineligible population |
| The effectiveness of school-based physical activity interventions for adolescent girls: A systematic review and meta-analysis | Owen et al, 2017. | Ineligible population |
| Childhood obesity: a (re) programming disease? | Paes et al, 2016. | Ineligible population |

| Title | Authors (Year) | Decision/Reason for exclusion |
|---|---|---|
| Do active video games benefit the motor skill development of non-typically developing children and adolescents: A systematic review | Page et al, 2017. | Ineligible population |
| Health game interventions to enhance physical activity self-efficacy of children: a quantitative systematic review | Pakarien et al, 2017. | Ineligible population |
| Commercial Off-The-Shelf Video Games for Reducing Stress and Anxiety: Systematic Review | Pallavicini et al, 2021, | Ineligible population |
| Effects of exercise motivations on body image and eating habits/behaviours: A systematic review | Pañao et al, 2020. | Ineligible population |
| Effectiveness of Universal Self-regulation-Based Interventions in Children and | Pandey et al, 2018. | Ineligible population |

| Title | Authors (Year) | Decision/Reason for exclusion |
|---|---|---|
| Adolescents: A Systematic Review and Meta-analysis | | |
| Understanding the Benefits of Brief Classroom-Based Physical Activity Interventions on Primary School-Aged Children's Enjoyment and Subjective Wellbeing: A Systematic Review | Papadopoulos et al, 2022. | Ineligible population |
| Do exercises used in injury prevention programmes modify cutting task biomechanics? A systematic review with meta-analysis | Pappas et al, 2015. | Ineligible population |
| The effect of school recess interventions on physical activity : a systematic review | Parrish et al, 2013. | Ineligible population |
| Interventions to Change School Recess Activity Levels in Children and Adolescents: A | Parrish et al, 2020. | Ineligible population |

| Title | Authors (Year) | Decision/Reason for exclusion |
|---|---|---|
| Systematic Review and Meta‑Analysis | | |
| Effects of Acute Physical Exercise With Low and High Cognitive Demands on Executive Functions in Children: A Systematic Review | Paschen et al, 2019. | Ineligible population |
| Active Breaks and Cognitive Performance in Pupils: A Systematic Review. | Pastor-Vicedo et al, 2021. | Ineligible population |
| Recreos activos como estrategia de promoción de la actividad física: una revisión sistemática. / Active recess as a strategy to promote physical activity: a systematic review. | Pastor-Vicedo et al, 2021. | Ineligible population |
| Physical Activity and Health in Children under 6 Years of Age: A Systematic Review | Pate et al, 2019. | Ineligible population |

| Title | Authors (Year) | Decision/Reason for exclusion |
|---|---|---|
| The Relationship Between Non-elite Sporting Activity and Calcaneal Bone Density in Adolescents and Young Adults: A Narrative Systematic Review | Patel et al, 2020. | Ineligible population |
| Self-Monitoring via Digital Health in Weight Loss Interventions: A Systematic Review Among Adults with Overweight or Obesity. | Patel et al, 2021. | Ineligible population |
| The Effects of Acute Exposure to Prolonged Sitting, with and Without Interruption, on Peripheral Blood Pressure Among Adults: A Systematic Review and Meta-Analysis | Paterson et al, 2022. | Ineligible population |
| Resistance training in children and youth: a meta-analysis | Payne 1997. | Ineligible population |
| Exercise and VO2 max in children: a meta-analysis | Payne et al, 1993. | Ineligible population |

| Title | Authors (Year) | Decision/Reason for exclusion |
|---|---|---|
| The effectiveness of interventions to increase physical activity among adolescent girls: a meta-analysis | Pearson et al, 2015. | Ineligible population |
| Effects of In-Classroom Physical Activity Breaks on Children’s Academic Performance, Cognition, Health Behaviours and Health Outcomes: A Systematic Review and Meta-Analysis of Randomised Controlled Trials | Peiris et al, 2022. | Ineligible population |
| Prevention of overweight and obesity in children and youth: a systematic review and meta-analysis | Peirson et al, 2015. | Ineligible population |
| Treatment of overweight and obesity in children and youth: a systematic review and meta-analysis | Peirson et al, 2015. | Ineligible population |

| Title | Authors (Year) | Decision/Reason for exclusion |
|---|---|---|
| A systematic review on the effects of resistance and plyometric training on physical fitness in youth- What do comparative studies tell us? | Peitz et al, 2018. | Ineligible population |
| Using Active Video Games for Physical Activity Promotion: A Systematic Review of the Current State of Research | Peng et al, 2013. | Ineligible population |
| Meta-analysis and systematic review of physical activity on neurodevelopment disorders, depression, and obesity among children and adolescents | Peng et al, 2022. | Ineligible population |
| Promoting health-related cardiorespiratory fitness in physical education: A systematic review | Peralta et al, 2020. | Ineligible population |
| Associations between outdoor play features and childrenâ€™s | Pereira et al, 2024. | Ineligible population |

| Title | Authors (Year) | Decision/Reason for exclusion |
|---|---|---|
| behavior and health: a systematic review | | |
| The effect of augmented feedback on the performance and learning of gross motor and sport-specific skills: A systematic review | Petancesvski et al, 2022. | Ineligible population |
| Development of Maximal Dynamic Strength During Concurrent Resistance and Endurance Training in Untrained, Moderately Trained, and Trained Individuals: A Systematic Review and Meta-analysis. | Petré et al, 2021, | Ineligible population |
| Effectiveness of Physical-Activity-Based Interventions Targeting Overweight and Obesity among University Students—A Systematic Review | Pfisterer et al, 2022. | Ineligible population |

| Title | Authors (Year) | Decision/Reason for exclusion |
|---|---|---|
| Associations Among K–12 Student Outcomes, National Standards, and Physical Education Curricular Models: A Systematic Review | Pfledderer et al, 2021. | Ineligible population |
| Effectiveness of school-based physical activity and sedentary time interventions in the prevention of childhood obesity for children from the age of 6 to 12 years: a systematic review and meta-analysis | Podnar et al, 2021. | Ineligible population |
| Comparative effectiveness of school-based interventions targeting physical activity, physical fitness or sedentary behaviour on obesity prevention in 6- to 12-year-old children: A systematic review and meta-analysis | Podnar et al, 2021. | Ineligible population |

| Title | Authors (Year) | Decision/Reason for exclusion |
|---|---|---|
| The effects of active video games on patients' rehabilitative outcomes: A meta-analysis | Pope et al, 2017. | Ineligible population |
| Effects of Exercise Training on Resting Testosterone Concentrations in Insufficiently Active Men: A Systematic Review and Meta-Analysis | Potter et al, 2021. | Ineligible population |
| School-Based Exercise Programs and Cardiometabolic Risk Factors: A Meta-analysis | Pozuelo-Carrascosa et al, 2018. | Ineligible population |
| Effectiveness of school-based physical activity programmes on cardiorespiratory fitness in children: a meta-analysis of randomised controlled trials | Pozuelo-Carrascosa et al, 2018. | Ineligible population |
| Effects of Aquatic Exercise on Muscle Strength in Young and Elderly Adults: A Systematic Review and Meta-Analysis of Randomized Trials. | Prado et al, 2022. | Ineligible population |

| Title | Authors (Year) | Decision/Reason for exclusion |
|---|---|---|
| A Systematic Review of Obesity Disparities Research | Pratt et al, 2017. | Ineligible population |
| Role of video games in improving health-related outcomes: a systematic review | Primack et al, 2012. | Ineligible population |
| Efficacy of physical activity interventions on psychological outcomes in refugee, asylum seeker and migrant populations: A systematic review and meta-analysis. | Purgato et al, 2021. | Ineligible population |
| Is School Gardening Combined with Physical Activity Intervention Effective for Improving Childhood Obesity? A Systematic Review and Meta-Analysis | Qi et al, 2021. | Ineligible population |
| Effects of interrupting prolonged sitting on postprandial glycemia and | Quan et al, 2021. | Ineligible population |

| Title | Authors (Year) | Decision/Reason for exclusion |
|---|---|---|
| insulin responses: A network meta-analysis. | | |
| Impact of mobile apps to combat obesity in children and adolescents: A systematic literature review | Quelly et al, 2016. | Ineligible population |
| Interventions for preventing ankle ligament injuries | Quinn et al, 2001. | Ineligible population |
| Treatment of clinical insulin resistance in children: a systematic review | Quinn et al, 2010. | Ineligible population |
| Physical activity interventions in children and young people with Type 1 diabetes mellitus: a systematic review with meta-analysis | Quirk et al, 2014. | Ineligible population |
| School Physical Education: The Effectiveness of Health-Related Interventions and | Quitério et al, 2013. | Ineligible population |

| Title | Authors (Year) | Decision/Reason for exclusion |
|---|---|---|
| Recommendations for Health-Promotion Practice | | |
| Toll like receptor expression induced by exercise in obesity and metabolic syndrome: A systematic review. | Rada et al, 2018. | Ineligible population |
| A systematic review of school-based physical activity interventions on children's wellbeing | Rafferty et al, 2016. | Ineligible population |
| Clinical interventions in overweight and obesity: a systematic literature review 2009-2014 | Rajmil et al, 2017. | Ineligible population |
| Strength and conditioning practices for the optimisation of speed and accuracy in cricket fast bowlers: A systematic review and meta-analysis. | Ramachandran et al, 2022. | Ineligible population |

| Title | Authors (Year) | Decision/Reason for exclusion |
|---|---|---|
| Effects of exercise training on blood rheology: a meta-analysis | Ramain et al, 2011. | Ineligible population |
| Effects of Plyometric Jump Training on Balance Performance in Healthy Participants: A Systematic Review With Meta-Analysis | Ramanchandran et al, 2021. | Ineligible population |
| Prevention of Anterior Cruciate Ligament Rupture in Female Athletes: A Systematic Review | Ramirez et al, 2014. | Ineligible population |
| Effects of Plyometric Jump Training on Vertical Jump Height of Volleyball Players: A Systematic Review with Meta-Analysis of Randomized-Controlled Trial. | Ramirez-Campillo et al, 2020. | Ineligible population |
| Effects of Plyometric Jump Training on Jump and Sprint Performance in Young Male Soccer Players: A Systematic Review and Meta-analysis. | Ramirez-Campillo et al, 2020. | Ineligible population |
| Effects of Plyometric Jump Training on Electromyographic Activity and Its Relationship to Strength and Jump Performance in Healthy Trained and Untrained Populations: A Systematic Review of Randomized Controlled Trials | Ramirez-Campillo et al, 2021. | Ineligible population |
| Effects of jump training on physical fitness and athletic performance in endurance runners: A meta-analysis: Jump training in endurance runners. | Ramirez-Campillo et al, 2021. | Ineligible population |
| Effects of Plyometric Jump Training on Repeated Sprint Ability in Athletes: A Systematic Review and Meta-Analysis. | Ramirez-Campillo et al, 2021. | Ineligible population |
| The effects of plyometric jump training on physical fitness attributes in basketball players: A meta-analysis. | Ramirez-Campillo et al, 2022. | Ineligible population |

| Title | Authors (Year) | Decision/Reason for exclusion |
|---|---|---|
| Effects of flywheel training on strength-related variables in female populations. A systematic review. | Raya-Gonzàlez et al, 2022. | Ineligible population |
| Young People and Physical Activity: A Systematic Review Matching Their Views to Effective Interventions | Rees et al, 2006. | Ineligible population |
| Influence of resistance training load on measures of skeletal muscle hypertrophy and improvements in maximal strength and neuromuscular task performance: A systematic review and meta-analysis. | Refalo et al, 2021. | Ineligible population |
| Effects of Lifestyle Modification Interventions to Prevent and Manage Child and Adolescent Obesity: A Systematic Review and Meta-Analysis | Rehana et al, 2020. | Ineligible population |

| Title | Authors (Year) | Decision/Reason for exclusion |
|---|---|---|
| Physical activity interventions in early life aimed at reducing later risk of obesity and related non‐communicable diseases: A rapid review of systematic reviews | Reilly et al, 2019. | Ineligible population |
| Effects of Exercise on the Resting Heart Rate: A Systematic Review and Meta-Analysis of Interventional Studies | Reimers et al, 2018. | Ineligible population |
| RelaciÃ³n entre actividad fÃsica, procesos cognitivos y rendimiento acadÃ©mico de escolares: revisiÃ³n de la literatura actual. / Relation of physical activity, cognitive and academic performance in children: Review of current literature | Reloba et al, 2016. | Ineligible population |
| Do text messages about health and development in young | Richardson et al, 2021. | Ineligible population |

| Title | Authors (Year) | Decision/Reason for exclusion |
|---|---|---|
| children affect caregiver behaviour and child outcomes? A systematic review4 | | |
| Effects of Physical Activity Intervention in Youth: A Review | Ringuet et al, 1998. | Ineligible population |
| Effectiveness of pre-school- and school-based interventions to impact weight-related behaviours in African American children and youth: a literature review | Robinson et al, 2014. | Ineligible population |
| Revisión Sistemática Sobre Programas Para La Prevención De Lesiones De La Extremidad Inferior En Jóvenes Deportistas. / A Systematic Review About Lower Extremity Injuries Prevention Programs For Young Athletes. | Robles-Palazón et al, 2009. | Ineligible population |

| Title | Authors (Year) | Decision/Reason for exclusion |
|---|---|---|
| Role of Physical Activity and Sedentary Behavior in the Mental Health of Preschoolers, Children and Adolescents: A Systematic Review and Meta‐Analysis. | Rodriguez-Ayllon et al, 2019. | Ineligible population |
| Intervention strategies for enhancing movement competencies in youth athletes: A narrative systematic review. | Rogers et al, 2020. | Ineligible population |
| A Systematic Review of Digital Interventions for Improving the Diet and Physical Activity Behaviors of Adolescents | Rose et al, 2017. | Ineligible population |
| Programming Interval Training to Optimize Time‐Trial Performance: A Systematic Review and Meta‐Analysis | Rosenblat et al, 2021. | Ineligible population |
| Exercise-based injury prevention in child and | Rossler et al, 2014. | Ineligible population |

| Title | Authors (Year) | Decision/Reason for exclusion |
|---|---|---|
| adolescent sport: a systematic review and meta-analysis | | |
| Interventions targeting working memory in 4–11 year olds within their everyday contexts: A systematic review | Rowe et al, 2019. | Ineligible population |
| Systematic Review and Meta-Analysis of Multi-Component Interventions Through Schools to Increase Physical Activity | Russ et al, 2015. | Ineligible population |
| Multidisciplinary Neuromuscular and Endurance Interventions on Youth Basketball Players: A Systematic Review with Meta-Analysis and Meta-Regression | Sacot et al, 2022. | Ineligible population |
| The association of resistance training with mortality: A | Saeidfard et al, 2019. | Ineligible population |

| Title | Authors (Year) | Decision/Reason for exclusion |
|---|---|---|
| systematic review and meta-analysis] | | |
| The Efects of Trunk Muscle Training on Physical Fitness and Sport‑Specifc Performance in Young and Adult Athletes: A Systematic Review and Meta‑Analysis | Saeterbakken et al, 2022. | Ineligible population |
| Effects of strategies to promote children's physical activity on potential mediators | Salmon et al, 2009. | Ineligible population |
| Educational and behavioral interventions in childhood obesity: A systematic review with metanalysis of randomized clinical trials | Sbruzzi et al, 2013. | Ineligible population |
| Muscular adaptations in low- versus high-load resistance training: A meta-analysis | Schoenfeld et al, 2016. | Ineligible population |

| Title | Authors (Year) | Decision/Reason for exclusion |
|---|---|---|
| Considering sex/gender in interventions to promote children's and adolescents' leisure-time physical activity: a systematic review and meta-analysis | Schulze et al, 2022. | Ineligible population |
| Childhood Obesity Evidence Base Project: A Systematic Review and Meta-Analysis of a New Taxonomy of Intervention Components to Improve Weight Status in Children 2–5 Years of Age, 2005–2019 | Scott-Sheldon et al, 2020. | Ineligible population |
| A systematic review of interventions targeting children and young peopleâ€™s physical activity behaviour in the home | Seims et al, 2023. | Ineligible population |
| Treatment of Obesity in Young People—a Systematic Review and Meta-analysis | Selvendran et al, 2018. | Ineligible population |

| Title | Authors (Year) | Decision/Reason for exclusion |
|---|---|---|
| Children's Physical Activity, Academic Performance, and Cognitive Functioning: A Systematic Review and Meta-Analysis | Sember et al, 2020. | Ineligible population |
| A Meta-Analysis of Obesity Interventions Among U.S. Minority Children | Seo et al, 2010. | Ineligible population |
| Study quality on groin injury management remains low: a systematic review on treatment of groin pain in athletes | Serner et al, 2015. | Ineligible population |
| Yoga in the schools: a systematic review of the literature | Serwacki et al, 2012. | Ineligible population |
| Environmental interventions to promote healthier eating and physical activity behaviours in institutions: a systematic review | Shaw et al, 2019. | Ineligible population |

| Title | Authors (Year) | Decision/Reason for exclusion |
| --- | --- | --- |
| A systematic review of home-based childhood obesity prevention studies | Showell et al, 2013, | Ineligible population |
| Pediatric Residency Obesity and Overweight Training Curricula: A Systematic Review | Silber et al, 2020. | Ineligible population |
| Can respiratory muscle training change the blood pressure levels in hypertension? A systematic review with meta‑analysis. | Silva et al, 2021. | Ineligible population |
| Effects of physical activity interventions on cognitive and academic performance in children and adolescents: a novel combination of a systematic review and recommendations from an expert panel | Singh et al, 2019. | Ineligible population |
| Effectiveness of school-based interventions to prevent obesity among children aged 4 to 12 years old in middle-income countries: A systematic review and meta-analysis | Singhal et al, 2021. | Ineligible population |
| A systematic review on workplace interventions to manage chronic musculoskeletal conditions. | Skamagki et al, 2018. | Ineligible population |
| The prospective association between objectively measured sedentary time, moderate-to-vigorous physical activity and cardiometabolic risk factors in youth: a systematic review and meta-analysis | Skrede et al, 2019. | Ineligible population |
| A meta-analysis to determine strength training related dose-response relationships for lower-limb muscle power development in young athletes | Slimani et al, 2018. | Ineligible population |

| Title | Authors (Year) | Decision/Reason for exclusion |
|---|---|---|
| Direct and Indirect Influence of Physical Education-Based Interventions on Physical Activity: A Review | Slingerland et al, 2011. | Ineligible population |
| The long-term effects of primary school-based obesity prevention interventions in children: A systematic review and meta-analysis | Smit et al, 2023. | Ineligible population |
| Health information technology in screening and treatment of child obesity: a systematic review | Smith et al, 2013. | Ineligible population |
| A systematic review of lifestyle interventions for chronic diseases in rural communities | Smith et al, 2016. | Ineligible population |
| Systematic literature review of built environment effects on physical activity and active transport - an update and new findings on health equity | Smith et al, 2017. . | Ineligible population |

| Title | Authors (Year) | Decision/Reason for exclusion |
|---|---|---|
| Is Cumulative Load Associated with Injuries in Youth Team Sport? A Systematic Review | Sniffen et al, 2022. | Ineligible population |
| School-based obesity prevention programs: a meta-analysis of randomized controlled trials | Sobol-Goldberg et al, 2013. | Ineligible population |
| The Efficacy of Injury Prevention Programs in Adolescent Team Sports: A Meta-analysis | Soomro et al, 2016. | Ineligible population |
| Golf and Physical Health: A Systematic Review | Sorbie et al, 2022. | Ineligible population |
| Physical activity and healthy eating in Brazilian students: a review of intervention programs | Souza et al, 2011. | Ineligible population |
| Does Exercise Influence Pediatric Bone? A Systematic Review | Specker et al, 2015. | Ineligible population |

| **Title** | **Authors (Year)** | **Decision/Reason for exclusion** |
|---|---|---|
| The effects of resistance training interventions on vertical jump performance in basketball players: a meta-analysis | Sperlich et al, 2016. | Ineligible population |
| Interventions for preventing obesity in children | Spinola et al, 2014. | Ineligible population |
| The effects of physical activity interventions on psychosocial outcomes in adolescents: A meta-analytic review | Spruit et al, 2016. | Ineligible population |
| A Systematic Review of the Relationships Between Physical Activity and Sleep in Early Childhood | St Laurent et al 2022. | Ineligible population |
| Dose-Response Relationship of Neuromuscular Training for Injury Prevention in Youth Athletes: A Meta-Analysis | Steib et al, 2017. | Ineligible population |

| **Title** | **Authors (Year)** | **Decision/Reason for exclusion** |
|---|---|---|
| A Meta-Analytic Review of Obesity Prevention Programs for Children and Adolescents: The Skinny on Interventions that Work | Stice et al, 2006. | Ineligible population |
| Are work organization interventions effective in preventing or reducing work-related musculoskeletal disorders? A systematic review of the literature. | Stock et al, 2018. | Ineligible population |
| Evidence based physical activity for school-age youth | Strong et al, 2005. | Ineligible population |
| Evaluation of the effectiveness of neuromuscular training to reduce anterior cruciate ligament injury in female athletes: a critical review of relative risk reduction and numbers-needed-to-treat analyses | Sugimoto et al, 2012. | Ineligible population |

| **Title** | **Authors (Year)** | **Decision/Reason for exclusion** |
|---|---|---|
| Specific exercise effects of preventive neuromuscular training intervention on anterior cruciate ligament injury risk reduction in young females: meta-analysis and subgroup analysis | Sugimoto et al, 2015. | Ineligible population |
| Motivational Interviewing for Weight Management Among Women: a Meta-Analysis and Systematic Review of RCTs | Suire et al, 2021. | Ineligible population |
| Interventions for treating obesity in children | Summerbell et al, 2003. | Ineligible population |
| Effects of school-based interventions for direct delivery of physical activity on fitness and cardiometabolic markers in children and adolescents: a systematic review of randomized controlled trials | Sun et al, 2013. | Ineligible population |
| Effectiveness of early care and education center-based interventions for improving cardiovascular fitness in early childhood: A systematic review and meta-analysis | Szeszulskia et al, 2019. | Ineligible population |
| Family-Centered Interventions for Treatment and Prevention of Childhood Obesity in | Tamayo et al, 2021. | Ineligible population |
| Influence of physical activity on bone strength in children and adolescents: a systematic review and narrative synthesis | Tan et al, 2014. | Ineligible population |
| Deoxygenation of inspiratory muscles during cycling, hyperpnoea and loaded breathing in health and disease: a systematic review. | Tanaka et al, 2018. | Ineligible population |
| A systematic review of interventions to promote | Temple et al, 2014. | Ineligible population |

| Title | Authors (Year) | Decision/Reason for exclusion |
|---|---|---|
| physical activity in the preschool setting | | |
| Exercise Timing in Type 2 Diabetes Mellitus: A Systematic Review. | Teo et al, 2018. | Ineligible population |
| Effect of Different Exercise Modalities on Oxidative Stress: A Systematic Review | Thirupathi et al, 2021. | Ineligible population |
| Pedagogical Approaches to and Effects of Fundamental Movement Skill Interventions on Health Outcomes: A Systematic Review | Tompsett et al, 2017. | Ineligible population |
| Converging Evidence Supporting the Cognitive Link between Exercise and Esport Performance: A Dual Systematic Review | Toth et al, 2020. | Ineligible population |
| A Systematic Review of Obesity Prevention Intervention | Tovar et al, 2014. | Ineligible population |

| Title | Authors (Year) | Decision/Reason for exclusion |
|---|---|---|
| Studies among Immigrant Populations in the US | | |
| Effect of Strength Training on Biomechanical and Neuromuscular Variables in Distance Runners: A Systematic Review and Meta-Analysis. | Trowell et al, 2020. | Ineligible population |
| What is the impact of structural and cultural factors and interventions within educational settings on promoting positive mental health and preventing poor mental health: a systematic review | Troy et al, 2022. | Ineligible population |
| Treatment of adolescent overweight and obesity | Tsiros et al, 2008. | Ineligible population |
| Prevention and treatment of pediatric obesity using mobile and wireless technologies: a systematic review | Turner et al, 2015. | Ineligible population |

| Title | Authors (Year) | Decision/Reason for exclusion |
|---|---|---|
| Exercise interventions for smoking cessation | Ussher et al, 2019. | Ineligible population |
| The Relationship Between Motor Competence and Physical Fitness from Early Childhood to Early Adulthood: A Meta‐Analysis | Utesch et al, 2019. | Ineligible population |
| Effects of tapering on neuromuscular and metabolic fitness in team sports: a systematic review and meta-analysis. | Vachon et al, 2021. | Ineligible population |
| Systematic review of interventions in the childcare setting with direct parental involvement: effectiveness on child weight status and energy balance-related behaviours | van de Kolk et al, 2019. | Ineligible population |
| Maintenance interventions for overweight or obesity in | van der Heijden et al, 2018. | Ineligible population |

| Title | Authors (Year) | Decision/Reason for exclusion |
|---|---|---|
| children: a systematic review and meta-analysis | | |
| Effective interventions in overweight or obese young children: systematic review and meta-analysis | van Hoek et al, 2014. | Ineligible population |
| Effectiveness of interventions to promote physical activity in children and adolescents: systematic review of controlled trials | van Sluijs et al, 2007. | Ineligible population |
| School‐based Physical Activity Interventions in Children and Adolescents: A Systematic Review | Vaquero-Solis et al, 2020. | Ineligible population |
| Effects of intervention programs on child and adolescent BMI: A meta-analysis study | Vasques et al, 2014. | Ineligible population |

| Title | Authors (Year) | Decision/Reason for exclusion |
|---|---|---|
| Entrenamiento de fuerza con bandas elásticas en niños y adolescentes: una revisión sistemática. / Resistance training using elastic band in children and adolescents. A systematic review. | Vázquez et al, 2022. | Ineligible population |
| Physical activity and prospective associations with indicators of health and development in children aged <5 years: a systematic review | Veldman et al 2021. | Ineligible population |
| Physical exercise and executive functions in preadolescent children, adolescents and young adults: a meta-analysis | Verburgh et al, 2014. | Ineligible population |
| Effectiveness of preventive school-based obesity interventions in low- and middle-income countries: a systematic review | Verstraeten et al, 2012. | Ineligible population |

| Title | Authors (Year) | Decision/Reason for exclusion |
|---|---|---|
| Resistance Training in Youth Improves Athletic Performance: A Systematic Review | Vesci et al, 2017. | Ineligible population |
| Skeletal muscle functional and structural adaptations after eccentric overload flywheel resistance training: a systematic review and meta-analysis. | Vicens-Borbas et al, 2017. | Ineligible population |
| Is inertial flywheel resistance training superior to gravity-dependent resistance training in improving muscle strength? A systematic review with meta-analyses. | Vicens-Borbas et al, 2018. | Ineligible population |
| Effects of Resistance Training Performed to Failure or Not to Failure on Muscle Strength, Hypertrophy, and Power Output: A Systematic Review With Meta-Analysis. | Vieira et al, 2021. | Ineligible population |

| Title | Authors (Year) | Decision/Reason for exclusion |
|---|---|---|
| Efficacy of school-based interventions for improving muscular fitness outcomes in children: A systematic review and meta-analysis | Vila-González et al, 2023. | Ineligible population |
| Systematic review of interventions for promoting active school transport | Villa-Gonzalez et al, 2018. | Ineligible population |
| Does Physical Activity Improve Cognition and Academic Performance in Children? A Systematic Review of Randomized Controlled Trials | Vorkapic et al, 2021. | Ineligible population |
| Promoting cycling: a review of interventions | Vuori et al, 2011. | Ineligible population |
| A systematic review of cognitive assessment in physical activity research involving children and adolescents. | Wade et al, 2020. | Ineligible population |
| Effects of Heat Acclimation and Acclimatisation on Maximal Aerobic Capacity Compared to Exercise Alone in Both Thermoneutral and Hot Environments: A Meta-Analysis and Meta-Regression. | Waldron et al, 2021. | Ineligible population |
| A Systematic Review of Rural, Theory-based Physical Activity Interventions | Walsh et al, 2017. | Ineligible population |
| What childhood obesity prevention programmes work? A systematic review and meta-analysis | Wang et al, 2015. | Ineligible population |
| Effects and dose–response relationship of high-intensity interval training on cardiorespiratory fitness in overweight and obese adults: a systematic review and meta-analysis. | Wang et al, 2021. | Ineligible population |

| Title | Authors (Year) | Decision/Reason for exclusion |
|---|---|---|
| The Effectiveness of Wearable Devices as Physical Activity Interventions for Preventing and Treating Obesity in Children and Adolescents: Systematic Review and Meta-analysis | Wang et al, 2022. | Ineligible population |
| A systematic review and meta-analysis of exercise interventions for youth mental health | Wang et al, 2022. | Ineligible population |
| Systematic review and meta-analysis of the efects of exercise on depression in adolescents | Wang et al, 2022. | Ineligible population |
| Strength of obesity prevention interventions in early care and education settings: A systematic review | Ward et al, 2017. | Ineligible population |
| Interventions for preventing obesity in children | Waters et al, 2014. | Ineligible population |

| Title | Authors (Year) | Decision/Reason for exclusion |
|---|---|---|
| Effect of classroom-based physical activity interventions on academic and physical activity outcomes: a systematic review and meta-analysis | Watson et al, 2017. | Ineligible population |
| The Effect of Resistance Training in Healthy Adults on Body Fat Percentage, Fat Mass and Visceral Fat: A Systematic Review and Meta-Analysis. | Wewege et al, 2022. | Ineligible population |
| Systematic review of interventions to increase physical activity and physical fitness in African-Americans | Whitt-Glover et al, 2019. | Ineligible population |
| School-based Internet obesity prevention programs for adolescents: A systematic literature review | Whittemore et al, 2013. | Ineligible population |
| Effectiveness of interventions to improve lifestyle behaviors | Wijtzes et al, 2017. | Ineligible population |

| Title | Authors (Year) | Decision/Reason for exclusion |
|---|---|---|
| among socially disadvantaged children in Europe | | |
| The Effects of Exercise Dosage on Neck-Related Pain and Disability: A Systematic Review With Meta-analysis | Wilhelm et al, 2020. | Ineligible population |
| Chronic effects of high‑intensity functional training on motor function: a systematic review with multilevel meta‑analysis | Wilke et al, 2020. | Ineligible population |
| Neuromuscular Training and Motor Control in Youth Athletes: A Meta-Analysis | Williams et al, 2021. | Ineligible population |
| Effectiveness Of Obesity Intervention Programs Based On Guidelines For Adolescent Students: Systematic Review | Wolf et al, 2019. | Ineligible population |
| A systematic review and meta-analysis of whole of | Wolfenden et al, 2014. | Ineligible population |

| Title | Authors (Year) | Decision/Reason for exclusion |
|---|---|---|
| community interventions to prevent excessive population weight gain | | |
| Strategies for enhancing the implementation of school-based policies or practices targeting risk factors for chronic disease(Review) | Wolfenden et al, 2017. | Ineligible population |
| Strategies to improve the implementation of healthy eating, physical activity and obesity prevention policies, practices or programmes within childcare services (Review) | Wolfenden et al, 2020. | Ineligible population |
| Interventions for increasing the moderate-vigorous intensity physical activity (MVPA) content of primary school physical education | Wong et al, 2021. | Ineligible population |
| Interventions to Increase Moderate-to-Vigorous Physical | Wong et al, 2021. | Ineligible population |

| Title | Authors (Year) | Decision/Reason for exclusion |
| --- | --- | --- |
| Activity in Elementary School Physical Education Lessons: Systematic Review | | |
| Interventions to Increase Moderate-to-Vigorous Physical Activity in Elementary School Physical Education Lessons: Systematic Review | Wong et al, 2021. | Ineligible population |
| Effects of school-based before-school physical activity programmes on children's | Woodforde et al, 2022. | Ineligible population |
| Blood Flow Restriction Training for Athletes: A Systematic Review. | Wortman et al, 2021. | Ineligible population |
| Effectiveness of shoulder injury prevention programs in an overhead athletic population: A systematic review | Wright et al, 2021. | Ineligible population |

| Title | Authors (Year) | Decision/Reason for exclusion |
| --- | --- | --- |
| The Effect of Intensity, Frequency, Duration and Volume of Physical Activity in Children and Adolescents on Skeletal Muscle Fitness: A Systematic Review and Meta-Analysis of Randomized Controlled Trials | Wu et al, 2021. | Ineligible population |
| Effect of Exercise Training on Physical Fitness Among Young Tennis Players: A Systematic Review | Xiao et al, 2022. | Ineligible population |
| Effects of chronic exercise interventions on executive function among children and adolescents: a systematic review with meta-analysis | Xue et al, 2019. | Ineligible population |
| The impact of isometric handgrip exercise and training on health‐related factors: A review | Yamada et al, 2022. | Ineligible population |

| Title | Authors (Year) | Decision/Reason for exclusion |
|---|---|---|
| Combined effects of physical activity and calcium on bone health in children and adolescents: a systematic review of randomized controlled trials | Yang et al, 2020. | Ineligible population |
| Family sports interventions for the treatment of obesity in childhood: a meta-analysis | Yang et al, 2022. | Ineligible population |
| Beneficial impact of exercise on bone mass in individuals under calorie restriction: a systematic review and Meta-analysis of randomized clinical trials. | Yarizadeh et al, 2021. | Ineligible population |
| Effect of Mobile Health Technology on Weight Control in Adolescents and Preteens: A Systematic Review and Meta-Analysis | Yien et al, 2021. | Ineligible population |
| A systematic review of interventions to improve the | Yoong et al, 2020. | Ineligible population |

| Title | Authors (Year) | Decision/Reason for exclusion |
|---|---|---|
| dietary intake, physical activity and weight status of children attending family day care services | | |
| The influence of dosing on effect size of exercise therapy for musculoskeletal foot and ankle disorders: a systematic review. | Young et al, 2018. | Ineligible population |
| Balance training for neuromuscular control and performance enhancement: a systematic review | Zech et al, 2010. | Ineligible population |
| Exergaming and obesity in youth: current perspectives | Zeng et al, 2016. | Ineligible population |
| Effects of Physical Activity on Motor Skills and Cognitive Development in Early Childhood: A Systematic Review | Zeng et al, 2017. | Ineligible population |

| Title | Authors (Year) | Decision/Reason for exclusion |
|---|---|---|
| Effect of sedentary behavior interventions on vascular function in adults: A systematic review and meta‑analysis. | Zheng et al, 2021. | Ineligible population |
| Does the gut microbiota contribute to the antiobesity effect of exercise? A systematic review and meta-analysis. | Zheng et al, 2022. | Ineligible population |
| Childhood obesity prevention interventions in childcare settings: systematic review of randomized and nonrandomized controlled trials | Zhou et al, 2014. | Ineligible population |
| Effects of different physical activities on brain-derived neurotrophic factor: A systematic review and bayesian network meta-analysis | Zhou et al, 2022. | Ineligible population |
| Characteristics of physical activity interventions and effects on cardiorespiratory | Braaksma et al, 2017. | Ineligible intervention |

| Title | Authors (Year) | Decision/Reason for exclusion |
|---|---|---|
| fitness in children aged 6-12 years-A systematic review. | | |
| The Impact of Schoolyard Greening on Children's Physical Activity and Socioemotional Health: A Systematic Review of Experimental Studies | Bikomeye et al, 2021 | Ineligible intervention |
| Preventing and treating childhood overweight and obesity in children up to 5 years old: A systematic review by intervention setting | Flynn et al, 2022. | Ineligible intervention |
| Screening for Hypertension in Children and Adolescents: Systematic Review for the U.S. Preventive Services Task Force | Gartlehner et al, 2020. | Ineligible intervention |
| A meta-analysis of motivational interviewing interventions for pediatric health behavior change | Gayes et al, 2014. | Ineligible intervention |

| Title | Authors (Year) | Decision/Reason for exclusion |
|---|---|---|
| Research gaps in physical activity, sedentary behavior, and obesity among children in Qatar: A systematic review | Hammadi et al, 2022. | Ineligible intervention |
| Meta-analysis of lifestyle modification interventions addressing overweight and | Janicke et al, 2021. | Ineligible intervention |
| Street connectivity, physical activity, and childhood obesity: A systematic review and meta‑analysis | Jia et al, 2019. | Ineligible intervention |
| Identifying efective intervention strategies to reduce children's screen time: a systematic review and meta-analysis | Jones et al, 2021. | Ineligible intervention |
| Physical Activity Interventions Among American Indian and Alaska Native Persons: A | Kokenge et al, 2020. | Ineligible intervention |

| Title | Authors (Year) | Decision/Reason for exclusion |
|---|---|---|
| Classroom Standing Desks and Sedentary Behavior: A Systematic Review | Minges et al, 2026. | Ineligible intervention |
| Digital Interventions to Improve Health Literacy Among Parents of Children Aged 0 to 12 Years With a Health Condition: Systematic Review | Mörelius et al, 2021. | Ineligible intervention |
| Very-low-energy diets and morbidity: a systematic review of longer-term evidence | Mulholland et al, 2012. | Ineligible intervention |
| School‑based education programmes for the prevention of unintentional injuries in children and young people | Orton et al, 2016. | Ineligible intervention |
| Components of primary care interventions to treat childhood overweight and obesity: a systematic review of effect | Sargent et al, 2011. | Ineligible intervention |

| Title | Authors (Year) | Decision/Reason for exclusion |
|---|---|---|
| Effectiveness of intervention strategies exclusively targeting reductions in children's sedentary time: a systematic review of the literature | Saunders et al, 2016. | Ineligible intervention |
| The Physiological and Psychological Benefits of Dance and its Effects on Children and Adolescents: A Systematic Review | Tao et al, 2022. | Ineligible intervention |
| Primary care prevention of cardiovascular risk behaviors in adolescents: A systematic review | Tissot et al, 2021. | Ineligible intervention |
| Motivational interviewing for treating overweight and obese youth: A systematic review | Vallabhan et al, 2023 | Ineligible intervention |
| Effects of high-intensity interval training on executive functions in children and adolescents: A Systematic Review and Meta-analysis | Wang et al, 2023. | Ineligible intervention |
| The effectiveness of nurse-led interventions to prevent childhood and adolescent overweight and obesity: A systematic review of randomised trials | Whitehead et al, 2021. | Ineligible intervention |
| Efficacy of Clinic-Based Telehealth vs. Face-to-Face Interventions for Obesity Treatment in Children and Adolescents in the United States and Canada: A Systematic Review | Whitley et al, 2021. | Ineligible intervention |
| Systematic review and meta-analysis of interventions targeting sleep and their impact on child body mass index, diet, and physical activity | Yoong et al, 2016. | Ineligible intervention |

| Title | Authors (Year) | Decision/Reason for exclusion |
|---|---|---|
| Family-focused physical activity, diet and obesity interventions in African-American girls: a systematic review | Barr-Anderson et al, 2013. | Ineligible study duration |
| Family-based interventions to increase physical activity in children: a systematic review, meta-analysis and realist synthesis | Brown et al, 2013. | Ineligible study duration |
| A Meta-Analysis of High-Intensity Interval Training on Glycolipid Metabolism in Children With Metabolic Disorders | Cao et al, 2022. | Ineligible study duration |
| Can active video games be part of the solution to promote physical activity in youth? A systematic review | Carmo et al, 2012. | Ineligible study duration |
| Physical activity and exercise interventions for childhood | Cesa et al, 2022. | Ineligible study duration |

| Title | Authors (Year) | Decision/Reason for exclusion |
|---|---|---|
| obesity: A systematic review with meta-analysis of randomized clinical trials | | |
| A Meta-Analytic Review of Obesity Prevention in the Schools: 1997-2008 | Cook-Cottone et al, 2009. | Ineligible study duration |
| Systematic review of acute physically active learning and classroom movement breaks on children's physical activity, cognition, academic performance and classroom behaviour: understanding critical design features | Daly-Smith et al, 2018. | Ineligible study duration |
| High-Intensity Interval Training Interventions in Children and Adolescents: A Systematic Review | Eddolls et al, 2017. | Ineligible study duration |
| Best practice dietetic management of overweight and obese children and adolescents: | Ho et al, 2013. | Ineligible study duration |

| Title | Authors (Year) | Decision/Reason for exclusion |
|---|---|---|
| A 2010 update of a systematic review | | |
| The effectiveness and promising strategies of obesity prevention and treatment programmes among adolescents from disadvantaged backgrounds: a systematic review | Kornet-van der Aa et al, 2017. | Ineligible study duration |
| School-related sedentary behaviours and indicators of health and well-being among children and youth: a systematic review | Kuzik et al, 2022. | Ineligible study duration |
| Factors that alter body fat, body mass, and fat-free mass in pediatric obesity | LeMura et al, 2002. | Ineligible study duration |
| Face-to-face physical activity incorporated into dietary intervention for overweight/obesity in children | Liang et al, 2022. | Ineligible study duration |

| Title | Authors (Year) | Decision/Reason for exclusion |
|---|---|---|
| and adolescents: a Bayesian network meta-analysis | | |
| A systematic review of studies using pedometers to promote physical activity among youth | Lubans et al, 2009. | Ineligible study duration |
| Physical Activity for Cognitive and Mental Health in Youth: A Systematic Review of Mechanisms | Lubans et al, 2016. | Ineligible study duration |
| Effect of Active Lessons on Physical Activity, Academic, and Health Outcomes: A Systematic Review | Martin et al, 2017. | Ineligible study duration |
| Differences in Physical Fitness and Body Composition Between Active and Sedentary | Mateo-Orcajada et al, 2022. | Ineligible study duration |
| Effectiveness of interventions on physical activity in overweight or obese children: a systematic review and | Nooijen et al, 2017. | Ineligible study duration |

| Title | Authors (Year) | Decision/Reason for exclusion |
|---|---|---|
| meta-analysis including studies with objectively measured outcomes | | |
| A systematic review of the implementation of obesity prevention interventions in early childcare and education settings using the RE-AIM framework | Sanchez-Flack et al, 2020. | Ineligible study duration |
| High-intensity interval training in overweight and obese children and adolescents: systematic review and meta-analysis | Thivel et al, 2019. | Ineligible study duration |
| What works in school-based energy balance behaviour interventions and what does not? A systematic review of mediating mechanisms | van Stralen et al, 2011. | Ineligible study duration |
| A Sex/Gender Perspective on Interventions to Reduce | Vondung et al, 2020. | Ineligible study duration |

| Title | Authors (Year) | Decision/Reason for exclusion |
|---|---|---|
| Sedentary Behaviour in Girls and Boys: Results of the genEffects Systematic Review | | |
| School-based physical activity interventions in rural and urban/suburban communities: A systematic review and meta-analysis | Pfledderer et al, 2021. | Ineligible outcome |
| Do behaviour change techniques promote physical activity in preschool children? A systematic review | Al-walah et al, 2023. | Ineligible outcome |
| Effects of physical activity interventions on cognitive performance of overweight or | Barceló et al, 2021. | Ineligible outcome |
| The effects of the Nordic hamstring exercise on sprint performance and eccentric knee flexor strength: A systematic review and meta-analysis of | Bautista et al, 2022 | Ineligible outcome |

| Title | Authors (Year) | Decision/Reason for exclusion |
|---|---|---|
| intervention studies among team sport players. | | |
| Physical activity interventions targeting perceived body image among adolescent girls | Dai et al, 2020. | Ineligible outcome |
| A Systematic Review and Meta-Analysis of the Effects of Outdoor Education Programs on Adolescents' Self-Efficacy | Fang et al, 2021. | Ineligible outcome |
| Effect of Warm-up on Fitness Performance of Schoolchildren. A Systematic Review | Fernandez-Agullo et al, 2022. | Ineligible outcome |
| Effects of physical education interventions on academic performance and cognition in children and adolescents | García-Hermoso et al, 2021 | Ineligible outcome |
| The effect of social media interventions on physical activity and dietary behaviours | Goodyear et al, 2021 | Ineligible outcome |

| Title | Authors (Year) | Decision/Reason for exclusion |
|---|---|---|
| in young people and adults: a systematic review | | |
| A systematic review of physical activity promotion in children and adolescents by community health workers | Guerra et al, 2015 | Ineligible outcome |
| Neurobiological evidence of physical activity interventions on mental health outcomes and cognition in youth: a systematic review of randomised controlled trials | Heinze et al, 2022. | Ineligible outcome |
| Social influence in childhood obesity interventions: a systematic review | Jalali et al, 2016. | Ineligible outcome |
| It's Not Just What You Do but the Way You Do It: A Systematic Review of Process Evaluation of Interventions to Improve Gross Motor Competence. | Jiani et al, 2021. | Ineligible outcome |

| Title | Authors (Year) | Decision/Reason for exclusion |
|---|---|---|
| A systematic review exploring body image programmes and interventions in physical education | Kerner et al, 2022 | Ineligible outcome |
| Effectiveness of active school transport interventions: a systematic review and update | Larouche et al, 2018. | Ineligible outcome |
| Physical Activity, Fitness, School Readiness, and Cognition in Early Childhood: A Systematic Review | Laurent et al, 2021. | Ineligible outcome |
| Mental health and socioeconomic status impact adherence to youth activity and dietary programs: a meta-analysis | Lemstra et al, 2021. | Ineligible outcome |
| Getting the fundamentals of movement: a meta-analysis of the effectiveness of motor skill interventions in children | Logan et al, 2012. | Ineligible outcome |

| Title | Authors (Year) | Decision/Reason for exclusion |
|---|---|---|
| Classroom-based physical activity and sedentary 2 behavior interventions in adolescents: a systematic review and meta-analysis. Journal of Physical 3 Activity and Health. | McMichan et al, 2018 | Ineligible outcome |
| Fundamental movement skill interventions in youth: a systematic review and meta-analysis | Morgan et al, 2013. | Ineligible outcome |
| What Is Known about School-Based Interventions for Health Promotion and Their Impact in Developing Countries? A Scoping Review of the Literature | Mukamana et al, 2016. | Ineligible outcome |
| A prática da capoeira no ambiente escolar para a formação integral do aluno: uma revisão sistemática. / The practice of capoeira in the | Ribeiro et al, 2021. | Ineligible outcome |

| Title | Authors (Year) | Decision/Reason for exclusion |
|---|---|---|
| school environment for student integral formation: A systematic review. | | |
| Effectiveness of school-based interventions promoting physical activity and reducing sedentary time among children: A meta-analysis of accelerometerâ€assessed controlled trials. | Rodrigo-Sanjoaquín et al, 2022. | Ineligible outcome |
| Exercise targeted at the LIPOXmax for overweight or obesity: A meta-analysis | Romain et al, 2012 | Ineligible outcome |
| Effect of classroom-based physical activity interventions on attention and on-task behavior in schoolchildren: A systematic review | Ruhland et al, 2021. | Ineligible outcome |
| A systematic review of school-based interventions to | Saraf et al, 2012 | Ineligible outcome |

| Title | Authors (Year) | Decision/Reason for exclusion |
|---|---|---|
| prevent risk factors associated with noncommunicable diseases | | |
| Physical activity interventions in Hispanic American girls and women | Sharma et al, 2008. | Ineligible outcome |
| Play Smart, Be Smart? Effect of Cognitively Engaging Physical Activity Interventions on Executive Function among Children 4~12 Years Old: A Systematic Review and Meta-Analysis | Song et al, 2022. | Ineligible outcome |
| Obesity prevention interventions for middle school-age children of ethnic minority: a review of the literature | Stevens et al, 2010. | Ineligible outcome |
| School-Based Physical Activity Interventions in Prevocational Adolescents: A Systematic Review and Meta-Analyses | van de Kop et al, 2019. | Ineligible outcome |

| Title | Authors (Year) | Decision/Reason for exclusion |
|---|---|---|
| Strategies for enhancing the implementation of school-based policies or practices targeting risk factors for chronic disease | Wolfenden et al, 2017. | Ineligible outcome |
| Effects of school nurse-led interventions in promoting physical activity and reducing sedentary behaviours in children and adolescents: a systematic review with meta-analysis | Yari Longobucco et al. 2023. | Ineligible outcome |
| Impact of Physical Activity-Based Interventions on Children and Adolescents' Physical Self-Concept: A Meta-Analysis | Zamorano-García et al, 2023. | Ineligible outcome |
| Responses of cardiorespiratory variables in crossover interventions of land running and deep-water running | Assunção et al., 2023 | Article not found |

| Title | Authors (Year) | Decision/Reason for exclusion |
|---|---|---|
| Does Regular Exercise Impact the Lung Function of Healthy Children and Adolescents? A Systematic Review and Meta-Analysis | Balbinot et al., 2022 | Article not found |
| Activity- and Occupation-Based Interventions to Support Mental Health, Positive Behavior, and Social Participation for Children and Youth: A Systematic Review | Cahill et al., 2020 | Article not found |
| Effects of Strength Training on Blood Pressure and Heart Rate Variability—A Systematic Review. | Corso et al., 2022 | Article not found |
| Empowerment Approaches in Childhood Weight Management: A Systematic Review | Earle et al., 2022 | Article not found |
| Treating the ticking time bomb: Childhood obesity | Ells et al., 2018 | Article not found |

| Title | Authors (Year) | Decision/Reason for exclusion |
|---|---|---|
| Treatments for childhood and adolescent obesity | Haddock et al., 1994 | Article not found |
| Impact of dietary and exercise interventions on anthropometric and metabolic outcomes in obese children and adolescents: A systematic review and metaanalysis of randomised controlled trials | Ho et al., 2013 | Article not found |
| Effects of Different Movement Programs on Motor Competence: A Systematic Review With Meta-Analysis. | Jiménez-Díaz et al., 2019 | Article not found |
| Interventions for Increasing Physical Activity in Low-Income, Ethnic Minority Children and Youths: Meta Analysis | Kim et al., 2018 | Article not found |
| Physical Exercise and Brain-Derived Neurotrophic Factor Concentration in | Menezes-Junior et al., 2022 | Article not found |

| Title | Authors (Year) | Decision/Reason for exclusion |
|---|---|---|
| Children and Adolescents: A Systematic Review With Meta-Analysis | | |
| Does exercise beneficially affect sex hormones when added to hypo-caloric diets in adults with overweight or obesity? A systematic review and meta-analysis of controlled clinical trials | Mohseni-Takalloo et al., 2022 | Article not found |
| Effect Of School-based Physical Activity Programs On Hamstring Flexibility: A Meta-analysis | Montero-Briceño et al., 2018 | Article not found |
| Effectiveness of peer-led programs for overweight and obesity in children: systematic review and meta-analysis | Nguyen et al., 2022 | Article not found |
| Assessing the cost effectiveness of public health interventions to prevent obesity: A systematic | Paech et al., 2012 | Article not found |

| Title | Authors (Year) | Decision/Reason for exclusion |
|---|---|---|
| review of the effectiveness of 16 obesity prevention interventions | | |
| Effectiveness of universal self-regulation-based interventions to improve self-regulation, and effects on distant health and social outcomes in children and adolescents: A systematic review and meta-analysis | Pandey et al., 2018 | Article not found |
| Effects of school-based interventions on body mass index in children and adolescents: A systematic overview and meta-analysis | Park et al., 2014 | Article not found |
| Effects Of Classroom Active Breaks On Academic-related Outcomes In Children: A Systematic Review And Meta-analysis | Peiris et al., 2022 | Article not found |

| Title | Authors (Year) | Decision/Reason for exclusion |
|---|---|---|
| Effective behavioural components in child and adolescent weight management programmes | Sahota et al., 2010 | Article not found |
| Adherence to mind-body therapies: A systematic review and meta-analysis | Shaw et al., 2018 | Article not found |
| The Effect Of Plyometric Training On Running Economy In Long Distance Runners | Spurrs et al., 2003 | Article not found |
| A Meta-analysis Of The Effect Of School-based Physical Activity Intervention On Students' Mvpa | Sun et al., 2021 | Article not found |
| Quantitative analysis of single- vs. multiple-set programs in resistance training | Wolfe et al., 2004 | Article not found |
| Effect of high-intensity interval training on cardiometabolic risk | Zhu et al., 2021 | Article not found |

| Title | Authors (Year) | Decision/Reason for exclusion |
|---|---|---|
| factors in childhood obesity: a meta-analysis | | |

**Table S3. Total outcomes.**

| Study (Year) | Domain | Generic outcome (as stated by the authors) | Outcome | Outcome measure |
|---|---|---|---|---|
| Aceves-Martins et al., 2022 | General | Anthropometry | Body mass index (BMI) | NR |
| Al-Khudairy et al., 2017 | Delivery of Care | Participants' views of the intervention | Participants' views of the intervention | Defined as documented accounts from participant feedback and measured at baseline and at least at six months (e.g. levels of satisfaction) |
| | Emotional Functioning/Wellbeing | Self-esteem | Self-esteem | Evaluated by a validated instrument such as Rosenberg Self-Esteem Scale and measured at baseline and at least at six months |
| | General | Anthropometric measures other than BMI | Body fat mass (% and kg) | Measured by bioelectrical impedance analysis or DXA or MRI scan |
| | | Changes in measured BMI or body weight | Body mass index (BMI) | KG/m² or BMI z score |
| | | Changes in measured BMI or body weight | Body weight | NR |
| | | Anthropometric measures other than BMI | Lean body mass | Measured by bioelectrical impedance analysis |
| | | Anthropometric measures other than BMI | Skinfold thickness | NR |
| | | Anthropometric measures other than BMI | Waist circumference | NR |
| | | Anthropometric measures other than BMI | Waist-to-hip ratio | NR |
| | Global Quality of Life | Health-related quality of life | Quality of life | Health-related quality of life: Paediatric Quality of Life Inventory |

| Study (Year) | Domain | Generic outcome (as stated by the authors) | Outcome | Outcome measure |
|---|---|---|---|---|
| | Metabolism and Nutrition | Behavior change of diet | Dietary behavior | Defined as validated measures of diet measured by either objective (e.g. Mandometer) or validated self-reported questionnaires |
| | | Behavior change of diet | Dietary intake | Defined as validated measures of diet as food records |
| | Physical Functioning | Behavior change of physical activity | Physical activity behavior | Defined as validated measures as self-reported measures or objective measures (e.g hours spent viewing television) |
| | | Behavior change of physical activity | Physical activity level | Assessed using objective tools or validated self-reported tools |
| | Social Functioning | Parenting skill and relationships | Parenting skill and relationships | Evaluated by a validated instrument and measured at baseline and at least at six months (e.g. parent-adolescent communication) |
| Albornoz-Guerrero et al., 2021 | Cardiac | | Blood Pressure | manual blood pressure cuff, automatic sphygmomanometer |
| | Emotional Functioning/Wellbeing | | Mood | the scale of intrinsic versus extrinsic orientation in the classroom |
| | | | Self-perception | self-perceptions profile questionnaire for children, the child and youth physical self-perception profile questionnaire |
| | | | Self-esteem | self-perceptions profile questionnaire for children, the child and youth physical self-perception profile questionnaire |
| | General | | Body composition | bio impedance, anthropometry, DXA, caliper, three-component model of body composition |
| | | | Body fat | bio impedance, anthropometry, DXA, caliper, three-component model of body composition |
| | | | Body mass index (BMI) | kg/m2 |
| | | | Hip circumference | bio impedance, anthropometry, DXA, caliper, three-component model of body composition |

| Study (Year) | Domain | Generic outcome (as stated by the authors) | Outcome | Outcome measure |
|---|---|---|---|---|
| | | | skinfold | bio impedance, anthropometry, DXA, caliper, three-component model of body composition |
| | | | strength and difficulties perceived by parents | the questionnaire of strengths and difficulties of the child reported by the parents |
| | | | Waist Circumference | bio impedance, anthropometry, DXA, caliper, three-component model of body composition |
| | | | waist-to-height ratio | bio impedance, anthropometry, DXA, caliper, three-component model of body composition |
| | | | weight | bio impedance, anthropometry, DXA, caliper, three-component model of body composition |
| | Global Quality of Life | | Health-related QOL | pediatric QOL questionnaire, the child health questionnaire, the QOL questionnaire for children between 7 and 13 year old |
| | Metabolism and Nutrition | | Eating behaviour | food frequency questionnaire, 3-day diet recall form, 24h-dietary recall, Block Kids Questionnaire, Dutch eating behaviour questionnaire for children, The Family Eating and Activity Habits questionnaire, Day in life questionnaire, 2-day nutritional dietary record. |
| | | | Food intake | food frequency questionnaire, 3-day diet recall form, 24h-dietary recall, Block Kids Questionnaire, Dutch eating behaviour questionnaire for children, The Family Eating and Activity Habits questionnaire, Day in life questionnaire, 2-day nutritional dietary record. |
| | Physical Functioning | | Aerobic fitness | accelerometry, pedometer, Physical Activity Questionnaire for Children, Leisure Score Index, walking test, handgrip strength test, ergospirometry test, jumping test |

| Study (Year) | Domain | Generic outcome (as stated by the authors) | Outcome | Outcome measure |
|---|---|---|---|---|
| | | | Muscle strength | bio impedance, anthropometry, DXA, caliper, three-component model of body composition |
| | | | physical activity level | accelerometry, pedometer, Physical Activity Questionnaire for Children, Leisure Score Index, walking test, handgrip strength test, ergospirometry test, jumping test |
| | | | sedentary behaviour | accelerometry, pedometer, Physical Activity Questionnaire for Children, Leisure Score Index, walking test, handgrip strength test, ergospirometry test, jumping test |
| | Psychiatric | | Anxiety | the multidimensional anxiety scale for children |
| | | | Depression | children's depression scale |
| Álvarez-Bueno et al., 2017 | Cognitive Functioning | Performance in core executive functions | Cognitive flexibility | Performance in core executive working cognitive flexibility |
| | | Metacognition | Metacognition | metacognition: including higher-level executive functions and cognitive life skills |
| | | Performance in non-executive cognitive function | Non-executive cognitive function | Performance in non-executive cognitive function (NR which non-executive functions) |
| | | Performance in core executive functions | Selective attention-inhibition | Performance in core executive working selective attention-inhibition |
| | | Performance in core executive functions | Working memory | Performance in core executive working memories |
| Andrade et al., 2018 | Blood and Lymphatic System | biochemical measurements | Cholesterol | NR |
| | | biochemical measurements | Triglycerides | NR |
| | Cardiac | biochemical measurements | Blood pressure | NR |
| | General | anthropometric measurements | Body mass index (BMI) | NR |

| Study (Year) | Domain | Generic outcome (as stated by the authors) | Outcome | Outcome measure |
|---|---|---|---|---|
| | | anthropometric measurements | waist circumference | NR |
| Andrade et al., 2019 | Emotional Functioning/Wellbeing | Psychological effects | Athletic competence | scales, questionnaires / survey. |
| | | Psychological effects | Behavioral conduct | scales, questionnaires / survey. |
| | | Psychological effects | Intrinsic motivation | scales, questionnaires / survey. |
| | | Psychological effects | Liking to enjoy the exergame | scales, questionnaires / survey. |
| | | Psychological effects | Perceived competence | scales, questionnaires / survey. |
| | | Psychological effects | Physical competence | scales, questionnaires / survey. |
| | | Psychological effects | Positive expectations | scales, questionnaires / survey. |
| | | Psychological effects | Psychological attraction to exergame | scales, questionnaires / survey. |
| | | Psychological effects | Satisfaction | scales, questionnaires / survey. |
| | | Psychological effects | Self-concept (self-steem) | scales, questionnaires / survey. |
| | | Psychological effects | Self-efficacy | scales, questionnaires / survey. |
| | | Psychological effects | Self-worth (self-steem) | scales, questionnaires / survey. |
| | Psychiatric | Psychological effects | Anxiety | scales, questionnaires / survey. |
| | | Psychological effects | Depression | scales, questionnaires / survey. |
| | Social Functioning | Psychological effects | Externalizing composite | scales, questionnaires / survey. |
| | | Psychological effects | Interpersonal relations | scales, questionnaires / survey. |
| | | Psychological effects | Peer support | scales, questionnaires / survey. |
| | | Psychological effects | Relationship with parents | scales, questionnaires / survey. |

| Study (Year) | Domain | Generic outcome (as stated by the authors) | Outcome | Outcome measure |
|---|---|---|---|---|
| | | Psychological effects | Social acceptance | scales, questionnaires / survey. |
| | | Psychological effects | Social stress | scales, questionnaires / survey. |
| Azevedo et al., 2023 | General | BMI and BMI z-score | Body mass index (BMI) | objectively measured (not-self-reported) weight and height |
| | | BMI and BMI z-score | Body mass index (BMI) | objectively measured (not-self-reported) weight and height |
| Bento et al., 2022 | Emotional Functioning/Wellbeing | Physical Fitness | Benefits | 8-item questionnaire |
| | | Physical Fitness | Competence support | 8-item questionnaire |
| | | Physical Fitness | Effort | 8-item questionnaire |
| | | Physical Fitness | Enjoyment | 8-item questionnaire |
| | | Physical Fitness | Fun | 8-item questionnaire |
| | | Physical Fitness | Importance | 8-item questionnaire |
| | | Physical Fitness | Intrinsic motivation | 8-item questionnaire |
| | | Physical Fitness | Pleasure | 8-item questionnaire |
| | | Physical Fitness | Restlessness | 8-item questionnaire |
| | | Physical Fitness | Satisfaction related to participation in PA | 8-item questionnaire |
| | General | Physical Fitness | Body fat | Body composition: waist circumference; body mass; body fat; lean body mass; sum of skinfolds; Power, assessed through jump tests; |
| | | Physical Fitness | Body mass | Body composition: waist circumference; body mass; body fat; lean body mass; sum of skinfolds; Power, assessed through jump tests; |
| | | Physical Fitness | Body mass index (BMI) | Body composition: waist circumference; body mass; body fat; lean body mass; sum of skinfolds; Power, assessed through jump tests; |

| Study (Year) | Domain | Generic outcome (as stated by the authors) | Outcome | Outcome measure |
|---|---|---|---|---|
| | | Physical Fitness | Body mass index (BMI) | Body composition: waist circumference; body mass; body fat; lean body mass; sum of skinfolds; Power, assessed through jump tests; |
| | | Physical Fitness | Lean body mass | Body composition: waist circumference; body mass; body fat; lean body mass; sum of skinfolds; Power, assessed through jump tests; |
| | | Physical Fitness | Sum of skinfolds | Body composition: waist circumference; body mass; body fat; lean body mass; sum of skinfolds; Power, assessed through jump tests; |
| | Physical Functioning | Physical Fitness | Agility | Body composition: waist circumference; body mass; body fat; lean body mass; sum of skinfolds; Power, assessed through jump tests; |
| | | Physical Fitness | Power | Body composition: waist circumference; body mass; body fat; lean body mass; sum of skinfolds; Power, assessed through jump tests; |
| | | Physical Fitness | Running speed | Body composition: waist circumference; body mass; body fat; lean body mass; sum of skinfolds; Power, assessed through jump tests; |
| | | Physical Fitness | Upper strength | Body composition: waist circumference; body mass; body fat; lean body mass; sum of skinfolds; Power, assessed through jump tests; |
| | | Physical Fitness | Moderate Physical Activity | assessed PA subjectively through questionnaires, whereas in the other two studies, it was objectively measured using accelerometers. |
| | | Physical Fitness | Reducing sedentary activity | assessed PA subjectively through questionnaires, whereas in the other two studies, it was objectively measured using accelerometers. |

| Study (Year) | Domain | Generic outcome (as stated by the authors) | Outcome | Outcome measure |
| --- | --- | --- | --- | --- |
| | | Physical Fitness | Total Physical Activity | assessed PA subjectively through questionnaires, whereas in the other two studies, it was objectively measured using accelerometers. |
| | | Physical Fitness | Vigorous Physical Activity | assessed PA subjectively through questionnaires, whereas in the other two studies, it was objectively measured using accelerometers. |
| Birch et al., 2019 | Blood and Lymphatic System | Lipid Profile | Lipid profile | NR |
| | Cardiac | Blood Pressure | Blood Pressure | NR |
| | Endocrine | Glucose, Inflammation, Insulin sensitivity/resistance, liver function | Alanine aminotransferase (ALT) | NR |
| | | Glucose, Inflammation, Insulin sensitivity/resistance, liver function | C-reactive protein | NR |
| | | Glucose, Inflammation, Insulin sensitivity/resistance, liver function | Glucose | NR |
| | | Glucose, Inflammation, Insulin sensitivity/resistance, liver function | HOMA-index (Homeostatic Model Assessment for Insulin Resistance) | NR |
| | | Glucose, Inflammation, Insulin sensitivity/resistance, liver function | IL-6 | NR |

| Study (Year) | Domain | Generic outcome (as stated by the authors) | Outcome | Outcome measure |
|---|---|---|---|---|
| | | Glucose, Inflammation, Insulin sensitivity/resistance, liver function | Inflammation | NR |
| | | Glucose, Inflammation, Insulin sensitivity/resistance, liver function | Insulin | NR |
| | | Glucose, Inflammation, Insulin sensitivity/resistance, liver function | Insulin sensitivity/resistance | NR |
| | | Glucose, Inflammation, Insulin sensitivity/resistance, liver function | liver function | NR |
| | General | BMI measures and adiposity measures | Body fat-SDS | NR |
| | | BMI measures and adiposity measures | Body mass | NR |
| | | BMI measures and adiposity measures | Body mass index (BMI) | NR |
| | | BMI measures and adiposity measures | Fat | NR |
| | | BMI measures and adiposity measures | Fat mass | NR |
| | | BMI measures and adiposity measures | Fat-free mass | NR |
| | | BMI measures and adiposity measures | Percentage body fat | NR |

| Study (Year) | Domain | Generic outcome (as stated by the authors) | Outcome | Outcome measure |
|---|---|---|---|---|
| | | BMI measures and adiposity measures | Waist circumference | NR |
| | | BMI measures and adiposity measures | Waist circumference-SDS | NR |
| Boff et al., 2017 | General | Weight loss | Body mass index (BMI) | BMI through Z-scores or BMI or BMI percentile |
| | | Weight loss | Body weight | NR |
| Bondyra-Wisniewska et al., 2021 | Blood and Lymphatic System | Lipid Parameters | High density lipoprotein (HDL) | NR |
| | | Lipid Parameters | Low density lipoprotein (LDL) | NR |
| | | Lipid Parameters | Total cholesterol | NR |
| | | Lipid Parameters | Triglycerides | NR |
| | Cardiac | Blood Pressure | Diastolic blood pressure | NR |
| | | Blood Pressure | Systolic blood pressure | NR |
| | General | Anthropometric | Body mass index (BMI) | NR |
| | | Anthropometric | zBMI | NR |
| Braaksmaa et al., 2018 | Physical Functioning | Cardiorespiratory Fitness | Cardiorespiratory fitness | Measured by different tests as Eurofit endurance shuttle run test or 20 m shuttle run test or maximal graded cycle ergometer test or maximal graded treadmill test or 6 minute walk test or PACER (progressive aerobic cardiovascular endurance run) or YAPHV (years of age to peak height velocity (skeletal maturity)) or VO2peak or yo-yo intermittent recovery level 1 or yo–yo intermittent endurance test |
| Brown et al., 2015 | General | Obesity outcomes | Body mass index (BMI) | Body mass index (kg/m²) or body mass index z-score (zBMI) |

| Study (Year) | Domain | Generic outcome (as stated by the authors) | Outcome | Outcome measure |
|---|---|---|---|---|
| | | Obesity outcomes | Body weight | NR |
| | | Obesity outcomes | Waist circumference | NR |
| Brown et al., 2019 | General | obesity | Per cent fat content | NR |
| | | obesity | Ponderal index | NR |
| | | obesity | Prevalence of overweight and obesity | NR |
| | | obesity | Skin-fold thickness | NR |
| | | obesity | Weight and height | NR |
| | | obesity | zBMI score/BMI | Weight and height |
| Burns et al., 2019 | Endocrine | Insulin sensitivity | Fasting glucose | NR |
| | | Insulin sensitivity | Fasting insulin | NR |
| Busnatu et al., 2022 | Blood and Lymphatic System | Cardiometabolic Factors | Glucose | NR |
| | | Cardiometabolic Factors | High density lipoprotein (HDL) | NR |
| | | Cardiometabolic factors | Low density lipoprotein (LDL) | NR |
| | Cardiac | Cardiometabolic Factors | Diastolic blood pressure | NR |
| | | Cardiometabolic Factors | Systolic blood pressure | NR |
| | General | Cardiometabolic Factors | Body mass index (BMI) | NR |
| | | Cardiometabolic Factors | Body weight | NR |
| | | Cardiometabolic Factors | Waist circumference | NR |
| Cao et al., 2019 | Physical Functioning | Cardiorespiratory Fitness | VO2max | yoyo test; 20 meters shuttle run test |

| Study (Year) | Domain | Generic outcome (as stated by the authors) | Outcome | Outcome measure |
|---|---|---|---|---|
| Cao et al., 2021 | Blood and Lymphatic System | Cardiometabolic risk factors | High density lipoprotein (HDL) | NR |
| | | Cardiometabolic risk factors | Low density lipoprotein (LDL) | NR |
| | | Cardiometabolic risk factors | Total cholesterol | NR |
| | | Cardiometabolic risk factors | Triglycerides | NR |
| | Cardiac | Cardiometabolic risk factors | Diastolic blood pressure | NR |
| | | Cardiometabolic risk factors | Systolic blood pressure | NR |
| | Endocrine | Cardiometabolic risk factors | Glucose | NR |
| | | Cardiometabolic risk factors | HOMA-index (Homeostatic Model Assessment for Insulin Resistance) | NR |
| | | Cardiometabolic risk factors | Insulin | NR |
| | General | Cardiometabolic risk factors | body fat % | NR |
| | | Cardiometabolic risk factors | Body mass | NR |
| | | Cardiometabolic risk factors | Body mass index (BMI) | NR |
| | | Cardiometabolic risk factors | Fat-free mass | NR |
| | | Cardiometabolic risk factors | visceral adipose tissue | NR |
| | | Cardiometabolic risk factors | Waist Circumference | NR |
| | Physical Functioning | Cardiometabolic risk factors | VO2max | NR |
| Casas et al., 2018 | Endocrine | Adiposity | Glucose tolerance | NR |
| | General | Body adiposity | Body mass index (BMI) | NR |
| | | Adiposity | Abdominal adiposity | NR |

| **Study (Year)** | **Domain** | **Generic outcome (as stated by the authors)** | **Outcome** | **Outcome measure** |
|---|---|---|---|---|
| | | Adiposity | Body composition | NR |
| | | Adiposity | Body mass index (BMI) | NR |
| | | Adiposity | Fat % | NR |
| | | Adiposity | fat mass | NR |
| | | Adiposity | Fat-free mass | NR |
| | | motor performance | Metabolic parameters | NR |
| | Physical Functioning | Muscle fitness | Medicine ball launch performance | NR |
| | | General motor performance | Vertical jump performance | NR |
| | | motor performance | strength | NR |
| Cesa et al., 2014 | Blood and Lymphatic System | Cardiometabolic risk factors | Triglycerides | NR |
| | Cardiac | Cardiometabolic risk factors | Diastolic blood pressure | NR |
| | | Cardiometabolic risk factors | Systolic blood pressure | NR |
| | General | Cardiometabolic risk factors | Body mass index (BMI) | NR |
| Chen et al., 2021 | Blood and Lymphatic System | Physical Indexes and Cardiovascular Risk Factors | High density lipoprotein (HDL) | NR |
| | | Physical Indexes and Cardiovascular Risk Factors | Low density lipoprotein (LDL) | NR |
| | | Physical Indexes and Cardiovascular Risk Factors | Total cholesterol | NR |
| | | Physical Indexes and Cardiovascular Risk Factors | Triglycerides | NR |

| **Study (Year)** | **Domain** | **Generic outcome (as stated by the authors)** | **Outcome** | **Outcome measure** |
|---|---|---|---|---|
| | Endocrine | Physical Indexes and Cardiovascular Risk Factors | Insulin | NR |
| | | Physical Indexes and Cardiovascular Risk Factors | Insulin resistance | NR |
| | General | Physical Indexes and Cardiovascular Risk Factors | body fat % | NR |
| | | Physical Indexes and Cardiovascular Risk Factors | Body mass index (BMI) | NR |
| | Physical Functioning | Physical Indexes and Cardiovascular Risk Factors | VO2peak | NR |
| Cheng et al., 2022 | Vascular | PWV, IMT and FMD. | Flow-mediated dilation (FMD) | NR |
| | | PWV, IMT and FMD. | Intima-media thickness | NR |
| | | PWV, IMT and FMD. | Pulse wave velocity | NR |
| Choe et al., 2022 | General | Changes in BMI | Body mass index (BMI) | NR |
| | | Changes in BMI | Body weight | NR |
| Clemente et al., 2022 | Blood and Lymphatic System | Health-related outcomes | High density lipoprotein (HDL) | NR |
| | | Health-related outcomes | Number of erythrocytes | NR |
| | | Health-related outcomes | Total cholesterol | NR |
| | | Health-related outcomes | Triglycerides | NR |
| | Cardiac | Health-related outcomes | Mean arterial blood pressure | NR |
| | | Health-related outcomes | Parasympathetic activity | NR |

| Study (Year) | Domain | Generic outcome (as stated by the authors) | Outcome | Outcome measure |
|---|---|---|---|---|
| | | Health-related outcomes | Resting and maximal heart rate | NR |
| | | Health-related outcomes | Sympathetic activity | NR |
| | | Health-related outcomes | Systolic blood pressure | NR |
| | Endocrine | Health-related outcomes | C-reactive protein | NR |
| | | Health-related outcomes | Metabolic syndrome | NR |
| | General | Body composition | Body fat percentage | NR |
| | | Body composition | Body mass index (BMI) | NR |
| | | Body composition | Height | NR |
| | | Body composition | Waist circumference | NR |
| | | Body composition | Weight | NR |
| | Physical Functioning | Physical fitness | Agility | NR |
| | | Physical fitness | Balance | NR |
| | | Physical fitness | Cardiorespiratory fitness | NR |
| | | Physical fitness | Explosive power | NR |
| | | Physical fitness | Flexibility | NR |
| | | Physical fitness | Jump ability | NR |
| | | Physical fitness | Maximal power output (POmax) | NR |
| | | Physical fitness | VO2peak | NR |
| | Vascular | Health-related outcomes | Vascular conductance | NR |
| | | Health-related outcomes | Vascular resistance | NR |

| **Study (Year)** | **Domain** | **Generic outcome (as stated by the authors)** | **Outcome** | **Outcome measure** |
|---|---|---|---|---|
| Collins et al., 2018 | General | Obesity (weight status) | Body fat mass (% and kg) | NR |
| | | Obesity (weight status) | Body fat-free mass (kg) | NR |
| | | Obesity (weight status) | Body mass index (BMI) | BMI (kg/m2); BMI percentile; BMI z-score |
| | | Obesity (weight status) | Body weight | NR |
| | | Obesity (weight status) | Lean body mass | NR |
| | | Obesity (weight status) | Skinfold thickness (mm) | NR |
| | | Obesity (weight status) | Waist circumference (cm) | NR |
| Collins et al., 2019 | Emotional Functioning/Wellbeing | "THE SELF (The term ‘the self’ is used in this paper to capture a range of specific terms that, while separate, are related (e.g. self-esteem, self-efficacy, self-perceptions)." | Body attractiveness | Questionnaires |
| | | "THE SELF (The term ‘the self’ is used in this paper to capture a range of specific terms that, while separate, are related (e.g. self-esteem, self-efficacy, self-perceptions)." | Exercise self-efficacy | Questionnaires |

| Study (Year) | Domain | Generic outcome (as stated by the authors) | Outcome | Outcome measure |
|---|---|---|---|---|
| | | "THE SELF (The term 'the self' is used in this paper to capture a range of specific terms that, while separate, are related (e.g. self-esteem, self-efficacy, self-perceptions)." | Global self-esteem | Questionnaires |
| | | "THE SELF (The term 'the self' is used in this paper to capture a range of specific terms that, while separate, are related (e.g. self-esteem, self-efficacy, self-perceptions)." | Physical condition | Questionnaires |
| | | "THE SELF (The term 'the self' is used in this paper to capture a range of specific terms that, while separate, are related (e.g. self-esteem, self-efficacy, self-perceptions)." | Physical self-worth | Questionnaires |
| | | "THE SELF (The term 'the self' is used in this paper to capture a range of specific terms that, while separate, are related | Physical strength | Questionnaires |

| Study (Year) | Domain | Generic outcome (as stated by the authors) | Outcome | Outcome measure |
| --- | --- | --- | --- | --- |
| | | (e.g. self-esteem, self-efficacy, self-perceptions)." | | |
| | | "THE SELF (The term ‘the self’ is used in this paper to capture a range of specific terms that, while separate, are related (e.g. self-esteem, self-efficacy, self-perceptions)." | Resistance training beliefs | Questionnaires |
| | | "THE SELF (The term ‘the self’ is used in this paper to capture a range of specific terms that, while separate, are related (e.g. self-esteem, self-efficacy, self-perceptions)." | Resistance training confidence | Questionnaires |
| | | "THE SELF (The term ‘the self’ is used in this paper to capture a range of specific terms that, while separate, are related (e.g. self-esteem, self-efficacy, self-perceptions)." | RT self-efficacy | Questionnaires |

| Study (Year) | Domain | Generic outcome (as stated by the authors) | Outcome | Outcome measure |
|---|---|---|---|---|
| | | "THE SELF (The term 'the self' is used in this paper to capture a range of specific terms that, while separate, are related (e.g. self-esteem, self-efficacy, self-perceptions)." | sport competence | Questionnaires |
| Colquitt et al., 2016 | Adverse Events | | Adverse events | NR |
| | Blood and Lymphatic System | | High density lipoprotein (HDL) | NR |
| | | | Triglycerides | NR |
| | Endocrine | | Insulin levels | NR |
| | | | Insulin resistance | NR |
| | General | BMI variable | Body Mass Index (BMI) | NR |
| | | BMI variable | Waist circumference | NR |
| | | BMI variable | Body weight | NR |
| | | BMI variable | Visceral fat | NR |
| | | BMI variable | Abdominal subcutaneous fat | NR |
| | Global Quality of Life | | Health-related QOL | NR |
| | Metabolism and Nutrition | | Sugar intake | NR |
| | | | Fast food intake | NR |
| | Physical Functioning | | Behavior change | NR |
| | Social Functioning | | Parent-child relationship | NR |

| Study (Year) | Domain | Generic outcome (as stated by the authors) | Outcome | Outcome measure |
|---|---|---|---|---|
| Costa et al., 2020 | Blood and Lymphatic System | TC (total cholesterol) and LDL levels. | Low density lipoprotein (LDL) | NR |
| | | TC (total cholesterol) and LDL levels. | Total cholesterol | NR |
| da Rosa-Santos et al., 2019 | Blood and Lymphatic System | CRF (Cardiometabolic Risk Factors) | High density lipoprotein (HDL) | NR |
| | | CRF (Cardiometabolic Risk Factors) | Low density lipoprotein (LDL) | NR |
| | | CRF (Cardiometabolic Risk Factors) | Triglycerides | NR |
| | Cardiac | CRF (Cardiometabolic Risk Factors) | Diastolic blood pressure | NR |
| | | CRF (Cardiometabolic Risk Factors) | Systolic blood pressure | NR |
| | Emotional Functioning/Wellbeing | CRF (Cardiometabolic Risk Factors) | Quality of life | NR |
| | Endocrine | CRF (Cardiometabolic Risk Factors) | Butyrylcholinesterase activity (BChE) | NR |
| | General | CRF (Cardiometabolic Risk Factors) | Adiposity | NR |
| | | CRF (Cardiometabolic Risk Factors) | Body mass | NR |
| | | CRF (Cardiometabolic Risk Factors) | Body mass index (BMI) | NR |
| | | CRF (Cardiometabolic Risk Factors) | Fat-free mass | NR |

| Study (Year) | Domain | Generic outcome (as stated by the authors) | Outcome | Outcome measure |
|---|---|---|---|---|
| | | CRF (Cardiometabolic Risk Factors) | Waist and hip circumference | NR |
| | | CRF (Cardiometabolic Risk Factors) | Waist circumference | NR |
| | Physical Functioning | CRF (Cardiometabolic Risk Factors) | Body flexibility | NR |
| | | CRF (Cardiometabolic Risk Factors) | Muscle endurance | NR |
| | | CRF (Cardiometabolic Risk Factors) | Physical activity level | NR |
| | Vascular | CRF (Cardiometabolic Risk Factors) | Endothelium-dependent vascular dilatation | NR |
| Delgado-Floody et al., 2019 | General | body compositions | Body mass | NR |
| | | body compositions | Body mass index (BMI) | NR |
| | | body compositions | Lean body mass | NR |
| | | body compositions | Skinfold thickness | NR |
| | | body compositions | Sum of skinfolds | NR |
| | | body compositions | trunk fat % | NR |
| | | body compositions | Waist circumference | NR |
| | Physical Functioning | cardiorespiratory fitness | VO2max | NR |
| | | cardiorespiratory fitness | VO2max/CRF | YYIET= Yo-Yo intermittent endurance test |
| Deshira et al., 2022 | Cardiac | Blood pressure | Blood pressure | PACER score |
| | | Blood pressure | Diastolic blood pressure | NR |
| | | Blood pressure | Systolic blood pressure | NR |

| Study (Year) | Domain | Generic outcome (as stated by the authors) | Outcome | Outcome measure |
|---|---|---|---|---|
| | General | Health outcomes (physical) | Body mass index (BMI) | NR |
| | | Health outcomes (physical) | Waist circumference | NR |
| | | Health outcomes (physical) | Weight reduction | NR |
| Dias et al., 2018 | Metabolism and Nutrition | Knowledge and/or behavioral change of diet | Portions of fruit, vegetables and water | Fruit, vegetables and water portions intake by 24- hour dietary recall |
| | Physical Functioning | Knowledge and/or behavioral change of physical activity | Time spent in moderate to vigorous physical activity | Minutes of moderate to vigorous physical exercise by accelerometry (5 consecutive days) |
| Dias et al., 2015 | General | Body composition | Body fat-free mass | NR |
| | | Body composition | Body weight | NR |
| | Physical Functioning | Cardiorespiratory Fitness | Peak oxygen consumption (VO2 peak) | Measured by VO2 peak |
| | Vascular | Vascular function | Flow-mediated dilation (FMD) | Measured by FMD peak (%) or FMD area under the curve (AUC) (%s) or FMD with glyceryl trinitrate (GTN) (%) or Resting brachial artery (BA) diameter |
| Dias et al., 2021 | Cardiac | Heart Rate Variability | HF | NR |
| | | Heart Rate Variability | LF | NR |
| | | Heart Rate Variability | LF/HF | NR |
| | | Heart Rate Variability | Mean RR | NR |
| | | Heart Rate Variability | RMSSD | NR |
| | | Heart Rate Variability | SD1 | NR |
| | | Heart Rate Variability | SD2 | NR |
| | | Heart Rate Variability | SDNN | NR |

| Study (Year) | Domain | Generic outcome (as stated by the authors) | Outcome | Outcome measure |
|---|---|---|---|---|
| | General | Anthropometric | Body Fat | NR |
| | | Anthropometric | Body mass index (BMI) | NR |
| Dobbins et al., 2013 | Blood and Lymphatic System | Lipid profile | Total cholesterol | Blood samples were taken for some studies after fasting, and for others with no fasting. |
| | Cardiac | Blood pressure | Mean systolic and diastolic blood pressure | Measured either manually using a mercury sphygmomanometer or via a Dinamap machine. The timing of when blood pressure was measured differed significantly across studies with timing varying between 5 and 15 min following active periods during school hours. |
| | | Pulse rate | Resting heart rate (beats/minute) | This outcome was measured by trained professionals during school time, during seated rest. |
| | | Blood pressure | Systolic blood pressure | Measured either manually using a mercury sphygmomanometer or via a Dinamap machine. The timing of when blood pressure was measured differed significantly across studies with timing varying between 5 and 15 min following active periods during school hours. |
| | General | BMI | Body mass index (BMI) | ($kg/m^2$)This outcome was measured by trained health professionals using calibrated scales. |
| | Physical Functioning | Maximal oxygen consumption | Maximal oxygen consumption (VO2max) | This outcome was measured in different ways by trained professionals. In some instances pulse rate recovery was used as a proxy for VO2max and in other instances actual maximal oxygen uptake was measured. |
| | | Rate of moderate to vigorous physical activity (MVPA) (per cent of sample engaged in MVPA) | Rates of moderate to vigorous physical activity during the school day | Rate assessed either through self-report or through the use of accelerometers (or both). The rate was calculated by dividing the number of students engaged in MVPA by the total number of students allocated to either the intervention or control group |

| Study (Year) | Domain | Generic outcome (as stated by the authors) | Outcome | Outcome measure |
|---|---|---|---|---|
| | | Duration of physical activity (time spent engaged in MVPA) | Time spent in moderate to vigorous physical activity | Time was measured as the total minutes per hour or week spent engaged in MVPA generally through self-report, although some studies collected these data via accelerometers |
| | | Television viewing (time spent watching TV) | Time spent watching television | Measured by self-report or parental report as the minutes per hour or week spent watching television |
| Eckstein et al., 2022 | Blood and Lymphatic System | High-density lipoprotein cholesterol | High density lipoprotein (HDL) | NR |
| | | Low-density lipoprotein cholesterol | Low density lipoprotein (LDL) | NR |
| | | Total cholesterol | Total cholesterol | NR |
| | | Triglycerides | Triglycerides | NR |
| | Cardiac | Cardiovascular risk factors | Diastolic blood pressure | NR |
| | | Cardiac autonomic function | Heart rate variability | NR |
| | | Cardiovascular risk factors | Mean systolic and diastolic blood pressure | NR |
| | | Cardiovascular risk factors | Systolic blood pressure | NR |
| | Endocrine | Myokines | Brain-derived neurotrophic factor (BDNF) | ng/mL |
| | | Insulin values | C-reactive protein | mg/L |
| | | Blood glucose | Fasting glucose | NR |
| | | Blood glucose | HbA1c | NR |
| | | Insulin values | Insulin | NR |
| | | Myokines | Irisin | ng/mL |

| Study (Year) | Domain | Generic outcome (as stated by the authors) | Outcome | Outcome measure |
|---|---|---|---|---|
| | | Myokines | Myostatin | NR |
| | | Serum oxidative stress biomarkers | Plasma malondialdehyde | mmol/mL |
| | | Myokines | Serum interleukin-15 | NR |
| | | Serum oxidative stress biomarkers | Superoxide dismutase | U/mL |
| | General | Body composition | Body fat mass | NR |
| | | Body composition | Body fat mass (%) | NR |
| | | Body composition | Body fat-free mass | NR |
| | | Anthropometric parameters | Body height | NR |
| | | Anthropometric parameters | Body mass | kg |
| | | Anthropometric parameters | Body mass index (BMI) | NR |
| | | Anthropometric parameters | Body weight | NR |
| | | Lean body mass | Lean body mass | NR |
| | | Regional fat | Regional fat | NR |
| | | Body composition | Trunk fat | NR |
| | | Anthropometric parameters | Waist circumference | NR |
| | Musculoskeletal and Connective Tissue | Body composition | Regional bone density | NR |
| | | Body composition | Total bone density | NR |
| | Physical Functioning | Physical fitness variables | Balance | NR |
| | | Physical fitness variables | Cardiorespiratory endurance | NR |
| | | Physical fitness variables | Cardiorespiratory fitness | NR |

| Study (Year) | Domain | Generic outcome (as stated by the authors) | Outcome | Outcome measure |
|---|---|---|---|---|
| | | Physical fitness variables | Flexibility | Sit and reach |
| | | Cardiorespiratory fitness | Gas exchange threshold | NR |
| | | Physical fitness variables | Maximal muscle strength | NR |
| | | Cardiorespiratory fitness | Maximum speed | km/h |
| | | Physical fitness variables | Muscle endurance | reps |
| | | Physical fitness variables | Muscle strength | NR |
| | | Physical fitness variables | Peak muscle power | Newton (N) |
| | | Cardiorespiratory fitness | Peak oxygen consumption (VO2 peak) | NR |
| | | Physical fitness variables | Power | Surgent vertical jump |
| | Vascular | Cardiovascular risk factors | Arterial stiffness | Right brachial-ankle pulse wave velocity (m/s-1) |
| Elber et al., 2019 | Blood and Lymphatic System | cardiovascular disease | Cholesterol | NR |
| | Cardiac | cardiovascular disease | Systolic blood pressure | NR |
| | General | obesity | body fat mass | NR |
| | | obesity | Body mass index (BMI) | NR |
| | Physical Functioning | cardiovascular disease | VO2 max | NR |
| Estéves-González et al., 2022 | Cardiac | Heart Rate Variability | HF | NR |
| | | Heart Rate Variability | LF | NR |
| | | Heart Rate Variability | LF/HF | NR |
| | | Heart Rate Variability | Mean RR | NR |

| Study (Year) | Domain | Generic outcome (as stated by the authors) | Outcome | Outcome measure |
|---|---|---|---|---|
| | | Heart Rate Variability | PNN50 | NR |
| | | Heart Rate Variability | RMSSD | NR |
| | | Heart Rate Variability | SDNN | NR |
| Farah et al., 2012 | Cardiac | Blood pressure change | Diastolic blood pressure | NR |
| | | Blood pressure change | Mean systolic and diastolic blood pressure | NR |
| | | Blood pressure change | Systolic blood pressure | NR |
| | General | Body mass | Body weight | NR |
| Feng et al., 2017 | General | Anthropometric | Body mass index (BMI) | NR |
| Frerichs et al., 2016 | General | Weight-related outcomes (obesity) | Body mass index (BMI) | BMI (kg/m2) or BMI z-scores (objective measurement of weight and height via calibrated scale and stadiometer) |
| | | Weight-related outcomes (obesity) | Body weight | Objective measurement of weight via calibrated scale |
| | | Weight-related outcomes (obesity) | Waist circumference | NR |
| | Metabolism and Nutrition | Diet behavior (obesity-related lifestyle behaviours) | Self-report of food servings | Self-reported questionnaire measures: fruit Servings, vegetable servings, sugar sweetened beverage servings, number of snacks/desserts per day |
| | Physical Functioning | Physical activity behavior (obesity-related lifestyle behaviours) | Time spent in moderate to vigorous physical activity | Measured by accelerometer or questionnaire |
| García-Hermoso et al., 2014 | Endocrine | Insulin resistance markers | Fasting glucose | Fasting glucose concentration was determined by enzymatic methods or using a glucose oxidase technique by using a radioimmunoassay (RIA) kit |

| **Study (Year)** | **Domain** | **Generic outcome (as stated by the authors)** | **Outcome** | **Outcome measure** |
|---|---|---|---|---|
| | | Insulin resistance markers | Fasting insulin | Fasting insulin levels were determined using a chemiluminescent assay, RIA, or by an Immulite analyzer |
| | General | Percentage of body fat | Body fat mass (%) | NR |
| García-Hermoso et al., 2015 | General | Body composition | Body fat mass (kg and %) | NR |
| | | Body composition | Body fat-free mass | NR |
| | | Body composition | Body mass index (BMI) | NR |
| | | Body composition | Body weight | NR |
| | | Body composition | Waist circumference | NR |
| García-Hermoso et al., 2016-a | Blood and Lymphatic System | Cardiometabolic risk factor (Lipids and lipoprotein) | High density lipoprotein (HDL) | The evaluations were made in the morning after 12 h of overnight fasting |
| | | Cardiometabolic risk factor (Lipids and lipoprotein) | Low density lipoprotein (LDL) | The evaluations were made in the morning after 12 h of overnight fasting |
| | | Cardiometabolic risk factor (Lipids and lipoprotein) | Total cholesterol | The evaluations were made in the morning after 12 h of overnight fasting |
| | | Cardiometabolic risk factor (Lipids and lipoprotein) | Triglycerides | The evaluations were made in the morning after 12 h of overnight fasting |
| | Cardiac | Cardiometabolic risk factor (blood pressure) | Diastolic blood pressure | Various methods were used to determine blood pressure |
| | | Cardiometabolic risk factor (blood pressure) | Systolic blood pressure | Various methods were used to determine blood pressure |
| | Endocrine | Cardiometabolic risk factor (Insulin resistance) | Fasting glucose | The evaluations were made in the morning after 12 h of overnight fasting |
| | | Cardiometabolic risk factor (Insulin resistance) | Fasting insulin | The evaluations were made in the morning after 12 h of overnight fasting |

| Study (Year) | Domain | Generic outcome (as stated by the authors) | Outcome | Outcome measure |
|---|---|---|---|---|
| | | Cardiometabolic risk factor (Insulin resistance) | HOMA-index (Homeostatic Model Assessment for Insulin Resistance) | The evaluations were made in the morning after 12 h of overnight fasting |
| | General | Body composition | Body fat mass (% or kg) | Measured by bioelectrical impedance or dual-energy x-ray absorptiometry |
| | | Body composition | Body mass index (BMI) | NR |
| | | Body composition | Body weight | Measure was assessed using a medical scale |
| | | Body composition | Waist circumference | Measured using the standard protocol |
| García-Hermoso et al., 2016-b | Blood and Lymphatic System | Lipids and lipoprotein | High density lipoprotein (HDL) | NR |
| | | Lipids and lipoprotein | Low density lipoprotein (LDL) | NR |
| | | Lipids and lipoprotein | Total cholesterol | NR |
| | | Lipids and lipoprotein | Triglycerides | NR |
| | Cardiac | Blood pressure | Systolic blood pressure | NR |
| | Endocrine | Adipokines | Adiponectin | NR |
| | | Insulin resistance | Fasting glucose | NR |
| | | Insulin resistance | Fasting insulin | NR |
| | | Insulin resistance | HOMA-index (Homeostatic Model Assessment for Insulin Resistance) | NR |
| | | Adipokines | Leptin | NR |

| **Study (Year)** | **Domain** | **Generic outcome (as stated by the authors)** | **Outcome** | **Outcome measure** |
|---|---|---|---|---|
| | General | Body composition | Body fat mass (kg and %) | NR |
| | | Body composition | Body mass index (BMI) | Body mass index (kg/m2) |
| | | Body composition | Body weight | NR |
| | | Body composition | Lean body mass | NR |
| | | Body composition | Subcutaneous adipose tissue (cm) | NR |
| | | Body composition | Visceral adipose tissue (cm) | NR |
| | | Body composition | Waist circumference (cm) | NR |
| García-Hermoso et al (2016-c) | Blood and Lymphatic System | Level of serum C-reactive protein | Serum C-reactive protein | All the techniques employed to determine CRP used ultrasensitive methods: nephelometry, latex-enhanced immunoturbidimetric assay, immunoradiometry assay, and highly sensitive enzyme-linked immunosorbent assay. |
| | Endocrine | Fasting insulin | Fasting insulin | NR |
| | General | Body composition (weight and body fat) | Body fat mass (%) | NR |
| | | Body composition (weight and body fat) | Body weight | NR |
| García-Hermoso et al., 2017-a | Endocrine | Adipokines | Adiponectin | NR |
| | | Adipokines | Leptin | NR |
| | | Adipokines | Resistin levels | NR |
| | | Adipokines | Visfatin levels | NR |

| Study (Year) | Domain | Generic outcome (as stated by the authors) | Outcome | Outcome measure |
|---|---|---|---|---|
| García-Hermoso et al., 2017-b | Vascular | Carotid Arterial Wall Thickness | Intima-media thickness (IMT) | Measured the carotid IMT by high-resolution B mode ultrasound at the carotid artery |
| Garcia-Hermoso et al., 2020-a | General | Adiposity | Body composition | DXA, MRI, bioelectrical impedance analysis |
| | | Adiposity | skinfold thickness | NR |
| | Physical Functioning | Cardiorespiratory Fitness | Cardiorespiratory fitness | maximal or submaximal tests on a cycle ergometer or treadmill with spirometry |
| Garcia-Hermoso et al., 2020-b | General | Physical Fitness | Body Fat | NR |
| | | Physical Fitness | Body mass index (BMI) | calculated as weight in kilograms divided by height in meters squared |
| | | Physical Fitness | skinfold thickness | NR |
| | | Physical Fitness | Waist Circumference | NR |
| | Physical Functioning | Physical Fitness | Cardiorespiratory fitness | NR |
| | | Fundamental Motor Skills | Fundamental motor skills | locomotor and object control skills |
| García-Hermoso et al., 2019 | Cardiac | Blood Pressure | Diastolic blood pressure | NR |
| | | Blood Pressure | Systolic blood pressure | NR |
| | General | Anthropometric | Body fat | NR |
| | | Anthropometric | Body mass index (BMI) | NR |
| | | Anthropometric | Obesity prevalence | NR |
| | | Anthropometric | Skinfold thickness | NR |
| | | Anthropometric | Waist Circumference | NR |
| | | Anthropometric | z-score BMI | NR |
| | Physical Functioning | Cardiorespiratory Fitness | Cardiorespiratory fitness | shuttle run test; 10x20m test |

| Study (Year) | Domain | Generic outcome (as stated by the authors) | Outcome | Outcome measure |
|---|---|---|---|---|
| | | Lower-body muscular strength | Lower-body muscular strength | squat jump |
| | | Speed-agility | Speed-agility | 10mx4 shuttle run test; obstacle course test; 20m test; 6m test |
| González-Ruiz et al., 2017 | Endocrine | Liver enzymes | Alanine aminotransferase (ALT) | Measured using commercially available test kits |
| | | Liver enzymes | Aspartate aminotransferase (AST) | Measured using commercially available test kits |
| | | Liver enzymes | Gamma-glutamyl transferase (GGT) | Measured using commercially available test kits |
| | | Intrahepatic fat | Intrahepatic fat | Measured with proton magnetic resonance spectra acquired in the same proton magnetic resonance spectroscopic system with a body matrix coil and a spine matrix |
| | General | Abdominal fat | Subcutaneous adipose tissue | Measured with magnetic resonance imaging or by ultrasound examination |
| | | Abdominal fat | Visceral adipose tissue | Measured with magnetic resonance imaging or by ultrasound examination |
| Gonzalez-Suarez et al., 2009 | General | Management of childhood obesity | Body fat mass (%) | Measured by bioelectric impedance, dual energy absorptiometry, or by the sum of skinfolds |
| | | Management of childhood obesity | Body mass index (BMI) | NR |
| | | Management of childhood obesity | Skinfold thickness | Triceps and subescapular skinfold thickness (mm) |
| | | Management of childhood obesity | Waist circumference | NR |

| Study (Year) | Domain | Generic outcome (as stated by the authors) | Outcome | Outcome measure |
|---|---|---|---|---|
| | | Management of childhood obesity | Waist-to-hip ratio | NR |
| Guerra et al., 2013 | General | Body mass index (BMI) | Body mass index (BMI) | $Kg/m^2$ |
| Guerra et al., 2014 | Cardiac | Blood pressure | Diastolic blood pressure | NR |
| | | Blood pressure | Systolic blood pressure | NR |
| | General | Body mass index (BMI) | Body mass index (BMI) | NR |
| | | Body weight | Body weight | NR |
| Hale et al., 2023 | Emotional Functioning/Wellbeing | Psychological well-being and ill-being of children | body image (steem, dissatisfaction) | Questionnaires and surveys, but it is not described, only the acronyms. |
| | | Psychological well-being and ill-being of children | Quality of life | Questionnaires and surveys, but it is not described, only the acronyms. |
| | | Psychological well-being and ill-being of children | self-perception | Questionnaires and surveys, but it is not described, only the acronyms. |
| | | Psychological well-being and ill-being of children | self-esteem | Questionnaires and surveys, but it is not described, only the acronyms. |
| | Psychiatric | Psychological well-being and ill-being of children | Anxiety | Questionnaires and surveys, but it is not described, only the acronyms. |
| | | Psychological well-being and ill-being of children | Depression | Questionnaires and surveys, but it is not described, only the acronyms. |
| | | Psychological well-being and ill-being of children | Poor well-being | Questionnaires and surveys, but it is not described, only the acronyms. |
| | | Psychological well-being and ill-being of children | Psychological ill-being | Questionnaires and surveys, but it is not described, only the acronyms. |
| | | Psychological well-being and ill-being of children | Stress | Questionnaires and surveys, but it is not described, only the acronyms. |

| Study (Year) | Domain | Generic outcome (as stated by the authors) | Outcome | Outcome measure |
|---|---|---|---|---|
| Hamel et al., 2011 | General | Health outcomes | Body fat mass (%) | % measured by DEXA |
| | | Health outcomes | Body mass index (BMI) | NR |
| | | Health outcomes | Body weight | NR |
| | | Health outcomes | Waist circumference | NR |
| | Physical Functioning | Physical activity | Days of moderate-vigorous physical activity per week | self report |
| | | Physical activity | Time spent in moderate to vigorous physical activity | Minutes of physical activity moderate-vigorous per week self-report or measured by accelerometry |
| Han et al., 2019 | Blood and Lymphatic System | | High density lipoprotein (HDL) | NR |
| | | | Low density lipoprotein (LDL) | NR |
| | | | Total cholesterol | NR |
| | | | Triglycerides | NR |
| | Cardiac | | Systolic blood pressure | NR |
| | | | Diastolic blood pressure | NR |
| | Endocrine | | Adiponectin | NR |
| | | | C-reactive protein | NR |
| | | | Fasting glucose | NR |
| | | | IL-6 | NR |
| | | | serum insulin | NR |

| **Study (Year)** | **Domain** | **Generic outcome (as stated by the authors)** | **Outcome** | **Outcome measure** |
|---|---|---|---|---|
| | | | TNF-α | NR |
| | General | | Body mass index (BMI) | NR |
| | | | Fat % | NR |
| | | | Fat-free mass | NR |
| | | | Waist Circumference | NR |
| | | | weight | NR |
| Harris et al., 2009 | General | Body composition | Body fat mass (% or kg) | NR |
| | | Body composition | Body fat mass (%) | NR |
| | | Body composition | Body mass index (BMI) | NR |
| | | Body composition | Lean body mass | NR |
| | | Body composition | Skinfold thickness | Triceps, subescapular and sum (mm) |
| | | Body composition | Waist circumference | NR |
| | | Body composition | Waist-to-hip ratio | NR |
| Hejazi et al., 2022 | Blood and Lymphatic System | HDL-C | High density lipoprotein (HDL) | NR |
| | | LDL-C | Low density lipoprotein (LDL) | NR |
| | | Triglycerides | Triglycerides | NR |
| | Endocrine | Adiponectin | Adiponectin | Adiponectin levels |
| | | C-Reactive Protein | C-reactive protein | NR |
| | | glucose metabolism | Fasting glucose | NR |
| | | glucose metabolism | HOMA-index (Homeostatic Model | NR |

| Study (Year) | Domain | Generic outcome (as stated by the authors) | Outcome | Outcome measure |
|---|---|---|---|---|
| | | | Assessment for Insulin Resistance) | |
| | | IL-6 | IL-6 | NR |
| | | glucose metabolism | Insulin | NR |
| | | Leptin | Leptin | NR |
| | Physical Functioning | VO2 max | VO2 max | NR |
| Hernandez-Martin et al., 2021 | General | body composition | Body mass index (BMI) | Stadiometer, Bioimpedance, DXA, Scale, stadiometer, Skinfold calliper, Skinfold calliper |
| | | body composition | Fat mass | Stadiometer, Bioimpedance, DXA, Scale, stadiometer, Skinfold calliper, Skinfold calliper |
| | | body composition | Lean body mass | Stadiometer, Bioimpedance, DXA, Scale, stadiometer, Skinfold calliper, Skinfold calliper |
| | Musculoskeletal and Connective Tissue | body composition | Bone mineral content | Stadiometer, Bioimpedance, DXA, Scale, stadiometer, Skinfold calliper, Skinfold calliper |
| Ho et al., 2013 | Blood and Lymphatic System | Metabolic | High density lipoprotein (HDL) | NR |
| | | Metabolic | Low density lipoprotein (LDL) | NR |
| | | Metabolic | Triglycerides | NR |
| | Endocrine | Metabolic | Fasting glucose | NR |
| | | Metabolic | Fasting insulin | NR |

| Study (Year) | Domain | Generic outcome (as stated by the authors) | Outcome | Outcome measure |
|---|---|---|---|---|
| | General | Weight change | Body mass index (BMI) | NR |
| | | Weight change | Body fat mass (%) | NR |
| | | Weight change | Lean body mass | NR |
| Jago et al., 2004 | Physical Functioning | Physical activity | Time spent in moderate to vigorous physical activity | Self-reported sweating; minutes of moderate to vigorous intensity physical activity from heart rate monitoring (50%/60% heart rate reserve); total self-resported physical activity (questionnaire); travel to school questionnaire to record self report mode of transportation; SOFIT to assess minutes of moderate intensity physical activity in PE lesson; SOPLAY to asses minutes of moderate intensity physical activity out of PE lesson. |
| Jull and Chen., 2013 | General | Weight loss | Body mass index (BMI) | BMI-SDS or z-BMI |
| | | Weight loss | Percentage overweight | [percent overweight = (effective BMI/BMI 50th percentile) –1] |
| Jung et al., 2018 | General | Visceral Fat | Visceral Fat | Computerized Tomography<br>Magnetic Resonance Imaging (MRI)<br>Dual energy x-ray absorptiometry (DEXA)<br>Ultrasound |
| Jurado-Castro et al., 2020 | Physical Functioning | Time spent engaged in MVPA | Minutes of MVPA | different protocols for accelerometer use. |
| | General | BMI | z-score BMI | NR |
| Kelley and Kelley., 2008 | Blood and Lymphatic System | High-density lipoprotein cholesterol (HDL-C) | High density lipoprotein (HDL) | The assessment of lipids and lipoproteins took place in the morning after |
| | | Non–high-density lipoprotein cholesterol (non–HDL-C) | Non-High-density lipoproteins (non–HDL-C) | calculated as total cholesterol (TC) minus HDL-C. The assessment of lipids and lipoproteins took place in the morning after |

| **Study (Year)** | **Domain** | **Generic outcome (as stated by the authors)** | **Outcome** | **Outcome measure** |
|---|---|---|---|---|
| | | Total cholesterol (TC) | Total cholesterol | The assessment of lipids and lipoproteins took place in the morning after |
| | General | Percent body fat | Body fat mass (%) | NR |
| | | Body weight | Body weight | NR |
| | Physical Functioning | cardiorespiratory capacity | Maximal oxygen consumption (VO2max) | (mL/kg/min) |
| Kelley et al., 2019 | Blood and Lymphatic System | | Fasting glucose | NR |
| | | | HbA1c | NR |
| | | | High density lipoprotein (HDL) | NR |
| | | | Low density lipoprotein (LDL) | NR |
| | | | Non-fasting glucose | NR |
| | | | Non-High-density lipoproteins (non–HDL-C) | NR |
| | | | Total cholesterol | NR |
| | | | Total cholesterol to HDL ratio | NR |
| | | | Triglycerides | NR |
| | Cardiac | | Diastolic blood pressure | NR |
| | | | Systolic blood pressure | NR |
| | Endocrine | | Fasting insulin | NR |
| | | | Non-fasting insulin | NR |

| **Study (Year)** | **Domain** | **Generic outcome (as stated by the authors)** | **Outcome** | **Outcome measure** |
|---|---|---|---|---|
| | General | Adiposity | Body fat % | NR |
| | | Adiposity | Body mass index (BMI) | NR |
| | | | body weight | NR |
| | | Adiposity | Fat mass | NR |
| | | | lean body mass | NR |
| | | | Waist circumference | NR |
| | | | waist/hip ratio | NR |
| | Metabolism and Nutrition | | Dietary intake | NR |
| | Physical Functioning | | Maximal strength | NR |
| | | | VO2max | NR |
| | | | Energy expenditure | NR |
| | | | Physical activity level | NR |
| Kelley et al., 2021 | Blood and Lymphatic System | Lipid and lipoprotein outcomes | High density lipoprotein (HDL) | NR (assessed with blood draw) |
| | | Lipid and lipoprotein outcomes | Low density lipoprotein (LDL) | NR (assessed with blood draw) |
| | | Lipid and lipoprotein outcomes | Total cholesterol | NR (assessed with blood draw) |
| | | Lipid and lipoprotein outcomes | Triglycerides | NR (assessed with blood draw) |
| | Cardiac | Blood pressure outcomes | Resting diastolic blood pressure (DBP) | Various types of sphygmomanometers |
| | | Blood pressure outcomes | Resting systolic blood pressure (SBP) | Various types of sphygmomanometers |

| Study (Year) | Domain | Generic outcome (as stated by the authors) | Outcome | Outcome measure |
|---|---|---|---|---|
| | Endocrine | Diabetes-related outcomes | Fasting glucose | NR (assessed with blood draw) |
| | | Diabetes-related outcomes | Fasting insulin | NR (assessed with blood draw) |
| | General | Adiposity outcomes | Body fat mass | Various methods |
| | | Adiposity outcomes | Body fat mass (%) | Various methods |
| | | Fat-free mass | Body fat-free mass | Various methods |
| | | Adiposity outcomes | Body mass index (BMI) | Measured from height and body weight |
| | | Body weight | Body weight | Various types of scales |
| | | Adiposity outcomes | Waist circumference | Tape measure |
| | Physical Functioning | Maximum oxygen consumption | Maximal oxygen consumption (VO2max) | Various methods |
| Kemp et al., 2021 | General | Cardiovascular Disease Risk | Alcohol and cigarette use | NR |
| | | Cardiovascular Disease Risk | Body mass index (BMI) | NR |
| | | Cardiovascular Disease Risk | Parents' alcohol use | NR |
| | | Cardiovascular Disease Risk | Parents' BMI | NR |
| | Metabolism and Nutrition | Cardiovascular Disease Risk | Fruit and vegetable intake | NR |
| | Physical Functioning | Cardiovascular Disease Risk | Physical activity level | NR |
| | Psychiatric | Cardiovascular Disease Risk | Depression | NR |
| Kim et al., 2017 | General | Body weight | Body weight | NR |
| Kropski et al., 2008 | General | Obesity | Body fat mass (%) | NR |
| | | Obesity | Body mass index (BMI) | NR |

| Study (Year) | Domain | Generic outcome (as stated by the authors) | Outcome | Outcome measure |
|---|---|---|---|---|
| | | Obesity | Obesity/overweight prevalence | NR |
| | | Obesity | Skinfold thickness | Triceps skinfold thickness (mm) |
| Laframboise et al., 2011 | Endocrine | Adiponectin levels | Adiponectin | NR |
| | General | Adiposity | Body fat mass (%) | NR |
| | | Adiposity | Body mass index (BMI) | BMI SD-score |
| | | Adiposity | Body weight | NR |
| | | Adiposity | Skinfold thickness | NR |
| | | Adiposity | Trunk and visceral fat composition | NR |
| | | Adiposity | Waist circumference | NR |
| | | Adiposity | Waist-to-hip ratio | NR |
| Lee et al., 2020 | Blood and Lymphatic System | Cardiometabolic factors | High density lipoprotein (HDL) | NR |
| | | Cardiometabolic factors | Low density lipoprotein (LDL) | NR |
| | | Cardiometabolic factors | Total cholesterol | NR |
| | | Cardiometabolic factors | Triglycerides | NR |
| | Cardiac | Blood Pressure | Diastolic blood pressure | NR |
| | | Blood Pressure | Systolic blood pressure | NR |
| | Endocrine | Inflammatory Markers | C-reactive protein | NR |
| | | Inflammatory Markers | Fasting glucose | NR |
| | | Inflammatory Markers | IL-6 | NR |

| **Study (Year)** | **Domain** | **Generic outcome (as stated by the authors)** | **Outcome** | **Outcome measure** |
|---|---|---|---|---|
| | | Inflammatory Markers | Insulin | NR |
| | | Inflammatory Markers | Insulin resistance | HOMA-IR |
| | | Inflammatory Markers | TNF-alfa | NR |
| | General | Body fat | Body mass index (BMI) | NR |
| | | Body fat | Fat-free mass | NR |
| | | Body fat | Waist Circumference | NR |
| | Physical Functioning | Physical Fitness | VO2peak | NR |
| Li and Chen et al., 2021 | Blood and Lymphatic System | CRF (Cardiometabolic Risk Factors) | High density lipoprotein (HDL) | NR |
| | | CRF (Cardiometabolic Risk Factors) | Low density lipoprotein (LDL) | NR |
| | | CRF (Cardiometabolic Risk Factors) | Total Cholesterol (TC) | NR |
| | | CRF (Cardiometabolic Risk Factors) | Triglyceride (TG) | NR |
| | Endocrine | CRF (Cardiometabolic Risk Factors) | HOMA-index (Homeostatic Model Assessment for Insulin Resistance) | NR |
| | General | CRF (Cardiometabolic Risk Factors) | Body mass index (BMI) | NR |
| | Physical Functioning | CRF (Cardiometabolic Risk Factors) | Peak oxygen consumption (VO2 peak) | NR |

| **Study (Year)** | **Domain** | **Generic outcome (as stated by the authors)** | **Outcome** | **Outcome measure** |
|---|---|---|---|---|
| Li et al., 2022 | Blood and Lymphatic System | Changes in BMI | High density lipoprotein (HDL) | NR |
| | | Changes in BMI | Low density lipoprotein (LDL) | NR |
| | | Changes in BMI | Total cholesterol | NR |
| | | Changes in BMI | Triglycerides | NR |
| | Cardiac | Changes in BMI | Blood Pressure | NR |
| | Endocrine | Changes in BMI | Fasting glucose | NR |
| | General | Changes in BMI | Body mass index (BMI) | NR |
| Littlewood et al., 2020 | Cardiac | Cardiometabolic factors | Diastolic blood pressure | NR |
| | | Cardiometabolic factors | Systolic blood pressure | NR |
| | Emotional Functioning/Wellbeing | Behavioural | Screen time | NR |
| | Endocrine | Cardiometabolic factors | Fasting insulin | NR |
| | | Cardiometabolic factors | HbA1c | NR |
| | | Cardiometabolic factors | Insulin resistance | HOMA-IR |
| | General | Anthropometric | Body fat % | NR |
| | | Anthropometric | Body mass index (BMI) | NR |
| | | Anthropometric | Fat mass | NR |
| | | Anthropometric | Waist circumference | NR |
| | Physical Functioning | Cardiometabolic factors | Physical activity levels | steps/day; IPAQ; accelerometer |
| | | Cardiometabolic factors | VO2max | NR |
| | Psychiatric | Psychological | HR-QOL | NR |

| Study (Year) | Domain | Generic outcome (as stated by the authors) | Outcome | Outcome measure |
|---|---|---|---|---|
| | | Psychological | overall psychological profile | NR |
| Liu et al., 2020 | Physical Functioning | Fundamental Motor Skills | Fundamental motor skills | Test of Gross Motor Development, balance (test children's balance); agility (reaction times); physical perception (Youth Physical Self Youth Physical Self and Pictorial Scale of Perceived Competence) |
| | | Physical Fitness | Physical fitness | NR |
| Lopes et al., 2019 | Blood and Lymphatic System | blood concentration of HDL-C | High density lipoprotein (HDL) | NR |
| Martin-Smith et al., 2020 | General | Body Composition | Body composition | |
| | Physical Functioning | Cardiorespiratory Fitness | VO2max | Direct and indirect methods |
| Mclean et al., 2003 | General | Weight management | Body mass index (BMI) | NR |
| | | Weight management | Body weight | NR |
| Mead et al., 2017 | Adverse Events | | Morbidity | NR |
| | | | Adverse events | NR |
| | Blood and Lymphatic System | | High density lipoprotein (HDL) | NR |
| | | | Low density lipoprotein (LDL) | NR |
| | | | Total cholesterol | NR |
| | | | Triglycerides | NR |
| | Cardiac | | Systolic blood pressure | NR |
| | | | Diastolic blood pressure | NR |
| | | | Heart rate change | NR |

| Study (Year) | Domain | Generic outcome (as stated by the authors) | Outcome | Outcome measure |
|---|---|---|---|---|
| | Delivery of Care | | Quality of care | NR |
| | Emotional Functioning/Wellbeing | | Self-perception | NR |
| | | | Self-esteem | NR |
| | | | Self-worth (self-perception) | NR |
| | | | Mood | NR |
| | | | Physical activity enjoyment | NR |
| | Endocrine | | Fasting insulin | NR |
| | | | Insulin | NR |
| | | | Adiponectin | NR |
| | | | Fasting glucose | OGTT |
| | | | Glucose | OGTT |
| | General | Changes in BMI | Body Mass Index (BMI) | DXA and pedometers |
| | | Changes in BMI | Body fat | DXA and pedometers |
| | | Changes in BMI | Body weight | DXA and pedometers |
| | | Changes in BMI | Waist circumference | DXA and pedometers |
| | | Changes in BMI | Overweight prevalence | DXA and pedometers |
| | | Changes in BMI | Fat-free mass | DXA and pedometers |
| | | Changes in BMI | Parents outcomes | DXA and pedometers |
| | | Changes in BMI | Skinfold thickness | NR |
| | Global Quality of Life | | Health-related QoL | NR |
| | Metabolism and Nutrition | | Dietary behavior | NR |

| Study (Year) | Domain | Generic outcome (as stated by the authors) | Outcome | Outcome measure |
|---|---|---|---|---|
| | Physical Functioning | | Behavior change | NR |
| | | | Screen time | NR |
| | | | Physical activity level | NR |
| | Social Functioning | | Parental acceptance | NR |
| Mears and Jago., 2016 | Physical Functioning | Moderate-to-vigorous physical activity | Time spent in moderate to vigorous physical activity | Measured by accelerometers, heart rate (HR) monitor, self-report |
| Medina-Blanco et al., 2011 | Physical Functioning | Physical activity | Time spent in physical activity | Measure by accelerometer and pedometer (minutes, days and minutes per day) |
| Mei et al., 2016 | General | BMI | Body mass index (BMI) | kg/m² |
| Méndez-Hernández et al. (2022) | Blood and Lymphatic System | Blood markers | Glucose | NR |
| | | Blood markers | Total cholesterol | NR |
| | | Blood markers | Triglycerides | NR |
| | Cardiac | Cardiometabolic Factors | Diastolic blood pressure | NR |
| | | Cardiometabolic Factors | Systolic blood pressure | NR |
| | Endocrine | Blood markers | Adiponectin | NR |
| | | Blood markers | Insulin | NR |
| | | Blood markers | Insulin resistance | HOMA-IR |
| | | Blood markers | Leptin | NR |
| | General | Anthropometric characteristics | Body fat % | NR |
| | | Anthropometric characteristics | Body mass index (BMI) | NR |

| Study (Year) | Domain | Generic outcome (as stated by the authors) | Outcome | Outcome measure |
|---|---|---|---|---|
| | | Anthropometric characteristics | Fat-free mass | NR |
| | | Anthropometric characteristics | Waist circumference | NR |
| Menjie et al., 2022 | Blood and Lymphatic System | Outcome indexes: body shape indicators, CRF indicators and cardiovascular disease metabolic risk | High density lipoprotein (HDL) | NR |
| | | Outcome indexes: body shape indicators, CRF indicators and cardiovascular disease metabolic risk | Low density lipoprotein (LDL) | NR |
| | | Outcome indexes: body shape indicators, CRF indicators and cardiovascular disease metabolic risk | Total cholesterol | NR |
| | | Outcome indexes: body shape indicators, CRF indicators and cardiovascular disease metabolic risk | Triglycerides | NR |
| | Cardiac | Outcome indexes: body shape indicators, CRF indicators and | Diastolic blood pressure | NR |

| Study (Year) | Domain | Generic outcome (as stated by the authors) | Outcome | Outcome measure |
|---|---|---|---|---|
| | | cardiovascular disease metabolic risk | | |
| | | Outcome indexes: body shape indicators, CRF indicators and cardiovascular disease metabolic risk | Systolic blood pressure | NR |
| | | Outcome indexes: body shape indicators, CRF indicators and cardiovascular disease metabolic risk | HRmax | NR |
| | General | Outcome indexes: body shape indicators, CRF indicators and cardiovascular disease metabolic risk | Body mass index (BMI) | NR |
| | | Outcome indexes: body shape indicators, CRF indicators and cardiovascular disease metabolic risk | Percentage body fat | NR |
| | | Outcome indexes: body shape indicators, CRF indicators and cardiovascular disease metabolic risk | Waist circumference | NR |

| **Study (Year)** | **Domain** | **Generic outcome (as stated by the authors)** | **Outcome** | **Outcome measure** |
|---|---|---|---|---|
| | Physical Functioning | Outcome indexes: body shape indicators, CRF indicators and cardiovascular disease metabolic risk | VO2 | NR |
| Mijalkovi et al., 2022 | Cardiac | Cardiorespiratory Fitness | Blood Pressure | Automatic arterial blood pressure monitor (ABPM), Shuttle run test (SRT) |
| | | Cardiorespiratory Fitness | Global isovolumetric relaxation time | Comprehensive transthoracic echocardiography (CTE) |
| | | Cardiorespiratory Fitness | Heart rate | Step test (ST) |
| | | Cardiorespiratory Fitness | Heart rate reserve | Shuttle Run Test (SRT) |
| | | Cardiorespiratory Fitness | Left ventricular systolic ejection fraction | Comprehensive transthoracic echocardiography (CTE) |
| | | Cardiorespiratory Fitness | Rest heart rate | Shuttle Run Test (SRT) |
| | Physical Functioning | Cardiorespiratory Fitness | Excess post-exercise oxygen consumption (EPOC) | Treadmill protocol (TRE) |
| | | Cardiorespiratory Fitness | VO2max | 6 min walk test (6MWT) |
| | | Cardiorespiratory Fitness | VO2peak | Treadmill protocol (TRE) |
| Millard-Staffor et al., 2013 | Blood and Lymphatic System | Metabolic risk factor | Apolipoprotein A-1 | NR |
| | | Metabolic risk factor | Apolipoprotein B | NR |
| | | Cardiovascular risk factor | High density lipoprotein (HDL) | NR |
| | | Cardiovascular risk factor | Low density lipoprotein (LDL) | NR |

| **Study (Year)** | **Domain** | **Generic outcome (as stated by the authors)** | **Outcome** | **Outcome measure** |
|---|---|---|---|---|
| | | Cardiovascular risk factor | Serum C-reactive protein | NR |
| | | Cardiovascular risk factor | Total cholesterol | NR |
| | | Cardiovascular risk factor | Triglycerides | NR |
| | | Metabolic risk factor | Tumor Necrosis Factor-alpha | NR |
| | Cardiac | Blood pressure | Diastolic blood pressure | NR |
| | | Blood pressure | Systolic blood pressure | NR |
| | Cognitive Functioning | Mental/Emotional | Cognitive function | NR |
| | Endocrine | Metabolic risk factor | Adiponectin | NR |
| | | Metabolic risk factor | Fasting glucose | NR |
| | | Metabolic risk factor | Fasting insulin | NR |
| | | Metabolic risk factor | HOMA-index (Homeostatic Model Assessment for Insulin Resistance) | NR |
| | | Metabolic risk factor | IL-6 | NR |
| | | Metabolic risk factor | Insulin sensitivity | NR |
| | | Metabolic risk factor | Insulin-like Growth Factor 1 | NR |
| | | Metabolic risk factor | Leptin | NR |
| | General | Adiposity | Body fat mass (% or kg) | NR |
| | | Adiposity | Body fat-free mass | NR |
| | | Musculoskeletal health | Body fat-free mass | NR |
| | | Adiposity | Body mass index (BMI) | NR |

| **Study (Year)** | **Domain** | **Generic outcome (as stated by the authors)** | **Outcome** | **Outcome measure** |
|---|---|---|---|---|
| | | Adiposity | Visceral adipose tissue | NR |
| | | Adiposity | Waist circumference | NR |
| | Musculoskeletal and Connective Tissue | Musculoskeletal health | Bone density | NR |
| | | Musculoskeletal health | Bone mineral content | NR |
| | Physical Functioning | Aerobic fitness | Aerobic fitness test performance | Measured by distance run test or heart response (during/post) or VO2max direct-Bike or VO2max direct-Treadmill or VO2max estimate-Lab test or Walk test |
| | | Muscular strength | Maximum dynamic strength | Measured by 1RM - Lower Body or 1RM - Upper body |
| | | Muscular strength | Maximum isometric strength | Measured by handgrip dynamometer |
| | | Muscular strength | Muscle endurance | Measured by Curl-up or Sit-up or Push-up or Weighted Ball Throw |
| | | Muscular strength | Standing Broad Jump | NR |
| | Psychiatric | Mental/Emotional | Anxiety | NR |
| | | Mental/Emotional | Depression | NR |
| Nagle et al., 2013 | General | Obesity | Body fat mass (%) | NR |
| | | Obesity | Body mass index (BMI) | BMI, BMI-z score |
| | | Obesity | Body weight | NR |
| | | Obesity | Waist circumference | NR |
| Oliveira et al., 2017 | General | Body composition | Body fat mass (%) | Measured by dual energy X-ray absorptiometry or bioelectrical impedance analysis |
| | | Body composition | Body mass index (BMI) | NR |
| | | Body composition | Lean body mass | NR |

| Study (Year) | Domain | Generic outcome (as stated by the authors) | Outcome | Outcome measure |
|---|---|---|---|---|
| | | Body composition | Waist circumference | Measured by measuring tape |
| | Physical Functioning | Neuromuscular fitness | Agility test | NR |
| | | Flexibility | Flexibility | Sit-and-reach test |
| | | Cardiorespiratory endurance | Maximal power output (POmax) | Measured during maximal cardiopulmonary tests, on a cycle ergometer or on a treadmill |
| | | Muscle strength | Maximum isometric strength | Handgrip muscle strength |
| | | Cardiorespiratory endurance | Peak oxygen consumption (VO2 peak) | Measured during maximal cardiopulmonary tests, on a cycle ergometer or on a treadmill |
| Oosterhoff et al., 2016 | Cardiac | Blood pressure | Diastolic blood pressure | NR |
| | | Blood pressure | Systolic blood pressure | NR |
| | General | Body mass index | Body mass index (BMI) | BMI or, when BMI was not reported, BMI z-scores (i.e. BMI standardized for age and sex) |
| Paes et al., 2015 | Blood and Lymphatic System | Lipid profile | High density lipoprotein (HDL) | NR |
| | | Lipid profile | Low density lipoprotein (LDL) | NR |
| | | Inflammatory status | Proinflammatory cytokines | e.g. proinflammatory adipokine interleukin-6; oxidative metabolites of myeloperoxidase; serum leptin; adiponectin |
| | | Lipid profile | Triglycerides | NR |
| | Cardiac | Hemodynamic Response | Cardiac output | NR |
| | | Cardiometabolic Risk | Cardiometabolic risk | NR |
| | | Hemodynamic Response | Sympathetic Nervous Activity | NR |

| Study (Year) | Domain | Generic outcome (as stated by the authors) | Outcome | Outcome measure |
|---|---|---|---|---|
| | | Hemodynamic Response | Systolic blood pressure | NR |
| | Physical Functioning | Cardiorespiratory Fitness | Cardiorespiratory fitness | NR |
| | | Energy expenditure | Energy expenditure in physical activity | NR |
| | Vascular | Endothelial Dysfunction | Endothelial dysfunction | e.g. endothelial progenitor cells; endothelial vasodilator capacity |
| Parikh and Stratton et al., 2011 | General | Adiposity | Abdominal fat | NR |
| | | Adiposity | Body fat mass (%) | Measured by energy x-ray absorptiometry (DEXA), sum of skinfolds, adiposity equations or hydrostatic weighing |
| | | Adiposity | Body mass index (BMI) | NR |
| | | Adiposity | Waist circumference | NR |
| | Physical Functioning | Cardiorespiratory fitness | Maximal oxygen consumption (VO2max) | Measured by maximal tests, indirect calorimetry, time until exhaustion or endurance time, or a change in exercise HR |
| | | Cardiorespiratory fitness | Peak oxygen consumption (VO2 peak) | Measured by maximal tests, indirect calorimetry, time until exhaustion or endurance time, or a change in exercise HR |
| Recchia et al., 2023 | Psychiatric | depressive symptoms | Depressive symptoms | validated depression rating scale |
| Ribeiro et al., 2022 | Endocrine | Insulin sensitivity | Insulin sensitivity | NR |
| | General | Body composition | Body fat | kg |
| | | Body composition | Body mass index (BMI) | kg/m² |
| | | Body composition | Lean body mass | kg |
| | | Body composition | Waist circumference | cm |
| | Physical Functioning | Cardiorespiratory fitness | Cardiorespiratory fitness | NR |
| | | Muscle strength | Muscle strength | NR |

| **Study (Year)** | **Domain** | **Generic outcome (as stated by the authors)** | **Outcome** | **Outcome measure** |
|---|---|---|---|---|
| | | Cardiorespiratory fitness | VO2 max | NR |
| | | Cardiorespiratory fitness | VO2 peak | NR |
| Ruotsalainen et al., 2015 | Emotional functioning/wellbeing | Psychological symptoms | Attitudes about appearance | Measured by questionnaires |
| | | Psychological symptoms | Eating disorder or problematic eating attitudes | Measured by questionnaires |
| | | Psychological symptoms | Physical self-perception or body satisfaction | Measured by questionnaires |
| | | Psychological symptoms | Self-esteem | Measured by questionnaires |
| | General | Body mass index (BMI) | Body mass index (BMI) | BMI kg/m²; BMI z-scores |
| | Global Quality of Life | Psychological symptoms | Quality of life | Measured by questionnaires |
| | Physical Functioning | Physical activity | Physical activity level | Measured objectively using an accelerometer or self-reported |
| | | Physical activity | Physical activity playing active and non-active videogames | Measured by questionnaires |
| | | Physical activity | Sedentary activities | Sedentary activity self-reported |
| | Psychiatric | Psychological symptoms | Depression | Measured by questionnaires |
| Saavedra et al., 2011 | Physical Functioning | Aerobic fitness | Peak oxygen consumption (VO2 peak) | Determined by a maximal or submaximal effort test on a treadmill or cycloergometer, except for one study which gave no information on the method of evaluation |
| Sbruzzi et al., 2013 | Blood and Lymphatic System | Lipid profile | High density lipoprotein (HDL) | NR |

| Study (Year) | Domain | Generic outcome (as stated by the authors) | Outcome | Outcome measure |
|---|---|---|---|---|
| | | Lipid profile | Total cholesterol (mg/dL) | NR |
| | Cardiac | Blood pressure | Diastolic blood pressure | NR |
| | | Blood pressure | Systolic blood pressure | NR |
| | General | Anthropometric | Body mass index (BMI) | BMI kg/m² or BMI z-scores |
| | | Body composition | Body weight | NR |
| | | Anthropometric | Waist circumference (cm) | NR |
| Schranz et al., 2013 | Emotional Functioning/Wellbeing | Psychological well-being | Self-concept | Perception of physical ability and appearance |
| | | Psychological well-being | Self-efficacy | Confidence to complete a given task |
| | | Psychological well-being | Self-esteem | NR |
| | General | Body composition | Body fat mass (% and kg) | Body composition was assessed using two common methodologies by the included studies: anthropometric measures such as girths and skinfolds and dual energy X-ray absorptiometry |
| | | Body composition | Body mass index (BMI) | NR |
| | | Body composition | Lean body mass | Body composition was assessed using two common methodologies by the included studies: anthropometric measures such as girths and skinfolds and dual energy X-ray absorptiometry |
| | | Body composition | Waist circumference | Body composition was assessed using two common methodologies by the included studies: anthropometric measures such as girths and skinfolds and dual energy X-ray absorptiometry |
| | Global Quality of Life | Psychological well-being | Quality of life | Measured by Pediatric Quality of Life Inventory [PedsQL], |

| **Study (Year)** | **Domain** | **Generic outcome (as stated by the authors)** | **Outcome** | **Outcome measure** |
|---|---|---|---|---|
| | Physical Functioning | Strength | Maximum dynamic strength | 1RM for upper and lower body |
| | | Strength | Maximum isometric strength | Isometric knee extension or hand grip strength |
| Schwartz et al., 2017 | Blood and Lymphatic System | Metabolic parameters | High density lipoprotein (HDL) | NR |
| | | Metabolic parameters | Low density lipoprotein (LDL) | NR |
| | | Metabolic parameters | Total cholesterol | NR |
| | | Metabolic parameters | Triglycerides | NR |
| | Cardiac | Metabolic parameters | Diastolic blood pressure | NR |
| | | Metabolic parameters | Mean systolic and diastolic blood pressure | NR |
| | | Metabolic parameters | Systolic blood pressure | NR |
| | Endocrine | Appetite regulating hormones | Adiponectin | NR |
| | | Metabolic parameters | Fasting glucose | glucose concentration |
| | | Metabolic parameters | Fasting insulin | insulin concentration |
| | | Appetite regulating hormones | Leptin | NR |
| | | Appetite regulating hormones | Serum AgRP, NPY and α-MSH | NR |
| | General | Body composition | Body fat mass (%) | Body composition was assessed whether by using skin-folds or air-displacement plethysmography or biphotonic absorptiometry or bio-electrical impedance analyses |

| Study (Year) | Domain | Generic outcome (as stated by the authors) | Outcome | Outcome measure |
|---|---|---|---|---|
| | | Body composition | Body fat-free mass | Body composition was assessed whether by using skin-folds or air-displacement plethysmography or biphotonic absorptiometry or bio-electrical impedance analyses |
| | | Body composition | Body weight | Body composition was assessed whether by using skin-folds or air-displacement plethysmography or biphotonic absorptiometry or bio-electrical impedance analyses |
| | Metabolism and Nutrition | Energy and macronutrient intake | Dietary intake | Self-reported dietary records to assess total energy intake |
| | | Energy and macronutrient intake | Macronutrient intake | Self-reported dietary records to assess protein intake, fat intake and carbohydrate intake |
| Seral-Cortes et al., 2021 | General | Body composition | Body mass index (BMI) | NR |
| Shin et al., 2019 | General | Changes in measured BMI or body weight | Body mass index (BMI) | $kg/m^2$ |
| | | Changes in measured BMI or body weight | Body weight | kg |
| | Metabolism and Nutrition | Sugar‐sweetened beverage intake | Dietary behavior | Two items regarding consumption of SSBs; Schools Physical Activity and Nutrition Survey (SPANS); California Health Interview Survey (CHIS) questions |
| | Physical Functioning | Screen time | Sedentary activities | Adolescent Sedentary Activity Questionnaire (ASAQ), California Health Interview Survey (CHIS) questions, Physical Activity Questionnaire for Adolescents (PAQ‐A) |
| | | Physical activity | Time spent in physical activity, Days of physical activity per week | Actigraph accelerometers (counts/min), Physical activity days per week, Average daily activity counts (counts/min); |
| Silva et al., 2014 | Cardiac | Physical fitness | Maximum heart rate (beats/minute) | NR |

| Study (Year) | Domain | Generic outcome (as stated by the authors) | Outcome | Outcome measure |
|---|---|---|---|---|
| | General | Body composition | Abdominal fat | Measured by dual X-ray absorptiometry (DEXA) |
| | | Body composition | Body fat mass (%) | Measured by electrical bioimpedance or using dual X-ray absorptiometry (DEXA) |
| | | Body composition | Body fat-free mass | Measured by electrical bioimpedance or using dual X-ray absorptiometry (DEXA) |
| | | Body composition | Body mass index (BMI) | Measured by z-score de BMI or por el método de suavizado "Latent Moderated Structural" (LMS) or percentil de IMC a través de software; |
| | | Body composition | Body weight | NR |
| | | Body composition | Skinfold thickness | Subscapular and suprailiac skinfolds |
| | | Body composition | Waist circumference | NR |
| | Metabolism and Nutrition | Food and Diet | Dietary behavior | Behaviors related to healthy eating; social and emotional aspects related to obesity behavior (Eating Disorder Examination" (EDE-Q) questionnaire for the psychopathological evaluation of eating disorders; and) diagnostic interview "Eating Disorders" (IDed-IV); |
| | | Food and Diet | Dietary intake | Total energy intake; total carbohydrates intake; total fats intake; total sugar intake (measured by questionnaire) |
| | | Physical fitness | Cardiovascular efficiency | NR |
| | | Physical fitness | Global and physical motor abilities | NR |

Physical Functioning

| **Study (Year)** | **Domain** | **Generic outcome (as stated by the authors)** | **Outcome** | **Outcome measure** |
|---|---|---|---|---|
| | | Physical fitness | Maximal oxygen consumption (VO2max) | The following tests were used to assess the CF: a) test in cycle ergometer with gas analysis; b) test on a treadmill with gas analysis and determination of VO2max. with incremental workload (speed and incline) until exhaustion; c) application of the test "Course Navette" with modifications; d) analysis of the efficiency of cardiovascular system (VO2 / FC); e) assessment of the basal metabolic rate; f) submaximal field test; g) EUROFIT test battery; h) test up and down steps (3 minutes); i) motor skills test; j) Balke test, and k) walk for 6 minutes. |
| | | Physical fitness | Peak oxygen consumption (VO2 peak) | The following tests were used to assess the CF: a) test in cycle ergometer with gas analysis; b) test on a treadmill with gas analysis and determination of VO2max. with incremental workload (speed and incline) until exhaustion; c) application of the test "Course Navette" with modifications; d) analysis of the efficiency of cardiovascular system (VO2 / FC); e) assessment of the basal metabolic rate; f) submaximal field test; g) EUROFIT test battery; h) test up and down steps (3 minutes); i) motor skills test; j) Balke test, and k) walk for 6 minutes. |
| | | Physical activity | Healthy habits related with PA practice | Measured by “The Family Habit Inventory” and “Child Behaviour Checklist” |
| | | Physical activity | Physical activity level | e.g. PA in free time; AP practice moderate and intense; usual PA practice; time dedicated to the practice of PA (measured by questionnaire and accelerometer and pedometer) |
| | | Physical activity | Sedentary activities | Time devoted to sedentary activities and television (measured by questionnaire and accelerometer and pedometer) |

| **Study (Year)** | **Domain** | **Generic outcome (as stated by the authors)** | **Outcome** | **Outcome measure** |
|---|---|---|---|---|
| Sims et al., 2015 | Physical Functioning | Physical activity level | Time spent in moderate to vigorous physical activity | Objective measurement or validated self-report measure (accelerometers; questionnaire) |
| | | Physical activity level | Time spent in physical activity | Objective measurement or validated self-report measure (accelerometers; questionnaire) |
| Sirico et al., 2018 | Blood and Lymphatic System | Inflammatory marker level | Plasminogen activator inhibitor-1 | NR |
| | | Inflammatory marker level | Serum C-reactive protein | NR |
| | | Inflammatory marker level | Tumor Necrosis Factor-alpha | NR |
| | Endocrine | Inflammatory marker level | Adiponectin | NR |
| | | Inflammatory marker level | IL-6 | NR |
| | | Inflammatory marker level | Leptin | NR |
| | | Inflammatory marker level | Resistin levels | NR |
| Snethen et al., 2016 | General | Weight loss | Body weight | NR |
| Solera-Martinez et al., 2021 | Blood and Lymphatic System | Cardiometabolic | Glucose | NR |
| | | Cardiometabolic | High density lipoprotein (HDL) | NR |
| | | Cardiometabolic | Low density lipoprotein (LDL) | NR |
| | | Cardiometabolic | Non-High-density lipoproteins (non–HDL-C) | NR |
| | | Cardiometabolic | Total cholesterol | NR |
| | | Cardiometabolic | Triglycerides | NR |

| Study (Year) | Domain | Generic outcome (as stated by the authors) | Outcome | Outcome measure |
|---|---|---|---|---|
| | Cardiac | Blood Pressure | Diastolic blood pressure | NR |
| | | Blood Pressure | Systolic blood pressure | NR |
| | General | Body Composition | Body fat | NR |
| | | Body Composition | Body mass index (BMI) | NR |
| | Physical Functioning | Cardiorespiratory Fitness | VO2peak | NR |
| Spadaccini et al., 2022 | Blood and Lymphatic System | | Lipid profile | NR |
| | Cardiac | | Blood Pressure | NR |
| | | | Heart rate recovery | NR |
| | Emotional Functioning/Wellbeing | | Psychological disorders | NR |
| | | | Psychological well-being | NR |
| | Endocrine | | Adiponectin | NR |
| | | | C-reactive protein | NR |
| | | | Glycaemic profile | NR |
| | | | Leptin | NR |
| | | | Resistin levels | NR |
| | General | | Body circumferences | NR |
| | | | Body composition | NR |
| | | | body weight | NR |
| | | | Height | NR |
| | | | Parent report | NR |
| | | | skinfolds | NR |

| Study (Year) | Domain | Generic outcome (as stated by the authors) | Outcome | Outcome measure |
|---|---|---|---|---|
| | Global Quality of Life | | Quality of life | NR |
| | Metabolism and Nutrition | | Eating disorders | NR |
| | | | Food desire | NR |
| | | | Food intake | NR |
| | Physical Functioning | | Balance | NR |
| | | | fatigue | NR |
| | | | Muscle strength | NR |
| | | | physical performance | NR |
| George et al. (2022) | General | Body fat percentage | Body fat mass (%) | NR |
| | | BMI | Body mass index (BMI) | NR |
| | | Weight status | Body weight | NR |
| | | Waist circumference | Waist circumference | NR |
| | Metabolism and Nutrition | Sugar-sweetened beverage intake | Dietary behavior | NR |
| | | Fruit and vegetable intake | Portions of fruit, vegetables and water | NR |
| | Physical Functioning | Physical activity | Physical activity level | NR |
| | | Sedentary activities | Sedentary activities | NR |
| Staniford et al., 2011 | General | Weight status/adiposity | Body fat mass (%) | NR |
| | | Weight status/adiposity | Body mass index (BMI) | BMI or BMI-SDS or percent BMI |
| | | Weight status/adiposity | Body weight | NR |
| | | Weight status/adiposity | Percent overweight | NR |
| | | Weight status/adiposity | Skinfold thickness | NR |

| Study (Year) | Domain | Generic outcome (as stated by the authors) | Outcome | Outcome measure |
|---|---|---|---|---|
| | | Weight status/adiposity | Waist circumference | NR |
| Steele et al., 2021 | General | Body composition | Fat mass | kg |
| | | Body composition | Fat-free mass | kg |
| Stoner et al., 2016 | Blood and Lymphatic System | Cardiometabolic risk factors | High density lipoprotein (HDL) | NR |
| | | Cardiometabolic risk factors | Low density lipoprotein (LDL) | NR |
| | | Cardiometabolic risk factors | Total cholesterol | NR |
| | | Cardiometabolic risk factors | Triglycerides | NR |
| | Cardiac | Cardiometabolic risk factors | Systolic blood pressure | NR |
| | Endocrine | Cardiometabolic risk factors | Area under the curve for glucose | NR |
| | | Cardiometabolic risk factors | Fasting glucose | NR |
| | | Cardiometabolic risk factors | Fasting insulin | NR |
| | | Cardiometabolic risk factors | HOMA-index (Homeostatic Model Assessment for Insulin Resistance) | NR |
| | General | Body composition | Body fat mass (%) | Measured by DXA or skinfolds or hydrostatic |
| | | Anthropometric | Body mass index (BMI) | BMI ($kg/m^2$) |
| | | Body composition | Lean body mass | Measured by DXA or skinfolds or hydrostatic |
| Thivel et al., 2018 | Blood and Lymphatic System | Cardiometabolic factors | Glucose | NR |
| | | Cardiometabolic factors | High density lipoprotein (HDL) | NR |

| Study (Year) | Domain | Generic outcome (as stated by the authors) | Outcome | Outcome measure |
|---|---|---|---|---|
| | | Cardiometabolic factors | Low density lipoprotein (LDL) | NR |
| | | Cardiometabolic factors | Total cholesterol | NR |
| | | Cardiometabolic factors | Triglycerides | NR |
| | Cardiac | Blood Pressure | Diastolic blood pressure | NR |
| | | Blood Pressure | Systolic blood pressure | NR |
| | Endocrine | Metabolic profile | Adiponectin | NR |
| | | Metabolic profile | HbA1c | NR |
| | | Metabolic profile | Insulin | NR |
| | | Metabolic profile | Insulin resistance | HOMA-IR |
| | | Metabolic profile | Leptin | NR |
| | | Metabolic profile | NO2- | NR |
| | General | Anthropometric characteristics and Body composition | Body fat % | Dual X-ray Absorptiometry was used in 2 studies . Bio-impedance analysis in 7 studies. Skinfold thickness measurement in 3 studies. Air displacement plethysmography (Bod Pod) is one study. Both BIA and skinfold thickness measurement were used. |
| | | Anthropometric characteristics and Body composition | Body Mass | Dual X-ray Absorptiometry was used in 2 studies . Bio-impedance analysis in 7 studies. Skinfold thickness measurement in 3 studies. Air displacement plethysmography (Bod Pod) is one study. Both BIA and skinfold thickness measurement were used. |
| | | Anthropometric characteristics and Body composition | Body mass index (BMI) | Dual X-ray Absorptiometry was used in 2 studies . Bio-impedance analysis in 7 studies. Skinfold thickness measurement in 3 studies. Air displacement |

| Study (Year) | Domain | Generic outcome (as stated by the authors) | Outcome | Outcome measure |
|---|---|---|---|---|
| | | | | plethysmography (Bod Pod) is one study. Both BIA and skinfold thickness measurement were used. |
| | | Anthropometric characteristics and Body composition | Fat-free mass | Dual X-ray Absorptiometry was used in 2 studies . Bio-impedance analysis in 7 studies. Skinfold thickness measurement in 3 studies. Air displacement plethysmography (Bod Pod) is one study. Both BIA and skinfold thickness measurement were used. |
| | | Anthropometric characteristics and Body composition | Waist Circumf. | Dual X-ray Absorptiometry was used in 2 studies . Bio-impedance analysis in 7 studies. Skinfold thickness measurement in 3 studies. Air displacement plethysmography (Bod Pod) is one study. Both BIA and skinfold thickness measurement were used. |
| | | Anthropometric characteristics and Body composition | z-score BMI | Dual X-ray Absorptiometry was used in 2 studies . Bio-impedance analysis in 7 studies. Skinfold thickness measurement in 3 studies. Air displacement plethysmography (Bod Pod) is one study. Both BIA and skinfold thickness measurement were used. |
| | Metabolism and Nutrition | Energy intake | Energy intake | Dietary recall (4-day questionnaire) |
| | Physical Functioning | Other fitness parameters | Countermovement jump | Countermovement Jump |
| | | Other fitness parameters | Maximal Leg Strength | NR |
| | | Other fitness parameters | Squat Jump | Squat Jump |
| | | Aerobic fitness | CHO oxidation | 14 studies used an incremental maximal test performed in the laboratory with measurement of maximal oxygen uptake (VO2max). YoYo intermittent field test was used in one study. The Astrand cycling test was used in one study. A 6 minutes maximal running test was used in 2 studies. |

| Study (Year) | Domain | Generic outcome (as stated by the authors) | Outcome | Outcome measure |
|---|---|---|---|---|
| | | Aerobic fitness | HRmax | 13 studies used an incremental maximal test performed in the laboratory with measurement of maximal oxygen uptake (VO2max). YoYo intermittent field test was used in one study. The Astrand cycling test was used in one study. A 6 minutes maximal running test was used in 2 studies. |
| | | Aerobic fitness | Max Aerob Sp | 16 studies used an incremental maximal test performed in the laboratory with measurement of maximal oxygen uptake (VO2max). YoYo intermittent field test was used in one study. The Astrand cycling test was used in one study. A 6 minutes maximal running test was used in 2 studies. |
| | | Aerobic fitness | RER | 15 studies used an incremental maximal test performed in the laboratory with measurement of maximal oxygen uptake (VO2max). YoYo intermittent field test was used in one study. The Astrand cycling test was used in one study. A 6 minutes maximal running test was used in 2 studies. |
| | | Aerobic fitness | VO2max | 11 studies used an incremental maximal test performed in the laboratory with measurement of maximal oxygen uptake (VO2max). YoYo intermittent field test was used in one study. The Astrand cycling test was used in one study. A 6 minutes maximal running test was used in 2 studies. |
| | | Aerobic fitness | VO2peak | 12 studies used an incremental maximal test performed in the laboratory with measurement of maximal oxygen uptake (VO2max). YoYo intermittent field test was used in one study. The Astrand cycling test was used in one study. A 6 minutes maximal running test was used in 2 studies. |
| | | Aerobic fitness | ΔHRR1 | 17 studies used an incremental maximal test performed in the laboratory with measurement of maximal oxygen uptake (VO2max). YoYo intermittent field test was used in one |

| Study (Year) | Domain | Generic outcome (as stated by the authors) | Outcome | Outcome measure |
|---|---|---|---|---|
| | | | | study. The Astrand cycling test was used in one study. A 6 minutes maximal running test was used in 2 studies. |
| | | Aerobic fitness | ΔHRR2 | 18 studies used an incremental maximal test performed in the laboratory with measurement of maximal oxygen uptake (VO2max). YoYo intermittent field test was used in one study. The Astrand cycling test was used in one study. A 6 minutes maximal running test was used in 2 studies. |
| | Vascular | Endothelial Function | Endothelial function | Flow-mediated dilatation |
| Upton et al., 2014 | General | Weight change | Body mass index (BMI) | BMI; BMI percentile; BMI z-score/SDS |
| | | Weight change | Body weight | NR |
| Valeriani et al., 2021 | General | Weight status | Body mass index (BMI) | NR |
| | | Weight status | zBMI | NR |
| van Wijnen et al., 2009 | Cognitive Functioning | Psychosocial | Cognitive competence (self-perception) | Measured by Harter-Perceived Competence Scale for Children (Harter, 1982) |
| | Delivery of Care | Psychosocial | Unhealthy weight control behaviours (self-perception) | List of nine unhealthy and five health weight control methods: respondents indicate use of each method over past month (yes/no) |
| | Emotional functioning/wellbeing | Psychosocial | Athletic competence | Measured by Perceived Competence Scale for Children |
| | | Psychosocial | Binge eating (self-perception) | One question: ‘In the past month, have you ever eaten so much food in such a short period of time that you would be embarrassed if others saw you (binge eating)? |
| | | Psychosocial | Body image (self-perception) | Satisfaction with body image measure by adapted body shape perception scale; Body image perception measure by Figural Rating Scale (4th–7th grades; Stunkard et al., 1983) & Schematic Figures for kindergarten–3rd grade (Collins, 1991) |

| Study (Year) | Domain | Generic outcome (as stated by the authors) | Outcome | Outcome measure |
|---|---|---|---|---|
| | | Psychosocial | Healthy attitudes (self-perception) | e.g. Dietary restraint, Disordered eating |
| | | Psychosocial | Media internalization (self-perception) | Four questions assessing internalization of media ideals for appearance/body shape (Heinberg et al., 1995; Neumark-Sztainer et al., 2000) |
| | | Psychosocial | Self-acceptance (self-perception) | Measured by Perceived Competence Scale for Children (Harter, 1982) |
| | | Psychosocial | Self-esteem (self-perception) | NR |
| | | Psychosocial | Self-worth (self-perception) | Global or general self-worth measured by Harter-Perceived Competence Scale for Children |
| | Social Functioning | Psychosocial | Aggression (self-perception) | Peer-rated aggression measure by fifteen questions about the behaviour of classmates including aggressive behaviour items, popularity items and prosocial items (Eron et al., 1971; Walder et al., 1961); Observed physical and verbal aggression measured by direct observation during free play, using the protocol by Joy et al. (1986); Parent report of aggressive and delinquent behaviour measure by Parent report form of the Child Behaviour Checklist (Achenbach, 1991); Perceptions of a mean and scary world |
| | | Psychosocial | Social acceptance (self-perception) | Measured by Perceived Competence Scale for Children (Harter, 1982) |
| Vasconcellos et al., 2014 | Blood and Lymphatic System | Inflammatory profile | Fibrinogen level | NR |
| | | Biochemical markers | High density lipoprotein (HDL) | NR |
| | | Biochemical markers | Low density lipoprotein (LDL) | NR |

| Study (Year) | Domain | Generic outcome (as stated by the authors) | Outcome | Outcome measure |
|---|---|---|---|---|
| | | Inflammatory profile | Serum C-reactive protein | NR |
| | | Biochemical markers | Total cholesterol | NR |
| | Cardiac | Hemodynamic | Diastolic blood pressure | NR |
| | | Hemodynamic | Resting heart rate (beats/minute) and Maximum heart rate (beats/minute) | NR |
| | | Hemodynamic | Systolic blood pressure | NR |
| | Endocrine | Inflammatory profile | Adiponectin | NR |
| | | Biochemical markers | Fasting glucose | NR |
| | | Biochemical markers | Fasting insulin | NR |
| | | Inflammatory profile | IL-6 | NR |
| | General | Body composition | Body fat mass (%) | NR |
| | | Body composition | Body mass index (BMI) | NR |
| | | Body composition | Waist circumference | NR |
| | Physical Functioning | Cardiorespiratory fitness | Cardiorespiratory fitness | NR |
| | | Muscle strength | Muscle strength | NR |
| | Vascular | Endothelial function | Flow-mediated dilation (FMD) | NR |
| Verjans-Janssen et al., 2018 | General | BMI | Body mass index (BMI) | BMI measured by z-score or percentile for age and gender |

| Study (Year) | Domain | Generic outcome (as stated by the authors) | Outcome | Outcome measure |
|---|---|---|---|---|
| | Metabolism and Nutrition | Nutrition behavior | Dietary intake | Fruit intake (exchanges/day or portions/day); Vegetable intake (exchanges/day or portions/day); Dairy intake (exchanges/day); Fats and oils intake (exchanges/day); Meat intake (exchanges/day); Grains intake (exchanges/day); Sweets and beverages intake (exchanges/day); Sugar-sweetened beverages intake (portions/day); Soda intake (portions/day); Chocolate and candy intake (portions/day); Snack intake (portions/day); Water consumption (servings/day); Saturated fat intake (mg/weekday); Total fat (g/day); Cholesterol (g/day); Protein (g/day); Carbohydrate (g/day); Fiber (g/day) |
| | Physical Functioning | Sedentary behavior | Sedentary activities | Sitting (h/day); TV watching (h/day); Computer and video games (h/day); Sedentary behavior (hours of screen viewing/day); TV viewing (frequency of TV viewing while getting ready for school; Time spent sedentary (min/day); TV viewing week day / weekend (% children watching TV 2 hours or less per week day / weekend) |
| | | Physical activity | Time spent in physical activity | MVPA (min/day); Outdoor play (h/day); Physical education (h/week); Supervised sports or dancing (h/week); Time spent in outdoor activities (h/week); Time spent in extracurricular sports courses (h/week); PA behavior (PA behavior compared to other children of same age and sex); Team sports; Light PA (min/day); Leisure-time MVPA (h/week); PA (% Children performing daily PA); Active > 60 min (days/week); Active games at lunchtime (% children performing active games at lunchtime); Being outside yesterday (% children being outside 2 hours or more after school yesterday); Being outside weekend day (% children being outside 2 hours or more on a weekend day); Jogging/running frequency (% children with improved |

| Study (Year) | Domain | Generic outcome (as stated by the authors) | Outcome | Outcome measure |
|---|---|---|---|---|
| | | | | frequency); Walking frequency (% children with improved frequency); Ball playing (% children improved frequency); Walking or riding bicycles to school (% children improved frequency) |
| Vissers et al., 2016 | General | Visceral adipose tissue | Visceral adipose tissue (VAT) | VAT assessed by medical imaging (computed tomography, magnetic resonance imaging, ultrasound) |
| Voskuil et al., 2017 | General | Body composition | Body fat mass (%) | Bioelectrical impedance analysis, dual-energy x-ray absorptiometry and estimation of % body fat from skin fold measurements |
| | | Body composition | Body mass index (BMI) | NR |
| | Physical Functioning | Accelerometer-measured physical activity | Physical activity level | Accelerometer-measured moderate-to-vigorous physical activity |
| Wang et al., 2022 | General | Visceral Fat | visceral adipose tissue | NR |
| Whitlock et al., 2010 | General | Weight change | Body mass index (BMI) | Change in BMI from baseline as the preferred measure of weight change when it was available. If BMI change was unavailable and could not be calculated or obtained from the author, we used change in BMI standard deviation score (SDS) as our second choice and change in percent overweight as the third choice. |
| Wolfa et al., 2019 | General | Obesity | Body mass index (BMI) | NR |
| | | Obesity | Waist Circumference | NR |
| Xu et al., 2022 | General | weight loss | body fat mass | NR |
| | | weight loss | Body mass index (BMI) | NR |
| | | weight loss | body weight | NR |
| Yukel et al., 2020 | Cardiac | Cardiovascular risk | Cardiovascular risk | An automated oscillograph |

| Study (Year) | Domain | Generic outcome (as stated by the authors) | Outcome | Outcome measure |
|---|---|---|---|---|
| | Emotional Functioning/Wellbeing | Healthy lifestyle | Healthy lifestyle | A self-administered non-quantitative FFQ |
| | | Quality of life | Quality of life | Health questionnaire |
| | General | Body fat percentage | Body fat percentage | Percentage of body fat was calculated from triceps and subscapular skinfolds measurements |
| | | Obesity | Body mass index (BMI) | kg/m² - Tanita BC 418MA Segmental Body Composition Analyzer |
| | | Obesity | Height | Seca Leicester Height Measure stadiometer, stadiometer, portable stadiometer, standing stadiometer (Seca model 720, Germany). |
| | | Obesity | Skinfold | mm - Harpenden calipers (HSK BI, British Indicators) |
| | | Obesity | Waist circumference | A measuring tape between the lower rib and the iliac-crest; Flexible tape |
| | | Obesity | Weight | SecaTM 899 digital scales, portable digital scale, lever scale |
| | Metabolism and Nutrition | Daily fruit and vegetable consumption | Daily fruit and vegetable consumption | A Day in the Life Questionnaire |
| | | Diet | Diet | Self-completed questionnaires |
| | Physical Functioning | Physical Fitness | Aerobic fitness | 20m shuttle run |
| | | Physical Fitness | Cardiorespiratory fitness | Monark ergometer bike (W/kg), 20m shuttle run |
| | | Physical Fitness | Peak power | calibrated friction loaded ergometer. |
| | | Physical Activity | Physical activity level | Actigraph, pedometer, questionnaire, accelerometer, CLASS questionnaire |
| | | Physical Fitness | Physical fitness | 1 min of sit‑ups, 1 min of push‑ups, a measured vertical jump, a measured long jump, a stand-and‑reach test, a timed 30‑m sprint, 20m shuttle run, 1 mile run, sit and reach, curl-ups, accelerometer |

| **Study (Year)** | **Domain** | **Generic outcome (as stated by the authors)** | **Outcome** | **Outcome measure** |
|---|---|---|---|---|
| | | Sedentary behaviour | Sedentary behaviour | Accelerometer |
| Zabatiero et al., 2018 | General | Sedentary behaviour | Body mass index (BMI) | NR |
| | Global Quality of Life | Sedentary behaviour | Health-related QOL | Pediatric QOL Inventory |
| | Physical Functioning | Sedentary behaviour | sedentary time | accelerometry and inclinometry |
| | | Sedentary behaviour | time spent in MVPA | accelerometry and inclinometry |
| Zhao et al., 2022-a | Endocrine | Inflammatory Response | C-reactive protein | NR |
| | | Inflammatory Response | IL-6 | NR |
| | | Inflammatory Response | TNF-α | NR |
| Zhao et al., 2022-b | Blood and Lymphatic System | Cardiometabolic Factors | Glucose | NR |
| | | Cardiometabolic Factors | High density lipoprotein (HDL) | NR |
| | | Cardiometabolic factors | Low density lipoprotein (LDL) | NR |
| | | Cardiometabolic Factors | Non-High-density lipoproteins (non–HDL-C) | NR |
| | | Inflammatory Markers | TNF-alfa | NR |
| | | Cardiometabolic Factors | Total cholesterol | NR |
| | | Cardiometabolic Factors | Triglycerides | NR |
| | Endocrine | Inflammatory Markers | C-reactive protein | NR |
| | | Cardiometabolic Factors | Adiponectin | NR |
| | | Cardiometabolic Factors | Chemerin | NR |
| | | Cardiometabolic Factors | IGF-1 | NR |

| Study (Year) | Domain | Generic outcome (as stated by the authors) | Outcome | Outcome measure |
|---|---|---|---|---|
| | | Cardiometabolic Factors | IGFBP-3 | NR |
| | | Inflammatory Markers | IL-6 | NR |
| | | Cardiometabolic Factors | Insulin resistance | HOMA-IR |
| | | Cardiometabolic Factors | Leptin | NR |
| | General | Anthropometric | Biceps circumference | NR |
| | | Anthropometric | Body mass index (BMI) | NR |
| | | Anthropometric | Body weight | NR |
| | | Anthropometric | Fat mass | NR |
| | | Anthropometric | Forearm circumference | NR |
| | | Anthropometric | Hip circumference | NR |
| | | Anthropometric | Limbs circumference | NR |
| | | Anthropometric | thigh circumference | NR |
| | | Anthropometric | torso circumference | NR |
| | | Anthropometric | Waist circumference | NR |
| | | Anthropometric | Waist circumference | NR |
| | | Anthropometric | waist/hip ratio | NR |
| Zhu et al., 2021 | General | obesity | Body fat percentage | NR |
| | | obesity | Body mass index (BMI) | NR |
| | | obesity | Body weight | NR |
| | | obesity | Waist circumference | NR |
| Zouhal et al., 2020 | Cardiac | Physical performance | HRmax | NR |

| Study (Year) | Domain | Generic outcome (as stated by the authors) | Outcome | Outcome measure |
|---|---|---|---|---|
| | General | Anthropometric characteristics | Body fat % | NR |
| | | Anthropometric characteristics | Body mass index (BMI) | NR |
| | | Anthropometric characteristics | Body weight | NR |
| | | Anthropometric characteristics | Fat-free mass | NR |
| | Physical Functioning | Physical performance | Lactate threshold | NR |
| | | Physical performance | Maximal strength | NR |
| | | Physical performance | Mean power | NR |
| | | Physical performance | Peak power | NR |
| | | Physical performance | Running economy | NR |
| | | Physical performance | Sprint performance | NR |
| | | Physical performance | Ventilatory threshold | NR |
| | | Physical performance | VO2max | NR |
| Liu et al., 2024 | Cardiac | Blood pressure | Blood pressure | NR |
| | General | Body composition | BMI | NR |
| | | Body composition | BMI-z | NR |
| | | Body composition | Fat mass | NR |
| | | Body composition | Body fat % | NR |
| | Physical Functioning | Cardiorespiratory fitness | VO2max | NR |
| | | | Muscular strength | NR |

| Study (Year) | Domain | Generic outcome (as stated by the authors) | Outcome | Outcome measure |
|---|---|---|---|---|
| Dos Santos et al., 2021 | Blood and Lymphatic System | | Total cholesterol | NR |
| | | | Low density lipoprotein (LDL) | NR |
| | Cardiac | | Resting heart rate and Maximum heart rate | NR |
| | Endocrine | | Fasting blood glucose | NR |
| | Physical Functioning | | Time spent in physical activity | NR |
| | | | Daily sedentary time | NR |
| García-Hermoso et al., 2020-c | Cardiac | | Systolic blood pressure | NR |
| | | | Diastolic blood pressure | NR |
| | General | | Body mass index (BMI) | NR |
| | | | Waist circumference | NR |
| | | | Body fat | NR |
| | | | Fat-free mass | NR |
| | Physical Functioning | | Agility | NR |
| Da Silva et al., 2020 | Endocrine | Plasma adipokines | Adiponectin | NR |
| | | Plasma adipokines | Leptin | NR |
| | | Plasma adipokines | Adipocyte fat (aFABP) | NR |
| Comeras-Chueca et al., 2021 | General | Health-related physical fitness | Body mass index (BMI) | dual-energy x-ray absorptiometry or bioelectrical impedance |
| | | Health-related physical fitness | Body weight | dual-energy x-ray absorptiometry or bioelectrical impedance |

| Study (Year) | Domain | Generic outcome (as stated by the authors) | Outcome | Outcome measure |
|---|---|---|---|---|
| | | Health-related physical fitness | Body fat % | dual-energy x-ray absorptiometry or bioelectrical impedance |
| | | Health-related physical fitness | Free-fat mass | dual-energy x-ray absorptiometry or bioelectrical impedance |
| | | Health-related physical fitness | Waist circumference | dual-energy x-ray absorptiometry or bioelectrical impedance |
| | Physical Functioning | Health-related physical fitness | Cardiorespiratory fitness | 20-m shuttle run test, 3-min step test or submaximal test with a cycle ergometer |
| | | Health-related physical fitness | Muscular fitness | 20-m shuttle run test, 3-min step test or submaximal test with a cycle ergometer |
| | | Motor competence | Motor competence | Bruininks-Oseretsky Test and Movement Assessment Battery for Children Test-Second Edition |
| Liu et al., 2020 | Blood and Lymphatic System | Cardiometabolic risk factors | Total cholesterol | NR |
| | | Cardiometabolic risk factors | High density lipoprotein (HDL) | NR |
| | | Cardiometabolic risk factors | Low density lipoprotein (LDL) | NR |
| | Cardiac | Cardiometabolic risk factors | Systolic blood pressure | NR |
| | | Cardiometabolic risk factors | Diastolic blood pressure | NR |
| | Endocrine | Cardiometabolic risk factors | Fasting blood glucose | NR |
| | | Cardiometabolic risk factors | Fasting insulin | NR |
| | | Cardiometabolic risk factors | HOMA-index (Homeostatic Model Assessment for Insulin Resistance) | NR |

| Study (Year) | Domain | Generic outcome (as stated by the authors) | Outcome | Outcome measure |
|---|---|---|---|---|
| | General | Cardiometabolic risk factors | Body mass index (BMI) | NR |
| | | Cardiometabolic risk factors | Body weight | NR |
| | | Cardiometabolic risk factors | Waist circumference | NR |
| | Physical Functioning | Cardiometabolic risk factors | VO2peak | NR |

NR = not reported; TNF-α – Tumor Necrosis Factor-alpha; ΔHRR1 – Change in Heart Rate Recovery 1 (first-minute heart rate recovery); ΔHRR2 – Change in Heart Rate Recovery 2 (second-minute heart rate recovery); IL-6 – Interleukin-6; PWV – Pulse Wave Velocity; IMT – Intima-Media Thickness; LF – Low Frequency (component of heart rate variability); LF/HR – Low Frequency to Heart Rate Ratio; Mean RR – Mean R-R Interval (average time between heartbeats); RMSSD – Root Mean Square of the Successive Differences (a measure of heart rate variability); SD1 – Standard Deviation 1 (short-term heart rate variability measure from Poincaré plot); SD2 – Standard Deviation 2 (long-term heart rate variability measure from Poincaré plot) SDNN – Standard Deviation of Normal-to-Normal Intervals (global measure of heart rate variability); HbA1c – Hemoglobin A1c (glycated hemoglobin, a marker of long-term blood glucose levels); MVPA – Moderate-to-Vigorous Physical Activity; PNN50 – Percentage of Normal-to-Normal Intervals Greater than 50 ms (heart rate variability metric); RMSSD – Root Mean Square of the Successive Differences (a measure of heart rate variability); DXA – Dual-Energy X-ray Absorptiometry (a technique for measuring body composition and bone density); AgRP – Agouti-Related Peptide (a neuropeptide involved in appetite regulation); NPY – Neuropeptide Y (a peptide involved in energy balance and appetite control); α-MSH – Alpha-Melanocyte-Stimulating Hormone (a peptide hormone involved in energy homeostasis and pigmentation); NO2 – Nitrogen Dioxide.

**Table S4. List of clinical trials included in all 137 reviews.**

| Trial 1st author | Trial DOI | Trial year | Sample Size |
|---|---|---|---|
| Martinez-Viscaiano | 10.1111/sms.14113 | 2022 | 562 |
| Smout | 10.1186/s12955-021-01907-5 | 2022 | 100 |
| Cao | 10.3390/jcm11185436 | 2022 | 40 |
| Cao | 10.1186/s12887-021-03079-z | 2022 | 36 |
| Seo | 10.1371/journal.pone.0245875 | 2021 | 242 |
| Zhang | NR | 2021 | 138 |
| Zhang | 10.1111/sms.13803 | 2021 | 59 |
| Irandoust | PMID: 33176381 | 2021 | 59 |
| Bogataj | 10.3390/nu13010238 | 2021 | 48 |
| Leandro | 10.23736/S0022-4707.20.11648-7 | 2021 | 41 |
| Mohammed | 10.1111/pedi.13203 | 2021 | 40 |
| Rasooli | 10.1139/apnm-2020-0171 | 2021 | 40 |
| Paahoo | 10.1123/pes.2020-0138 | 2021 | 30 |
| Vasconcellos | 10.1080/02701367.2019.1711007 | 2021 | 13 |
| Rigamonti | 10.3390/nu12010208 | 2020 | 644 |
| Prado | 10.1016/j.amepre.2020.06.010 | 2020 | 280 |
| Yu | 10.3390/nu12010194 | 2020 | 171 |
| Perez-Sousa | 10.1080/02701367.2019.1645939 | 2020 | 170 |
| Romero-Perez | 10.3390/ijerph17134655 | 2020 | 105 |
| Aguilar-Cordero | 10.1093/ajh/hpz174 | 2020 | 98 |
| Liang | 10.3390/ijerph17217984 | 2020 | 87 |
| Kim | 10.4162/nrp.2020.14.3.262 | 2020 | 66 |
| Ceballos-Gurrola | 10.47197/retos.v38i38.77003 | 2020 | 62 |
| Lee | 10.1139/apnm-2019-0993 | 2020 | 60 |
| Da Silva | 10.1016/j.physbeh.2019.112728 | 2020 | 50 |
| Kim | 10.1515/jpem-2019-0327 | 2020 | 48 |
| Lopez-Alarcon | 10.1530/EC-19-0461 | 2020 | 45 |
| Pahoo | 10.5812/ijp.99760 | 2020 | 45 |
| Plavsic | 10.1139/apnm-2019-0137 | 2020 | 44 |
| Miguet | 10.1139/apnm-2019-0160 | 2020 | 43 |
| Abassi | 10.1515/hmbci-2020-0031 | 2020 | 43 |
| Seabra | 10.1016/j.pcad.2020.07.007 | 2020 | 40 |
| Duft | 10.1038/s41598-020-73943-y | 2020 | 37 |
| Brand | 10.1080/02640414.2020.1725384 | 2020 | 35 |
| Brasil | 10.1080/02640414.2020.1792189 | 2020 | 35 |

| Trial 1st author | Trial DOI | Trial year | Sample Size |
|---|---|---|---|
| Silva | 10.1080/09603123.2019.1612041 | 2020 | 33 |
| Johansson | 10.1186/s12887-020-02338-9 | 2020 | 28 |
| Roh | 10.3390/ijerph17072505 | 2020 | 20 |
| Qin | ANF | 2020 | |
| Taiyang | 10.1371/journal.pone.0271845 | 2019 | 2995 |
| Learmonth | 10.1038/s41366-018-0300-1 | 2019 | 1009 |
| Olive | 10.1037/edu0000338 | 2019 | 821 |
| Sadeghi | 10.1111/ijpo.12135 | 2019 | 782 |
| Sharma | 10.1089/chi.2018.0010 | 2019 | 672 |
| Zask | 10.1071/he12010 | 2019 | 560 |
| Byrnes | 10.1037/adb0000442 | 2019 | 411 |
| Lopez-Alarcon | 10.1111/ijpo.12499 | 2019 | 366 |
| Espinoza-Silva | 10.1016/j.endinu.2019.05.005 | 2019 | 274 |
| Warshburger | 10.3390/nu11092053 | 2019 | 266 |
| Hao | 10.1177/1010539519848275 | 2019 | 229 |
| Davis | 10.1038/s41366-019-0482-1 | 2019 | 175 |
| Williams | 10.1093/tbm/ibz015 | 2019 | 175 |
| Dalziell | 10.1002/berj.3514 | 2019 | 143 |
| Hacke | 10.1111/sms.13390 | 2019 | 135 |
| Coknaz | 10.1007/s00431-019-03457-x | 2019 | 106 |
| Seo | 10.3390/nu11010137 | 2019 | 103 |
| Haghshenas | NR | 2019 | 100 |
| Djaafar | 10.31838/ijpr/2019.11.02.043 | 2019 | 98 |
| Ye | 10.3390/ijerph16214080 | 2019 | 81 |
| Farpour-Lambert | 10.1111/cob.12335 | 2019 | 74 |
| Garza | 10.3390/ijerph16245133 | 2019 | 71 |
| Soltero | 10.1016/j.amepre.2018.07.034 | 2019 | 68 |
| Leahy | 10.1123/pes.2018-0039 | 2019 | 68 |
| Gao | 10.1016/j.jshs.2018.12.001 | 2019 | 65 |
| Xiong | 10.3390/jcm8040469 | 2019 | 60 |
| McGann | 10.3390/ijerph191911914 | 2019 | 54 |
| Bonney | PMID: 31568378 | 2019 | 52 |
| Horsak | 10.1016/j.gaitpost.2019.02.032 | 2019 | 51 |
| Jensen | 10.1037/cpp0000268 | 2019 | 47 |
| Xiang | 10.3389/fpsyg.2019.01385 | 2019 | 44 |
| Sung | 10.1007/s00421-018-4051-4 | 2019 | 40 |

| Trial 1st author | Trial DOI | Trial year | Sample Size |
|---|---|---|---|
| Chen | 10.1016/j.jadohealth.2018.08.022 | 2019 | 40 |
| Alves | 10.1590/0001-3765201920181264 | 2019 | 40 |
| Moslehi | 10.1016/j.obmed.2019.100104 | 2019 | 30 |
| Alonso-Fernández | 10.1016/j.scispo.2019.04.001 | 2019 | 26 |
| Huang | 10.2147/DMSO.S223514 | 2019 | 21 |
| Parsons | 10.1111/jspn.12235 | 2019 | 11 |
| Waters | 10.1186/s12889-017-4625-9 | 2018 | 2965 |
| Messiah | 10.1007/s10900-017-0393-9 | 2018 | 2261 |
| D'Agostino | 10.1016/j.puhe.2018.02.025 | 2018 | 1546 |
| Martínez-Vizcaíno | 10.1136/bjsports-2018-099655 | 2018 | 741 |
| Hoor | 10.1186/s12966-018-0727-8 | 2018 | 695 |
| Barkin | 10.1001/jama.2018.9128 | 2018 | 610 |
| French | 10.2105/AJPH.2018.304696 | 2018 | 534 |
| Sherwood | 10.1111/ijpo.12523 | 2018 | 421 |
| Aperman‐Itzhak | 10.1080/19325037.2018.1486755 | 2018 | 396 |
| Wylie-Rosett | 10.1186/s12966-017-0639-z | 2018 | 365 |
| Hull | 10.1111/ijpo.12197 | 2018 | 272 |
| Lucas | 10.1016/j.puhe.2018.02.014 | 2018 | 232 |
| Goldfield | 10.1016/j.physbeh.2018.04.026 | 2018 | 228 |
| Delgado-Floody | 10.1007/s00431-018-3149-3 | 2018 | 197 |
| Ketelhut | 10.1007/s11332-018-0463-0. | 2018 | 160 |
| Leeuwen | 10.1093/fampra/cmy061 | 2018 | 154 |
| Soltero | 10.1002/oby.22300 | 2018 | 136 |
| Ahmad | 10.1186/s12937-018-0379-1 | 2018 | 134 |
| Bishop | 10.1016/j.childyouth.2018.01.037 | 2018 | 122 |
| Latorre-Román | 10.1111/cch.12550 | 2018 | 111 |
| Van Biljon | 10.1016/j.jpeds.2018.07.067 | 2018 | 109 |
| Armstrong | 10.1089/chi.2017.0089 | 2018 | 101 |
| Geria | 10.1111/telefone.12379 | 2018 | 101 |
| Dias | 10.1007/s40279-017-0777-0 | 2018 | 99 |
| Ingul | 10.1016/j.pcad.2018.01.012 | 2018 | 99 |
| Otterbach | 10.1186/s40608-018-0188-2 | 2018 | 94 |
| Njardvik | 10.1093/jpepsy/jsy055 | 2018 | 90 |
| Wald | 10.1177/0009922817733703 | 2018 | 73 |
| Dongqi | ANF | 2018 | 68 |
| Costigan | 10.1080/02640414.2017.1356026 | 2018 | 65 |

| Trial 1st author | Trial DOI | Trial year | Sample Size |
|---|---|---|---|
| Wunram | 10.1007/s00787-017-1071-2 | 2018 | 64 |
| A Bruñó | 10.1016/j.pedn.2018.04.008 | 2018 | 52 |
| Liu | 10.1515/jpem-2017-0431 | 2018 | 50 |
| Vantieghen | 10.1038/s41390-018-0047-3 | 2018 | 48 |
| Chuensiri | 10.1089/chi.2017.0024 | 2018 | 48 |
| Cheunsiri | NR | 2018 | 48 |
| Staiano | 10.1111/ijpo.12438 | 2018 | 46 |
| Cvetković | 10.1111/sms.13241 | 2018 | 42 |
| Cvetković | 10.22190/FUPES180604039M | 2018 | 42 |
| Bharath | 10.1007/s00421-018-3898-8 | 2018 | 40 |
| Vidmar | 10.1111/ijpo.12464 | 2018 | 35 |
| Alhassan | 10.1016/j.pmedr.2018.05.009 | 2018 | 32 |
| Hamila | 10.1016/j.scispo.2018.03.076 | 2018 | 31 |
| Mameli | 10.1111/ijpo.12201 | 2018 | 30 |
| Wong | 10.1123/pes.2017-0198 | 2018 | 30 |
| Morissey | 10.1055/a-0577-4280 | 2018 | 29 |
| Morrissey | 10.1055/a-0639-0726 | 2018 | 29 |
| Morrissey | PMID: 29710370 | 2018 | 29 |
| I Lousa | 10.1038/pr.2018.15 | 2018 | 26 |
| Yli-Piipari | 10.1089/acm.2017.0130 | 2018 | 22 |
| Carnier | 10.1159/000139148 | 2018 | 20 |
| Jung | 10.26582/k.50.1.2 | 2018 | 20 |
| Branco | 10.1519/JSC.0000000000002877 | 2018 | 18 |
| Alemayehu | 10.14814/phy2.13888 | 2018 | 16 |
| Yetgin | NR | 2018 | 16 |
| Paschoal | 10.24220/2318-0897v27n3a4196 | 2018 | 15 |
| Vardar | 10.1080/13813455.2017.1369998 | 2018 | 12 |
| Birnbaum | 10.1080/02640414.2016.1166390 | 2017 | 1293 |
| Natale | 10.1177/0890117116661156 | 2017 | 1211 |
| Taveras | 10.1001/jamapediatrics.2017.1325 | 2017 | 721 |
| Butte | 10.1002/oby.21929 | 2017 | 549 |
| Schroeder | 10.1016/j.pedn.2017.07.004 | 2017 | 521 |
| Gatto | 10.1111/ijpo.12102 | 2017 | 319 |
| Gallota | 10.1080/02640414.2016.1225969 | 2017 | 230 |
| Johnston | 10.14485/HBPR.4.5.5 | 2017 | 198 |
| Arlinghaus | 10.5888/pcd14.170130 | 2017 | 189 |

| Trial 1st author | Trial DOI | Trial year | Sample Size |
|---|---|---|---|
| Cvejic | 10.22190/FUPES1703437C | 2017 | 178 |
| Wilfley | 10.1001/jamapediatrics.2017.2960 | 2017 | 172 |
| Fitzgibbon | 10.1002/oby.20269. | 2017 | 147 |
| Rosas-Nexticapa | NR | 2017 | 121 |
| Lee | 10.3390/ijerph14101178 | 2017 | 115 |
| Lira | 10.1177/0260106017720350 | 2017 | 107 |
| Robertson | 10.1136/archdischild-2016-311514 | 2017 | 105 |
| Tan | 10.1123/pes.2016-0107 | 2017 | 104 |
| Anderson | 10.1002/oby.21967 | 2017 | 103 |
| Hatfield | 10.1177/1524839916642714 | 2017 | 101 |
| Benestad | 10.1136/archdischild-2015-309813 | 2017 | 94 |
| Garibay-Nieto | 10.1210/jc.2016-2701 | 2017 | 83 |
| Burkart | 10.1016/j.pmedr.2017.08.002 | 2017 | 76 |
| Zwinkels | 10.1249/01.mss.0000518945.27391.2b | 2017 | 68 |
| Tripicchio | 10.1089/chi.2017.0021 | 2017 | 64 |
| Yuan | ANF | 2017 | 64 |
| Lopes | 10.1080/17408989.2017.1341474 | 2017 | 60 |
| Nobre | 10.1519/JSC.0000000000001684 | 2017 | 59 |
| Xie | 10.7717/peerj.3286 | 2017 | 58 |
| Martin-Smith | 10.1123/pes.2018-0155 | 2017 | 56 |
| Pizzi | NR | 2017 | 54 |
| Pizzi | NR | 2017 | 54 |
| Pizzi | NR | 2017 | 54 |
| Lima | 10.5935/2359-4802.20170032 | 2017 | 45 |
| Shinn | 10.1037/cpp0000204 | 2017 | 43 |
| Fiorilli | 10.2147/DMSO.S122110 | 2017 | 41 |
| Son | 10.1080/10641963.2017.1288742 | 2017 | 40 |
| Staiano | 10.1111/ijpo.12117 | 2017 | 38 |
| Chen | 10.1111/jhn.12446 | 2017 | 36 |
| Staiano | 10.1002/oby.20210 | 2017 | 33 |
| Zehsaz | 10.1016/j.scispo.2016.07.007 | 2017 | 32 |
| Alhassan | 10.1080/17477160701520108 | 2017 | 32 |
| Lazzer | 10.1007/s40618-016-0551-4 | 2017 | 30 |
| Saelens | 10.1089/chi.2016.0233 | 2017 | 29 |
| Rey | 10.1123/pes.2016-0105 | 2017 | 24 |
| Bluher | 10.1016/j.metabol.2016.11.015 | 2017 | 20 |

| Trial 1st author | Trial DOI | Trial year | Sample Size |
|---|---|---|---|
| Ouerghi | 10.1159/000471882 | 2017 | 18 |
| Graziano | 10.1097/DBP.0000000000000499 | 2017 | 16 |
| Brown | 10.1080/14635240.2016.1157511 | 2016 | 3290 |
| Bogart | 10.1542/peds.2015-2493 | 2016 | 2439 |
| Davis | 10.1016/j.ypmed.2016.05.018 | 2016 | 1898 |
| Hollis | 10.1038/ijo.2016.107 | 2016 | 1150 |
| Pesce | 10.3389/fpsyg.2016.00349 | 2016 | 920 |
| Wong | 10.1097/MPG.0000000000001088 | 2016 | 877 |
| Jarani | 10.1080/02640414.2015.1031161 | 2016 | 760 |
| Daly | 10.1002/jbmr.2688 | 2016 | 727 |
| Warshburger | 10.1016/j.appet.2016.04.007 | 2016 | 685 |
| Cruz | 10.1177/1524839916629974 | 2016 | 655 |
| Tarp | 10.1371/journal.pone.0158087 | 2016 | 632 |
| Danielsson | 10.1111/apa.13360 | 2016 | 589 |
| Feng | 10.4278/ajhp.140801-QUAN-384 | 2016 | 555 |
| Bhave | 10.1136/archdischild-2015-308673 | 2016 | 491 |
| Lubans | 10.1186/s12966-016-0420-8 | 2016 | 361 |
| Babic | 10.1016/j.ypmed.2016.07.014 | 2016 | 322 |
| Alberga | 10.1139/apnm-2015-0413 | 2016 | 304 |
| Goldfield | 10.1139/apnm-2016-0386 | 2016 | 304 |
| Mendoza | 10.1016/j.amepre.2015.09.017 | 2016 | 160 |
| Silva | 10.1123/jpah.2013-0199 | 2016 | 132 |
| George | 10.1016/j.jneb.2015.12.010 | 2016 | 126 |
| Traberg | 10.1371/journal.pone.0157182 | 2016 | 115 |
| Haines | 10.1002/oby.21314 | 2016 | 112 |
| Mayorga-Vega | 10.1177/1356336X15599010 | 2016 | 111 |
| Hay | 10.1038/ijo.2015.241 | 2016 | 106 |
| Norman | 10.1111/ijpo.12013 | 2016 | 106 |
| Davis | 10.1177/1357633X15586642 | 2016 | 103 |
| Ho | 10.1177/2333794X16669014 | 2016 | 99 |
| Martinez | 10.1590/S1980-6574201600040022 | 2016 | 94 |
| Seabra | 10.1080/02640414.2016.1140219 | 2016 | 88 |
| Mazurak | 10.1002/oby.21355 | 2016 | 87 |
| Truby | 10.1371/journal.pone.0151787 | 2016 | 87 |
| Christison | 10.1089/g4h.2015.0097 | 2016 | 84 |
| Thompson | 10.5993/AJHB.40.4.9 | 2016 | 80 |

| Trial 1st author | Trial DOI | Trial year | Sample Size |
|---|---|---|---|
| Cohen | 10.17269/CJPH.107.5470 | 2016 | 78 |
| Ham | 10.1016/j.anr.2016.03.003 | 2016 | 75 |
| Hobkrik | 10.1089/met.2011.0050 | 2016 | 75 |
| Koutsandréou | 10.1249/MSS.0000000000000869 | 2016 | 71 |
| Racil | 10.1139/apnm-2015-0384 | 2016 | 68 |
| Foster | 10.1155/2016/2609504 | 2016 | 60 |
| Dos Santos | 10.1177/2333794X16644139 | 2016 | 55 |
| Yoshimoto | NR | 2016 | 52 |
| Lynch | 10.1177/2150131916644888 | 2016 | 51 |
| Chen | 10.1037/hea0000390 | 2016 | 50 |
| Duman | 10.4274/jcrpe.2284 | 2016 | 50 |
| Racil | 10.5604/20831862.1198633 | 2016 | 47 |
| Tan | 10.1139/apnm-2015-0174 | 2016 | 46 |
| Farinatti | 10.1123/pes.2015-0191 | 2016 | 44 |
| Kim | 10.1016/j.anr.2015.07.006 | 2016 | 42 |
| Koot | 10.1038/ijo.2015.175 | 2016 | 42 |
| Fleischman | 10.1111/cob.12166 | 2016 | 40 |
| Ghahramani | 10.13005/bbra/2318 | 2016 | 40 |
| Yu | 10.5409/wjcp.v5.i3.293 | 2016 | 38 |
| Johnson | 10.1016/j.jsams.2015.05.002 | 2016 | 36 |
| Lopes | 10.1080/02640414.2016.1142107 | 2016 | 33 |
| Zehsaz | 10.1080/00913847.2016.1248223 | 2016 | 32 |
| Gerosa-Neto | 10.12965/jer.1632770.385 | 2016 | 32 |
| Kong | 10.1371/journal.pone.0158589 | 2016 | 31 |
| Crouter | 10.1080/02640414.2016.1209305 | 2016 | 30 |
| Kim | 10.33549/fisiolres.932997 | 2016 | 28 |
| Xinhui | ANF | 2016 | 28 |
| Chunyan | ANF | 2016 | 25 |
| Jung | NR | 2016 | 23 |
| Kargarfard | 10.1080/00913847.2016.1200442 | 2016 | 20 |
| Vasconcellos | 10.1080/02640414.2015.1064150 | 2016 | 20 |
| Mota | 10.21767/2471-8173.100010 | 2016 | 17 |
| Ricci-Vitor | 10.7322/jhgd.119257. | 2016 | 15 |
| Kong | 10.1155/2016/4073618 | 2016 | 10 |
| Erfle | 10.1111/josh.12217 | 2015 | 10206 |
| Cao | 10.1016/j.amepre.2014.12.014 | 2015 | 2446 |

| Trial 1st author | Trial DOI | Trial year | Sample Size |
|---|---|---|---|
| Wilksch | 10.1017/S003329171400289X | 2015 | 1316 |
| Rexen | 10.1111/sms.12293 | 2015 | 1247 |
| Xu | 10.1371/journal.pone.0141421 | 2015 | 1182 |
| Madsen | 10.1089/chi.2015.0002 | 2015 | 879 |
| Roth | 10.1249/MSS.0000000000000703 | 2015 | 709 |
| Taveras | 10.1001/jamapediatrics.2015.0182 | 2015 | 549 |
| Jago | 10.1186/s12966-015-0289-y | 2015 | 508 |
| Cohen | 10.1249/MSS.0000000000000452 | 2015 | 460 |
| Subramanian | 10.7860/JCDR/2015/14881.6818 | 2015 | 439 |
| Alaimo | 10.1007/s10900-015-0005-5 | 2015 | 410 |
| Thompson | 10.1186/s12966-015-0199-z | 2015 | 400 |
| Farias | 10.1016/j.jped.2014.06.004 | 2015 | 386 |
| Pakpour | 10.1542/peds.2014-1987 | 2015 | 357 |
| Goldfield | 10.1037/ccp0000038 | 2015 | 304 |
| Alberga | 10.1038/ijo.2015.133 | 2015 | 304 |
| Nyberg | 10.1371/journal.pone.0116876 | 2015 | 243 |
| Taylor | 10.1542/peds.2015-0595 | 2015 | 206 |
| Zamrazilova | 10.1038/ijo.2015.167 | 2015 | 184 |
| Schmidt | 10.1123/jsep.2015-0069 | 2015 | 181 |
| De Vries | 10.1111/apa.12880 | 2015 | 161 |
| Kilanowsk | 10.1111/phn.12175 | 2015 | 138 |
| Peng | NR | 2015 | 121 |
| Parra-Medina | 10.1089/chi.2014.0120 | 2015 | 118 |
| Serra-Paya | 10.1371/journal.pone.0144502 | 2015 | 113 |
| Kokkvoll | 10.1136/archdischild-2014-307107 | 2015 | 97 |
| Barnett | 10.1016/j.pmedr.2015.08.007 | 2015 | 95 |
| Wang | 10.1186/s12889-015-2535-2 | 2015 | 90 |
| Gerards | 10.1371/journal.pone.0122240 | 2015 | 86 |
| Horner | 10.1123/pes.2015-0067 | 2015 | 81 |
| Larsen | 10.3109/02813432.2015.1067511 | 2015 | 80 |
| Gallotta | 10.3389/fnhum.2015.00577 | 2015 | 78 |
| Carvalho-Ferreira | 10.1016/j.peptídeos.2014.11.010 | 2015 | 75 |
| Chen | 10.1016/j.pedn.2015.01.026 | 2015 | 70 |
| Visuthranukul | 10.1038/pr.2015.142 | 2015 | 70 |
| Masquio | 10.1017/S0007114515001129 | 2015 | 69 |
| Vernadakis | 10.1016/j.compedu.2015.01.001 | 2015 | 66 |

| **Trial 1st author** | **Trial DOI** | **Trial year** | **Sample Size** |
|---|---|---|---|
| Costigan | 10.1016/j.pmedr.2015.11.001 | 2015 | 65 |
| Anderson | 10.1177/1090198114547813 | 2015 | 62 |
| Bruyndonckx | 10.1542/peds.2014-1577 | 2015 | 61 |
| Chen | NR | 2015 | 60 |
| Sharma | 10.1007/s10935-015-0395-2 | 2015 | 57 |
| Falbe | 10.1016/j.acap.2015.02.004 | 2015 | 55 |
| Lambrick | 10.1080/02640414.2015.1048521 | 2015 | 55 |
| Ting | ANF | 2015 | 50 |
| Martin | 10.5604/20831862.1173644 | 2015 | 49 |
| Lau | 10.1080/17461391.2014.933880 | 2015 | 48 |
| Monteiro | 10.1186%2Fs12944-015-0152-9 | 2015 | 48 |
| Abraham | 10.1371/journal.pone.0125673 | 2015 | 48 |
| Inoue | 10.1016/j.jdiacomp.2014.11.002 | 2015 | 45 |
| Dias | 10.1249/MSS.0000000000000705 | 2015 | 44 |
| Prado | 10.1123/pes.2015-0018 | 2015 | 43 |
| Donath | 10.1111/cch.12232 | 2015 | 41 |
| Faigenbaum | 10.1519/JSC.0000000000000812. | 2015 | 41 |
| Mingwei | ANF | 2015 | 41 |
| Al Saif | 10.1589/jpts.27.1697 | 2015 | 40 |
| Youssef | 10.1123/pes.2014-0008 | 2015 | 39 |
| Andersen | 10.1136/bjsports-2011-090333 | 2015 | 39 |
| Wong | 10.1096/fasebj.29.1_supplement.747.18 | 2015 | 38 |
| Jones | 10.1123/pes.2015-0116 | 2015 | 37 |
| Crouter | 10.1371/journal.pone.0141584. | 2015 | 36 |
| Direito | 10.2196/jmir.4568 | 2015 | 34 |
| Monteiro | 10.1186/s12944-015-0152-9 | 2015 | 32 |
| Chen | 10.13481/j.1671-587x.20150537 | 2015 | 30 |
| Park | 10.14352/jkaie.2015.19.1.99 | 2015 | 30 |
| Lee | 10.1589/jpts.27.1903 | 2015 | 30 |
| Mahgoub | NR | 2015 | 30 |
| Fisher | 10.1371/journal.pone.0138853 | 2015 | 28 |
| Brandao | NR | 2015 | 27 |
| McNarry | 10.1139/apnm-2015-0051 | 2015 | 26 |
| Kelly | 10.1515/jpem-2014-0470 | 2015 | 26 |
| Vasconcelos | 10.1055/s-0034-1398654 | 2015 | 25 |
| Nunes | 10.1080/02640414.2015.1080384 | 2015 | 25 |

| Trial 1st author | Trial DOI | Trial year | Sample Size |
|---|---|---|---|
| Antunes | 10.1590/2359-3997000000095 | 2015 | 25 |
| Brandão | NR | 2015 | 25 |
| Epstein | 10.1016/j.eatbeh.2015.02.001 | 2015 | 24 |
| Liangmei | ANF | 2015 | 24 |
| Nourse | 10.1016/j.jpeds.2015.06.015 | 2015 | 20 |
| Mendelson | 10.1111/ijpo.255 | 2015 | 20 |
| Lee | 10.1589/jpts.27.3063 | 2015 | 20 |
| Cunha | 10.1080/02701367.2014.982782 | 2015 | 18 |
| Chansavang | NR | 2015 | 18 |
| Andre | 10.1007/s40519-015-0219-7 | 2015 | 16 |
| Murphy | 10.1177/0009922814528038 | 2015 | 13 |
| Doughty | 10.1089/chi.2014.0004 | 2015 | 12 |
| Sim | 10.1249/MSS.0000000000000687 | 2015 | 10 |
| Kipping | 10.1136/bmj.g3256 | 2014 | 2221 |
| Dzewaltowski | 10.1542/peds.2013-2621 | 2014 | 2211 |
| Lana | 10.1016/j.ypmed.2013.11.015 | 2014 | 2001 |
| Kain | 10.1155/2014/618293 | 2014 | 1949 |
| Tarro | 10.1186/1745-6215-15-58 | 2014 | 1939 |
| Andrade | 10.1186/s12966-014-0153-5 | 2014 | 1440 |
| Grydeland | 10.1136/bjsports-2013-092284 | 2014 | 1324 |
| Li | 10.1186/1471-2458-14-1282 | 2014 | 921 |
| Damsgaard | 10.1017/S0007114514003043 | 2014 | 834 |
| Alexander | 10.1002/oby.20557 | 2014 | 749 |
| Martinez-Vizcaino | 10.1186/s12966-014-0154-4 | 2014 | 712 |
| Alkon | 10.1186/1471-2458-14-215 | 2014 | 689 |
| Santos | 10.1001/jamapediatrics.2013.3688 | 2014 | 687 |
| Jago | 10.1186/s12966-014-0114-z | 2014 | 600 |
| Adab | 10.1136/bmjopen-2013-004579 | 2014 | 574 |
| Wilks | 10.1177/2047487312465691 | 2014 | 429 |
| Habib-Mourad | 10.1186/1471-2458-14-940 | 2014 | 374 |
| Smith | 10.1542/peds.2014-1012 | 2014 | 361 |
| Berry | 10.1038/nutd.2013.42 | 2014 | 358 |
| Foley | 10.1186/1479-5868-11-46 | 2014 | 322 |
| Natale | 10.1177/1524839914523429 | 2014 | 307 |
| Sigal | 10.1001/jamapediatria.2014.1392 | 2014 | 304 |
| Sigal | 10.1001/jamapediatrics.2014.1392 | 2014 | 304 |

| Trial 1st author | Trial DOI | Trial year | Sample Size |
|---|---|---|---|
| Hillman | 10.1542/peds.2013-3219 | 2014 | 296 |
| Meyer | 10.1371/journal.pone.0087929 | 2014 | 289 |
| Markert | 10.3390/ijerph111010327 | 2014 | 289 |
| Simons | 10.1371/journal.pone.0126023 | 2014 | 260 |
| Maddison | 10.1186/s12966-014-0111-2 | 2014 | 251 |
| Maddison | 10.1186/s12966-12014-10111-12962 | 2014 | 251 |
| Ericsson | 10.1111/j.1600-0838.2012.01458.x | 2014 | 220 |
| Khan | 10.1542/peds.2013-2246 | 2014 | 220 |
| McKinney | 10.1080/10852352.2014.881180 | 2014 | 159 |
| Nogueira | 10.1016/j.bone.2014.08.006 | 2014 | 151 |
| Dâmaso | NR | 2014 | 146 |
| Ning | 10.1007/s00394-013-0612-9 | 2014 | 145 |
| Dâmaso | 10.1080/02640414.2014.900692 | 2014 | 139 |
| Rodrıguez-Moran | NR | 2014 | 115 |
| Kong | 10.1186/1471-2458-14-180 | 2014 | 104 |
| Epstein | 10.1089/chi.2013.0123 | 2014 | 101 |
| Telles | 10.1186/1753-2000-7-37 | 2014 | 98 |
| Krustrup | 10.1111/sms.12277 | 2014 | 97 |
| Shore | 10.1177/1054773813485240 | 2014 | 92 |
| Van der Baan-Slootweg | 10.1001/jamapediatrics.2014.521 | 2014 | 90 |
| Love-Osborne | 10.1089/chi.2013.0165 | 2014 | 82 |
| Boodai | 10.1186/1745-6215-15-234 | 2014 | 82 |
| Sanches | 10.1016/j.jacl.2014.02.007 | 2014 | 79 |
| Trost | 10.1001/jamapediatrics.2013.3436 | 2014 | 75 |
| Savoye | 10.2337/dc13-1571 | 2014 | 75 |
| Ackel-D'Elia | 10.1055/s-0033-1345128 | 2014 | 72 |
| Steven | 10.1089/chi.2013.0107 | 2014 | 72 |
| Kilanowski | 10.1177/1059840513506999 | 2014 | 64 |
| Masquio | 10.1111/ijcp.12573 | 2014 | 60 |
| Gonzalez-Heredia | NR | 2014 | 60 |
| Bendiksen | 10.1080/17461391.2014.884168 | 2014 | 59 |
| Schranz | 10.1136/bjsports-2013-092209 | 2014 | 56 |
| Xiuming | 10.3969/j.issn.1004-0188.2014.04.023 | 2014 | 52 |
| Velazquez | 10.1186/1471-2431-14-175 | 2014 | 49 |
| Boer | NR | 2014 | 46 |
| Boutelle | 10.1093/jpepsy/jst142 | 2014 | 44 |

| Trial 1st author | Trial DOI | Trial year | Sample Size |
|---|---|---|---|
| Krafft | 10.1002/oby.20518 | 2014 | 43 |
| Campos | 10.1519/JSC.0b013e3182a996df | 2014 | 42 |
| Martí | 10.3305/nh.2015.31.2.7929 | 2014 | 42 |
| Li | 10.13481/j.1671-587x.20140536 | 2014 | 40 |
| Keating | 10.1155/2014/834865 | 2014 | 38 |
| Harder-Lauridsen | 10.1186/1471-2431-14-273 | 2014 | 38 |
| Silva | 10.1590/S0101-32892014000200002 | 2014 | 36 |
| Hamilton-Shield | 10.3310/hta18470 | 2014 | 36 |
| Weigensberg | 10.1186/1472-6882-14-28 | 2014 | 35 |
| Stark | 10.1093/jpepsy/jsu059 | 2014 | 33 |
| Chaouachi | 10.1519/JSC.0000000000000305 | 2014 | 30 |
| Starkoff | 10.1139/apnm-2016-0240 | 2014 | 27 |
| Starkoff | 10.7575/aiac.ijkss.v.2n.3p.1 | 2014 | 27 |
| Thivel | 10.1016/j.physbeh.2014.06.013 | 2014 | 26 |
| Errickson | NR | 2014 | 24 |
| Sanders | NR | 2014 | 24 |
| Looney | 10.1177/0009922814541803 | 2014 | 22 |
| Seabra | 10.1111/sms.12268 | 2014 | 20 |
| Tang | 10.6133/apjcn.2014.23.3.02 | 2014 | 20 |
| McCormack | 10.1111/j.2047-6310.2013.00180.x | 2014 | 18 |
| Mendelson | 10.4187/respcare.02948 | 2014 | 18 |
| Gunnarsdottir | 10.1027/1901-2276/a000024 | 2014 | 16 |
| Rynders | 10.1249/MSS.0b013e31823cef5e | 2014 | 16 |
| Brito | 10.1111/ijpo.240 | 2014 | 15 |
| Shultz | PMID: 25435766 | 2014 | 11 |
| Procopio | 10.23860/thesis-procopio-andrew-2014 | 2014 | 6 |
| Shultz | NR | 2014 | 3 |
| Meng | 10.1371/journal.pone.0077971 | 2013 | 8301 |
| Bonsergent | 10.1016/j.amepre.2012.09.055 | 2013 | 3538 |
| Annesi | 10.1007/s12529-013-9361-7 | 2013 | 2308 |
| Christiansen | 10.1111/sms.12088 | 2013 | 1348 |
| Johnston | 10.1111/josh.12013 | 2013 | 835 |
| Safdie | 10.21149/spm.v55s3.5138 | 2013 | 830 |
| Melnyk | 10.1016/j.amepre.2013.05.013 | 2013 | 779 |
| Heidemann | 10.1016/j.ypmed.2013.04.015 | 2013 | 717 |
| Telford | 10.1249/MSS.0b013e318293b1ee | 2013 | 708 |

| Trial 1st author | Trial DOI | Trial year | Sample Size |
|---|---|---|---|
| Copeland | 10.2337/dc12-2534 | 2013 | 699 |
| Klakk | 10.1186/1471-2431-13-170 | 2013 | 632 |
| Cunha | 10.1371/journal.pone.0057498 | 2013 | 574 |
| Elder | 10.1111/j.2047-6310.2013.00164.x | 2013 | 541 |
| Reed | 10.1123/jpah.10.2.185 | 2013 | 470 |
| Sacchetti | 10.1111/josh.12076 | 2013 | 438 |
| Laguna | 10.1016/j.numecd.2012.10.002 | 2013 | 437 |
| Herscovici | PMID: 24096971 | 2013 | 405 |
| Siergrist | 10.1016/j.nut.2012.12.011 | 2013 | 402 |
| Whittemore | 10.1016/j.jadohealth.2012.07.014 | 2013 | 384 |
| Davoli | 10.1542/peds.2013-1738 | 2013 | 372 |
| Horton | NR | 2013 | 361 |
| Dewar | 10.1016/j.amepre.2013.04.014 | 2013 | 357 |
| Nemet | 10.1186/1471-2431-13-45 | 2013 | 342 |
| Fairclough | 10.1186/1471-2458-13-626 | 2013 | 318 |
| Marild | 10.1111/j.2047-6310.2012.00105.x | 2013 | 265 |
| Wright | 10.1016/j.ijnurstu.2012.09.004 | 2013 | 251 |
| Bielec | 10.3109/01460862.2013.777818 | 2013 | 243 |
| Löfgren | 10.1249/MSS.0b013e31827c0889 | 2013 | 232 |
| McFarlin | 10.1111/j.1740-8709.2011.00398.x | 2013 | 221 |
| Eather | 10.1016/j.ypmed.2012.10.019 | 2013 | 213 |
| Davis | 10.1089/chi.2013.0053 | 2013 | 210 |
| Bellows | 10.5014/ajot.2013.005777 | 2013 | 201 |
| Wong | 10.1111/jocn.12098 | 2013 | 185 |
| Smith | 10.1177/1059840512472708 | 2013 | 160 |
| Madsen | 10.1001/jamapediatrics.2013.1071 | 2013 | 156 |
| Nguyen | 10.1038/ijo.2012.74 | 2013 | 151 |
| Lochrie | 10.1037/cpp0000020 | 2013 | 130 |
| Pesce | 10.1177/0017896912444176 | 2013 | 125 |
| Olvera | 10.1016/j.amepre.2012.11.018 | 2013 | 122 |
| Haines | 10.1001/jamapediatrics.2013.2356 | 2013 | 121 |
| Wake | 10.1136/bmj.f3092 | 2013 | 118 |
| Schuna | 10.1111/josh.12005 | 2013 | 116 |
| Mirza | 10.3945/ajcn.112.042630 | 2013 | 113 |
| Dennis | 10.1089/chi.2011.0092 | 2013 | 112 |
| Vásquez | 10.3305/nh.2013.28.2.6159 | 2013 | 111 |

| Trial 1st author | Trial DOI | Trial year | Sample Size |
|---|---|---|---|
| Fang | 10.1037/a0030925 | 2013 | 108 |
| Patrick | 10.1177/193229681300700322 | 2013 | 101 |
| Lucertini | 10.1080/17461391.2012.746732 | 2013 | 101 |
| Olvera | 10.1093/jpepsy/jst041 | 2013 | 99 |
| Dâmaso | 10.1155/2013/541032 | 2013 | 97 |
| Elizondo-Montemayor | 10.1111/jhn.12070 | 2013 | 96 |
| Grydeland | 10.1186/1479-5868-10-17 | 2013 | 96 |
| Castro | 10.1016/j.amepre.2012.11.024 | 2013 | 95 |
| Takai | NR | 2013 | 94 |
| Takai | NR | 2013 | 94 |
| Saelens | 10.1093/jpepsy/jst054 | 2013 | 89 |
| Buchan | 10.1186/1471-2458-13-498 | 2013 | 89 |
| Lai | 10.7150/ijbs.4918 | 2013 | 88 |
| Milano | 10.1590/S0004-27302013000700006 | 2013 | 75 |
| Viciana | NR | 2013 | 75 |
| Hammons | 10.1016/j.jneb.2013.01.023 | 2013 | 73 |
| Nidhi | 10.1089/acm.2011.0868 | 2013 | 72 |
| De la Cruz-Muñoz | 10.1007/s11695-012-0730-0 | 2013 | 71 |
| Branscum | 10.1097/FCH.0b013e31826d7607 | 2013 | 71 |
| Vivian | 10.4172/2165-7904.1000191 | 2013 | 67 |
| Huang | 10.1007/s12020-012-9808-7 | 2013 | 65 |
| D Sheehan | 10.1016/j.jshs.2013.02.002 | 2013 | 64 |
| Blakely Brown | 10.1177/0145721712465343 | 2013 | 64 |
| Kellam | 10.21149/spm.v55s3.5142 | 2013 | 62 |
| Kong | 10.1155/2013/575016 | 2013 | 60 |
| Kilanowsk | 10.1097/FCH.0b013e31829d277e | 2013 | 59 |
| Davis | 10.1093/jpepsy/jst005 | 2013 | 58 |
| Staiano | 10.1002/oby.20282 | 2013 | 54 |
| Wright | 10.1002/oby.20388 | 2013 | 50 |
| Zhao | 10.3389/fendo.2022.972954 | 2013 | 45 |
| Lee | 10.1152/ajpendo.00285.2013 | 2013 | 44 |
| Dias | NR | 2013 | 44 |
| Bender | 10.1089/chi.2012.0118 | 2013 | 43 |
| Farah | 10.1111/j.2047-6310.2012.00145.x | 2013 | 43 |
| Boudreau | 10.1016/j.amepre.2012.11.026 | 2013 | 41 |
| Thompson | 10.1111/phn.12009 | 2013 | 41 |

| Trial 1st author | Trial DOI | Trial year | Sample Size |
|---|---|---|---|
| O'Connor | 10.1111/j.1365-2214.2011.01344.x | 2013 | 40 |
| Silva | 10.6063/motricidade.9(1).2459 | 2013 | 38 |
| Siwik | 10.3122/jabfm.2013.02.120118 | 2013 | 35 |
| Racil | 10.1007/s00421-013-2689-5 | 2013 | 34 |
| Antunes | 10.1590/S0103-05822013000300015 | 2013 | 34 |
| Militão | 10.2147/DMSO.S52166 | 2013 | 34 |
| Boer | 10.1177/0269215513498609 | 2013 | 32 |
| Ghorbanian | 10.5812/ijem.8178 | 2013 | 30 |
| Koubaa | 10.9790/3013-32103137 | 2013 | 29 |
| Obert | 10.1002/oby.20495 | 2013 | 28 |
| Karner-Rezek | 10.2147/IJGM.S40187 | 2013 | 28 |
| Regaieg | 10.4103/2230-8210.122619 | 2013 | 28 |
| Carnier | 10.1016/j.appet.2013.05.018 | 2013 | 26 |
| Fazelifar | 10.1590/s1517-86922013000500010 | 2013 | 24 |
| Calcaterra | 10.1515/jpem-2012-0157 | 2013 | 22 |
| Hansen | 10.1080/02640414.2013.792951 | 2013 | 20 |
| Alberga | 10.3810/psm.2013.09.2028 | 2013 | 19 |
| Mucci | 10.1002/ppul.22646 | 2013 | 18 |
| Ramon-Krauel | 10.1089/chi.2013.0022 | 2013 | 17 |
| Jeon | 10.1016/j.imr.2013.10.001 | 2013 | 15 |
| Sandbakk | 10.1519/JSC.0b013e3182752f08 | 2013 | 15 |
| Shing | 10.1519/JSC.0b013e31827e1644 | 2013 | 7 |
| Willi | 10.1111/j.2047-6310.2011.00042.x | 2012 | 4363 |
| Brandstetter | 10.1159/000336255 | 2012 | 2154 |
| Williamson | 10.1038/oby.2012.60 | 2012 | 2060 |
| Llargués | 10.1016/j.endonu.2012.03.002 | 2012 | 2010 |
| Klish | 10.1097/MPG.0b013e3182318b39 | 2012 | 1714 |
| Rush | 10.1017/S0007114511003151 | 2012 | 1348 |
| Alvirde-García | NR | 2012 | 1224 |
| De Coen | 10.1017/S1368980012000687 | 2012 | 1102 |
| Rush | 10.1017/S0007114513002316 | 2012 | 926 |
| Ezendam | 10.1001/archpediatrics.2011.204 | 2012 | 883 |
| Coleman | 10.1186/1479-5868-9-80 | 2012 | 827 |
| Crespo | 10.1007/s12160-011-9332-7 | 2012 | 808 |
| Jago | 10.1186/1479-5868-9-83 | 2012 | 793 |
| Daniels | 10.1038/ijo.2012.96 | 2012 | 698 |

| Trial 1st author | Trial DOI | Trial year | Sample Size |
|---|---|---|---|
| Bugge | 10.1249/MSS.0b013e31824bd579 | 2012 | 696 |
| Cui | 10.1136/bmjopen-2011-000721 | 2012 | 682 |
| Telford | 10.2105/AJPH.2011.300220 | 2012 | 620 |
| Yin | 10.1089/chi.2011.0085 | 2012 | 574 |
| Bacardí-Gascon | 10.3305/nh.2012.27.3.5756 | 2012 | 532 |
| Rosário | 10.3390/ijerph9041355 | 2012 | 464 |
| Story | 10.1038/oby.2012.89 | 2012 | 454 |
| Yin | 10.1089/chi.2012.0125. | 2012 | 423 |
| Piziak | 10.3390/ijerph9041319 | 2012 | 413 |
| Lubans | 10.1001/archpediatrics.2012.41 | 2012 | 357 |
| Maddison | 10.1186/1479-5868-9-54 | 2012 | 322 |
| Magnusson | 10.1093/her/cys049 | 2012 | 321 |
| Finkelstein | 10.1016/j.jpeds.2013.01.009 | 2012 | 285 |
| Wright | 10.1089/chi.2012.0045 | 2012 | 251 |
| Davis | 10.1001/2012.jama.10762 | 2012 | 222 |
| Debar | 10.1542/peds. 2011-0863 | 2012 | 208 |
| DeBar | 10.1542/peds.2011-0863 | 2012 | 208 |
| Davis | 10.1001/jama.2015.16174. | 2012 | 205 |
| Chehuen | 10.1590/S1517-86922011000400003 | 2012 | 205 |
| Sigmund | 10.1186/1471-2458-12-570 | 2012 | 176 |
| Ives | 10.4321/S1139-76322012000300005 | 2012 | 174 |
| Slusser | 10.1089/chi.2011.0060 | 2012 | 160 |
| Toulabi | 10.1016/j.jfma.2011.05.007 | 2012 | 152 |
| Niet | 10.1111/j.2047-6310.2012.00048.x | 2012 | 141 |
| Makni | 10.1038/ijo.2011.257 | 2012 | 131 |
| Lloyd-Richardson | 10.1542/peds.2011-3283 | 2012 | 118 |
| Gronbaek | 10.1097/MPG.0b013e31822cdedf | 2012 | 117 |
| Lisón | 10.1016/j.acap.2012.03.003 | 2012 | 110 |
| Barkin | 10.1542/peds.2011-3762 | 2012 | 106 |
| Savoye | 10.1542/peds.2010-0697 | 2012 | 105 |
| Quattrin | 10.1542/peds.2012-0701 | 2012 | 105 |
| Kirk | 10.1016/j.jpeds.2012.01.041 | 2012 | 102 |
| Hasson | 10.1038/oby.2010.343 | 2012 | 100 |
| Herrick | 10.1111/j.1746-1561.2012.00722.x | 2012 | 100 |
| Weeks | 10.1111/j.2047-6310.2011.00026.x | 2012 | 99 |
| Waling | 10.1155/2012/913965 | 2012 | 93 |

| Trial 1st author | Trial DOI | Trial year | Sample Size |
|---|---|---|---|
| Kamal | 10.1186/1758-5996-4-27 | 2012 | 93 |
| Da Silva | 10.1159/000345840 | 2012 | 84 |
| Ramirez | 10.3305/nh.2012.27.3.5725 | 2012 | 84 |
| Carnier | 10.1016/j.physbeh.2011.08.014 | 2012 | 83 |
| Romero | 10.4278/ajhp.090123-ARB-24 | 2012 | 81 |
| Da Silva | 10.1002/ppul.21502 | 2012 | 76 |
| Bohnert | 10.1177/0272431612466174 | 2012 | 76 |
| Bocca | 10.1001/archpediatrics.2012.1638 | 2012 | 75 |
| Mayorga-Vega | 10.2466/06.10.25.PMS.115.6.984-996 | 2012 | 75 |
| Talakoub | NR | 2012 | 72 |
| Kalavainen | 10.1038/sj.ijo.0803628 | 2012 | 70 |
| Maloney | 10.1089/g4h.2011.0009 | 2012 | 64 |
| Hogg | 10.1515/jpem-2012-0027 | 2012 | 61 |
| Adsiz | NR | 2012 | 60 |
| Sijie | NR | 2012 | 60 |
| Cheng | NR | 2012 | 60 |
| Cheng | NR | 2012 | 60 |
| Piano | 10.1097/MEG.0b013e32835793ac | 2012 | 58 |
| Christison | 10.1177/0009922811429480 | 2012 | 48 |
| Legantis | PMID: 22648470 | 2012 | 48 |
| Gueugnon | 10.1139/h2012-045 | 2012 | 47 |
| Lee | 10.2337/db12-0214 | 2012 | 45 |
| Lee | NR | 2012 | 44 |
| Wilson | 10.1177/1359105311434050 | 2012 | 43 |
| Wagener | 10.1111/j.2047-6310.2012.00065.x | 2012 | 41 |
| Buchan | 10.4081/jphr.2012.e24 | 2012 | 41 |
| Campos | 10.1007/s12020-012-9613-3 | 2012 | 40 |
| Cao | 10.13481/j.1671-587x.2012.03.005 | 2012 | 40 |
| Woo | 10.1007/s00431-011-1518-2 | 2012 | 39 |
| Da Silva | 10.4187/respcare.01307 | 2012 | 35 |
| Staiano | 10.1177/193229681200600412 | 2012 | 31 |
| Biljon | NR | 2012 | 31 |
| Araujo | 10.1371/journal.pone.0042747 | 2012 | 30 |
| Kim | NR | 2012 | 30 |
| Park | 10.1186/1471-2431-12-111 | 2012 | 29 |
| Hardy | 10.1515/jpem.2011.386 | 2012 | 27 |

| Trial 1st author | Trial DOI | Trial year | Sample Size |
|---|---|---|---|
| Goldfield | 10.1093/jpepsy/jss084 | 2012 | 26 |
| Casazza | 10.1097/MPG.0b013e31823df207 | 2012 | 26 |
| Davis | 10.1249%2FMSS.0b013e31821f5d4e | 2012 | 26 |
| Song | PMID: 22976739 | 2012 | 22 |
| Partsalaki | 10.1515/jpem-2012-0131 | 2012 | 21 |
| Seo | 10.4196/kjpp.2012.16.3.175 | 2012 | 20 |
| De Bock | 10.1017/S136898001100200X | 2012 | 18 |
| Rosenkranz | 10.1152/japplphysiol.00663.2011 | 2012 | 16 |
| Shaib | 10.1177/0145721712446635 | 2012 | 15 |
| Silva | NR | 2012 | 14 |
| Moller | 10.1152/japplphysiol.00316.2010 | 2012 | 12 |
| Sevin | 10.3906/sag-1009-1179 | 2011 | 6847 |
| Jago | 10.1249/MSS.0b013e31820c9797 | 2011 | 4063 |
| Jansen | 10.3109/17477166.2011.575151 | 2011 | 2622 |
| Mohammadi | NR | 2011 | 1300 |
| Hawthorne | 10.1177/1059840510391669 | 2011 | 1293 |
| Katz | 10.1111/j.1746-1561.2010.00553.x | 2011 | 1180 |
| Heer | 10.2105/AJPH.2011.300177 | 2011 | 901 |
| Nemet | 10.1016/j.jpeds.2010.10.040 | 2011 | 725 |
| Siegrist | 10.1111/j.1600-0838.2011.01387.x | 2011 | 724 |
| Llargues | 10.1136/jech.2009.102319 | 2011 | 704 |
| Aburto | 10.1249/mss.0b013e318217ebec. | 2011 | 699 |
| Puder | 10.1136/bmj.d6195 | 2011 | 652 |
| Taveras | 10.1001/archpediatrics.2011.44 | 2011 | 457 |
| Thivel | 10.1007/s00431-011-1466-x | 2011 | 457 |
| Greening | 10.1038/oby.2010.329 | 2011 | 450 |
| Winter | 10.1080/02568543.2011.580211 | 2011 | 405 |
| Cliff | 10.1249/MSS.0b013e3181e741e8 | 2011 | 402 |
| Wyatt | 10.1136/bmjopen-2010-000026 | 2011 | 398 |
| Hendy | 10.1016/j.appet.2011.01.024 | 2011 | 382 |
| Ralph | 10.3945/ajcn.110.009142 | 2011 | 322 |
| Towey | 10.1155%2F2011%2F619643 | 2011 | 272 |
| Resaland | 10.1111/j.1600-0838.2009.01028.x | 2011 | 256 |
| Kolsgaard | 10.1186/1471-2431-11-47 | 2011 | 230 |
| Murer | 10.3945/ajcn.110.002212 | 2011 | 203 |
| Christie | 10.1186/1745-6215-12-242 | 2011 | 200 |

| Trial 1st author | Trial DOI | Trial year | Sample Size |
|---|---|---|---|
| Bean | 10.1177/0009922810393497 | 2011 | 186 |
| Davis | 10.1037/a0021766 | 2011 | 161 |
| Baranowski | 10.1016/j.amepre.2010.09.029 | 2011 | 133 |
| Iversen | PMID: 21886292 | 2011 | 119 |
| Wafa | 10.3109/17477166.2011.566340 | 2011 | 107 |
| Howe | 10.1155/2011/358581 | 2011 | 106 |
| Davis | 10.1016/j.jada.2011.05.009 | 2011 | 104 |
| Lubans | 10.1016/j.ypmed.2011.01.009 | 2011 | 100 |
| Jones | 10.1123/pes.23.4.600 | 2011 | 97 |
| Sun | PMID: 21362327 | 2011 | 93 |
| French | 10.1038/oby.2010.328 | 2011 | 90 |
| Dâmaso | 10.1016/j.peptídeos.2011.04.025 | 2011 | 86 |
| Huang | 10.1111/j.1365-2214.2010.01173.x | 2011 | 85 |
| Boutelle | 10.1038/oby.2010.238. | 2011 | 80 |
| Crocker | 10.1038/ijo.2011.182 | 2011 | 72 |
| Barkin | 10.1177/0009922810379039 | 2011 | 72 |
| Morgan | 10.1038/ijo.2010.151 | 2011 | 71 |
| Kalavainen | 10.1007/s12519-011-0324-2 | 2011 | 70 |
| Bryant | 10.1177/1740774511424766 | 2011 | 70 |
| Ardoy | 10.1016/j.recesp.2011.01.009 | 2011 | 67 |
| Rosa | 10.4274/jcrpe.v3i3.23 | 2011 | 66 |
| Coppins | 10.1038/ejcn.2011.43 | 2011 | 65 |
| Fisher | 10.1186/1471-2431-11-97 | 2011 | 64 |
| Buchan | 10.1002/ajhb.21166 | 2011 | 57 |
| Buchan | 10.1111/j.1600-0838.2011.01303.x | 2011 | 57 |
| Berry | 10.1891/1540-4153.9.4.186 | 2011 | 56 |
| Chen | 10.1016/j.jadohealth.2010.11.243 | 2011 | 54 |
| Cronk | 10.4278/ajhp.091222-quan-396 | 2011 | 54 |
| Sun | 10.3760/cma.j.issn.0366-6999.2011.03.001 | 2011 | 51 |
| Danielsen | 10.1016/j.orcp.2012.06.003 | 2011 | 49 |
| Pitson | 10.1258/jhsrp.2010.010076 | 2011 | 47 |
| Kim | 10.7570/kjo.2011.20.3.138 | 2011 | 45 |
| Gallota | 10.1111/j.1600-0838.2009.01009.x | 2011 | 44 |
| Faigenbaum | 10.1123/pes.23.4.573 | 2011 | 40 |
| Saygun | 10.11613/BM.2015.013 | 2011 | 40 |
| Ozgur | ANF | 2011 | 40 |

| Trial 1st author | Trial DOI | Trial year | Sample Size |
|---|---|---|---|
| Zorba | NR | 2011 | 40 |
| Zorba | PMID: 22212270 | 2011 | 40 |
| Saygin | 10.5897/AJPP11.114 | 2011 | 39 |
| Saygın | NR | 2011 | 39 |
| Davis | 10.1249/MSS.0b013e31821f5d4e | 2011 | 38 |
| Suh | 10.4093/dmj.2011.35.4.418 | 2011 | 38 |
| Mello | 10.1111/j.1751-7176.2010.00388.x | 2011 | 30 |
| Elloumi | 10.1002/pri.470 | 2011 | 28 |
| Doyle-Baker | 10.1139/h11-042 | 2011 | 27 |
| Farris | PMID: 22163176 | 2011 | 25 |
| Roshan | NR | 2011 | 24 |
| Gallotta | 10.1111/sms.2011.21.issue-2 | 2011 | 21 |
| Sperlich | 10.1519/JSC.0b013e3181d67c38 | 2011 | 19 |
| Stark | 10.1038/oby.2010.87 | 2011 | 18 |
| Huang | ANF | 2011 | |
| Li | 10.1016/S0895-3988(10)60050-5 | 2010 | 4700 |
| Hollar | 10.1353/hpu.0.0304 | 2010 | 3769 |
| Foster | 10.1056/NEJMoa1001933 | 2010 | 2267 |
| Mauriello | 10.1016/j.ypmed.2010.08.004 | 2010 | 1800 |
| Boyle-Holmes | 10.1177/1090198109343895 | 2010 | 1464 |
| Hoelscher | 10.1038/oby.2009.430 | 2010 | 1107 |
| Katz | NR | 2010 | 1106 |
| Klein | 10.1016/j.eclnm.2010.03.002 | 2010 | 1050 |
| Aguilar | 10.1016/j.jpeds.2009.12.046 | 2010 | 1044 |
| Levy | 10.1186/1471-2458-12-152 | 2010 | 1020 |
| Fitzgibbon | 10.1038/oby.2010.314 | 2010 | 618 |
| Kriemler | 10.1136/bmj.c785 | 2010 | 540 |
| Neumark-Sztainer | 10.1016/j.amepre.2010.07.017 | 2010 | 356 |
| Fraser | 10.1111/j.1099-0860.2010.00329.x | 2010 | 325 |
| Klesges | 10.1001/archpediatrics.2010.196 | 2010 | 303 |
| Haire-Joshu | 10.1038/oby.2009.435 | 2010 | 296 |
| Dzewaltowski | 10.1186/1479-5868-7-90 | 2010 | 273 |
| Robinson | 10.1001/archpediatrics.2010.197 | 2010 | 261 |
| Black | 10.1542/peds.2009-1832 | 2010 | 235 |
| Singhal | 10.1038/ejcn.2009.150 | 2010 | 209 |
| Okely | 10.1016/j.jpeds.2010.03.028 | 2010 | 165 |

| Trial 1st author | Trial DOI | Trial year | Sample Size |
|---|---|---|---|
| El Ansari | 10.3390/ijerph7041649 | 2010 | 160 |
| Gittelsohn | 10.1038/oby.2009.436 | 2010 | 117 |
| Sacher | 10.1038/oby.2009.433 | 2010 | 116 |
| Lubans | 10.1016/j.psychsport.2010.06.009 | 2010 | 108 |
| Lubans | 10.1016/j.ypmed.2009.12.003 | 2010 | 108 |
| Shih | 10.1016/j.metabol.2009.06.035 | 2010 | 106 |
| Ford | 10.1136/adc.2009.165340 | 2010 | 106 |
| Shaibi | 10.1177/2150131910377909 | 2010 | 102 |
| Duggins | 10.3122/jabfm.2010.03.080266 | 2010 | 83 |
| Waling | 10.3945/jn.110.125435 | 2010 | 83 |
| De la Cruz-Munoz | 10.1016/j.soard.2010.06.004 | 2010 | 78 |
| Baquet | 10.1519/JSC.0b013e3181d1575a | 2010 | 77 |
| Díaz | 10.1016/j.jada.2009.10.042 | 2010 | 76 |
| Rosenkranz | 10.1186/1471-2458-10-81 | 2010 | 76 |
| Ferguson | 10.1038/sj.ijo.0800968 | 2010 | 70 |
| Matvienko | 10.4278/ajhp.08050146 | 2010 | 70 |
| Lubans | 10.1016/j.ypmed.2009. 12.003. | 2010 | 67 |
| Chen | 10.1093/pubmed/fdp105 | 2010 | 67 |
| Chen | 10.1093%2Fpubmed%2Ffdp105 | 2010 | 67 |
| T Reinehr | 10.1016/j.clnu.2009.12.010 | 2010 | 66 |
| Coleman | 10.1177/0145721710377360 | 2010 | 62 |
| Johnston | 10.1038/oby.2009.241 | 2010 | 60 |
| Tan | 10.1123/pes.22.3.477 | 2010 | 60 |
| Zhen | 10.1371/journal.pone.0235951 | 2010 | 60 |
| Berntsen | 10.3109/17477160902957166 | 2010 | 60 |
| Lee | 10.4070/kcj.2010.40.4.179 | 2010 | 54 |
| De Piano | 10.1097/MEG.0b013e3283346df2 | 2010 | 50 |
| Olvera | 10.1038/oby.2009.439 | 2010 | 46 |
| Fulkerson | 10.1038/oby.2009.434 | 2010 | 44 |
| Bathrellou | 10.14310/horm.2002.1267 | 2010 | 42 |
| Chae | 10.1515/jpem.2010.168 | 2010 | 38 |
| Olvera | 10.5993/ajhb.34.2.2 | 2010 | 37 |
| Prado | 10.1055/s-0030-1267158 | 2010 | 33 |
| Togashi | 10.1080/15438620903423924 | 2010 | 33 |
| Foschini | 10.1038/oby.2009.247 | 2010 | 32 |
| Ounis | 10.1111/j.1651-2227.2010.01920.x | 2010 | 32 |

| **Trial 1st author** | **Trial DOI** | **Trial year** | **Sample Size** |
|---|---|---|---|
| Olvera | 10.1038/ oby.2009.439 | 2010 | 31 |
| Saelens | 10.3109/17477166.2010.482157 | 2010 | 29 |
| Ounis | 10.1159/000275888 | 2010 | 28 |
| Kong | 10.1177/0009922810370364 | 2010 | 28 |
| Velez | 10.1519/JSC.0b013e3181cc230a | 2010 | 28 |
| Whyte | 10.1016/j.metabol.2010.01.002 | 2010 | 27 |
| Adamo | 10.1139/H10-078 | 2010 | 26 |
| Kim | 10.1016/j.jnutbio.2010.10.001 | 2010 | 25 |
| Penko | 10.1007/s12160-010-9164-x | 2010 | 24 |
| Faude | 10.1111/j.1600-0838.2009.01087.x | 2010 | 22 |
| Tsang | 10.1155/2010/672751 | 2010 | 20 |
| Kim | NR | 2010 | 20 |
| Kim | NR | 2010 | 20 |
| Lau | 10.1016/S1728-869X(10)60008-1 | 2010 | 18 |
| Lee | 10.1123/ijsnem.20.4.275 | 2010 | 18 |
| Boddy | NR | 2010 | 16 |
| Van Der Heijden | 10.1038/oby.2009.274 | 2010 | 15 |
| Van Der Heijden | 10.1249/MSS.0b013e3181df16d9 | 2010 | 12 |
| Ingul | 10.1001/arquipediatria.2010.158 | 2010 | 10 |
| Balagopal | 10.1038/oby.2009.498 | 2010 | 8 |
| Marcus | 10.1038/ijo.2009.38 | 2009 | 3135 |
| Donnelly | 10.1016/j.ypmed.2009.07.022 | 2009 | 1490 |
| Gentile | PMID: 19765270 | 2009 | 1323 |
| Gentille | 10.1186/1741-7015-7-49 | 2009 | 1201 |
| Haerens | 10.1016/j.pec.2009.03.020 | 2009 | 1171 |
| Sighn | 10.1001/archpediatrics.2009.2 | 2009 | 1108 |
| Sichieri | 10.1017/S1368980008002644 | 2009 | 927 |
| Schinke | 10.1016/j.ypmed.2009.08.001 | 2009 | 916 |
| Do Prado | 10.2223/JPED.1889 | 2009 | 688 |
| Angelopoulos | 10.1093/eurpub/ckp004 | 2009 | 646 |
| Ramírez | NR | 2009 | 619 |
| Singh | 10.1111/j.1467-789X.2008.00475.x | 2009 | 520 |
| Araujo-Soares | 10.1080/08870440802040707 | 2009 | 291 |
| Wake | 10.1136/bmj.b3308 | 2009 | 258 |
| Petty | 10.1093/jpepsy/jsp007 | 2009 | 207 |
| Dorgo | 10.1519/JSC.0b013e3181b8d42a | 2009 | 192 |

| Trial 1st author | Trial DOI | Trial year | Sample Size |
|---|---|---|---|
| Kalarchian | 10.1542/peds.2008-3727 | 2009 | 192 |
| Walther | 10.1161/CIRCUL | 2009 | 182 |
| Walther | 10.1161/CIRCULATIONAHA.109.865808 | 2009 | 182 |
| Evans | 10.1080/17477160802314997 | 2009 | 168 |
| Wickham | 10.1089/met.2008.0038 | 2009 | 165 |
| Gavan | 10.1016/j.nutres.2009.05.007 | 2009 | 162 |
| Adam | 10.1159/000234415 | 2009 | 162 |
| Shalitin | 10.1159/000245931 | 2009 | 162 |
| Reinehr | 10.1136/adc.2008.143594 | 2009 | 152 |
| Estabrooks | 10.1016/j.amepre.2008.09.024 | 2009 | 135 |
| Kelishadi | 10.1080/07315724.2009.10719792 | 2009 | 120 |
| Macias-Cervantes | 10.1007/s00431-008-0907-7 | 2009 | 76 |
| Nowicka | 10.1177/1403494809344444 | 2009 | 76 |
| Van Vlierberghe | 10.1080/17477160802220533 | 2009 | 66 |
| Leite | 10.1590/S1413-35552009005000009 | 2009 | 64 |
| Davis | 10.1038/oby.2009.19 | 2009 | 54 |
| Davis | 10.1038%2Foby.2009.19 | 2009 | 54 |
| Tjønna | 10.1042/CS20080249 | 2009 | 54 |
| Kovacs | 10.1556/APhysiol.96.2009.3.7 | 2009 | 51 |
| Obert | 10.1136/bjsm.2007.039941 | 2009 | 50 |
| McGuigan | 10.1519/jsc.0b013e3181876a56 | 2009 | 48 |
| Guijun | ANF | 2009 | 47 |
| Farpour-Lambert | 10.1016/j.jacc.2009.08.030 | 2009 | 44 |
| Davis | 10.1249/MSS.0b013e31819b6aea | 2009 | 41 |
| Velázquez-López | 10.1016/S1575-0922(09)73311-X | 2009 | 40 |
| Karacebey | 10.1177/14732300090370 | 2009 | 40 |
| Karacabey | 10.1177/147323000903700523 | 2009 | 40 |
| Prado | 10.1055/s-0029-1233486 | 2009 | 38 |
| Murphy | 10.3109/17477160902846187 | 2009 | 35 |
| Peralta | 10.1016/j.ypmed.2009.04.007 | 2009 | 32 |
| Hagstromer | 10.1111/j.1651-2227.2008.01116.x | 2009 | 31 |
| Sgro | 10.1519/JSC.0b013e3181910746 | 2009 | 31 |
| Tsang | PMID: 24150562 | 2009 | 30 |
| Ounis | 10.1016/j.ando.2009.03.003 | 2009 | 27 |
| Gamelin | 10.1007/s00421-008-0955-8 | 2009 | 22 |
| Elloumi | 10.1111/j.1651-2227.2009.01365.x | 2009 | 21 |

| Trial 1st author | Trial DOI | Trial year | Sample Size |
|---|---|---|---|
| Tsang | NR | 2009 | 20 |
| Benavides | 10.1016/j.ctcp.2008.12.004 | 2009 | 14 |
| Sanigorski | 10.1038/ijo.2008.79 | 2008 | 1807 |
| Webber | 10.1016/j.amepre.2007.11.018 | 2008 | 1712 |
| Foster | 10.1542/peds.2007-1365 | 2008 | 1349 |
| Ploeg | 10.1542/peds.2013-2383 | 2008 | 1157 |
| Vizcaíno | 10.1038/sj.ijo.0803738 | 2008 | 1044 |
| Simon | 10.1038/ijo.2008.99 | 2008 | 954 |
| Kipping | 10.1136/adc.2007.116970 | 2008 | 679 |
| Graf | 10.1080/02640410801930176 | 2008 | 615 |
| Jones | 10.1186/1479-5868-5-42 | 2008 | 606 |
| Vizcaino | NR | 2008 | 530 |
| Paineau | 10.1001/archpediatrics.2007.2 | 2008 | 477 |
| Paineau | NR | 2008 | 471 |
| Spruijt-Metz | 10.1080/17477160802113415 | 2008 | 459 |
| Hamelink-Basteen | 10.1007/BF03086988 | 2008 | 426 |
| Taylor | 10.3945/ajcn.2007.25749 | 2008 | 389 |
| Jurak | NR | 2008 | 328 |
| Salmon | 10.1038/sj.ijo.0803805 | 2008 | 311 |
| Salmon | 10.1093/heapro/dah502 | 2008 | 293 |
| Hasselstrom | 10.1007/s00223-008-9166-x | 2008 | 243 |
| Reed | 10.1016/j.ypmed.2008.02.020 | 2008 | 237 |
| McManus | 10.1016/j.ypmed.2008.06.001 | 2008 | 210 |
| Gutin | 10.1080/17477160801896457 | 2008 | 206 |
| Rosado | 10.1186/1475-2891-7-28 | 2008 | 147 |
| Schiel | 10.1258/jtt.2007.070504 | 2008 | 140 |
| Hughes | 10.1542/peds.2007-1786 | 2008 | 134 |
| McManus | 10.1016/j.ypmed.2008.06 | 2008 | 132 |
| Sollerhed | 10.1111/j.1600-0838.2007.00636.x | 2008 | 132 |
| Knöpfli | 10.1016/j.jadohealth.2007.08.015 | 2008 | 128 |
| Kelishadi | 10.1111/j.1365-2265.2008.03220.x | 2008 | 100 |
| Weeks | 10.1359/jbmr.080226 | 2008 | 99 |
| Janicke | 10.1001/archpedi.162.12.1119 | 2008 | 93 |
| Doyle | 10.1016/j.jadohealth.2008.01.011 | 2008 | 80 |
| Benson | 10.1038/ijo.2008.5 | 2008 | 78 |
| Alves | 10.1590/s0102-311x2008001400020 | 2008 | 78 |

| Trial 1st author | Trial DOI | Trial year | Sample Size |
|---|---|---|---|
| Benson | NR | 2008 | 78 |
| Icahn School of Medicine at Mount Sinai | NR | 2008 | 77 |
| Vissers | 10.1016/j.eclnm.2008.05.002 | 2008 | 76 |
| Muth | 10.18043/ncm.69.6.432 | 2008 | 75 |
| Epstein | 10.1001/archpediatrics.2007.45 | 2008 | 70 |
| Rice | 10.1177/0009922807306168 | 2008 | 68 |
| Benson | 10.1016/j.cct.2006.11.004 | 2008 | 66 |
| Covell | 10.1016/j.apnr.2006.12.004 | 2008 | 62 |
| Munsch | 10.1159/000129659 | 2008 | 56 |
| Chang | 10.1111/j.1467-789X.2007.00455.x | 2008 | 49 |
| Tsiros | 10.1093/ajcn/87.5.1134 | 2008 | 47 |
| Henaghan | 10.1123/pes.20.2.169 | 2008 | 46 |
| Dove | 10.1096/fasebj.22.2_supplement.793 | 2008 | 42 |
| Alwis | 10.1186/1476-5918-7-8 | 2008 | 42 |
| Craeynest | 10.1016/j.eatbeh.2007.03.002 | 2008 | 38 |
| Weigel | 10.1016/j.jneb.2007.07.009 | 2008 | 37 |
| Speroni | 10.1111/j.1744-6155.2008.00149.x | 2008 | 32 |
| Robertson | 10.1136/adc.2008.139162 | 2008 | 27 |
| Wong | NR | 2008 | 24 |
| Wong | NR | 2008 | 24 |
| Wong | NR | 2008 | 24 |
| Wong | NR | 2008 | 24 |
| Wong | PMID: 18461212 | 2008 | 24 |
| Naylor | 10.1249/MSS.0b013e318182a9e0 | 2008 | 23 |
| Leach | 10.1016/j.jmpt.2008.06.003 | 2008 | 22 |
| Nemet | 10.1515/JPEM.2008.21.5.461 | 2008 | 22 |
| Rohrer | 10.1515/JPEM.2008.21.9.837 | 2008 | 22 |
| Weintraub | 10.1001/archpediatrics.2007.43 | 2008 | 21 |
| Mhurchu | 10.1186/1479-5868-5-8 | 2008 | 20 |
| Lazzer | 10.1007/BF03346399 | 2008 | 19 |
| Kim | 10.1111/j.1365-2265.2007.03058.x | 2008 | 17 |
| Wong | NR | 2008 | 12 |
| Haerens | 10.1016/j.jadohealth.2006.09.028 | 2007 | 2840 |
| Jiang | 10.1111/j.1365-2214.2007.00738.x | 2007 | 2452 |
| Jiang | 10.1111/j.1365–2214.2007.00738.x. | 2007 | 2425 |
| Liu | PMID: 17458137 | 2007 | 753 |

| Trial 1st author | Trial DOI | Trial year | Sample Size |
|---|---|---|---|
| James | 10.1136/bmj.39342.571806.55 | 2007 | 644 |
| Lazaar | 10.1111/j.1651-2227.2007.00426.x | 2007 | 425 |
| Stock | 10.1542/peds.2006-3003 | 2007 | 383 |
| Danielzik | 10.1111/j.1651-2227.2007.00165.x | 2007 | 344 |
| Ahamed | 10.1249/01.mss.0000241654.45500.8e | 2007 | 287 |
| Haerens | 10.1016/j.pec.2007.01.003 | 2007 | 281 |
| Dreimane | 10.1016/j.diabres.2006.05.017 | 2007 | 264 |
| MSavoye | 10.1001/jama.297.24.2697 | 2007 | 209 |
| Wilfley | 10.1001/jama.298.14.1661 | 2007 | 204 |
| Barbeu | 10.1038/oby.2007.247 | 2007 | 201 |
| Rodearmel | 10.1542/peds.2006-2927 | 2007 | 192 |
| Tanas | 10.1186%2F1471-2431-7-33 | 2007 | 190 |
| McCallum | 10.1038/sj.ijo.0803509 | 2007 | 163 |
| Reinehr | 10.1038/sj.ijo.0803637 | 2007 | 131 |
| Huang | PMID: 18265541 | 2007 | 120 |
| Golley | 10.1542/peds.2006-1746 | 2007 | 111 |
| Tian | 10.1080/17408989.2015.1072509 | 2007 | 110 |
| Davis | 10.1080%2F02701367.2007.10599450 | 2007 | 94 |
| Fullerton | 10.1038/oby.2007.306 | 2007 | 86 |
| Caranti | 10.1016/j.metabol.2007.05.004 | 2007 | 83 |
| Satoh | 10.1111/j.1742-7924.2007.00077.x | 2007 | 81 |
| Berry | 10.1016/j.apnr.2006.01.007 | 2007 | 80 |
| Rosenbaum | 10.1210/jc.2006-1516 | 2007 | 79 |
| Johnston | 10.1016/j.jadohealth.2013.04.001 | 2007 | 71 |
| Johnston | 10.1080/17477160701305864 | 2007 | 71 |
| Kalavainen | 10.1038/ijo.2011.1 | 2007 | 70 |
| Johnston | 10.1542/peds.2006-3321 | 2007 | 60 |
| McMurray | 10.2310/6650.2007.06031 | 2007 | 58 |
| Eliakim | 10.1515/jpem.2007.20.6.711 | 2007 | 54 |
| Carrel | 10.1001/archpedi.161.6.561 | 2007 | 53 |
| Chehab | 10.1016/j.jadohealth.2006.12.009 | 2007 | 46 |
| Nowicka | 10.1080/17477160701379810 | 2007 | 44 |
| Park | 10.1159/000104137 | 2007 | 44 |
| Shelton | 10.1111/j.1440-1754.2007.01150.x | 2007 | 43 |
| Vignolo | 10.1038/sj.ejcn.1602819 | 2007 | 31 |

| Trial 1st author | Trial DOI | Trial year | Sample Size |
|---|---|---|---|
| Gillis | 10.1515/jpem.2007.20.2.197 | 2007 | 27 |
| Kim | 10.1038/oby.2007.360 | 2007 | 26 |
| Melnyk | 10.1016/j.pedhc.2007.02.009 | 2007 | 23 |
| Vajda | 10.1556/APhysiol.94.2007.3.4 | 2007 | 21 |
| Elloumi | 10.1016/j.scispo.2007.09.001 | 2007 | 20 |
| Kaufman | 10.1123/pes.19.1.82 | 2007 | 20 |
| Kelly | 10.1016/j.metabol.2007.03.009 | 2007 | 19 |
| Heyman | 10.1123/pes.19.4.408 | 2007 | 16 |
| Klijn | 10.1186/1471-2431-7-19 | 2007 | 15 |
| Bell | 10.1210/jc.2007-0779 | 2007 | 14 |
| Casazza | 10.1016/j.eatbeh.2006.01.007 | 2007 | 5 |
| Haerens | 10.1093/her/cyl115 | 2006 | 2840 |
| Pate | 10.1161/circulationaha.106.177052 | 2006 | 1539 |
| Spiegel | 10.1038/oby.2006.11 | 2006 | 1013 |
| Patrick | 10.1001/archpedi.160.2.128 | 2006 | 878 |
| Verstraete | 10.1017/S1368980007223900 | 2006 | 764 |
| Haerens | NR | 2006 | 709 |
| Haerens | NR | 2006 | 557 |
| Reilly | 10.1136/bmj.38979.623773.55 | 2006 | 545 |
| Jago | 10.1016/j.ypmed.2005.12.010 | 2006 | 473 |
| Fitzgibbon | 10.1038/oby.2006.186 | 2006 | 401 |
| Serbescu | 10.1080/08035250600599719. | 2006 | 370 |
| Marks | 10.1016/j.jadohealth.2005.11.002 | 2006 | 319 |
| Haerens | 10.1038/oby.2006.98 | 2006 | 294 |
| Harrison | 10.1016/j.jsams.2006.06.012 | 2006 | 293 |
| Amaro | 10.1007/s00431-006-0153-9 | 2006 | 241 |
| Reinehr | 10.1093/ajcn/84.3.490 | 2006 | 240 |
| Young | 10.1001/archpedi.160.12.1255 | 2006 | 221 |
| Keller | 10.1055/s-0028-1105883 | 2006 | 183 |
| Reinehr | 10.1097/01.mpg.0000235752.29735.31 | 2006 | 119 |
| Faigenbaum | NR | 2006 | 118 |
| Faigenbaum | NR | 2006 | 118 |
| Rodearmel | 10.1038/oby.2006.158 | 2006 | 105 |
| Ebbeling | 10.1542/peds.2005-0983 | 2006 | 103 |
| Davis | 10.1038/oby.2006.232 | 2006 | 100 |
| Linden | 10.1359/jbmr.060304 | 2006 | 99 |

| Trial 1st author | Trial DOI | Trial year | Sample Size |
|---|---|---|---|
| Rudolf | 10.1136%2Fadc.2005.089896 | 2006 | 94 |
| Daley | 10.1542/peds.2006-1285 | 2006 | 81 |
| Robbins | 10.1097/00006199-200605000-00007 | 2006 | 77 |
| Jelalian | 10.1038/sj.ijo.0803069 | 2006 | 76 |
| Tock | 10.1097/01.meg.0000243872.86949.95 | 2006 | 73 |
| Meyer | 10.1016/j.jacc.2006.07.035 | 2006 | 67 |
| Nabkasorn | 10.1093/eurpub/cki159 | 2006 | 59 |
| Fredericks | 10.4314/sajrs.v28i1.25929 | 2006 | 58 |
| Williamson | 10.1038/oby.2006.140 | 2006 | 57 |
| Ingle | 10.1080/02640410500457117 | 2006 | 47 |
| Garcıa-Morales | 10.1016/j.clinthera.2006.05.008 | 2006 | 46 |
| Maahs | 10.4158/ep.12.1.18 | 2006 | 40 |
| Stergioulas | 10.1139/h06-020 | 2006 | 38 |
| Edwards | 10.1038/sj.ejcn.1602353 | 2006 | 37 |
| Golan | 10.1079/bjn20061757 | 2006 | 37 |
| Kotzamanidis | 10.1519/R-16194.1 | 2006 | 30 |
| Goldfield | 10.1542/peds.2005-3052 | 2006 | 30 |
| Impellizzeri | 10.1055/s-2005-865839 | 2006 | 29 |
| Dâmaso | 10.1590/S1517-86922006000500008 | 2006 | 28 |
| Shaibi | 10.1249/01.mss.0000227304.88406.0f | 2006 | 22 |
| Shaibi | 10.1249/01.mss.000227304.88406 | 2006 | 22 |
| Pate | 10.2105/AJPH.2004.045807 | 2005 | 2744 |
| Pate | 10.2105%2FAJPH.2004.045807 | 2005 | 2744 |
| Graf | 10.1017/s1047951105000594 | 2005 | 1678 |
| Coleman | 10.1001/archpedi.159.3.217 | 2005 | 896 |
| Yin | 10.1038/oby.2005.267 | 2005 | 601 |
| Kafatos | 10.1038/sj.ejcn.1602216 | 2005 | 541 |
| Chavarro | 10.1007/s10552-005-0404-5 | 2005 | 508 |
| Korsten-Reck | 10.1038/sj.ijo.0802875 | 2005 | 496 |
| Fitzgibbon | 10.1016/j.jpeds.2004.12.019 | 2005 | 362 |
| Hopper | 10.1080/02701367.2005.10599275 | 2005 | 238 |
| Palmer | 10.1080/19325037.2005.10608164 | 2005 | 223 |
| Goran | 10.1038/oby.2005.86 | 2005 | 209 |
| Harrell | 10.1097/01.smj.0000182499.59715.07 | 2005 | 205 |

| Trial 1st author | Trial DOI | Trial year | Sample Size |
|---|---|---|---|
| McCallum | 10.1111/j.1440-1754.2005.00689.x | 2005 | 163 |
| Resnicow | 10.1038/oby.2005.212 | 2005 | 147 |
| Yin | PMID: 15977132 | 2005 | 140 |
| Frenn | 10.1016/j.apnr.2004.04.003 | 2005 | 103 |
| Rooney | NR | 2005 | 87 |
| Rooney | PMID: 16138517 | 2005 | 87 |
| Kirschenbaum | 10.1038/oby.2005.187 | 2005 | 83 |
| Yu | 10.1519/14994.1 | 2005 | 82 |
| Yu | NR | 2005 | 82 |
| Daley | 10.1186/1471-2458-5-113 | 2005 | 81 |
| Jiang | 10.1136/adc.2005.071753 | 2005 | 68 |
| Rooney | NR | 2005 | 59 |
| Carrel | 10.1001/archpedi.159.10.963 | 2005 | 50 |
| Nemet | 10.1542/peds.2004-2172 | 2005 | 46 |
| Faigenbaum | 10.1123/pes.17.3.237 | 2005 | 43 |
| Epstein | 10.1207/s15324796abm3003_4 | 2005 | 41 |
| Stella | 10.1590/S0100-879X2005001100017 | 2005 | 40 |
| Ribeiro | 10.1161/01.CIR.0000161959.04675.5A | 2005 | 39 |
| St. Elizabeth's Medical Center | NR | 2005 | 30 |
| Lazzer | 10.1038/sj.ijo.0802845 | 2005 | 27 |
| Kim | 10.4040/jkan.2005.35.5.858 | 2005 | 27 |
| Lazzer | 10.1038/sj.ijo.0802977 | 2005 | 26 |
| Savoye | 10.1016/j.jada.2004.12.009 | 2005 | 25 |
| Hara | 10.1007/s00421-005-1374-8 | 2005 | 21 |
| Balagopal | 10.1016/j.jpeds.2004.11.033 | 2005 | 21 |
| Nassis | 10.1016/j.metabol.2005.05.013 | 2005 | 19 |
| Nourry | 10.1007/s00421-005-1341-4 | 2005 | 18 |
| Sacher | 10.1111/j.1365-277x.2004.00578.x | 2005 | 11 |
| Balagopal | 10.1210/jc.2004-2427 | 2005 | 8 |
| Kain | 10.1038/sj.ijo.0802611 | 2004 | 3086 |
| Kain | NR | 2004 | 1636 |
| Treviño | 10.1001/archpedi.158.9.911 | 2004 | 1419 |
| French | 10.2105/ajph.94.9.1507 | 2004 | 750 |
| James | 10.1136/bmj.38077.458438.ee | 2004 | 644 |
| James | NR | 2004 | 574 |

| Trial 1st author | Trial DOI | Trial year | Sample Size |
|---|---|---|---|
| Bayne-Smith | 10.2105/ajph.94.9.1538 | 2004 | 442 |
| Lakes | 10.1016/j.appdev.2004.04.002 | 2004 | 209 |
| Braet | 10.1093/jpepsy/jsh054 | 2004 | 122 |
| Baquet | 10.1519/13813.1 | 2004 | 100 |
| Rolland-Cachera | 10.1038/sj.ijo.0802605 | 2004 | 99 |
| Woo | 10.1161/01.CIR.0000126599.47470.BE | 2004 | 82 |
| Denninson | 10.1001/archpedi.158.2.170 | 2004 | 77 |
| Abroms | 10.4278/0890-1171-19.1.28 | 2004 | 70 |
| MacKelvie | 10.1016/j.bone.2003.12.017 | 2004 | 64 |
| Epstein | 10.1037/0278-6133.23.4.371 | 2004 | 63 |
| Golan | 10.1038/oby.2004.45 | 2004 | 60 |
| Manjunath | NR | 2004 | 60 |
| Dietrich | NR | 2004 | 59 |
| White | 10.1038/oby.2004.132 | 2004 | 57 |
| Dao | 10.1038/sj.ijo.0802535 | 2004 | 55 |
| Dao | 10.1038/sj.ijo.0802542 | 2004 | 55 |
| Grey | 10.1111/j.1746-1561.2004.tb06595.x | 2004 | 41 |
| Lau | NR | 2004 | 38 |
| Watts | 10.1016/j.jpeds.2004.02.027 | 2004 | 28 |
| Lazzer | 10.1038/oby.2004.30 | 2004 | 26 |
| Kelly | 10.1016/j.jpeds.2004.08.004 | 2004 | 25 |
| McManus | 10.1055/s-2005-837438 | 2004 | 25 |
| Watts | 10.1016/j.jacc.2004.01.032 | 2004 | 19 |
| Caballero | 10.1093/ajcn/78.5.1030 | 2003 | 1704 |
| Lohman | 10.1016/j.ypmed.2003.08.004 | 2003 | 1367 |
| van Beurden | 10.1016/S0091-7435(02)00044-0 | 2003 | 1045 |
| Pate | 10.4278/0890-1171-17.3.171 | 2003 | 436 |
| Warren | 10.1093/heapro/dag402 | 2003 | 213 |
| Neumark-Sztainer | 10.1016/S0091-7435(03)00057-4 | 2003 | 201 |
| MacKelvie | 10.1542/peds.112.6.e447 | 2003 | 64 |
| Robinson | NR | 2003 | 61 |
| Robinson | PMID: 12713212 | 2003 | 61 |
| Beech | PMID: 12713210 | 2003 | 60 |
| Story | PMID: 12713211 | 2003 | 54 |
| Story | NR | 2003 | 53 |
| Jago | ANF | 2003 | 46 |

| Trial 1st author | Trial DOI | Trial year | Sample Size |
|---|---|---|---|
| Harvey | 10.1038/oby.2003.87 | 2003 | 37 |
| Baranowski | PMID: 12713209 | 2003 | 35 |
| Baranowski | PMID:12713209 | 2003 | 35 |
| Baranowski | NR | 2003 | 31 |
| Beech | NR | 2003 | 30 |
| Beech | NR | 2003 | 30 |
| Sallis | 10.1016/s0749-3797(02)00646-3 | 2003 | 24 |
| Rowland | 10.1136%2Fadc.88.1.8 | 2003 | 21 |
| McMurray | 10.1016/s1054-139x(02)00348-8 | 2002 | 1140 |
| Manois | 10.1006/pmed.1998.0388 | 2002 | 1046 |
| Manios | 10.1079/BJN2002672 | 2002 | 1046 |
| Eliakim | 10.1007/s00431-002-0980-2 | 2002 | 202 |
| Sung | 10.1136/adc.86.6.407 | 2002 | 82 |
| Mitchell | 10.1542/peds.109.5.e73 | 2002 | 81 |
| Gutin | 10.1093/ajcn/75.5.818 | 2002 | 80 |
| Kang | 10.1097/00005768-200212000-00010 | 2002 | 80 |
| Humphries | 10.1097/00005768-200209000-00005 | 2002 | 78 |
| Barbeau | 10.1067/mpd.2002.127497 | 2002 | 74 |
| Flanagan | 10.1080/02701367.2002.10609029 | 2002 | 58 |
| Faigenbaum | 10.1080/02701367.2002.10609041 | 2002 | 55 |
| Wilson | 10.1207/s15324796abm2404_07 | 2002 | 53 |
| Saelens | 10.1038/oby.2002.4 | 2002 | 44 |
| Falk | 10.1515/jpem.2002.15.5.597 | 2002 | 30 |
| Jelalian | 10.1023/A:1014179811950 | 2002 | 16 |
| Blau | 10.1378/chest.121.4.1117 | 2002 | 13 |
| Sahota | 10.1136/bmj.323.7320.1027 | 2001 | 634 |
| Sahota | 10.1136/bmj.323.7320.1029 | 2001 | 613 |
| Baquet | 10.1055/s-2001-14343 | 2001 | 551 |
| Warshburger | 10.1038/sj.ijo.0801708 | 2001 | 197 |
| Nova | 10.1046/j.1467-0658.2001.00135.x | 2001 | 186 |
| Epstein | 10.1038/oby.2001.103 | 2001 | 89 |
| Nichols | 10.1067/mpd.2001.116698 | 2001 | 67 |
| Manjunath | NR | 2001 | 34 |
| Levine | 10.1002/eat.1091 | 2001 | 24 |
| Gately | 10.1038/sj.ijo.0801405 | 2000 | 217 |
| McKay | 10.1016/S0022-3476(00)70095-3 | 2000 | 145 |

| Trial 1st author | Trial DOI | Trial year | Sample Size |
|---|---|---|---|
| Braet | 10.1016/S0005-7894(00)80004-0 | 2000 | 109 |
| Gutin | 10.1038/oby.2000.3 | 2000 | 79 |
| Epstein | PMID: 10965646 | 2000 | 67 |
| Sothern | 10.1080/713794562 | 2000 | 56 |
| Sothern | 10.1080/713794562 | 2000 | 56 |
| Stratton | 10.1080/001401300750003961 | 2000 | 47 |
| Eliakim | 10.1055/s-2000-3779 | 2000 | 38 |
| Colchico | 10.2105/ajph.90.6.977 | 2000 | 30 |
| DeStefano | 10.1034/j.1399-5448.2000.010202.x | 2000 | 15 |
| Nader | 10.1001/archpedi.153.7.695 | 1999 | 3714 |
| Gortmaker | 10.1001/archpedi.153.4.409 | 1999 | 1295 |
| Robinson | 10.1001/jama.282.16.1561 | 1999 | 225 |
| Winett | 10.1023%2FA%3A1023233416859 | 1999 | 180 |
| Sothern | PMID: 10432772 | 1999 | 87 |
| Owens | 10.1097/00005768-199901000-00022 | 1999 | 74 |
| Ferguson | 10.1093/ajcn/69.6.1130 | 1999 | 43 |
| Schwingshandl | 10.1136/adc.81.5.426 | 1999 | 30 |
| Burke | 10.1016/S0022-3476(98)70315-4 | 1998 | 720 |
| Burke | NR | 1998 | 471 |
| Harrell | 10.1542/peds.102.2.371 | 1998 | 422 |
| Mo-Suwan | 10.1093/ajcn/68.5.1006 | 1998 | 292 |
| Mo-Suwan | NR | 1998 | 170 |
| Stephens | NR | 1998 | 90 |
| Ewart | 10.2105/ajph.88.6.949 | 1998 | 88 |
| Stergioulas | 10.1080/08035259850156986 | 1998 | 28 |
| Treuth | 10.1097/00005768-199812000-00013 | 1998 | 20 |
| Treuth | 10.1097/00005768-199807000-00017 | 1998 | 11 |
| Sallis | 10.2105/AJPH.87.8.1328 | 1997 | 955 |
| Marshal | 10.1123/apaq.14.3.222 | 1997 | 406 |
| Welsman | 10.1136%2Fbjsm.31.2.139 | 1997 | 96 |
| Lillegaard | 10.3109/17518429709167353 | 1997 | 91 |
| Stolley | 10.1177/109019819702400204 | 1997 | 55 |
| Gutin | 10.1016/s0022-3476(97)70280-4 | 1997 | 35 |
| Ignico | 10.1080/02701367.1995.10607659 | 1997 | 28 |
| Rimmer | 10.1080/02701367.1997.10608868 | 1997 | 25 |
| Torigoe | 10.1111/j.1442-200x.1997.tb03551.x | 1997 | 23 |

| Trial 1st author | Trial DOI | Trial year | Sample Size |
|---|---|---|---|
| Piéron | 10.1177/1356336X9600200204 | 1996 | 7721 |
| Luepker | 10.1001/jama.1996.03530340032026 | 1996 | 5106 |
| McKenzie | 10.1006/pmed.1996.0074 | 1996 | 5106 |
| Harrell | 10.1016/s0022-3476(96)70332-3 | 1996 | 1274 |
| Donnelly | 10.1002/j.1550-8528.1996.tb00541.x | 1996 | 338 |
| Falk | 10.2165/00007256-199622030-00004 | 1996 | 32 |
| Faigenbaum | 10.1519/1533-4287(1996)010<0109:TEOSTA>2.3.CO;2 | 1996 | 24 |
| Vandongen | 10.1006/pmed.1995.1003 | 1995 | 1240 |
| VanDongen | ANF | 1995 | 1147 |
| Flores | PMCID: PMC1382101 | 1995 | 110 |
| Connolly | NR | 1995 | 56 |
| Blessing | 10.1123/pes.7.2.192 | 1995 | 25 |
| Shephard | 10.1123/pes.6.1.75 | 1994 | 546 |
| Epstein | 10.1016/0306-4603(94)90038-8 | 1994 | 44 |
| Epstein | 10.1037//0278-6133.13.5.373 | 1994 | 36 |
| Israel | 10.1093/jpepsy/19.6.737 | 1994 | 36 |
| Flodmark | PMID: 8474806 | 1993 | 1774 |
| Sallis | 10.1111/j.1749-6632.1993.tb18844.x | 1993 | 549 |
| Duffy | 10.1111/j.1469-7610.1993.tb01107.x | 1993 | 27 |
| Faigenbaum | 10.1123/pes.5.4.339 | 1993 | 24 |
| Norris | 10.1016/0022-3999(92)90114-H | 1992 | 147 |
| Hansen | 10.1136/bmj.303.6804.682 | 1991 | 132 |
| Zervas | 10.2466/pms.1991.72.3c.1215 | 1991 | 18 |
| Epstein | PMID: 2232019 | 1990 | 76 |
| Reybrouck | 10.1111/j.1651-2227.1990.tb11336.x | 1990 | 14 |
| Bush | NR | 1989 | 431 |
| Siegel | 10.1123/pes.1.2.145 | 1989 | 50 |
| Killen | 10.1001/jama.1988.03410120074030 | 1988 | 1447 |
| Rocchini | NR | 1988 | 72 |
| Rocchini | PMID: 3288957 | 1988 | 45 |
| Becque | PMID: 3357722 | 1988 | 22 |
| Hills | 10.1111/j.1365-2214.1988.tb00592.x | 1988 | 20 |
| Roth | 10.1097/00006842-198707000-00004 | 1987 | 2441 |
| Epstein | 10.1037//0022-006x.55.1.91 | 1987 | 76 |
| Mellin | PMID: 3819254 | 1987 | 66 |
| Rocchini | 10.1161/01.hyp.10.3.267 | 1987 | 50 |

| Trial 1st author | Trial DOI | Trial year | Sample Size |
|---|---|---|---|
| Rocchini | NR | 1987 | 50 |
| Epstein | 10.1016/S0005-7894(87)80016-3 | 1987 | 41 |
| Hayashi | NR | 1987 | 18 |
| Savage | PMID: 3702647 | 1986 | 48 |
| Epstein | 10.1037/0022-006X.54.3.400 | 1986 | 41 |
| Senediak | 10.1017/S0141347300012027 | 1985 | 45 |
| Epstein | 10.1016/S0005-7894(85)80002-2 | 1985 | 41 |
| Campaigne | 10.1080/00913847.1985.11708949 | 1985 | 40 |
| Israel | 10.1016/S0005-7894(85)80043-5 | 1985 | 33 |
| Epstein | 10.1016/s0022-3476(85)80506-0 | 1985 | 23 |
| Epstein | 10.1016/S0005-7894(85)80046-0 | 1985 | 19 |
| Epstein | 10.1037//0022-006x.52.3.429 | 1984 | 53 |
| Kirschenbaum | 10.1016/S0005-7894(84)80051-9 | 1984 | 40 |
| Linder | PMID: 6353127 | 1983 | 50 |
| Brownell | NR | 1983 | 42 |
| Campaigne | NR | 1982 | 19 |
| Epstein | 10.1037//0022-006x.49.5.674 | 1981 | 76 |
| Brown | NR | 1981 | 38 |
| Xiuming | ANF | | 70 |

NR: Not reported; ANF: Article not found

**Table S5. Characteristics of 137 reviews included.**

| Study | Country | Design | Participants | Included RCTs (n) | Setting | Who received the intervention | Intervention(s) |
|---|---|---|---|---|---|---|---|
| **Reviews Including Physical Activity-Only Interventions** | | | | | | | |
| Zouhal et al., 2020 | France | SR | F and M; 5-90 years; Obesity + overweight; Other conditions: NR; n = 692. | 116 | NR | Children and adolescents | Aerobic endurance 8-56 weeks; HIIT: 5-21 weeks; Resistance and Strength training: 6-56 weeks; |
| Liu et al., 2024 | United Kingdom | SRMA | F and M; 5-18 years; Overweight + Obesity + Normal weight; Other conditions: No; n = 577 | 77 | School | Children and adolescents | HIIT: 6-12 weeks; Running, games, resistance training, sprint cycling. |
| Garcia-Hermoso et al., 2020-a | Spain | SRMA | F and M; 5-12 years; normal weight + overweight + obesity. Another condition: NR. n = 48185. | 66 | NR | Children | Fitness infusion 28-39 weeks; Teaching strategies; |
| Kelley et al., 2019 | United States of America | SRMA | F and M; 2-18years; Obesity and Overweight Other conditions: NR; N = 2792. | 57 | NR | Children and adolescents | Aerobic exercises 6-36 weeks; Strength training 6-36 weeks. Combined aerobic and strength 6-36 weeks. |
| Steele et al., 2021 | United Kingdom | SRMA | F and M; 7 – 16 years; Normal weight + obesity. Other conditions: NR; N = NR. | 54 | NR | Children and adolescents | MICT = moderate intensity continuous training 4-12 weeks; IT = interval training 4-12 weeks; |
| Xu et al., 2022 | China | SRMA | F and M; 9.3-17 years; Overweight/Obesity; Other conditions = NO. n = 2.599. | 46 | NR | Children and adolescents | Aerobic training; Resistance training; Combined Training; HIIT training. |

| Study | Country | Design | Participants | Included RCTs (n) | Setting | Who received the intervention | Intervention(s) |
|---|---|---|---|---|---|---|---|
| | | | | | | | 20-54 weeks; 20-50 weeks; 30-90 weeks; 6-52 weeks. |
| Kelley et al., 2021 | United States of America | SRMA | F and M; 7-19 years; Obesity; Other condition: hyperlipidemia, hypertension or prehypertension, hyperinsulinemia and insulin resistance; n =NR | 39 | Schools | Children and adolescents | Aerobic, strength training, both and usual care 6-24 weeks. |
| Paes et al., 2015 | Brazil | SR | F and M; 8-17y; Normal weight + overweight/obesity; Other health conditions= NR; n= NR | 39 | NR | Children and adolescents | PA (aerobic, resistance, combined and recreational activities); 4 to 13 weeks |
| Dos Santos et al., 2021 | Brazil | SR | F and M; 6-19 years; Overweight + Obesity; Other condition = No; n = 335 | 37 | NR | Children and adolescents | Dance: 16 weeks - 2 years; African jazz, hip-hop, step, rumba, salsa, ballet, cheerleading, modern, line dance. |
| Álvarez-Bueno et al., 2017 | Spain | SRMA | F and M; 4-14y; normal weight + Overweight/obesity; Other health condition= NR; n= 5527 | 36 | Curricular school time; Out of school; Summer camp | Children and adolescents | PA (physical education lessons; active breaks; PA extracurricular activities); 4 to 54 weeks |
| Lee et al., 2020 | Republic of Korea | SRMA | F and M; 7-18 years; Obesity and overweight; Other conditions: NR; n = NR. | 27 | NR | Children and adolescents | Aerobic exercise 8-54weeks; Resistance exercise 22-24 weeks; Combined aerobic training 8-22weeks. |

| Study | Country | Design | Participants | Included RCTs (n) | Setting | Who received the intervention | Intervention(s) |
|---|---|---|---|---|---|---|---|
| Méndez-Hernández et al., 2022 | Mexico | SRMA | F and M; 5-19years; Obesity and overweight; other conditions: NR; n= 1834. | 27 | Only home-based reported | Children and adolescents | Strength training 5-52 weeks. |
| Cheng et al., 2022 | China | SRMA | F and M; 7-17 years; Overweight + Obesity; Other conditions: NR; n=661 | 26 | NR | Children and adolescents | Whole body physically active games 8 weeks; Aerobics 8-32 weeks; Combined 8-24 weeks; Aerobics + sports 12-24 weeks; Strength 12-24 weeks; Dance revolution 12 weeks; Multiple sports 12 weeks; HIIT 12 weeks;Control NR. |
| Parikh and Stratton et al., 2011 | United Kingdom | SR | F and M; 5-18y; Normal weight + overweight/obesity; Other health conditions= NR; n= 18210 | 25 | NR | Children and adolescents | PA (aerobic exercises); medium to high intensity, 7 weeks to 10 months |
| Braaksma et al., 2018 | Netherlands | SR | F and M; 6-12y; Normal weight + overweight/obesity; Other health condition= NR; n= 7071 | 23 | School; Home; Research facility; Combination of school and community | Children and parents | PA (aerobic exercises); 1 – 5 days/wk; 40 – 80’/day, 80–100% of maximal aerobic speed or 50–80% VO2peak, 6 to 104 weeks |
| Garcia-Hermoso et al., 2020-b | Spain | SRMA | F and m; 7-18 years; overweight + obesity; other conditions: NR; N = 1790. | 23 | NR | Children and adolescents | Aerobic 8-36 weeks; Strength 12-22 weeks; Concurrent 10-22 weeks; |

| Study | Country | Design | Participants | Included RCTs (n) | Setting | Who received the intervention | Intervention(s) |
|---|---|---|---|---|---|---|---|
| Hale et al., 2023 | United Kingdom | SR | F and M; 6-11 years; Normal weight + Overweight + Obesity; Other condition = NR; n = 1392. | 23 | School | Children | Aerobics 9-35 weeks; Aerobics + football 20-26 Weeks; Aerobics + sports and fitness skills 26 weeks; |
| Casas et al., 2018 | Argentina | SRMA | F and M; 6-18y; Normal weight + overweight/obesity; Other health condition= No; n= 1135 | 22 | NR | Children and adolescents | PA (resistance exercises); 1 - 3 days/wk, 10 – 40'/day, 50-100% 1RM, 6 to 60 weeks |
| Ribeiro et al., 2022 | Portugal | SRMA | F and M; 10,9 – 17,7 years; obesity; other conditions: NR; n = NR. | 21 | NR | Adolescents | Isolated Resistance training (RT) program 4-48 weeks; |
| Dias et al., 2021 | Brazil | SRMA | F and M; 5-18 years; Obesity; Other condition: NR. N = 398. | 19 | NR | Children and adolescents | Resistance training 12 weeks; Sport 12 weeks. |
| García-Hermoso et al., 2020-c | Spain | SRMA | F and M; 1-6 years; Overweight + Obesity + Normal weight; Other conditions: No; n = 7,843 | 19 | NR | Children and parents | PA interventions: 10-16 weeks; Active lifestyle, quick walking, slow running, jumping, rope skipping, semi-squating and slow crawling. |
| Li and Chen et al., 2021 | China | SRMA | F and M; 8-17 years; Obesity; other condition = no. n = 704. | 19 | NR | Children and adolescents | MIIT for 12-20 weeks; Continuous aerobic 10-12 weeks; HIIT 8-12 weeks; Combined (aerobic + strength) 8-12 weeks; Strength 8 weeks; Yoga 8 weeks; Soccer 12 weeks; |

| Study | Country | Design | Participants | Included RCTs (n) | Setting | Who received the intervention | Intervention(s) |
|---|---|---|---|---|---|---|---|
| | | | | | | | Rope jumping 12 weeks; Control 8-20 weeks. |
| Collins et al., 2018 | United Kingdom | SRMA | F and M; 8-16y; Normal weight + overweight/obesity; Other health condition= NR; n= 1153 | 18 | School; Community | Children and adolescents | PA (resistance exercises); 2 – 6 days/wk, 10 – 90'/day, 50 – 100% 1RM, 6 to 24 weeks |
| Costa et al., 2020 | Brazil | SRMA | F and M; 7-17 years; Normal weight + overweight + obesity; Other conditions: NR; n = 698; | 18 | NR | Children and adolescents | Control 8-12 weeks; HIIT 12 weeks; Endurance, mixed practices and strength 10-24 weeks; Rope interval 6-8 weeks; Endurance bike: 8 weeks; Mixed Sports and Activities 10 weeks; Combined 16 weeks and others. |
| Martin-Smith et al., 2020 | United Kingdom | SRMA | F and M; 10,7 – 17 years; Obesity and Overweight; Other conditions: NR; n = NR | 18 | NR | Adolescents | HIIT/Sprint interval training 5-15 weeks. |
| Cao et al., 2019 | China | SRMA | F and M; 6-17 years; Normal weight + overweight + obesity; other conditions: NR; n = 563. | 17 | School and clinical; | Children and adolescents | HIIT 6-24 WEEKS; MICT 6-24 WEEKS; |
| Clemente et al., 2022 | Portugal | SR | F and M; 8,9-18,2; Normal weight + Obesity; other conditions: metabolic syndrome; n = NR. | 17 | NR | Children and adolescents | Soccer 8-40/43 weeks. Training duration varied between 12 and 90 min. |

| Study | Country | Design | Participants | Included RCTs (n) | Setting | Who received the intervention | Intervention(s) |
|---|---|---|---|---|---|---|---|
| da Rosa-Santos et al., 2019 | Brazil | SR | F and M; 12-19 years; Overweight; Other conditions = NR; n = 20 | 17 | NR | Adolescents | Combined resistance and Aerobics 10 weeks; Resistance 12 weeks |
| Recchia et al., 2023 | China | SRMA | F and M; 8-19 years and 9-15 years(obesity group); Other conditions = diabetes; n = 2441. | 17 | NR | Children and adolescents | Aerobic training for 4-144 weeks. |
| Schwartz et al., 2017 | France | SRMA | F and M; 5-18y; Overweight/obesity; Other health conditions= NR; n= 549 | 16 | NR | Children and adolescents | PA (aerobic, resistance and combined exercises); 2 – 3 days/wk, 16 – 60'/day, 10 weeks to 12 months |
| Comeras-Chueca et al., 2021 | Spain | SRMA | F and M; 6-18 years; Overweight + Obesity; Other conditions: No; n = 1,232 | 15 | NR | Children and adolescents | Active Video Games: 8-24 weeks; Xbox 360 with Kinect, Nintendo Wii, Sony PlayStation 2, dance mats, and interactive video game cycling. Games included Just Dance, Wii Fit, Wii Sports, Kinect Adventures, Kinect Sport, Dance Central, Dance Dance Revolution (DDR), and EyeToy. |
| Deshira et al., 2022 | United States of America | SR | F and M; 6-15 years; Overweight/Obesity; Other conditions = NR. n = NR. | 15 | Park and Camp | Children and adolescents | Fit2Play; Health lifestyle fitness. 6-43 WEEKS. |

| Study | Country | Design | Participants | Included RCTs (n) | Setting | Who received the intervention | Intervention(s) |
|---|---|---|---|---|---|---|---|
| Mears and Jago., 2016 | United Kingdom | SRMA | F and M; 5-18y; Normal weight + overweight/obesity; Other health conditions= NR; n= 3547 | 15 | School; Home | Children and adolescents | PA (general sports; games; jumping rope; dance; aerobic exercises; active commuting); PA + Diet; 2 - 7 days/wk, 60 – 135'/day |
| Thivel et al., 2018 | France | SRMA | F and M; 8-18years; Obesity and Overweight; Other conditions: NR. N = NR. | 15 | NR | Children and adolescents | HIIT 4-24 weeks; |
| Wang et al., 2022 | China | SRMA | F and M; 6-18 years; Overweight + obesity; Other conditions: NR; n = 1134. | 15 | NR | Children and adolescents | Aerobic exercise 12-43 years; Resistance Exercise 12-43; Combined Exercise (aerobic + resistance) 12-43 years; HIIT 12 weeks; |
| Zhao et al., 2022-a | China | SRMA | F and M, 10-20 years; Obesity; Other conditions: NR; n = 307. | 15 | NR | Children and adolescents | Exercise Only(aerobic) 6-24 weeks; Exercise training 4-52 weeks; Diet 4 weeks-1year. |
| Bento et al., 2022 | Spain | SR | F and M; 10-19 years; Normal weight + overweight + obesity; Other conditions = NR; n=NR. | 14 | School | Children and adolescents | Aerobic 8 weeks; HIIT 7-12 weeks; MIIT 12 weeks; LIIE 12 weeks. |
| García-Hermoso et al., 2017-a | Chile | SRMA | F and M; 6-17y; Overweight/obesity; Other health condition= NR; n=347 | 14 | NR | Children and adolescents | PA (combined exercises); 2 – 5 days/wk, 10-90'/day; 60-110% VO2peak, 6 to 24 weeks |
| Hejazi et al., 2022 | Iran | SRMA | F and M; 10,5-16,6y; Overweight/Obesity; Other | 14 | NR | Children and adolescents | Combined training; aerobic training; Interval training, P+HIIT. |

| Study | Country | Design | Participants | Included RCTs (n) | Setting | Who received the intervention | Intervention(s) |
|---|---|---|---|---|---|---|---|
| | | | Health conditions = NR. n = 468. | | | | 12 weeks; 8-39 weeks. |
| Hernandez-Martin et al., 2021 | Spain | SRMA | F and M; 9-12 years; Normal weight + obesity; Other condition = NR; n = 1643. | 14 | NR | Children and adolescents | Control 20-24 weeks; Football Training 12-24 weeks; |
| Zhao et at., 2022-b | China | SRMA | F and M; 13-18 years; Obesity; other conditions: NR; n = NR. | 14 | NR | Adolescents | Aerobic training 6-24 weeks; Aerobic training + resistance training; 8-24 weeks; Resistance training 8-22 weeks; HIIT 12 WEEKS; |
| Busnatu et al., 2022 | Romania | SRMA | F and M; 6-18 years; Overweight; Other conditions: NR; n: 479. | 12 | Treadmill, walking-jogging, running sprints, weight machines and free weight | Children and adolescents | Aerobic training 4-24 weeks; Strength training 12-24 weeks; Multicomponent 12 weeks. |
| Cao et al., 2021 | China | SRMA | F and M; 10-16 years; Overweight + obesity; other conditions: NR. N= 325. | 12 | School and lab | Children and adolescents | HIIT 6-24 weeks; MICT: moderate-intensity continuous training 6-24 weeks; |
| García-Hermoso et al., 2016-b | Chile | SRMA | F and M; 11-18y; Overweight/obesity; Other health condition= NR; n=555 | 12 | NR | Children and adolescents | PA (aerobic, resistance and combined exercises); 2-3 days/wk, 20 - 60’/day, 10 to 48 weeks |
| Jung et al., 2018 | United States of America | SRMA | F and M; 6 – 19 years; Obesity; other conditions: NAFDL, metabolic Syndrome; n = nr. | 12 | NR | Children and adolescents | Aerobic training 12-52 weeks; Resistance Training 12-21 weeks; |

| Study | Country | Design | Participants | Included RCTs (n) | Setting | Who received the intervention | Intervention(s) |
|---|---|---|---|---|---|---|---|
| Kelley and Kelley., 2008 | United States of America | SRMA | F and M; 8-16y; Normal weight + overweight/obesity; Other health condition= type 1 diabetes; n= 404 | 12 | NR | Children and adolescents | PA (aerobic exercises): 3 – 5 days/wk, 20 – 135’/day, 44 - 90% VO2max, 5 to 16 weeks |
| Cesa et al., 2014 | Brazil | SRMA | F and M; 6-12y; Normal weight + overweight/obesity; Other health condition= NR; n= 10748 | 11 | School; Out of school | Children and parents | PA (increased time in PA physical education classes and/or extracurricular activities); 2 - 6 days/wk, 20 – 110’/day; 6 months to 3 years |
| Collins et al., 2019 | United Kingdom | SRMA | F and M; 5-18 years; Normal weight + Overweight + obesity; Other conditions NR; n = 256. | 11 | School and Community | Adolescents | Control time NR; Strength training 8-24 weeks. |
| Da Silva et al., 2020 | Brazil | SR | F and M; adolescents; age: NR; Overweight + Obesity; Other conditions: NR; n = 169 | 11 | NR | Adolescents | HIIT: 12-26 weeks; Ergometer rowling, cycle ergometer, outdoor all-out shots. |
| Eckstein et al., 2022 | Germany | SR | F and M; 10-15.2 years; Normal weight + obesity; Other conditions: DM1, metabolic disease. N = 472. | 11 | After school activity program at an indoor gym; Training sessions were conducted at the university and PE at school; hospital setting. | Children and adolescents | Taekwondo for 16 weeks; judo 60min judo sessions; kung fu 24 weeks; tai chi 24 weeks. |

| Study | Country | Design | Participants | Included RCTs (n) | Setting | Who received the intervention | Intervention(s) |
|---|---|---|---|---|---|---|---|
| Han et al., 2019 | China | SRMA | F and M; 7-17 years; overweight + obesity; other conditions: NR; n = 623. | 11 | NR | Children and adolescents | Physical Activity (soccer, aerobic training, resistance training, combined training, after-school exercise, dance, jump roping, stationary cycling) 5-26 weeks. |
| Solera-Martinez et al., 2021 | Spain | SRMA | F and M; 5-12 years; Obesity and overweight; Other conditions: NR. N = 512. | 11 | Cycling and Shuttle runs. | Children | HIIT 6-12 weeks. |
| Estéves-González et al., 2022 | Spain | SRMA | F and M; Age: NR; Obesity; Other conditions; Diabetes Mellitus 1; n = 252. | 10 | NR | Children and adolescents | Aerobic training: 6-72weeks; Resistance training 12 weeks; Combined aerobic and resistance training 6 weeks. |
| Laframboise et al., 2011 | Canada | SR | F and M; 6.5-16y; Normal weight + overweight/obesity; Other health condition= type 1 diabetes; n=2521 | 10 | NR | Children and adolescents | PA (aerobic exercises); 1 – 5 days/wk, 30 – 90'/day, 50-90% FCmáx, 8 weeks to 8 months |
| Liu, W et al., 2020 | China | SR | F and M; 6-12 years; overweight + obesity; other conditions: NR; N= NR. | 10 | School and lab | Children | Active video game(Nintendo Wii and Xbox Kinect, and one study developed their own purpose-built AVG)s 6-12 weeks; |
| Valeriani et al., 2021 | Italy | SR | F and M; 0-18 years; Obesity and overweight; | 10 | Home-based Scholl-based clinics | Children and adolescents | Exergames 6-40weeks; |

| Study | Country | Design | Participants | Included RCTs (n) | Setting | Who received the intervention | Intervention(s) |
|---|---|---|---|---|---|---|---|
| | | | other conditions: NR; n = NR. | | | | |
| Andrade et al., 2019 | Brazil | SRMA | F and M; 6-19 years; Overweight and Obesity; Other condition = NR. n = 336. | 9 | Laboratory, laboratory and school, community Center, | Children and adolescents | Control 6-20 weeks; Exergames 6-20 weeks; Competitive games; Cooperative games; Several games, Wii, Xbox, Dance Revolution, ranging from 30 - 60 min of activity for 10 weeks, 3 times per week; Exergame + physical activity 1 time per week, for 10 weeks. |
| García-Hermoso et al., 2014 | Spain | SRMA | F and M; 6-18y; Overweight/obesity; Other health condition = NR; n=367 | 9 | NR | Children and adolescents | PA (sports; walking/running; ergometer-based; jump rope); 3 – 6 days/wk, 30 – 90/day, 66-75% VO2max, 6 to 36 weeks |
| García-Hermoso et al., 2015 | Chile | SRMA | F and M; 6-18y; Overweight/obesity; Other health condition= NR; n=365 | 9 | NR | Children and adolescents | PA (aerobic and combined exercises; sports); 2 – 4 days/wk, 45 – 90’/day, 55-65% VO2max, 8 to 24 weeks |
| García-Hermoso et al., 2016-a | Chile | SRMA | F and M; 6-17y; Overweight/obesity; Other health condition= NR; n=274 | 9 | NR | Children and adolescents | PA (aerobic exercises); 2 – 4 days/wk, 18 – 45’/day, 80 – 100% VO2max, 4 to 24 weeks |

| Study | Country | Design | Participants | Included RCTs (n) | Setting | Who received the intervention | Intervention(s) |
|---|---|---|---|---|---|---|---|
| García-Hermoso et al., 2016-c | Chile | SRMA | F and M; 6.7 - 17.1y; Overweight/obesity; Other health condition= NR; n=427 | 9 | NR | Children and adolescents | PA (resistance and aerobic exercises; sports); 2 – 6 days/wk, 20 – 90'/day, 55 - 85% VO2max, 6 to 24 weeks |
| Jago et al., 2004 | United States of America | SR | F and M; 5-18y; Normal weight + overweight/obesity; Other health condition= NR; n= 919 | 9 | School; Camp; Clubs | Children and adolescents | PA (increased PA during school breaks, travel to school and others extracurricular time): 4 weeks to 5 months |
| Liu et al., 2020 | China | SRMA | F and M; 10-16 years; Obesity; Other conditions: NR; n = 309 | 9 | NR | Children and adolescents | HIIT: 4-12 weeks. MICT: 4-12 weeks |
| Saavedra et al., 2011 | Spain | SRMA | F and M; school age; Overweight/obesity; Other health conditions= NR; n= 349 | 9 | NR | Children | PA (aerobics, resistance and combined exercises; sports); 3-5 days/wk, 29-90'/day, 8 to 36 weeks |
| Farah et al., 2012 | Brazil | SR | F and M; 10-17y; Overweight/obesity; Other health condition= NR; n=760 | 8 | NR | Adolescents | PA (aerobic and combined exercises); 1 – 6 days/wk, 40 – 90'/day, 55 - 95% HRmax and 55 – 75% 1RM, 8 to 48 weeks |
| Sirico et al., 2018 | Italy | SRMA | F and M; 18y or younger; Overweight/obesity; Other health conditions= NR; n= 250 | 7 | NR | Children and adolescents | PA (aerobic and combined exercises); 3 days/wk, 20 – 60'/day; 6 to 20 weeks |

| Study | Country | Design | Participants | Included RCTs (n) | Setting | Who received the intervention | Intervention(s) |
|---|---|---|---|---|---|---|---|
| Delgado-Floody et al., 2019 | Chile | SR | F and M; 9-16 years; Overweight/Obesity; Other Health conditions = NR. n = 136. | 6 | NR | Children and adolescents | HIIE, LIIE, CON, MIIT, P+ HIIT, HIIT. 6 Weeks; 6-26 weeks, 12 weeks. |
| Dias et al., 2015 | Australia | SRMA | F and M; 7-15.8y; Overweight/obesity; Other health condition= NR; n= 219 | 6 | NR | Children and adolescents | PA (combined exercise): 2 – 5 days/wk, 30 – 75'/day, 50 – 80% VO2peak and 55% to 70% 1RM (only one study specified the intensity of the resistance exercises), 6 to 12 weeks |
| García-Hermoso et al., 2017-b | Chile | SRMA | F and M; 6-18y; Overweight/obesity; Other health condition = NR; n=303 | 6 | NR | Children and adolescents | PA (aerobic, resistance and combined exercises); 3 days/wk, 60–70'/day, 60–75% VO2peak and 60% 1RM, 14 to 24 weeks |
| Littlewood et al., 2020 | Australia | SR | F and M; 2-17 years; Obesity; Other conditions: NR; n = 6629 | 6 | Home Environment | Children and adolescents | Switch for 20 weeks. |
| Oliveira et al., 2017 | Portugal | SRMA | F and M; 8-17y; Overweight/obesity; Other health conditions = NR; n= 141 | 6 | NR | Children and adolescents | PA (sports); 2 – 4 days/wk, 45 - 90 min/day, 50 - 80% FCMax, 3 to 6 months |
| **Reviews Including Physical Activity Combined with Other Interventions** | | | | | | | |
| Brown et al., 2019 | United Kingdom | SRMA | F and M; 0-18 years; Obesity + overweight; Other conditions: No ; n = NR. | 153 | Home, schools, childcare, healthcare, community | Children, Adolescents and Parents | Physical Activity + Nutritional components 52-104 weeks; usual care 52-104 weeks. |

| Study | Country | Design | Participants | Included RCTs (n) | Setting | Who received the intervention | Intervention(s) |
|---|---|---|---|---|---|---|---|
| Luckner et al., 2012 | United Kingdom | SRMA | F and M; 6-18y; Normal weight + overweight/obesity; Other health conditions= NR; n=41419 | 103 | School; Out of school | Children, Adolescents and Parents | PA (reduction of screen time); Educational (health lifestyle); Diet; Educational + PA; Educational + Diet; PA + Educational + Diet; 1 to 90 months |
| Birch et al., 2019 | United Kingdom | SR | F and M; 4-19 years; Obesity; other conditions = yes. n = 8 to 203. | 98 | Clinic (Hospital) Academic Institution; Community | Children and adolescents | Exercise + diet 12-24 weeks |
| Oosterhoff et al., 2016 | Netherlands | SRMA | F and M; 4-12y; Normal weight + overweight/obesity; Other health conditions= (pre)hypertension; n= 72934 | 85 | School | Children and parents | Educational (materials and/or lectures about dietary and activity behaviors); PA (increase of physical education load, reduction sedentary time) + Educational; Diet (changes in foods provided by school) + Educational; PA + Diet; PA+ Diet + Educational; 1.5 to 96 months |

| Study | Country | Design | Participants | Included RCTs (n) | Setting | Who received the intervention | Intervention(s) |
|---|---|---|---|---|---|---|---|
| Feng et al., 2017 | China | SRMA | F and M; 6-19y; Normal weight + overweight/obesity; Other health condition= NR; n= 72620 | 76 | School | Children and adolescents | PA (increased PA intensity and duration within and out of school); Educational (lessons, workshops, materials about health at school); Diet (modification of school lunches); Others (weight management/ school policies / psychological counseling / infrastructure support); 3 months to 4 years |
| Mead et al., 2017 | United Kingdom | SRMA | F and M; 6-11 years; Obesity + overweight; Other conditions: asthma, type 2 diabetes, metabolic syndrome, depression, anxiety and fatty liver diagnoses ; n = 8461. | 70 | Primary care, secondary care, university research clinics, community, home and schools | Children and parents | Nutritional + Physical Activity + Behavioral components 2-104 weeks; usual care 2-104 weeks. |
| Staniford et al., 2011 | United Kingdom | SR | F and M; 5-18y; Overweight/obesity; Other health conditions = NR; n= NR | 61 | NR | Children, Adolescents and Parents | Others (multiple health behaviors including nutrition, PA and/or sedentary behaviors coupled with behavioral change/modification strategies and incorporated family involvement); 10 to 52 weeks |

| Study | Country | Design | Participants | Included RCTs (n) | Setting | Who received the intervention | Intervention(s) |
|---|---|---|---|---|---|---|---|
| Menjie et al., 2022 | China | SRMA | F and M; 5-19 years; Obesity Other conditions – NR; n = 2995. | 47 | NR | Children and adolescents | Control with diet 12 weeks; Control without physical activity 12-16 weeks; Control or usual training/activities 8-48 weeks; Aerobics 12-16 weeks; Diet + HIIT 12 weeks; HIIT 12 weeks; Equipment exercise 8 weeks; Judo + aerobics 12 weeks; Aerobic training nr; Plyometrics 12 weeks; Physical Activity 6 weeks and others. |
| Al-Khudairy et al., 2017 | United Kingdom | SRMA | F and M; 12-17 years; Obesity + overweight; Other conditions: NR ; n = 4781. | 44 | Schools, community, home, research clinics and hospitals | Adolescents and parents | Nutritional + Physical Activity + Behavioral components 26-104 weeks; usual care 26-104 weeks. |
| Dobbins et al., 2013 | Canada | SR | F and M; 6-18y; Normal weight + overweight/obesity; Other health condition= No; n=36593 | 44 | School; Home; Community | Children, Adolescents and Parents | Educational (material and counseling about self-esteem, nutrition, physical activity and motivational strategies); Educational + PA (increased PA in physical education classes and at home); 12 weeks to 6 years |

| Study | Country | Design | Participants | Included RCTs (n) | Setting | Who received the intervention | Intervention(s) |
|---|---|---|---|---|---|---|---|
| Seral-Cortes et al., 2021 | Spain | SRMA | F and M; 6-12 years; normal weight + obesity; other conditions: diabetes Mellitus II; n = NR. | 41 | School and community center. Medical center; | Children | PA, D and BS, 14-168 Weeks; PA and BD for 24-192; PA and D 12-96 weeks; D and BS for 72 weeks; D 6-144 weeks; PA 6-144 weeks; BS 24-96weeks. |
| Schranz et al., 2013 | Australia | SRMA | F and M; 6-18y; Overweight/obesity; Other health conditions= NR; n= NR | 40 | NR | Children and adolescents | PA (resistance and combined exercises); PA + Educational (nutrition and exercise education); PA + Others (behavioral therapy); 3–5 days/wk, 50-92%1RM and 60-80% VO2ma, 6 to 52 weeks |
| Guerra et al., 2014 | Brazil | SRMA | F and M; 6-18y; Normal weight + overweight/obesity; Other health condition= No; n=35690 | 38 | School | Children, Adolescents and Parents | Educational (Diet and PA lessons/support; school food service changes); PA (dance classes; reduction screen time); Educational + PA; 8 weeks to 6 years |
| Spadaccini et al., 2022 | Italy | SR | F and M; 6-19 years; Obesity; other conditions: NR; n = 5510. | 36 | Health clinics | Children, Adolescents and Parents | Dietary intervention + physical Activity intervention + psychotherapy. 5,4-43 weeks. |

| Study | Country | Design | Participants | Included RCTs (n) | Setting | Who received the intervention | Intervention(s) |
|---|---|---|---|---|---|---|---|
| Millard-Stafford et al., 2013 | United States of America | SR | F and M; 8.9 - 16.9y; Overweight/obesity; Other health condition= NR; n= 1578 | 33 | NR | Children and adolescents | PA (sports; games; tai chi chuan; yoga; aerobic, resistance and combined exercises); PA + Diet (restrictions); PA + Educational (lifestyle related to nutrition, physical activity or behavior change); PA + Diet + Educational; 80 – 420'/wk, 6 to 36 weeks |
| Albornoz-Guerrero et al., 2021 | Chile | SR | F and M. 6-12 years; Overweight + obesity other conditions: NR. n = 4434. | 29 | health centers, educational centers, university centers, community centers. | Children and parents | Physical activity and nutrition 26-52weeks; Education 26-52 weeks; Behavioral and education 26-52weeks; physical activity 26-52 weeks. Behavioral and nutrition 2-52 weeks; physical activity and behavioural 2-52 weeks; Physical activity and behavioral and education and nutrition 26-52 weeks; physical activity and behavioural and nutrition 26-52 weeks. |
| Brown et al., 2015 | United Kingdom | SRMA | F and M; School age; Normal weight + overweight/obesity; Other health conditions= type 2 | 29 | General practice; Community; Home; Hospital; School; University and workplace | Children and adolescents | PA; PA + Diet; 3 to 8 months. *There is no additional information on interventions |

| Study | Country | Design | Participants | Included RCTs (n) | Setting | Who received the intervention | Intervention(s) |
|---|---|---|---|---|---|---|---|
| | | | diabetes and metabolic syndrome; n= 11527 | | | | |
| Boff et al., 2017 | Brazil | SR | F and M; 10-19y; Overweight/obesity; Other health condition= NR; n= 2099 | 26 | Clinics; Homes; Schools | Adolescents and parents | Educational (PA and/or nutrition education); PA (aerobic and resistance exercises; games; yoga); 10 weeks to 24 months |
| Sbruzzi et al., 2013 | Brazil | SRMA | F and M; 6-12y; Normal weight + overweight/obesity;Other health conditions= hypertension, dyslipidemia; n= 23617 | 26 | NR | Children and parents | Educational (multicomponent interventions designed to promote behavior changes to prevent or treat obesity); 6 to 48 months |
| Lopes et al., 2019 | Brazil | SRMA | F and M; 7-19 years; Normal weight + overweight + obesity; Other conditions = NR; n = 1199. | 25 | NR | Children and adolescents | Control group + nutritional Guidance 12 weeks; Control group 12 weeks; Continuous aerobic 48 weeks; HIIT + nutritional 12 weeks; Continuous Aerobic 12 weeks; Nutritional + exercise 12 weeks and others. |
| Verjans-Janssen et al., 2018 | Netherlands | SR | F and M; 4-12y; Normal weight + overweight/obesity; Other health conditions = NR; n=39101 | 25 | School | Children, Adolescents and Parents | Others (changes to the school's policies or physical and social environment or budget for implementing PA and diet activities); 10 weeks to 4 years |

| Study | Country | Design | Participants | Included RCTs (n) | Setting | Who received the intervention | Intervention(s) |
|---|---|---|---|---|---|---|---|
| Vasconcellos et al., 2014 | Brazil | SR | F and M; 12-17y; Overweight/obesity; Other health conditions = NR; n=1635 | 24 | NR | Adolescents | PA (aerobic, resistance and combined exercises; sports; dance); PA + Diet or Educational; 2 – 5 days/wk, 16 -90'/day, 7 to 52 weeks |
| Bondyra-Wisniewska et al., 2021 | Poland | SR | F and M; 6-18 years; Obesity and Overweight; Other conditions: NR. N = 1587. | 23 | NR | Children and adolescents | Nutritional Intervention 4weeks-2years; Physical Activity 10 weeks – 2 years. |
| Silva et al., 2014 | Spain | SR | F and M; 12-19y; Overweight/obesity; Other health conditions= NR; n= 2501 | 23 | School | Adolescents and parents | Educational (material and counseling about healthy eating, increased PA levels and reduction of screen time); 2 - 5 days/wk, 45 -90'/day, 12 weeks to 5 years |
| Gonzalez-Suarez et al., 2009 | Spain | SRMA | F and M; 11+ y; Normal weight + overweight/obesity; Other health condition = NR; n=NR | 22 | School | Children, Adolescents and Parents | Educational (lessons about changes in nutrition, PA and hours spent in TV viewing) + PA (increased time and intensity of physical education classes); 6 months to 2 years |
| Mclean et al., 2003 | United Kingdom | SR | F and M; 6-16y; Overweight/obesity; Other health condition= type 2 diabetes; n=763 | 21 | Home | Children, Adolescents and Parents | Others (family based interventions changing food intake and physical activity); 9 to 104 weeks |

| Study | Country | Design | Participants | Included RCTs (n) | Setting | Who received the intervention | Intervention(s) |
|---|---|---|---|---|---|---|---|
| Choe et al., 2022 | United States of America | SRMA | F and M; 4-16 Years; Normal weight + overweight + obesity; Other condition: NR; n = 768. | 19 | School | Children and adolescents | Physical Activity + Diet 5-16 years; Physical Education + Health Education 12 weeks; Exercise 12-16 weeks. |
| Yuksel et al., 2020 | Turkey | SR | F and M; 4-15 years; Normal weight + obesity. Other conditions: NR. n = 6000. | 19 | School | Children and adolescents | Lifestyle intervention for 1 year. Intervention 52-260 weeks;Child health weight 10 weeks; Multicomponent intervention 86 weeks. School based PA intervention 12-260 weeks; Comprehensive school health intervention 208 weeks. |
| Frerichs et al., 2016 | United States of America | SR | F and M; 10-18y; Normal weight + overweight/obesity; Other health condition= NR; n=14278 | 18 | School; Home; Community | Children and adolescents | Educational (PA and diet educational material; environmental and behavioral changes); 5 weeks to 3 years |
| Harris et al., 2009 | Canada | SRMA | F and M; 5-18y; Normal weight + overweight/obesity; Other health condition= NR; n= 18141 | 18 | School | Children, Adolescents and Parents | PA (increased PA and exercise levels); PA + Educational (nutrition or health education); 2 – 5 days/week, 10 – 90'/day, 6 months to 3 years |

| Study | Country | Design | Participants | Included RCTs (n) | Setting | Who received the intervention | Intervention(s) |
|---|---|---|---|---|---|---|---|
| Kim et al., 2017 | United States of America | SRMA | F and M; 8-16y; Normal weight + overweight/obesity; Other health condition= NR; n=1777 | 18 | NR | Children and adolescents | PA (recreational sports, martial arts, aerobic and resistance exercises); PA + Diet (diet prescription); 4 weeks to 2 years |
| Mei et al., 2016 | China | SRMA | F and M; 6-18y; Normal weight + overweight/obesity; Other health conditions= NR; n= 22381 | 18 | School | Children and adolescents | PA (increased PA level in and outside school); PA + Educational (health education); 2–5 days/wk, 20 – 100'/wk, 1 month to 6 years |
| Eberl et al., 2019 | Germany | SR | F and M; 8-12 years; Normal weight + overweight; Other conditions = NO. n = NR. | 17 | NR | Children and adolescents | Football group 13-26 weeks; NUT, Nutrition intervention group NR; Control 13-26 weeks. |
| Azevedo et al., 2023 | United Kingdom | SRMA | F and M; 3-16 years; Normal weight + overweight + obesity; Other condition = NR; n = 2.352. | 16 | Home | Children, Adolescents and Parents | Control/ usual care 24-48 weeks; Educational contend/ counseling 24-48 weeks. |
| Snethen et al., 2016 | United States of America | SRMA | F and M; 6-15y; Overweight/obesity; Other health condition= NR; n= 2317 | 16 | NR | Children | PA; Diet; PA + Diet; Educational; Diet + Educational; Others (behavioral).<br>*There is no additional information on interventions |

| Study | Country | Design | Participants | Included RCTs (n) | Setting | Who received the intervention | Intervention(s) |
|---|---|---|---|---|---|---|---|
| Godin et al., 2015 | Canada | SR | F and M; Elementary and secondary school age; Normal weight + overweight /obesity; Other health condition= NR; n=NR | 15 | Elementary school in rural and remote communities or reserves | Children, Adolescents and Parents | Educational (diet; diabetes knowledge; healthy eating; PA) |
| Ho et al., 2013 | Australia | SRMA | F and M; 5-18y; Overweight/obesity; Other health condition= NR; n= 879 | 15 | School; Hospital; Community | Children, Adolescents and Parents | Diet (caloric/food restriction); PA (aerobic, resistance and combined exercises); PA + Diet; 6 weeks to 6 months |
| Voskuil et al., 2017 | United States of America | SR | F; 6-18y; Normal weight + overweight/obesity; Other health conditions= NR; n= 14120 | 15 | NR | Children and adolescents | Educational (PA and/or diet counseling/information); PA (exercise or sports); Educational + PA; 2 – 5 days/wk, 20 – 90'/day, 12 weeks to 2 years |
| Whitlock et al., 2010 | United States of America | SRMA | F and M; 4-18y; Overweight/obesity; Other health conditions= NR; n= 2807 | 15 | Health primary care | Children and adolescents | Educational (PA and/or diet counseling/information); Pharmacological (drug administration); Educational + Pharmacological; 24 weeks to 1 year |
| González-Ruiz et al., 2017 | Chile | SRMA | F and M; 6-18y; Overweight/obesity; Other health condition= NR; n= 1231 | 14 | NR | Children and adolescents | PA (aerobic and resistance exercises); PA + Educational (nutrition/behavior education); 2- 5 days/wk, 20'–60'/day, 10 to 96 weeks |

| Study | Country | Design | Participants | Included RCTs (n) | Setting | Who received the intervention | Intervention(s) |
|---|---|---|---|---|---|---|---|
| Hamel et al., 2011 | Ireland | SR | F and M; 8-18y; Normal weight + overweight/obesity; Other health condition= NR; n=6226 | 14 | School; Home; Camp/troop | Children, Adolescents and Parents | Educational (computer and web-based interventions to increase PA alone or in combination with a dietary intervention); 2 weeks to 2 years |
| Kropski et al., 2008 | United States of America | SR | F and M; 4-14y; Normal weight + overweight/obesity; Other health conditions= NR; n=43130 | 14 | School; School and home | Children, Adolescents and Parents | Educational (lesson about reducing carbonated beverages and benefits of a healthy diet); PA (increase intensity in physical education lessons; reduction of screen time; family games); PA + Diet (reduction of energy from fat; changes in school lunches); 3 days/wk, 6 months to 4 years |
| Ruotsalainen et al., 2015 | Finland | SR | F and M; 10-18y; Overweight/obesity; Other health conditions= No; n= 1321 | 14 | Home; Home and community; Schools; Hospitals | Adolescents | PA (supervised exercise); Others (behavioral management skills; material and information support; social support); PA + Others; PA + Diet (Dietary regimen or restriction); 8 to 24 weeks |

| Study | Country | Design | Participants | Included RCTs (n) | Setting | Who received the intervention | Intervention(s) |
|---|---|---|---|---|---|---|---|
| Sims et al., 2015 | United Kingdom | SRMA | F and M; 5-18y; Overweight/obesity; Other health conditions= NR; n= 8687 | 14 | School; community | Children, Adolescents and Parents | Educational (healthy eating and PA lessons/counseling; behavior modification); PA (increased time in physical education classes); PA + Educational; 4 weeks to 3 years |
| Mijalkovic et al., 2022 | Serbia | SR | F and M; 6-10 years; Obesity; Other conditions: NR. n = 2810. | 13 | School | Children | AL - Additional lessons, 26 weeks (60min). Kids4Fit-Multidisciplinary weight reduction program 12-156weeks (2 x week; 2 x weeks (20min). |
| Stoner et al., 2016 | New Zealand | SRMA | F and M; 10-19y; Overweight/obesity; Other health conditions = prehypertension; n= 556 | 13 | School; Hospital; University | Adolescents | PA (aerobic, resistance and combined exercises); Educational (nutritional and PA behavior changes); PA + Educational; 2-3 days/wk, 60 – 90'/day, 8 to 36 weeks |
| Vissers et al., 2016 | Belgium | SRMA | F and M; 7-19y; Overweight/obesity; Other health conditions = NAFLD; n= 782 | 13 | NR | Children and adolescents | Diet (food/calorie restriction); PA (aerobic, resistance and combined exercises):55 – 75% VO2peak, 60% 1RM, 120 – 200'/wk; Diet + PA; 3 months to 1 year |

| Study | Country | Design | Participants | Included RCTs (n) | Setting | Who received the intervention | Intervention(s) |
|---|---|---|---|---|---|---|---|
| Chen et al., 2021 | China | SRMA | F and M; 8-10 (mean); Overweight + Obesity; Other condition: NR. N = 504. | 12 | NR | Children | Aerobic Exercise 8-12 weeks; Resistance Exercise 12 weeks; Combined Exercises (AE+ RE) 16 weeks; Aerobic Exercise + Diet 16 weeks; Control 8-16 weeks. |
| Guerra et al., 2013 | Brazil | SRMA | F and M; 6-16y; Normal weight + overweight/obesity; Other health condition= NR; n= NR | 12 | School | Children and adolescents | PA (sports; multiple exercises): 75 - 270'/wk, moderate intensity; Educational (PA and nutrition education); 2 weeks to 48 months |
| Wolfa et al., 2019 | Brazil | SR | F and M; 10-17 years; Normal weight + overweight + obesity; Other conditions: NR. N = NR. | 12 | School | Adolescents | Educational intervention 10-20 weeks; educational + nutritional counseling; Educational + physical activity 12-104 weeks. |
| Shin et al., 2019 | Korea | SRMA | F and M; 10-19 years; Normal weight + obesity+ overweight; Other conditions: NR. n = 1472. | 11 | NR | Adolescents | Use of mobile phone: personalized SMS 24 weeks."switch-off 4 healthy minds" 6month24weeks. Use of mobile phone 3mont12weeks. Apps for improving fitness for 8 weeks. And others. |
| Zhu et al., 2021 | China | SRMA | F and m; 7-16 years; Obesity and overweight; | 11 | NR | Adolescents | HIIT for 6-12 weeks; Blank control 6-12 weeks; Dietary |

| Study | Country | Design | Participants | Included RCTs (n) | Setting | Who received the intervention | Intervention(s) |
|---|---|---|---|---|---|---|---|
| | | | other conditions = no. n= 488. | | | | intervention 12 weeks; Walking 12 weeks; |
| Jurado-Castro et al., 2020 | Spain | SRMA | F and M; 9 ± 1.57 years; Obesity; Other condition: NR. N = 478. | 10 | NR | Children and adolescents | Multicomponent intervention 12-104 weeks; Active video game 24 weeks; |
| Upton et al., 2014 | United Kingdom | SR | F and M; 4-16y; Overweight/obesity; Other health conditions= NR; n= 1002 | 10 | Community | Children, Adolescents and Parents | Others (Family-based intervention for weight-management); 12 weeks to 1 year |
| Burns et al., 2019 | United States of America | SRMA | F and M; 12-18 years; Normal weight + obesity + overweight; Other conditions: NR; n = 311. | 9 | NR | Adolescents | Resistance training 12-16 weeks; Resistance training + nutritional education 16 weeks. |
| Zabatiero et al., 2018 | Australia | SRMA | F and M; 6-12 years; overweight + obesity; Other conditions: NR; n = 1252. | 9 | School | Children and parents | School-based intervention 20 weeks; Behavior + physical activity 34 weeks; Sedentary behavior + physical activity + diet 26 weeks; control 2-=34 weeks. |
| Li et al., 2022 | China | SRMA | F and M; 6-18 years; Overweight + obesity; Other conditions: NR; n = 915. | 8 | NR | Children and adolescents | Diet + physical activity 6-52 weeks; Physical activity + health education 8-24 weeks; Physical activity 12 weeks; Control 6-52 weeks. |

| Study | Country | Design | Participants | Included RCTs (n) | Setting | Who received the intervention | Intervention(s) |
|---|---|---|---|---|---|---|---|
| Colquitt et al., 2016 | United Kingdom | SRMA | F and M; 0-6 years; Obesity + overweight; Other conditions: NR; n = 923. | 7 | Outpatient setting, primary care, community, home and obesity research clinic | Children and parents | Nutritional + Physical Activity + Behavioral components 26-156 weeks; usual care 16-156 weeks. |
| Kemp et al., 2021 | United Kingdom | SR | F and M;9-16 years; Normal weight + overweight; other conditions = NR. n = 1727 | 7 | Community-based setting; Usual Care; | Children, Adolescents and Parents | Family-based e-health 8-12 weeks; control 8-12weeks; |
| Medina-Blanco et al., 2011 | Mexico | SR | F and M; 5-12y; Normal weight + overweight/obesity; Other health conditions= NR; n= 1263 | 7 | NR | Children and parents | Educational (lessons about healthy eating and increased PA); PA (increased intensity and time of PA); PA + educational; 2-3 days/wk, 45 – 180'/wk |
| van Wijnen et al., 2009 | Netherlands | SR | F and M; 5-18y; Overweight/obesity; Other health conditions = NR; n= 2502 | 7 | School | Children and adolescents | Educational (behavioral change about diet and PA level); Diet (change in snacks); PA (resistance exercises); 16 weeks to 3 years |
| Andrade et al., 2018 | United States of America | SR | F and M; 6-12y; Overweight/Obesity; Other health conditions = NR; n = 10.365 | 6 | School | Children and parents | Nutritional education, Physical education, Physical activity, modified cafeteria offering, nutrition/lifestyle e/curriculum; 43 – 156 weeks; 96 weeks. |

| Study | Country | Design | Participants | Included RCTs (n) | Setting | Who received the intervention | Intervention(s) |
|---|---|---|---|---|---|---|---|
| Dias et al., 2018 | Brazil | SR | F and M; 8-15y; Overweight/obesity; Other health condition= NR; n= 2484 | 6 | School; Home; Medical center | Children | Educational (games about diet and exercise); 9-25 sessions, 25-40'/session, 1-6 months |
| Aceves-Martins et al., 2022 | United Kingdom | SRMA | F and M; 8.8 -15.8 years; Obesity + Overweight. Other conditions: Diabetes Mellitus. N = 2302. | 4 | - Public secondary school in a municipality in the urban area; - Public primary care unit within a secondary care hospital; - Pediatric obesity clinic at a public hospital; - Family medicine unit of a public hospital; - Public elementary school; - Public children's hospital; - Clinical nutrition research unit of a public hospital; - Public family medicine unit; - Pediatric clinic of a third-level hospital; - Private school | Children and adolescents | Nutritional Component: 4-52 weeks; PA component 4-52 weeks; Psychological or behavioral component: 2-12 weeks, And others. |
| Jull and Chen., 2013 | New Zealand | SRMA | F and M; 6-14y; Overweight/obesity; Other health conditions = NR; n=266 | 4 | University clinics; Community offices; Psychiatric outpatient clinic | Children and parents | Educational (PA; diet; behavioral skills): 10 – 120'/day, 10 weeks to 6 months |

| Study | Country | Design | Participants | Included RCTs (n) | Setting | Who received the intervention | Intervention(s) |
|---|---|---|---|---|---|---|---|
| Nagle et al., 2013 | United States of America | SR | F and M; 9.9-16.9y; Overweight/obesity; Other health condition= NR; n= NR | 4 | Health clinics; Medical offices | Children and adolescents | Educational (multidisciplinary intervention consisting of therapy sessions and counseling about diet and PA); Educational + PA (aerobic and recreational exercises); Diet (individualized diets); Diet + PA; 3 to 12 months |

NR: Not reported; SR: Systematic review; SRMA: Systematic review and Meta-analysis; RCT: Randomized controlled trial; F: female; M: male; HIIT: High-intensity interval training; MICT: Moderate-intensity continuous training; MIIT: Moderate-intensity interval training; LIIE: Low-intensity interval exercise; HIIE: High-intensity interval exercise; HRmax: Heart rate maximum; VO2peak: Peak oxygen uptake; VO2max: Maximum oxygen uptake; 1RM: One repetition maximum; NUT: Nutrition; DDR: Dance Dance Revolution; AVG: Active video game; PA: Physical activity; D: Diet; BD: Dietary behavior; BS: Sedentary behavior.

**Table S6. Frequency of all unique outcomes and domains.**

| Domains | Cumulative outcome frequency, N | Subdomains | Outcomes | Frequency, N | Frequency, % (reviews)* |
|---|---|---|---|---|---|
| General | 251 | body composition | Body Mass Index (BMI) | 72 | 52.6 |
| | | | Body weight | 39 | 28.5 |
| | | | Body fat | 33 | 24.1 |
| | | | Body Fat-free mass | 29 | 21.2 |
| | | | Skinfold thickness | 16 | 11.7 |
| | | | Waist Circumference | 15 | 10.9 |
| | | | Regional fat | 13 | 9.5 |
| | | | Body mass | 7 | 5.1 |
| | | | Height | 6 | 4.4 |
| | | | Obesity/Overweight prevalence | 6 | 4.4 |
| | | | Waist-to-hip ratio | 5 | 3.6 |
| | | | Hip circumference | 3 | 2.2 |
| | | | Subcutaneous adipose tissue | 3 | 2.2 |
| | | | Parents' BMI | 2 | 1.5 |
| | | | Body circumferences | 1 | 0.7 |
| | | | Waist-to-height ratio | 1 | 0.7 |
| Physical Functioning | 128 | | Time spent in physical activity | 17 | 12.4 |
| | | | Muscle strength | 14 | 10.2 |

| Domains | Cumulative outcome frequency, N | Subdomains | Outcomes | Frequency, N | Frequency, % (reviews)* |
|---|---|---|---|---|---|
| | | | Physical activity level | 12 | 8.8 |
| | | | Sedentary behavior | 12 | 8.8 |
| | | | Cardiorespiratory Fitness | 11 | 8.0 |
| | | | Maximal Oxygen Uptake (VO2max) | 8 | 5.8 |
| | | | Peak Oxygen Uptake (VO2peak) | 7 | 5.1 |
| | | | Agility | 6 | 4.4 |
| | | | Healthy lifestyle | 5 | 3.6 |
| | | | Flexibility | 4 | 2.9 |
| | | | Aerobic fitness | 3 | 2.2 |
| | | | Balance | 3 | 2.2 |
| | | | Jump ability | 3 | 2.2 |
| | | | Peak muscle power | 3 | 2.2 |
| | | | Energy expenditure in physical activity | 2 | 1.5 |
| | | | Maximal power output | 2 | 1.5 |
| | | | Muscle endurance | 2 | 1.5 |
| | | | Muscle Power | 2 | 1.5 |
| | | | Physical activity behavior | 2 | 1.5 |
| | | | Physical performance | 2 | 1.5 |

| Domains | Cumulative outcome frequency, N | Subdomains | Outcomes | Frequency, N | Frequency, % (reviews)* |
|---|---|---|---|---|---|
| | | | Explosive power | 1 | 0.7 |
| | | | Fatigue | 1 | 0.7 |
| | | | Maximum speed | 1 | 0.7 |
| | | | Mean power | 1 | 0.7 |
| | | | Motor competence | 1 | 0.7 |
| | | | Physical activity playing active and non-active videogames | 1 | 0.7 |
| | | | Running economy | 1 | 0.7 |
| | | | Sprint performance | 1 | 0.7 |
| Blood and Lymphatic System | 118 | lipid profile | High-density lipoproteins (HDL) | 31 | 22.6 |
| | | | Total Cholesterol | 28 | 20.4 |
| | | | Low-density lipoproteins (LDL) | 26 | 19.0 |
| | | | Triglycerides | 25 | 18.2 |
| | | | Non-High-density lipoproteins (non–HDL-C) | 3 | 2.2 |
| | | | Lipid profile | 2 | 1.5 |
| | | | Total cholesterol to HDL ratio | 1 | 0.7 |
| | | | Number of erythrocytes | 1 | 0.7 |
| | | | Plasma malondialdehyde | 1 | 0.7 |

| Domains | Cumulative outcome frequency, N | Subdomains | Outcomes | Frequency, N | Frequency, % (reviews)* |
|---|---|---|---|---|---|
| Cardiac | 94 | blood pressure | Systolic blood pressure (SBP) | 31 | 22.6 |
| | | | Diastolic blood pressure (DBP) | 27 | 19.7 |
| | | | Blood pressure | 8 | 5.8 |
| | | | Mean systolic and diastolic blood pressure | 4 | 2.9 |
| | | | Mean arterial blood pressure | 1 | 0.7 |
| | | | Maximum Heart Rate (HRmax) | 5 | 3.6 |
| | | | Resting and maximal heart rate | 5 | 3.6 |
| | | | Heart rate variability | 4 | 2.9 |
| | | | Heart rate recovery | 2 | 1.5 |
| | | | Mean Respiratory Rate | 2 | 1.5 |
| | | | Sympathetic activity | 2 | 1.5 |
| | | | Cardiac output | 1 | 0.7 |
| | | | Parasympathetic activity | 1 | 0.7 |
| | | | Ventilatory threshold | 1 | 0.7 |
| Emotional Functioning/Wellbeing | 37 | | Self-esteem | 9 | 6.6 |
| | | | Self-efficacy | 4 | 2.9 |
| | | | Self-perception | 4 | 2.9 |
| | | | Self-worth (self-perception) | 3 | 2.2 |

| **Domains** | **Cumulative outcome frequency, N** | **Subdomains** | **Outcomes** | **Frequency, N** | **Frequency, % (reviews)*** |
|---|---|---|---|---|---|
| | | | Intrinsic motivation | 2 | 1.5 |
| | | | Mood | 2 | 1.5 |
| | | | PA enjoyment | 2 | 1.5 |
| | | | Alcohol and cigarette use | 1 | 0.7 |
| | | | Attitudes about appearance | 1 | 0.7 |
| | | | Body image (self-perception) | 1 | 0.7 |
| | | | Media internalization (self-perception) | 1 | 0.7 |
| | | | Parents' alcohol use | 1 | 0.7 |
| | | | Physical self-perception or body satisfaction | 1 | 0.7 |
| | | | Positive expectations | 1 | 0.7 |
| | | | Psychological attraction to exergame | 1 | 0.7 |
| | | | Psychological well-being | 1 | 0.7 |
| | | | Self-acceptance (self-perception) | 1 | 0.7 |
| | | | Social-emotional development | 1 | 0.7 |
| Endocrine | 117 | glucose metabolism | Fasting blood glucose | 13 | 9.5 |
| | | | Fasting insulin | 9 | 6.6 |
| | | | Glucose | 8 | 5.8 |

| **Domains** | **Cumulative outcome frequency, N** | **Subdomains** | **Outcomes** | **Frequency, N** | **Frequency, % (reviews)*** |
|---|---|---|---|---|---|
| | | | Homeostatic Model Assessment-Insulin Resistance (HOMA-IR) | 7 | 5.1 |
| | | | Insulin resistance | 7 | 5.1 |
| | | | Insulin levels | 4 | 2.9 |
| | | | Glycated Haemoglobin (HbA1c) | 3 | 2.2 |
| | | | Insulin sensitivity | 2 | 1.5 |
| | | | Insulin-like Growth Factor (IGF-1) | 2 | 1.5 |
| | | | Blood insulin | 1 | 0.7 |
| | | | Insulin-like Growth Factor Binding Protein-3 (IGFBP-3) | 1 | 0.7 |
| | | | Non-fasting glucose | 1 | 0.7 |
| | | | Non-fasting insulin | 1 | 0.7 |
| | | | Serum insulin | 1 | 0.7 |
| | | | Adiponectin | 9 | 6.6 |
| | | | Leptin levels | 9 | 6.6 |
| | | | C-reactive protein | 6 | 4.4 |
| | | | Interleukin-6 (IL-6) | 6 | 4.4 |
| | | | Tumor Necrosis Factor (TNF-α) | 5 | 3.6 |
| | | | Resistin levels | 3 | 2.2 |

| Domains | Cumulative outcome frequency, N | Subdomains | Outcomes | Frequency, N | Frequency, % (reviews)* |
|---|---|---|---|---|---|
| | | | Adipocyte fat (aFABP) | 1 | 0.7 |
| | | | Alanine aminotransferase (ALT) | 1 | 0.7 |
| | | | Apolipoprotein A-1 | 1 | 0.7 |
| | | | Apolipoprotein B | 1 | 0.7 |
| | | | Aspartate aminotransferase (AST) | 1 | 0.7 |
| | | | Brain-derived neurotrophic factor (BDNF) | 1 | 0.7 |
| | | | Chemerin | 1 | 0.7 |
| | | | Fibrinogen level | 1 | 0.7 |
| | | | Intrahepatic fat | 1 | 0.7 |
| | | | Irisin | 1 | 0.7 |
| | | | Lactate threshold | 1 | 0.7 |
| | | | Metabolic syndrome | 1 | 0.7 |
| | | | Myostatin | 1 | 0.7 |
| | | | Plasminogen activator inhibitor-1 | 1 | 0.7 |
| | | | Proinflammatory cytokines | 1 | 0.7 |
| | | | Serum AgRP. NPY and α-MSH | 1 | 0.7 |
| | | | Serum interleukin-15 | 1 | 0.7 |
| | | | Superoxide dismutase | 1 | 0.7 |

| **Domains** | **Cumulative outcome frequency, N** | **Subdomains** | **Outcomes** | **Frequency, N** | **Frequency, % (reviews)*** |
|---|---|---|---|---|---|
| | | | Visfatin levels | 1 | 0.7 |
| Metabolism and Nutrition | 28 | food intake | Food intake | 9 | 6.6 |
| | | | Fruit and vegetable intake | 2 | 1.5 |
| | | | Portions of fruit. vegetables. and water | 2 | 1.5 |
| | | | Dietary behavior | 9 | 6.6 |
| | | | Eating disorders | 3 | 2.2 |
| | | | Food desire | 1 | 0.7 |
| | | | Gamma-glutamyl transferase (GGT) | 1 | 0.7 |
| | | | Self-report of food servings | 1 | 0.7 |
| Global Quality of Life | 13 | | Quality of Life | 7 | 5.1 |
| | | | Health-related Quality of Life | 6 | 4.4 |
| Psychiatric | 11 | | Depression | 7 | 5.1 |
| | | | Anxiety | 4 | 2.9 |
| Social Functioning | 10 | | Relationship with parents | 3 | 2.2 |
| | | | Social acceptance | 2 | 1.5 |
| | | | Aggression (self-perception) | 1 | 0.7 |
| | | | Parent acceptance | 1 | 0.7 |
| | | | Parenting skill and relationships | 1 | 0.7 |

| Domains | Cumulative outcome frequency, N | Subdomains | Outcomes | Frequency, N | Frequency, % (reviews)* |
|---|---|---|---|---|---|
| | | | Social stress | 1 | 0.7 |
| | | | Strength and difficulties perceived by parents | 1 | 0.7 |
| Cognitive Functioning | 8 | | Cognitive competence (self-perception) | 1 | 0.7 |
| | | | Cognitive flexibility | 1 | 0.7 |
| | | | Food knowledge | 1 | 0.7 |
| | | | Metacognition | 1 | 0.7 |
| | | | Non-executive cognitive function | 1 | 0.7 |
| | | | Physical activity knowledge | 1 | 0.7 |
| | | | Selective attention-inhibition | 1 | 0.7 |
| | | | Working memory | 1 | 0.7 |
| Vascular | 7 | | Flow-mediated dilation (FMD) | 2 | 1.5 |
| | | | Arterial stiffness | 1 | 0.7 |
| | | | Endothelial dysfunction | 1 | 0.7 |
| | | | Intima-media thickness (IMT) | 1 | 0.7 |
| | | | Vascular conductance | 1 | 0.7 |
| | | | Vascular resistance | 1 | 0.7 |
| Adverse Events | 6 | | Adverse events | 4 | 2.9 |
| | | | Morbidity | 2 | 1.5 |

| **Domains** | **Cumulative outcome frequency, N** | **Subdomains** | **Outcomes** | **Frequency, N** | **Frequency, % (reviews)*** |
|---|---|---|---|---|---|
| Perceived Health Status | 5 | | Perceived Athletic competence | 2 | 1.5 |
| | | | Perceived Physical condition | 2 | 1.5 |
| | | | Healthy attitudes (self-perception) | 1 | 0.7 |
| Musculoskeletal and Connective Tissue | 4 | | Total bone density | 2 | 1.5 |
| | | | Bone mineral content | 1 | 0.7 |
| | | | Regional bone density | 1 | 0.7 |
| Delivery of care | 4 | | Participants' views of the intervention | 1 | 0.7 |
| | | | Peer support | 1 | 0.7 |
| | | | Quality of care | 1 | 0.7 |
| | | | Unhealthy weight control behaviours (self-perception) | 1 | 0.7 |

* The percentages represent the frequency relative to the number of included reviews.
